# Supplementary material for: Systematic review of process evaluations of interventions in trials investigating sedentary behaviour in adults
Source: BMJ Open. 2022 Jan 25;12(1):e053945. doi: 10.1136/bmjopen-2021-053945 (PMC8804646; doi:10.1136/bmjopen-2021-053945)
Supplement: Supplementary data [file bmjopen-2021-053945supp002.pdf]

Supplementary file 2_search strategies May 11th 2020

Database: CINAHL (EBSCOhost), search modes - Boolean/Phrase, 1982-:

S1 (MH "Life Style, Sedentary")
S2 TI (sedentary or sitting or sedentariness or sedentarism)
S3 TX ((sedentary or sitting or seated) N5 (behavio* or lifestyle or life-style)))
S4 TX ((sedentary N3 (adult* or men or women or males or females or individuals or people or population*))
S5 TI (((sitting or sit or seated or stationary or standing) N3 (task* or time or bout* or work* or break*))) OR AB (((sitting or sit or seated or stationary or standing) N3 (task* or time or bout* or work* or break*)))
S6 TX((inactiv* or no exercise or nonexercise or non exercise) N3 (adult* or men or women or males or females or individuals or people))
S7 TX "low energy expenditure"
S8 TX "physical* inactiv*"
S9 TX (leisure time N5 ("physical* activ*" or passive or inactiv*)))
S10 TX "physical activity level*"
S11 TX ((sitting or lying) N2 posture*)
S12 TX (prolong* N2 (reclin* or sit or sitting or seated))
S13 TI((computer* or television or tv or video game? or videogame? or gaming) and (sedentary or "physical* activity*" or sitting or seated or underactiv* or under activ*))
S14 TX "chair rise*"
S15 TX "sit* less"
S16 TX ((light or low) N1 "physical activ*")
S17 TX ((decrease or reduc* or discourag* or lessen*) N3 (sit or sitting or stand or standing or "physical* inactiv*"))
S18 TX (time N5 (computer* or television or tv or "video game*" or videogame* or gaming or screen or media))
S19 TX ((watch* or view*) N5 (television or tv))
S20 TI (play* N5 ("video game*" or videogame* or "computer game*")) OR AB (play* N5 ("video game*" or videogame* or "computer game*"))
S21 TX allocat* random*
S22 (MH "Placebos")
S23 TX placebo*
S24 TX random* allocat*
S25 TX randomi* control* trial*
S26 TX clinic* n1 trial*
S27 PT Clinical trial
S28 (MH "Clinical Trials+")
S29 AB randomized
S30 AB randomly
S31 MH "Random Assignment"
S32 S21 or S22 or S23 or S24 or S25 or S26 or S27 or S28 or S29 or S30 or S31
S33 (MH "Program Evaluation")
S34 Tx ((process* evaluat*))
S35 TX ((program* evaluat*))
S36 (MH "Process Assessment (Health Care)")
S37 S1 OR S2 OR S3 OR S4 OR S5 OR S6 OR S7 OR S8 OR S9 OR S10 OR S11 OR S12 OR S13 OR S14 OR S15 OR S16 OR S17 OR S18 OR S19 OR S20
S38 S21 OR S22 OR S23 OR S24 OR S25 OR S26 OR S27 OR S28 OR S29 OR S30 OR S31
S39 S33 OR S34 OR S35 or S36
S40 S37 AND S38 AND S39

Database: SPORTDiscus (EBSCOhost), search modes - Boolean/Phrase:

S1 SU Sedentary Lifestyle
S2 TI (sedentary or sitting or sedentariness or sedentarism)
S3 TI ((sedentary or sitting or seated) N5 (behavio* or lifestyle or life-style))) OR AB (seated) N5 (behavio* or lifestyle or life-style)) OR AB ((sedentary or sitting or seated) N5 (behavio* or lifestyle or life-style))) OR AB (seated) N5 (behavio* or lifestyle or life-style))
S4 TI ((sedentary N3 (adult* or men or women or males or females or individuals or people or population*))) OR AB (

(sedentary N3 (adult* or men or women or males or females or individuals or people or population*)))
S5 TI (((sitting or sit or seated or stationary or standing) N3 (task* or time or bout* or work* or break*))) OR AB (((sitting or sit or seated or stationary or standing) N3 (task* or time or bout* or work* or break*)))
S6 TX(inactiv* or no exercise or nonexercise or non exercise) N3 (adult* or men or women or males or females or individuals or people))
S7 TI "low energy expenditure" OR AB "low energy expenditure"
S8 TI "physical* inactiv*" OR AB "physical* inactiv*"
S9 TI (leisure time N5 ("physical* activ*" or passive or inactiv*))) OR AB (leisure time N5 ("physical* activ*" or passive or inactiv*))
S10 TI "physical activity level*" OR AB "physical activity level*"
S11 TI ((sitting or lying) N2 posture*) OR AB ((sitting or lying) N2 posture*)
S12 TI (prolong* N2 (reclin* or sit or sitting or seated)) OR AB (prolong* N2 (reclin* or sit or sitting or seated))
S13 TX((computer* or television or tv or video game? or videogame? or gaming) and (sedentary or physical* activity* or sitting or seated or underactiv* or under activ*))
S14 TI "chair rise*" OR AB "chair rise*"
S15 TI "sit* less" OR AB "sit* less"
S16 TI ((light or low) N1 "physical activ*") OR AB ((light or low) N1 "physical activ*")
S17 TI ((decrease or reduc* or discourag* or lessen*) N3 (sit or sitting or stand or standing or "physical* inactiv*")) OR AB ((decrease or reduc* or discourag* or lessen*) N3 (sit or sitting or stand or standing or "physical* inactiv*"))
S18 TI (time N5 (computer* or television or tv or "video game*" or videogame* or gaming or screen or media)) OR AB (time N5 (computer* or television or tv or "video game*" or videogame* or gaming or screen or media))
S19 TI ((watch* or view*) N5 (television or tv)) OR AB ((watch* or view*) N5 (television or tv))
S20 TI (play* N5 ("video game*" or videogame* or "computer game*")) OR AB (play* N5 ("video game*" or videogame* or "computer game*"))
S21 ((DE "RANDOMIZED controlled trials")))
S22 TX allocat* random*
S23 DE "QUANTITATIVE research"
S24 DE "PLACEBOS (Medicine)"
S25 TX placebo*
S26 TX random* allocat*
S27 TX random* assign*
S28 TX randomi* control* trial*
S29 TX clinic* n1 trial*
S30 DE "CLINICAL trials"
S31 AB randomly
S32 AB randomized
S33 S21 OR S22 OR S23 OR S24 OR S25 OR S26 OR S27 OR S28 OR S29 OR S30 OR S31 OR S32
S34 SU program evaluation
S35 TX program* evaluat*
S36 TI process* evaluat*
S37 S34 OR S35 OR S36
S38 S1 OR S2 OR S3 OR S4 OR S5 OR S6 OR S7 OR S8 OR S9 OR S10 OR S11 OR S12 OR S13 OR S14 OR S15 OR S16 OR S17 OR S18 OR S19 OR S20
S39 S33 AND S37 AND S38

Database: Cochrane Database of Systematic Reviews (Wiley):

#1 MeSH descriptor: [Sedentary Behavior] this term only
#2 sedentary or sitting or sedentariness or sedentarism:ti
#3 (sedentary or sitting or seated) near/5 (behavio* or lifestyle or life-style):ti,ab,kw (Word variations have been searched)
#4 sedentary near/3 (adult* or men or women or males or females or individuals or people or population*):ti,ab,kw (Word variations have been searched)
#5 (sitting or sit or seated or stationary or standing) near/3 (task* or time or bout* or work* or break*):ti,ab,kw (Word variations have been searched)
#6 ((inactiv* or no exercise or nonexercise or non exercise) near/3 (adult* or men or women or males or females or individuals or people)):ti,ab,kw
#7 "low energy expenditure":ti,ab,kw (Word variations have been searched)
#8 ("physical* inactive" or "physical inactivity"):ti,ab,kw (Word variations have been searched)
#9 "leisure time" near/5 ("physical* activ*" or passive or inactiv*):ti,ab,kw (Word variations have been searched)

#10 "physical activity level*":ti,ab,kw (Word variations have been searched)
#11 (sitting or lying) near/2 posture*:ti,ab,kw (Word variations have been searched)
#12 prolong* near/2 (reclin* or sit or sitting or seated):ti,ab,kw (Word variations have been searched)
#13 "chair rise*":ti,ab,kw (Word variations have been searched)
#14 "sit* less":ti,ab,kw (Word variations have been searched)
#15 (light or low) near/1 "physical activ*":ti,ab,kw (Word variations have been searched)
#16 time near/5 (computer* or television or tv or "video game*" or videogame* or gaming or screen or media):ti,ab,kw (Word variations have been searched)
#17 (watch* or view*) near/5 (television or tv):ti,ab,kw (Word variations have been searched)
#18 play* near/5 ("video game*" or videogame* or "computer game*"):ti,ab,kw (Word variations have been searched)
#19 (decrease or reduc* or discourag* or lessen*) near/3 (sit or sitting or stand or standing or "physical* inactiv*"):ti,ab,kw (Word variations have been searched)
#20 ((computer* or television or tv or video game* or videogame* or gaming) and (sedentary or physical* activity* or sitting or seated or underactiv* or under activ*)):ti
#21 {or #1-#20}
#22 MeSH descriptor: [Program Evaluation] this term only
#23 ("program* evaluation*"):ti,ab,kw
#24 "process* evaluation*":ti,ab,kw
#25 MeSH descriptor: [Process Assessment, Health Care] this term only
#26 {or #22-#25}
#27 #21 and #26

Database: Cochrane Central Register of Controlled Trials (Wiley):

#1 MeSH descriptor: [Sedentary Behavior] this term only
#2 sedentary or sitting or sedentariness or sedentarism:ti
#3 (sedentary or sitting or seated) near/5 (behavio* or lifestyle or life-style):ti,ab,kw (Word variations have been searched)
#4 sedentary near/3 (adult* or men or women or males or females or individuals or people or population*):ti,ab,kw (Word variations have been searched)
#5 (sitting or sit or seated or stationary or standing) near/3 (task* or time or bout* or work* or break*):ti,ab,kw (Word variations have been searched)
#6 ((inactiv* or no exercise or nonexercise or non exercise) near/3 (adult* or men or women or males or females or individuals or people)):ti,ab,kw
#7 "low energy expenditure":ti,ab,kw (Word variations have been searched)
#8 ("physical* inactive" or "physical inactivity"):ti,ab,kw (Word variations have been searched)
#9 "leisure time" near/5 ("physical* activ*" or passive or inactiv*):ti,ab,kw (Word variations have been searched)
#10 "physical activity level*":ti,ab,kw (Word variations have been searched)
#11 (sitting or lying) near/2 posture*:ti,ab,kw (Word variations have been searched)
#12 prolong* near/2 (reclin* or sit or sitting or seated):ti,ab,kw (Word variations have been searched)
#13 "chair rise*":ti,ab,kw (Word variations have been searched)
#14 "sit* less":ti,ab,kw (Word variations have been searched)
#15 (light or low) near/1 "physical activ*":ti,ab,kw (Word variations have been searched)
#16 time near/5 (computer* or television or tv or "video game*" or videogame* or gaming or screen or media):ti,ab,kw (Word variations have been searched)
#17 (watch* or view*) near/5 (television or tv):ti,ab,kw (Word variations have been searched)
#18 play* near/5 ("video game*" or videogame* or "computer game*"):ti,ab,kw (Word variations have been searched) 478
#19 (decrease or reduc* or discourag* or lessen*) near/3 (sit or sitting or stand or standing or "physical* inactiv*"):ti,ab,kw (Word variations have been searched)
#20 ((computer* or television or tv or video game* or videogame* or gaming) and (sedentary or physical* activity* or sitting or seated or underactiv* or under activ*)):ti
#21 {or #1-#20}
#22 MeSH descriptor: [Program Evaluation] this term only
#23 ("program* evaluation*"):ti,ab,kw
#24 "process* evaluation*":ti,ab,kw
#25 MeSH descriptor: [Process Assessment, Health Care] this term only
#26 {or #22-#25}
#27 #21 and #26

AMED (Allied and Complementary Medicine) (OVID) <1985 to May 2020>:

1 Sedentary Lifestyle/
2 (sedentary or sitting or sedentariness or sedentarism).ti.
3 ((sedentary or sitting or seated) adj5 (behavio* or lifestyle or life-style)).tw.
4 ((inactiv* or no exercise or nonexercise or non exercise) adj3 (adult? or men or women or males or females or individuals or people)).tw.
5 (sedentary adj3 (adult? or men or women or males or females or individuals or people or population?)).tw.
6 ((sitting or sit or seated or stationary or standing) adj3 (task* or time or bout* or work* or break*)).tw.
7 low energy expenditure.tw.
8 physical* inactiv*.tw.
9 (leisure time adj5 (physical* activ* or passive or inactiv*)).tw.
10 "physical activity level*".tw.
11 ((sitting or lying) adj2 posture*).tw.
12 (prolong* adj2 (reclin* or sit or sitting or seated)).tw.
13 chair rise?.tw.
14 "sit* less".tw.
15 ((light or low) adj "physical activ*").tw.
16 ((decrease or reduc* or discourag* or lessen*) adj3 (sit or sitting or stand or standing or physical* inactiv*)).tw.
17 (time adj5 (computer* or television or tv or video game? or videogame? or gaming or screen or media)).tw.
18 ((watch* or view*) adj5 (television or tv)).tw.
19 (play* adj5 (video game? or videogame? or computer game?)).tw.
20 ((computer* or television or tv or video game? or videogame? or gaming) and (sedentary or physical* activity* or sitting or seated or underactiv* or under activ*)).ti.
21 or/1-20 [sedentary behaviour terms]
22 process evaluat*.mp.
23 "Outcome and Process Assessment"/
24 program evaluat*.mp.
25 or/22-24 [process evaluation]
26 21 and 25 [sedentary behaviour and process evaluation]

<u>Database: Embase Classic+Embase (OVID) <1947 to 2020 May 08>:</u>

1 Sedentary Lifestyle/
2 (sedentary or sitting or sedentariness or sedentarism).ti.
3 ((sedentary or sitting or seated) adj5 (behavio* or lifestyle or life-style)).tw.
4 ((inactiv* or no exercise or nonexercise or non exercise) adj3 (adult? or men or women or males or females or individuals or people)).tw.
5 (sedentary adj3 (adult? or men or women or males or females or individuals or people or population?)).tw.
6 ((sitting or sit or seated or stationary or standing) adj3 (task* or time or bout* or work* or break*)).tw.
7 low energy expenditure.tw.
8 physical* inactiv*.tw.
9 (leisure time adj5 (physical* activ* or passive or inactiv*)).tw.
10 "physical activity level*".tw.
11 ((sitting or lying) adj2 posture*).tw.
12 (prolong* adj2 (reclin* or sit or sitting or seated)).tw.
13 chair rise?.tw.
14 "sit* less".tw.
15 ((light or low) adj "physical activ*").tw.
16 ((decrease or reduc* or discourag* or lessen*) adj3 (sit or sitting or stand or standing or physical* inactiv*)).tw.
17 (time adj5 (computer* or television or tv or video game? or videogame? or gaming or screen or media)).tw.
18 ((watch* or view*) adj5 (television or tv)).tw.
19 (play* adj5 (video game? or videogame? or computer game?)).tw.
20 ((computer* or television or tv or video game? or videogame? or gaming) and (sedentary or physical* activity* or sitting or seated or underactiv* or under activ*)).ti.
21 or/1-20 [sedentary behaviour terms]
22 Randomized controlled trial/
23 Controlled clinical study/
24 22 or 23
25 Random*.tw.
26 randomization/
27 intermethod comparison/
28 placebo.tw.

29 (compare or compared or comparison).ti.
30 ((evaluated or evaluate or evaluating or assessed or assess) and (compare or compared or comparing or comparison)).ab.
31 (open adj label).tw.
32 ((double or single or doubly or singly) adj (blind or blinded or blindly)).tw.
33 double blind procedure/
34 parallel group*1.tw.
35 (crossover or cross over).tw.
36 ((assign* or match or matched or allocation) adj5 (alternate or group*1 or intervention*1 or patient*1 or subject*1 or participant*1)).tw.
37 (assigned or allocated).tw.
38 (controlled adj7 (study or design or trial)).tw.
39 (volunteer or volunteers).tw.
40 human experiment/
41 trial.ti.
42 or/25-41
43 42 or 24
44 (random* adj sampl* adj7 ("cross section*" or questionnaire*1 or survey* or database*1)).tw. not (comparative study/ or controlled study/ or randomi?ed controlled.tw. or randomly assigned.tw.)
45 Cross-sectional study/ not (randomized controlled trial/ or controlled clinical study/ or controlled study/ or randomi?ed controlled.tw. or control group*1.tw.)
46 (((case adj control*) and random*) not randomi?ed controlled).tw.
47 (Systematic review not (trial or study)).ti.
48 (nonrandom* not random*).tw.
49 "Random field*".tw.
50 (random cluster adj3 sampl*).tw.
51 (review.ab. and review.pt.) not trial.ti.
52 "we searched".ab. and (review.ti. or review.pt.)
53 "update review".ab.
54 (databases adj4 searched).ab.
55 (rat or rats or mouse or mice or swine or porcine or murine or sheep or lambs or pigs or piglets or rabbit or rabbits or cat or cats or dog or dogs or cattle or bovine or monkey or monkeys or trout or marmoset*1).ti. and animal experiment/ (1058538)
56 Animal experiment/ not (human experiment/ or human/)
57 or/44-56
58 43 not 57 [Cochrane Embase RTC search filter Jan 2015]
59 program evaluat*.mp.
60 health care quality/
61 process* evaluat*.mp.
62 or/59-61 [process evaluation]
63 21 and 58 and 62 [sedentary behaviour and RCTs and process evaluations]
64 remove duplicates from 63

Database: APA PsycInfo (OVID) <1806 to May Week 1 2020>:

1 SEDENTARY BEHAVIOR/
2 (sedentary or sitting or sedentariness or sedentarism).ti.
3 ((sedentary or sitting or seated) adj5 (behavio* or lifestyle or life-style)).tw.
4 ((inactiv* or no exercise or nonexercise or non exercise) adj3 (adult? or men or women or males or females or individuals or people)).tw.
5 (sedentary adj3 (adult? or men or women or males or females or individuals or people or population?)).tw.
6 ((sitting or sit or seated or stationary or standing) adj3 (task* or time or bout* or work* or break*)).tw.
7 low energy expenditure.tw.
8 physical* inactiv*.tw.
9 (leisure time adj5 (physical* activ* or passive or inactiv*)).tw.
10 "physical activity level*".tw.
11 ((sitting or lying) adj2 posture*).tw.
12 (prolong* adj2 (reclin* or sit or sitting or seated)).tw.
13 chair rise?.tw.
14 "sit* less".tw.
15 ((light or low) adj "physical activ*").tw.
16 ((decrease or reduc* or discourag* or lessen*) adj3 (sit or sitting or stand or standing or physical* inactiv*)).tw.
17 (time adj5 (computer* or television or tv or video game? or videogame? or gaming or screen or media)).tw.

18 ((watch* or view*) adj5 (television or tv)).tw.
19 (play* adj5 (video game? or videogame? or computer game?)).tw.
20 ((computer* or television or tv or video game? or videogame? or gaming) and (sedentary or physical* activity* or sitting or seated or underactiv* or under activ*)).ti.
21 or/1-20 [sedentary behaivour]
22 Treatment Effectiveness Evaluation/
23 exp Treatment Outcomes/
24 Psychotherapeutic Outcomes/
25 PLACEBO/
26 exp Followup Studies/
27 placebo*.tw.
28 random*.tw.
29 comparative stud*.tw.
30 (clinical adj3 trial*).tw.
31 (research adj3 design).tw.
32 (evaluat* adj3 stud*).tw.
33 (prospectiv* adj3 stud*).tw.
34 ((singl* or doubl*or trebl* or tripl*) adj3 (blind* or mask*)).tw.
35 or/22-34 [RCT filter adapted from Watson RJ, Richardson PH 1999]
36 program evaluat*.mp.
37 process* evaluat*.mp.
38 evaluation/
39 or/36-38 [process evaluation terms]
40 21 and 35 and 39 [sedentary behaviour and rcts and process evalutions]

<u>Database: Ovid MEDLINE(R) and Epub Ahead of Print, In-Process & Other Non-Indexed Citations and Daily <1946 to May 08, 2020>:</u>

1 Sedentary Lifestyle/
2 (sedentary or sitting or sedentariness or sedentarism).ti.
3 ((sedentary or sitting or seated) adj5 (behavio* or lifestyle or life-style)).tw.
4 ((inactiv* or no exercise or nonexercise or non exercise) adj3 (adult? or men or women or males or females or individuals or people)).tw.
5 (sedentary adj3 (adult? or men or women or males or females or individuals or people or population?)).tw.
6 ((sitting or sit or seated or stationary or standing) adj3 (task* or time or bout* or work* or break*)).tw.
7 low energy expenditure.tw.
8 physical* inactiv*.tw.
9 (leisure time adj5 (physical* activ* or passive or inactiv*)).tw.
10 "physical activity level*".tw.
11 ((sitting or lying) adj2 posture*).tw.
12 (prolong* adj2 (reclin* or sit or sitting or seated)).tw.
13 chair rise?.tw.
14 "sit* less".tw.
15 ((light or low) adj "physical activ*").tw.
16 ((decrease or reduc* or discourag* or lessen*) adj3 (sit or sitting or stand or standing or physical* inactiv*)).tw.
17 (time adj5 (computer* or television or tv or video game? or videogame? or gaming or screen or media)).tw.
18 ((watch* or view*) adj5 (television or tv)).tw.
19 (play* adj5 (video game? or videogame? or computer game?)).tw.
20 ((computer* or television or tv or video game? or videogame? or gaming) and (sedentary or physical* activity* or sitting or seated or underactiv* or under activ*)).ti.
21 or/1-20 [sedentary behaviour terms]
22 Program Evaluat*.mp. (62861)
23 "Outcome and Process Assessment (Health Care)"/
24 "Process Assessment (Health Care)"/
25 process evaluat*.mp.
26 or/22-25 [process evaluation]
27 randomized controlled trial.pt.
28 controlled clinical trial.pt.
29 randomized.ab.
30 placebo.ab.
31 drug therapy.fs.

32 randomly.ab.
33 trial.ab.
34 groups.ab.
35 27 or 28 or 29 or 30 or 31 or 32 or 33 or 34
36 exp animals/ not humans.sh.
37 35 not 36 [Cochrane RCT filter 2008, sensitivity maximimising]
38 21 and 26 and 37 [sedentary behaviour and process evaluation and RCTs]

Database: Web of Science: Indexes=SCI-EXPANDED, SSCI, CPCI-S, CPCI-SSH, ESCI (Clarivate), Timespan= 1900-2020:

1 TI=((sedentary or sitting or sedentariness or sedentarism))
2 TS=(((sedentary or sitting or seated) NEAR/5 (behavio* or lifestyle or life-style)))
3 TS=((inactive* or "non exercise" or "nonexercise" or "no exercise") near/3 (adult* or men or women or males or females or individuals or people))
4 TS=((sedentary) near/3 (adult* or men or women or males or females or individuals or people or population*))
5 TS=(("leisure time" NEAR/5 ("physical* activ*" or passive or inactiv*)))
6 TS=("physical activity level*" or "physical* inactiv*")
7 TOPIC: (((sitting or lying) near/2 posture*))
8 TOPIC: ((nonexercis* or "non exercis*" or "no exercis*")
9 TOPIC: ("chair rise")
10 TS=((sitting or sit or seated or stationary or standing) NEAR/3 (task* or time or bout* or work* or break*))
11 TS=("sit* less")
12 TOPIC: (((light or low) near/1 "physical activ*"))
13 TS=((decrease or reduc* or discourag* or lessen*) NEAR/3((sit or sitting or stand or standing or "physical* inactiv*")))
14 TS=(time NEAR/5 (computer* or television or tv or "video game*" or videogame* or gaming or screen or media))
15 TS=((watch* or view*) NEAR/5 (television or tv))
16 TS= (play* NEAR/5 ("video game*" or "videogame*" or "computer game*"))
17 TI=((computer* or television or tv or "video game?" or videogame? or gaming) and (sedentary or "physical* activity*" or sitting or seated or underactiv* or under activ*))
18 TOPIC: (random* or RCT or placebo or clinical Near/1 trial*)
19 TS=("program* evaluat*")
20 TS=("process evaluat*")
21 #20 OR #19
22 #1 or #2 or #3 or #4 or #5 or #6 or #7 or #8 or #9 or #10 or #11 or #12 or #13 or #14 or #15 or #16 or #17
23 #22 AND #21 AND #18

Databases: ProQuest Dissertations & Theses A&I, from January 01, 1990 to March 15, 2019:

ti((computer* OR television OR tv OR "video game" OR "videogame*" OR gaming) AND (sedentary OR physical* activity* OR sitting OR seated OR underactiv* OR under activ*)) OR ti(sedentary OR sitting OR elementariness OR sedentary OR (sedentary OR sitting OR seated) N5 (behavio* OR lifestyle OR life-style)) OR ti((sitting OR sit OR seated OR stationary OR standing) N3 (task* OR time OR bout* OR work* OR break*)) OR ti("physical* inactiv*" OR "chair rise*" OR "low energy expenditure" OR "sit less") OR ti((watch* OR view*) N5 (television OR tv)) OR ti(play* N5 ("video game*" OR videogame* OR "computer game*")) OR ti(time N5 (computer* OR television OR tv OR "video game*" OR videogame* OR gaming OR screen OR media)) OR ti((computer* OR television OR tv OR "video game" OR "videogame*" OR gaming) AND (sedentary OR physical* activity* OR sitting OR seated OR underactiv* OR "under activ*")) AND ti("process* evaluation*" OR "program* evaluation*") AND ti(Random* OR RCT OR clinical N1 trial*)
