# Supplementary material for: Systematic review of process evaluations of interventions in trials investigating sedentary behaviour in adults
Source: BMJ Open. 2022 Jan 25;12(1):e053945. doi: 10.1136/bmjopen-2021-053945 (PMC8804646; doi:10.1136/bmjopen-2021-053945)
Supplement: Supplementary data [file bmjopen-2021-053945supp003.pdf]

Supplementary file 3. Characteristics of 17 included RCTs_27.05.21

**Randomised Control Trials**									
**Study** *(Authors (Year)*, **Country** *(of process evaluation report)*	**Study aims**	**Inclusion/exclusion criteria**	**Sample size, n assigned to intervention /control**	**Participant characteristic** *(Age (mean (SD) or %), Gender (% female), Ethnicity)*	**Study design, RCT type, group, setting**	**Interventio n description** *(Content, duration)*	**Control description**	**Data collection and follow ups** *(time-points)*	**Outcome measures for treatment effects** *(identified in the study reports)*
**Adams (2012)**<br><br>USA	Reduce sedentary behaviour, increase light physical activity.<br><br>(Feasibility trial)	**Inclusion:**<br>1. Women between the ages of 35-85;<br>2. BMI >25;<br>3. Be willing to receive intervention materials and messages by email;<br>4. Plan to attend all program and data collection sessions.<br>**Exclusion:**<br>Any reported conditions that prohibited standing or walking.	75<br><br>I: 47<br>C: 28	**Age:**<br>I: 56.73 (12.64)<br>C: 61.38 (12.1)<br><br>**Gender:**<br>100%<br><br>**Ethnicity:**<br>89% Caucasian<br>11% African-American	Cluster randomised controlled<br><br>Weight-loss support club (cluster unit)	On Our Feet intervention – combination of 2 face-to-face interactive group sessions, and 6 weekly email messages.<br><br>6 weeks	Waiting list	Baseline<br>6 weeks	1. Time spent in SB; light and moderate PA (accelerometer; IPAQ, Godin Leisure-Time Activity Questionnaire);<br>2. Participant's self-rated level of confidence for reducing sitting and increasing PA behaviours;<br>3. BMI and waist circumference.
**Albright (2015)**<br><br>USA	Increase moderate to vigorous physical activity.	**Inclusion:**<br>1. Mother of infant aged 2-12 months;<br>2. Inactive (<30 minutes of MVPA/week);<br>3. Healthy, able to do moderate intensity physical activity;<br>4. BMI =18.5-40;<br>5. Not planning to become pregnant in the next 12 months;<br>6. Aged 18-45;<br>7. Had health insurance;<br>8. Read/understood	311<br><br>I: 154<br>C: 157	**Age:**<br>I: 31.6 (5.5)<br>C: 32.1 (5.9)<br><br>**Gender:**<br>100%<br><br>**Ethnicity:**<br>31.5% Native Hawaiian/ Pacific Islander<br>33.8% Asian (Japanese, Filipino, other	Randomised controlled<br><br>Parallel groups<br><br>Community	Tailored telephone counselling, information on website, and pedometer.<br><br>12 months	Information in print or standard website.	Baseline<br>1 month<br>3 months<br>6 months<br>12 months (immediatel after intervention<br>18 months	1. Time spent in MVPA (Active Australia Survey; accelerometer; exercise log);<br>2. Time spent sitting while travelling; at work; watching TV, etc. (Active Australia Survey);<br>3. Body mass index;<br>4. Self-efficacy for PA (instrument designed to assess self-confidence to overcome barriers to PA, modified with questions tailored to new mothers);<br>5. Psychosocial mediators

Supplementary file 3. Characteristics of 17 included RCTs_27.05.21

		English;<br>9. Physician's written approval if history of contraindicated conditions.<br>**Exclusion:**<br>1. Pregnant;<br>2. Planning to leave Oahu, Hawaii in the next year (permanently move away);<br>3. Diagnosis of cancer, coronary heart disease (including atrial fibrillation), insulin-dependent diabetes mellitus (IDDM), and other atherosclerotic cardiovascular diseases (e.g., stroke).		Asian)<br>16.4% Mixed race<br>15.1% White<br>2.6% Black/ Native American<br>0.6% Unknown					survey.
**Benedetti (2020)**<br><br>Brazil	Improve physical activity level.	**Inclusion:**<br>1. Aged ≥60;<br>2. No severe physical and/or mental health impairments;<br>3. Had not participated in physical activity programs in the past 6 months.<br>**Exclusion:**<br>History of heart attack and/or stroke in the past 6 months, cancer diagnosis and/or other severe medical conditions.	114<br><br>BCG: 36<br>TEG: 52<br>C: 26	**Age:**<br>BCG: 69.7 (6.9)<br>TEG: 71.3 (7.3)<br>C: 67.2 (5.8)<br><br>**Gender:**<br>80.7%<br><br>**Ethnicity:**<br>Not reported	Cluster randomised controlled<br><br>Public health centres (cluster unit)	BCG: 12 weekly meetings behavioural change programme that was adapted from "Active Living Every Day" from USA.<br><br>TEG: 12-week (3 times per week) exercise class conducted at	No intervention	Baseline<br>3 months<br>6 months<br>12 months	1. Time spent in SB; light PA; and MVPA (accelerometers);<br>2. BMI;<br>3. Quality of life (WHOQOL-BREF and WHOQOL-OLD).

Supplementary file 3. Characteristics of 17 included RCTs_27.05.21

						local HCs.			
**Berendsen (2015)**<br><br>The Netherlands	Improve physical activity and dietary behaviour.	**Inclusion:**<br>1. Weight-related health risk;<br>2. Inactive lifestyle (not doing 30 minutes moderate physical activity for at least 5 days per week);<br>3. Motivated for behavioural change;<br>4. BMI= 25-30, with a large waist circumference (men greater than 102 cm, women greater than 88 cm) with comorbidity (cardiovascular disease and/or T2DM, arthrosis and sleep apnoea), or<br>5. BMI= 30-35, with a normal or large waist circumference with comorbidity, or<br>6. BMI= 35-40, with a normal or large waist circumference with risk factors for cardiovascular disease or T2DM and without other comorbidities.<br>**Exclusion:**<br>1. Serious mobility limitations precluding participation;<br>2. Pregnancy.	411<br><br>I: 247<br>C: 164	**Age:**<br>I: 55.9 (12.3)<br>C: 53.8 (12.4)<br><br>**Gender:**<br>64.7%<br><br>**Nationality:**<br>88.8% Dutch	Cluster randomised controlled<br><br>GP practices (Cluster unit)	Supervised exercise programme based on BeweegKuur – individual and group meetings with lifestyle advisor, dietitian, and intensive support from physical therapist.<br><br>12 months	Start-up exercise programme based on BeweegKuur – same number of meetings with lifestyle advisor and dietitian as the intervention group, few numbers of meeting with physical therapist.<br><br>12 months	Activity monitor, physiological measures:<br>Baseline<br>12 months<br>24 months<br><br>IPAQ, dietary habits:<br>Baseline<br>6 months<br>12 months<br>18 months<br>24 months<br><br>EQ-6D, healthcare costs:<br>Baseline, then every 3 months until 24 months	1. Time spent PA (accelerometer; IPAQ), sedentary, standing or active (accelerometer);<br>2. Dietary habits;<br>3. Quality of Life (EQ-6D);<br>4. Medication;<br>5. Side-effects;<br>6. Direct and indirect costs;<br>7. Health risk, e.g. waist circumference, body composition, blood pressure, resting heart rate, blood biochemistry, and physical fitness.
**Biddle (2017)**	Reduce sitting time.	**Inclusion:**<br>1. Age 18-40, BMI ≥30	187	**Age:**<br>I: 32.4 (5.4)	Randomised controlled	STAND – A group-based	Information leaflet focusing	Baseline<br>3 months	1. Time spent in SB;<br>2. Number of breaks in SB

Johansson JF, *et al. BMJ Open* 2022; 12:e053945. doi: 10.1136/bmjopen-2021-053945

Supplementary file 3. Characteristics of 17 included RCTs_27.05.21

UK		(≥27.5 for South Asians).<br>2. Age 18-40, BMI ≥25 (≥23 for South Asians), with ≥1 additional risk factor for diabetes.<br>**Exclusion:**<br>Significant illness, steroid use, diabetes, pregnancy or an inability to communicate in English.	I: 94<br><br>C: 93	C: 33.3 (5.8)<br><br>**Gender:**<br>68.5%<br><br>**Ethnicity:**<br>19.8% black and minority ethnic groups	Parallel groups<br><br>Community	structured education workshop.<br><br>6 weeks	on T2DM, the importance of increasing physical activity and decreasing sedentary behaviour.	12 months	(SB to upright movement) per day (Both by IPAQ and accelerometer);<br>3. Biochemical variables (glucose control, insulin sensitivity, cholesterol levels);<br>4. Anthropometric data (BP, weight, body composition, waist circumference);<br>5. Quality of life (EQ-5D);<br>6. Self-efficacy for SB change;<br>7. Anxiety and depressions (HADS).
**Blunt (2018)**<br><br>Canada	Increase physical activity levels.	**Inclusion:**<br>1. Age 18-85;<br>2. ≥1 self-reported or measured risk factor for chronic disease including: BMI >25, <150 min of exercise/week, ≥3 hours sitting/day, <8 fruit and vegetable servings/day, diagnosis of metabolic syndrome or T2DM.<br>**Exclusion:**<br>Unable to comprehend the letter of information and consent documentation.	118<br><br>I: 59<br>C: 59	**Age:**<br>I: 56.8 (12.3)<br>C: 58.6 (14.7)<br><br>**Gender:**<br>78.8%<br><br>**Ethnicity:**<br>97.5% White	Randomised controlled<br><br>Parallel group<br><br>Primary care health centres	3-phases HealtheSteps™ program – in-person lifestyle coaching, and access to a suite of eHealth technology support.<br><br>18 months	Usual-care wait-list control to begin HealtheSteps™ 6 months after baseline.	Baseline<br>6 months (end of active phase intervention)<br><br>Additional for intervention group in minimally-support phase:<br>12 months<br>18 months	1. Mean daily steps (pedometer; self-report);<br>2. Time spent in PA; sitting (IPAQ);<br>3. Eating habits (STC; modified DINE);<br>4. Quality of life (EQ-5D; EQ-VAS);<br>5. Weight and body composition<br>6. Blood pressure;<br>7. Adverse events.
**Elramli (2017)**<br><br>UK	Increase average daily step count.	**Inclusion:**<br>1. Aged ≥18;<br>2. Confirmed diagnosis of Rheumatoid Arthritis (RA) according to ACR/EULAR 2010 criteria, within 5	76<br><br>I: 39<br>C: 37	**Age:**<br>I: 58.2 (13.5)<br>C: 58.6 (15.8)<br><br>**Gender:**<br>83.9%	Randomised controlled<br><br>Parallel groups	Walk for Rheumatoid Arthritis (WARA) – 6 group sessions in	1 group education session on importance of exercise and healthy diet;	Baseline<br>13 weeks<br>26 weeks<br>52 weeks	1. Daily step count (accelerometer);<br>2. Time spent in SB (accelerometer);<br>3. Time spent in sitting; PA (IPAQ);

Supplementary file 3. Characteristics of 17 included RCTs_27.05.21

		years of diagnosis.<br>**Exclusion:**<br>1. Pregnant, severe hypertension, joint replacement within last 6 months, unstable cardiac conditions, or other serious pathology which would affect ability to take part in physical activity;<br>2. Unable to understand written and spoken English or had cognitive impairment.		 **Ethnicity:**<br>Not reported	Community	first 7 weeks, 2 booster group sessions in week 14 and 28, personal support from physiotherapist on week 7, 9, and 11. Pedometers and PA diaries were given with instructions.<br><br>28 weeks	and written educational material. At end of trial (12-month), provided pedometer and PA diaries, with advice on use.		4. Disease activity (SDAI);<br>5. RA Quality of life (RAQoL);<br>6. Functional capacity (6MWT; MHAQ; hand grip test);<br>7. Cardiovascular risk factors (Blood biochemical variables; ASSIGN score Version 1.5.1; BMI; waist and hip circumferences);<br>8. Dietary assessment (DINE);<br>9. PA self-efficacy.
**Harris (2018)**<br><br>UK	Increase physical activity.	**Inclusion:**<br>1. Aged 45-75;<br>2. Registered at 1 of the 6 participating general practices;<br>3. Able to walk outside the home and with no contraindications to increasing their moderate intensity physical activity levels.<br>**Exclusion:**<br>1. Achieving at least 150 minutes of at least moderate intensity physical activity weekly;<br>2. Living in residential or nursing home, or housebound;<br>3. ≥3 falls, or ≥1 fall required attention, within	1,023<br><br>I:<br>Postal: 339<br>Nurse: 346<br><br>C: 338	**Age:**<br>45-54: 33.2%<br>55-64: 37.8%<br>65-75: 28.9%<br><br>**Gender:**<br>64.1%<br><br>**Ethnicity:**<br>80.3% White<br>10.3% Black<br>6.9% Asian<br>2.5% Other	Randomised controlled<br><br>Parallel groups by household<br><br>Community	1. Postal – pedometer, physical activity diary, and instructions for a 12-week walking programme sent by post.<br><br>2. Nurse support – provided pedometer, physical activity diary, and instructions by a practice nurse, who	Usual physical activity, provided a pedometer and guidance on a 12-week walking programme at end of trial.	Baseline<br>3 months<br>12 months	1. Daily step count (accelerometer);<br>2. Time spent in at least moderate PA (accelerometer);<br>3. Time spent in SB (accelerometer);<br>4. Self-reported PA (GPPAQ; IPAQ);<br>5. Cost-effectiveness to health services;<br>6. Exercise self-efficacy;<br>7. Anxiety, depression;<br>8. Quality of life (EQ-5D);<br>9. BMI; waist circumference; body fat;<br>10. Adverse events;<br>11. Health service use.

Supplementary file 3. Characteristics of 17 included RCTs_27.05.21

		last year;<br>4. Terminal illness, dementia, significant cognitive impairment, blind, new onset chest pain, MI, pregnant, conditions which GP judged for exclusion.				also provided 3 meetings over 3 months to facilitate participants to be more active.			
**Lakerveld (2012)**<br><br>The Netherlands	Improve lifestyle behaviour (dietary, physical activity, and/or smoking).	**Inclusion:**<br>1. Aged 30-50;<br>2. Moderate or high risk of CVD (according to SCORE), or a high risk of T2DM (according to ARIC Study).<br>**Exclusion:**<br>1. Having diabetes;<br>2. Previous CVD;<br>3. Pregnancy;<br>4. Current malignant disease;<br>5. (Severe) mobility problems.	622<br><br>I: 314<br>C: 308	**Age:**<br>I: 43.6 (5.1)<br>C: 43.4 (5.5)<br><br>**Gender:**<br>58%<br><br>**Ethnicity:**<br>Not reported	Randomised controlled<br><br>Parallel groups<br><br>General Practices	Cognitive behavioural programme aimed at modifying dietary, and/or physical activity, and/or smoking behaviour, maximum of six individual counselling sessions of 30 minutes, followed by 3-monthly booster sessions by phone.<br><br>Intervention duration unclear	Provision of health brochures only	Baseline<br>6 months<br>12 months<br>24 months	1. Cardiovascular risk score;<br>2. Diabetes risk score;<br>3. Dietary behaviour (Food Frequency Questionnaire);<br>4. Time spent in PA and SB (SQUASH; a subscale of AQuAA);<br>5. Smoking behaviour;<br>6. Determinants of behavioural change;<br>7. Medical care utilisation;<br>8. BMI, waist-hip circumferences;<br>9. Cost-effectiveness and cost-utility in the societal perspective;<br>10. Quality of life (EQ-5D);<br>11. Blood pressure;<br>12. Blood biochemistry.
**Lane (2010)**<br><br>Ireland	To assess the impact of a community	**Inclusion:**<br>1. A population sample of women participating in a mass 10 km event;	176<br><br>I: 85<br>C: 91	**Age:**<br>21-49: 84%<br><br>**Gender:**	Randomised controlled<br><br>Parallel	2 booklets delivered by post – Booklet 1	Placebo treatment – a healthy eating and nutrition	Baseline<br>6 weeks	1. Time spent in sitting;<br>2. Time spent in sufficient PA levels;<br>3. Time spent in total PA (All

Supplementary file 3. Characteristics of 17 included RCTs_27.05.21

	based, low-contact intervention on the physical activity habits of insufficiently active women.	2.Consented to follow-ups 2 and 6 months afterwards;<br>3. Those who had relapsed to insufficient levels of physical activity were invited.		100%<br><br>**Ethnicity:**<br>Not reported	groups<br><br>Community	targeted the earliest stages of motivational readiness, and step-by-step guide to increase motivation. Booklet 2 targeted already motivated and active stage with information about moderate intensity PA, and staying active.	booklet, delivered by post.		of above by bespoke self-report questionnaire);<br>4. Readiness to change (exercise motivational stage).
**Matson (2018)**<br><br>USA	To decrease sitting; increase standing time and light physical activity.<br><br>(Pilot trial)	**Inclusion:**<br>1. Kaiser Permanente Washington (KPWA) members;<br>2. Age >60;<br>3. BMI 30–50 kg/m²;<br>4. Not residing in long-term care or skilled nursing, no diagnosis of dementia, and no serious mental or a potentially terminal illness.<br>**Exclusion:**<br>1. Unable to stand, were not able to walk one block;<br>2. Participating in another intervention study;	60<br><br>I: 29<br>C: 31	Age:<br>I: 69.0 (4.7)<br>C: 67.8 (5.2)<br><br>Gender:<br>68.3%<br><br>Ethnicity:<br>95.0% Not Hispanic or Latino<br>1.7% Hispanic or Latino<br>3.3% Unknown	Randomised controlled<br><br>Parallel groups<br><br>KPWA primary care clinics	2 health coaching sessions; 4 follow-up health coaching phone calls; and written materials, and email reminders. A wrist-worn device programmed to serve as an outward reminder	Healthy living intervention usually available to the KPWA members<br><br>12 weeks	Baseline<br>12 weeks	1. Time spent in sitting (total time, and number of periods of sitting for ≥30 minutes continuously);<br>2. Daily number of sit-to-stand transitions (breaks from sitting) (Both of above by accelerometer);<br>3. Short Physical Performance Battery;<br>4. Blood pressure;<br>5. Fasting glucose level;<br>6. Total cholesterol level;<br>7. Depressive symptoms (PHQ-8);<br>8.Adverse events.

Supplementary file 3. Characteristics of 17 included RCTs_27.05.21

		3. Reported sitting time of less than 7 hours per day;<br>4. Could not communicate by phone, or speak and read English.				strategy for taking breaks from sitting.<br><br>12 weeks			
**Matthews (2016)**<br><br>UK	Increase walking, reduce sedentary behaviour.	**Inclusion:**<br>1. Aged 18-65;<br>2. Ambulatory and able to walk unaided for 10 minutes at a time, based on self/carer report;<br>3. Any level of intellectual disabilities;<br>4. Not currently taking part in any other research study.<br>**Exclusion:**<br>1. Wheelchair user or significant mobility problems;<br>2. Severe challenging behaviour, or other needs requiring constant one-to-one support from staff;<br>3. Involved in regular physical activity - meeting current public health recommendations for physical activity, for six months or more.	102<br><br>I: 54<br>C: 48	**Age:**<br>I: 44.9 (13.5)<br>C: 47.7 (12.3)<br><br>**Gender:**<br>44.1%<br><br>**Ethnicity:**<br>Not reported	Cluster randomised controlled<br><br>Intellectual disabilities community -based organisations (cluster unit)	Walk Well programme – 3 face-to-face physical activity consultations, written resources for participants and carers, and an individualised, structured walking programme<br><br>12 weeks	12-week waiting list control	Baseline<br>12 weeks<br>24 weeks	1. Daily step count (accelerometer);<br>2. Time spent in SB; MVPA; total PA (accelerometer; IPAQ-S);<br>3. BMI; waist circumference;<br>4. Quality of life (EQ-5D; Subjective Vitality Scale);<br>5. Self-Efficacy for Activity for Persons with Intellectual Disability and Self-Efficacy for Exercise Scale.
**Poston (2013)**<br><br>UK	Behavioural intervention comprising dietary and physical activity	**Inclusion:**<br>1. Pregnant with booking BMI ≥30;<br>2. Singleton pregnancy, gestational age >15^{+0} weeks and <17^{+6} weeks' gestation.<br>**Exclusion:**	183<br><br>I: 94<br>C: 89	**Age:**<br>I: 30.4 (5.7)<br>C: 30.7 (4.9)<br><br>**Gender:**<br>100%<br><br>**Ethnicity:**	Randomised controlled<br><br>Parallel groups<br><br>Antenatal clinics	One-to-one appointment with the health trainer; weekly group sessions for 8 consecutive	Usual antenatal care	Baseline (15^{+0} -18^{+6} weeks' gestation)<br>27^{+0} -28^{+6} weeks' gestation<br>34^{+0} -36^{+0}	1. Attitudinal assessment questionnaire - perceived benefits and barriers and confidence to carry out the dietary and PA behaviours;<br>2. Quality of life (EQ-5D);<br>3. Edinburgh Post Natal Depression Score (EPDS);

Supplementary file 3. Characteristics of 17 included RCTs_27.05.21

	changes to improve glycaemic control in obese pregnant women.<br><br>(Feasibility trial)	1. Gestation <15^{+0} weeks and >17^{+6} weeks;<br>2. Pre-existing diabetes;<br>3. Pre-existing essential hypertension (treated);<br>4. Pre-existing renal disease, multiple pregnancies, systemic lupus erythematosus (SLE), antiphospholipid syndrome, sickle cell disease; thalassemia; celiac disease, currently prescribed metformin; thyroid disease or current psychosis.		56.3% White<br>38.3% Black<br>1.6% Asian<br>3.8% Other		weeks from approximately 19 weeks' gestation; dietary advice, and physical activity level advice; plus usual antenatal care.<br><br>8 weeks		weeks' gestation	4. Dietary assessment;<br>5. Time spent in SB; light PA; MVPA (accelerometer; RPAQ);<br>6. Maternal outcomes: diagnosis of GDM and pre-eclampsia, gestational weight gain, mode of delivery, blood loss at delivery, inpatient nights, detailed clinical and family history, health in current pregnancy, early pregnancy data (ultrasound scan, nuchal screening), blood pressure, routine blood results;<br>7. Neonatal outcomes: birthweight, anthropometry, inpatient nights.
**School of Public Health, HKU (2017)**<br><br>Hong Kong	Healthier lifestyle by adopting Zero Time Exercise (ZTEx), and enhance positive family communication and personal and family wellbeing.	**Inclusion:**<br>1. Aged ≥18 years;<br>2. Parents/grandparents with ≥1 child/grandchild aged 3–17;<br>3. Primary education or higher; and able to read and write Chinese;<br>**Exclusion:**<br>Serious health conditions that might prevent from participating in low intensity physical activity.	728<br><br>I: 386<br>C:342	**Age:**<br>Majority aged 30-49<br>I: 87%<br>C: 84%<br><br>**Gender:**<br>92.1%<br><br>**Ethnicity:**<br>Not reported	Cluster randomised controlled<br><br>Integrated Family Service Centres (cluster unit)	Physical activity intervention – 4 group sessions over 12 months; biweekly/ monthly mobile messages to improve physical activity habit.<br><br>12 months	Healthy eating intervention – similar structural design as intervention group.<br><br>12 months	Baseline<br>3 months<br>6 months<br>12 months	1. Time spent in SB; PA (IPAQ-C);<br>2. Physical fitness performance (hand grip strength; time spent standing on 1 leg; foot pedalling duration);<br>3. Dietary habits;<br>4. Self-reported wellbeing (personal-health; happiness; family harmony).
**Spittaels (2007)**	Increase physical activity.	**Inclusion:**<br>1. Aged 25-55;<br>2. No history of	526<br><br>I:	**Age:**<br>I:<br>Group 1: 39.7	Randomised controlled	Group 1. Online-tailored	Online non-tailored standard	Baseline<br>6 months	1. Time spent in PA; SB (IPAQ).

Supplementary file 3. Characteristics of 17 included RCTs_27.05.21

Belgium		cardiovascular disease;<br>3. Internet access (including email access) either at home or at work.<br><br>**Exclusion:**<br>Not specified.	Group 1: 174<br>Group 2: 175<br><br>C: 177	(8.9)<br>Group 2: 39.3 (8.7)<br><br>C: 40.9 (8.0)<br><br>Gender:<br>30.6%<br><br>**Ethnicity:**<br>Not reported	Parallel groups<br><br>Internet	physical activity advice + 8-week stage-based reinforcement emails.<br><br>Group 2. Online-tailored physical activity advice.<br><br>6 months	physical activity advice – based on information present in the computer-tailored programme.		In addition, in 1 of 6 worksites (n= 57):<br>2. Time spent in MVPA (accelerometer);<br>3. BMI; body fat; blood pressure; heart rate at rest.
**Stathi (2019)**<br><br>UK	Promote active ageing in socially disengaged, inactive older adults.<br><br>(Feasibility trial)	**Inclusion:**<br>1. Sedentary retired adults aged ≥65, reported spending <20 min per week in the past month in MVPA;<br>2. Capable of walking at least 200m.<br><br>**Exclusion:**<br>1. Disease or disability that seriously precluded participation in out-of-house activities, diagnosis of dementia;<br>2. Already meeting current PA recommendations, and regularly engaging with local groups and Activities.	39 Participants:<br><br>I: 22<br>C: 17<br><br>(15 voluntary Activators)	**Age:**<br>I: 72.9 (7.3)<br>C: 75 (6.4)<br><br>**Gender:**<br>43.6%<br><br>**Ethnicity:**<br>97% White	Randomised controlled<br><br>Parallel groups<br><br>Community	ACE (Active, Connected, Engaged) intervention –<br>One-to-one support from a peer volunteer (activator) to attend local activities continuously.<br><br>6 months	Waiting-list control group, and received written materials about local initiatives.	Baseline<br>6 months	1. Number of out of house activities;<br>2. Time spent in SB; lifestyle PA (accelerometer);<br>3. Lower limb function (SPPB);<br>4. Wellbeing (life-satisfaction; subjective wellbeing; resilience; and vitality);<br>5. Self-perceived barriers to activity in the neighbourhood.
**Williams 2019**	Reduce sedentary	Inclusion:<br>1. A diagnosis of any	40	Age:<br>I+C: 43 years	Randomised controlled	WTW intervention	Treatment as usual which	Baseline<br>17 weeks	1. Time spent in SB; light PA; MVPA (accelerometer);

Supplementary file 3. Characteristics of 17 included RCTs_27.05.21

UK	behaviour, increase physical activity.<br><br>(Pilot study)	serious mental illness;<br>2. Meeting any one of the following criteria: i) overweight, ii) at risk of or have diabetes, iii) in the clinician's view, have a sedentary lifestyle, iv) or smoke tobacco;<br>3. Ability to provide informed consent and understands English;<br>4. Aged ≥18 years.	I: 20<br>C: 20	(20–56)<br><br>Gender: 45%<br><br>Ethnicity:<br>50% Black<br>27.5% White<br>12.5% Mixed<br>7.5 Asian<br>2.5 Other	Parallel groups<br><br>3 community mental health teams	including an initial education session, fortnightly coaching, provision of pedometers and access to a weekly walking group.<br><br>17 weeks	consisted of care coordination plus written information on the benefits of increasing activity levels.	6 months	2. Self-report SB and PA (IPAQ);<br>3. Motivation to engage in PA (BREQ-2);<br>4. Blood biochemistry;<br>5. Blood pressure;<br>6. BMI; waist circumference;<br>7. Mental Wellbeing (WEMWBS);<br>8. Functional mobility (TUG test).

Keys:
6MWT = 6-minute Walk Test; ACR/EULAR 2010 criteria = American College of Rheumatology/ European League Against Rheumatism 2010 criteria; ARIC = Atherosclerosis Risk in Communities; AQuAA = Activity Questionnaire for Adolescents & Adults; ASSIGN score = a cardiovascular risk score developed by Dundee University (2006); BCG = Behaviour Change Group; BMI = Body Mass Index; BP = blood pressure; BREQ-2 = Behavioural Regulation in Exercise Questionnaire-2; C = Control group; CVD = Cardiovascular disease; DINE = Dietary Instrument for Nutrition Education; EPDS = Edinburgh Post Natal Depression Score; EQ-5D/6D = European Quality of Life-5 dimensions/6 dimensions; EQ-VAS = European Quality of Life-Visual Analogue Scale; GI = glycaemic index; GP = General practitioner; GPPAQ = General Practice PA Questionnaire; HADS = Hospital Anxiety and Depression Scale; HCP = Health care provider; I = Intervention group; IDDM = insulin-dependent diabetes mellitus; IPAQ = International Physical Activity Questionnaire; IPAQ-C = International Physical Activities Questionnaire-Chinese version; IPAQ-S = International Physical Activity Questionnaire-Short version; KPWA = Kaiser Permanente Washington; MHAQ = Modified Stanford Health Assessment Questionnaire; MI = myocardial infarction; MVPA = Moderate to vigorous physical activity; n = Number of persons; PA = Physical activity; PHQ-8 = Patient Health Questionnaire; RA = Rheumatoid Arthritis; RAQoL = RA Quality of Life; RCT = Randomised Controlled Trial; RPAQ = Recent Physical Activity Questionnaire; SB = Sedentary behaviour; SCORE = Systematic Coronary Risk Evaluation; SD = standard deviation; SDAI = Simple disease activity index; SMART = Specific, Measurable, Achievable, Relevant and Time specific; SPPB = Short Physical Performance Battery; SQUASH = Short Questionnaire to Assess Health Enhancing Physical Activity; STC = Starting the Conversation questionnaire; T2DM = Type 2 Diabetes Mellitus; TEG = Traditional Exercise Group; TUG test = Timed Get Up and Go Test; WEMWBS = Warwick-Edinburgh Mental Wellbeing Scale; WHOQOL = World Health Organization Quality of Life; WTW = Walk this Way
