# Supplementary material for: Systematic review of process evaluations of interventions in trials investigating sedentary behaviour in adults
Source: BMJ Open. 2022 Jan 25;12(1):e053945. doi: 10.1136/bmjopen-2021-053945 (PMC8804646; doi:10.1136/bmjopen-2021-053945)
Supplement: Supplementary data [file bmjopen-2021-053945supp004.pdf]

Supplementary file 4.Characteristics of 17 included process evaluations_27.05.21

**Process Evaluation studies**				
**Study** *(Author (Year)*, **Country**	**Aims** *(whether process evaluation was pre-specified before commencing RCT)*	**Sample size and sampling method**	**Study Design (Data collection methods, e.g., mixed methods)**	**Frameworks for process evaluation**
**Adams (2012)**<br><br>USA	To explore overweight and obese women's perceptions of benefits, challenges and effectiveness of the intervention to reduce SB and increase PA. (Pre-specified)	I: n= 47<br>All participants in the intervention group were asked to complete the questionnaires at the mid-point of the intervention, and intervention end or withdrawing.<br><br>1 researcher<br>The researcher leading the PhD project.	Mixed methods:<br>1. By completing online questionnaires in different weeks during the intervention period, the participants evaluated their perceived benefits and barriers, frequency of using the intervention materials, and the effectiveness and ease of use of the intervention elements; and were asked to provide suggestions for improvement.<br>2. The researcher recorded her observations of the challenges, benefits, and costs in implementing the intervention.<br>3. Attendance and retention data were collected to determine attrition.	Not specified
**Albright (2015)**<br><br>USA	To quantify and compare the barriers to MVPA, frequency of achieving MVPA goals, and the relation of persistent barriers to achievement of goals. (Uncertain whether pre-specified or not)	I: n= 115<br>Study records of all participants in intervention group were used.<br><br>Staff conducted the telephone counselling sessions<br>Sessions were recorded, then selected for evaluation (Selection method and number of staff included were unclear – assuming random selection of the records).	1. Checklist to assess fidelity in 80 of the 1,586 recorded telephone counselling sessions.<br>2. Quantified information from telephone counselling sessions to evaluate goals set and achieved, and barriers.<br>3. Study records for assessing the use of intervention materials and attritions.	Not specified
**Benedetti (2020)**<br><br>Brazil	To conduct a comprehensive programme evaluation including all dimensions of RE-AIM using quantitative and qualitative data. (Uncertain whether pre-specified or not)	Participants in the programme<br>Sample size and sampling method not specified, assuming the BCG group only.<br><br>Staff<br>Professionals delivering the programmes, community health workers, and local and city administrators overseeing public health centers. Sample size and sampling method not specified.	Mixed methods:<br>1. 12 focus groups and 32 interviews with participants in the programme, staff delivering the intervention, or those overseeing the venues at the end of the trial.<br>2. Quantitative data in study records about participation, treatment effects, and fidelity.<br>3. Checklist for assessing implementation.	Framework: RE-AIM Framework (Glasgow et al., 1999)

Johansson JF, *et al. BMJ Open* 2022; 12:e053945. doi: 10.1136/bmjopen-2021-053945

Supplementary file 4.Characteristics of 17 included process evaluations_27.05.21

**Berendsen (2015)**<br><br>The Netherlands	To provide an insight into possible barriers and facilitators in execution and sustainability of lifestyle interventions in primary care. (Pre-specified)	I: n= 247, C: n= 164<br>All participants in intervention and control groups.<br><br>25 Health Care Providers<br>8 physiotherapists, 7 dietitians, 10 lifestyle advisors (who were practice nurses/ dietitian/ physiotherapists) were selected for the interviews (sampling method not specified).	Mixed methods:<br>1. Face-to-face, semi-structured interviews with HCPs at the end of the trial on fidelity, dose, context and strategy for implementation, and sustainability.<br>2. Questionnaires to participants every 3 months about dose and satisfaction.<br>3. HCP registries and logbooks completed during the trial about dose, fidelity, and attrition.	Frameworks: RE-AIM Framework (Glasgow et al., 1999); Steckler & Linnan (2002); Saunders et al. (2005); Grant et al. (2013)
**Biddle (2017)**<br><br>UK	To understand the trial outcome findings from the delivery of the workshop and participant behaviour change strategies. (Pre-specified)	I: n= 71 (then n= 45 at 6 weeks after the workshop; n=10 at 12 months)<br>All participants provided feedback immediately after the workshop, and were contacted at 6 weeks afterwards. Invitations sent to 28 participants at the end of the trial (12 months).<br><br>2 Educator/ Facilitator<br>All the workshop educator and facilitator were interviewed at the end of the trial.	Mixed methods:<br>1. Evaluation sheet completed by participants immediately after the educational workshop.<br>2. Phone interviews 6 weeks after the workshop.<br>3. Phone interviews at the end of the trial on following the intervention, awareness of risk, and suggestions for improvement.<br>4. Face-to-face interview with each workshop educator/ facilitator at the end of the trial on intervention delivery, anticipated effectiveness of the intervention, and suggestions for improvement.	Framework: MRC Guidance (Craig et al., 2008)
**Blunt (2018)**<br><br>Canada	To examine the acceptability of the intervention programme. (Pre-specified)	I: n= 13<br>All participants (n= 39) who attended the follow-up assessment at 12 months were invited to participate in an interview; 13/32 agreed participants purposefully chosen, according to baseline measures, e.g., average step count, and self-rated health.<br><br>12 Coaches<br>All coaches delivered the intervention, except 1 was unavailable due to scheduling conflicts.	1. Semi-structured interviews with coaches upon programme completion at 6 months, exploring experiences, barriers, and facilitators in delivering the intervention, and suggestions for improvement.<br>2. Semi-structured interview with participants at 12 months about the experience making health behaviour changes, programme successes and challenges, and suggestions for improving intervention.	Not specified
**Elramli (2017)**<br><br>UK	To explore participant views regarding the effectiveness of WARA intervention. (Pre-specified)	I: n= 10<br>Participants were chosen from the 3 recruiting hospitals, including both genders, who did and did not change PA level and step counts.	Semi-structured 30-minute phone interview at 6 months to explore participant's views about the effectiveness and overall views of the intervention.	Not specified
**Harris (2018)**	To examine the mechanisms of change by under-standing of	Nurse-supported group I: 295 completed by participants, 251 completed by nurses for participants	Mixed methods:<br>1. Semi-structured phone interviews with participants at the end of the trial, to explore their	Framework: MRC Guidance (Moore et al., 2015)

Supplementary file 4.Characteristics of 17 included process evaluations_27.05.21

UK	how the intervention was delivered and received, and how this may have affected the outcomes.<br>(Pre-specified)	All participants (n= 346) and nurses asked to complete the alliance questionnaires.<br><br><u>Nurse-supported group: n= 21, Postal group: n= 22</u><br>Semi-structured interviews: Participants consented at baseline, completed intervention at 12 months, selected according to step-count change, and baseline characteristics.<br><br><u>7 Nurses</u><br>All 8 nurses were invited to focus group/ interviews; 1 was unavailable and did not participate.	experiences.<br>2. Semi-structured focus groups/ interviews with nurses at the end of the trial to explore experiences of delivering PA consultations.<br>3. Patient alliance questionnaire and nurse alliance questionnaire on quality of delivery and participant responsiveness, covering different intervention aspects (e.g., working together and goal-setting, number of appointments).<br>4. Intervention session audio-records and checklists for fidelity and dose.<br>5. Return of participant's PA diary for participation, fidelity, and dose.<br>6. Trial administrative records about participation, dose, and fidelity.	
**Lakerveld (2012)**<br><br>The Netherlands	To describe the intervention's reach, effectiveness in terms of process outcomes, adoption, and implementation of intervention.<br>(Pre-specified)	<u>I: n= 267</u><br>All participants (n =314) were asked to complete the questionnaire.<br><br><u>8 Practice nurses</u><br>All the nurses delivering the intervention.	1. Trial records for participations, dose, and treatment effects.<br>2. Questionnaires to participants at 6 months to evaluate satisfaction and effects on determinants of lifestyle behavioural change.<br>3. Questionnaires to nurses at 6 months to evaluate the training and their confidence in delivering the intervention.<br>4. 2 counselling sessions conducted by each nurse was tape-recorded to assess the nurse's competence.	Framework: RE-AIM Framework (Dzewaltowski et al., 2004)
**Lane (2010)**<br><br>Ireland	To explore the effectiveness and acceptability of intervention booklets.<br>(Aim is not specified, but assumed according to the reported results; and process evaluation is assumed to be pre-specified)	<u>I: n= 85</u><br>Participants in the intervention group were contacted.	3 weeks and 6 weeks after baseline data were recorded:<br>Questionnaires were mailed or emailed to participants.	Not specified
**Matson (2018)**	Collecting qualitative results to further inform the feasibility	<u>I: n= 22</u><br>The health coaches reported that 23 of all 29 participants were available, interested, or	Semi-structured exit interviews with participants within 10 days of the final follow-up, to explore their experiences and perceived health impact of the	Not specified

Johansson JF, *et al. BMJ Open* 2022; 12:e053945. doi: 10.1136/bmjopen-2021-053945

Supplementary file 4.Characteristics of 17 included process evaluations_27.05.21

USA	and acceptability of the interventions.<br>(Pre-specified)	appropriate for the interview, thus the 23 participants were invited, but 1 participant declined.	intervention.	
**Matthews (2016)**<br><br>UK	To explore the feasibility of a 12-week walking intervention for adults with intellectual disabilities, in relation to context, recruitment and retention, reach, implementation and fidelity.<br>(Pre-specified)	<u>I: n= 20</u><br>Participants who had and did not have successful outcomes.<br><br><u>6 Key stakeholders</u><br>The health professional delivering the intervention; the researcher responsible for intervention delivery and management; 1 participant with positive study outcomes; 1 participant with no significant outcomes; 1 carer; a day centre manager	Mixed methods:<br>All conducted after the end of intervention:<br>1. Semi-structured interviews or focus groups with participants to explore their attitudes towards physical activity and walking, perceived benefits, drawbacks and impact of increased activity, subjective feelings of wellbeing, and any changes in view during the intervention period.<br>2. Interviews with key stakeholders to gain insight from a variety of individuals involved in the study.<br>3. Data input spreadsheet which recorded multiple elements including attendance, reasons for withdrawal from the study, for gaining insight regarding recruitment, retention and reach of the intervention.	Frameworks: MRC Guidance (Moore et al., 2015), WHO (2001); RE-AIM Framework (Glasgow et al., 2012); Steckler & Linnan (2002)
**Poston (2013)**<br><br>UK	To refine the intervention protocol through process evaluation of intervention fidelity.<br>(Pre-specified)	<u>I: n= 9, C: n= 12</u><br>Participants recruited from each study site, using a maximum diversity sampling approach, following an informed consent procedure.<br><br><u>130 audio diaries from Health trainers</u><br>Number of Health trainers completed included, or sampling method not specified.	Mixed methods:<br>All conducted after the end of intervention:<br>1. 17 face-to-face and 4 telephone semi-structured interviews with participants during their pregnancy, to capture their experiences and perceptions of the trial and intervention.<br>2. Audio diaries of health trainers in which they reflected on the fidelity and feasibility of the intervention delivery.<br>3. Study database for evaluating attendance.	Framework: Steckler & Linnan (2002)
**School of Public Health, HKU (2017)**<br><br>Hong Kong	To explore the opinions and experiences of the programme; to evaluate the effectiveness of the programme.<br>(Pre-specified)	<u>I: n= 24, C: n= 8</u><br>Participants who attended all the 4 sessions were invited.<br><br><u>8 Social workers and 1 Clerical staff</u><br>Sampling method not specified.	All conducted at the end of the trial:<br>1. Focus groups with participants to explore their experiences, and the impact of the intervention on their living habits and wellbeing.<br>2. Interviews with staff to collect comments about this study, and suggestions for future improvement.<br>3. Fidelity checks conducted for every session to ensure the quality and implementation of the intervention. Methods and results not reported.	Not specified.
**Spittaels (2007)**	To investigate the effectiveness of	<u>Tailored advice+emails group: n= 128, Tailored advice group: n= 139, C: n= 156</u>	All completed at the end of intervention:<br>1. Questionnaire to all participants to investigate	Not specified

Supplementary file 4.Characteristics of 17 included process evaluations_27.05.21

Belgium	intervention outside laboratory.<br>(Uncertain whether pre-specified or not)	All participants were asked to complete the questionnaire; included participants were those responded.	whether participants remembered the advice, read the advice, and considered the advice had had a positive impact on their physical activity behaviour.<br>2. Further questions to the Tailored advice+emails intervention group to investigate the number of emails received and read, and their opinion on the provision of emails.	
**Stathi (2019)**<br><br>UK	To determine the relative usefulness of different intervention components, to identify ways to refine or improve the intervention.<br>(Pre-specified)	<u>I: n= 20</u><br>Sampling method not specified.<br><br><u>13 Activators</u><br>Sampling method not specified.<br><br><u>2 Coordinators</u><br>Sampling method not specified.	Mixed methods:<br>All conducted at the end of intervention:<br>1. Quantitative process evaluation via a self-administered questionnaire which assessed changes in confidence to get out and about, social support, autonomy, competence, and relatedness.<br>2. 14 semi-structured exit interviews and 7 focus groups conduced with participants, activators and coordinators, to evaluate the effectiveness and suggestions of intervention elements.<br>3. Trial records for evaluating recruitment rate, attendance, completion rate, and acceptability of the intervention.	Framework: MRC Guidance (Moore et al., 2015)
**Williams 2019**<br><br>UK	To establish the feasibility and acceptability of the Walk this Way (WTW) intervention<br>(Pre-specified)	<u>I: n= 5</u><br>Participants who agreed to be interviewed; sampling method unclear.	Mixed methods:<br>1. Semi-structured interviews to evaluate how participants experienced the intervention, and suggestions for improving the intervention.<br>2. Trial records for calculating recruitment rate, attendance, number of participants completed the intervention and refused outcomes measurements.	Not specified.

Keys: ACR/EULAR 2010 criteria = American College of Rheumatology/ European League Against Rheumatism 2010 criteria; ARIC = Atherosclerosis Risk in Communities; BCG = Behaviour Change Group; BMI = Body Mass Index; C = Control group; CVD = Cardiovascular disease; GP = General practitioner; HCP = Health care provider; I = Intervention group; IPAQ = International Physical Activity Questionnaire; MRC: Medical Research Council; MVPA = Moderate to vigorous physical activity; n = number of persons; PA = Physical activity; PhD = Doctor of Philosophy; RCT = Randomised controlled trial; SB = Sedentary behaviour; SCORE = Systematic Coronary Risk Evaluation; T2DM = Type 2 Diabetes Mellitus; sTEG = Traditional Exercise Group; WHO: World Health Organisation

<u>References for process evaluation theoretical frameworks:</u>

Dzewaltowski, D. A., Glasgow, R. E., Klesges, L. M., Estabrooks, P. A., & Brock, E. (2004). RE-AIM: Evidence-based standards and a web resource to improve translation of research into practice. Annals of Behavioral Medicine, 28(2), 75-80.

Supplementary file 4.Characteristics of 17 included process evaluations_27.05.21

Glasgow RE, Vogt TM, Boles SM. Evaluating the public health impact of health promotion interventions: the RE-AIM framework. Am J Public Health. 1999;89(9):1322–7.

Glasgow R, Boles S, Vogt T: Reach Effectiveness Adoption Implementation Maintenance (RE-AIM). [19-Feb-2012]; 2012. www.re-aim.org.

Grant A, Treweek S, Dreischulte T, Foy R, Guthrie B. Process evaluations for cluster-randomised trials of complex interventions: a proposed framework for design and reporting. Trials. 2013;14:15.

Moore GF, Audrey S, Baerker M, Bond L, Bonell C, Hardeman W, et al. Process evaluation of complex interventions: Medical Research Council guidance. BMJ. 2015;350:h1258.

Saunders RP, Evans MH, Joshi P. Developing a process-evaluation plan for assessing health promotion program implementation: a how-to guide. Health Promot Pract. 2005;6(2):134–47.

Steckler A, Linnan L. Process Evaluation for Public Health Interventions and Research. San Francisco: Jossey-Bass; 2002.

World Health Organisation [WHO]. Process Evaluation Workbook. Geneva: WHO; 2001.
