# Supplementary material for: Systematic review of process evaluations of interventions in trials investigating sedentary behaviour in adults
Source: BMJ Open. 2022 Jan 25;12(1):e053945. doi: 10.1136/bmjopen-2021-053945 (PMC8804646; doi:10.1136/bmjopen-2021-053945)
Supplement: Supplementary data [file bmjopen-2021-053945supp005.pdf]

Supplementary file 5. Delivery and mechanisms_27.05.21

Study (Year)	Intended delivery (aim/ intervention description)	Actual delivery (difference from the intended delivery)	Intended mechanism (theoretical model/ logic model)
**Adams (2012)**	**On Our Feet intervention** – combination of 2 face-to-face interactive group sessions, and 6 weekly email messages. 1-2 Weeks were led in-person by the researcher. 3-6 Weeks were conducted over the internet, mainly by email.<br><br>Participants were given feedback on their initial levels of SB and PA, were led through a goal setting activity and provided with self-monitoring tools, e.g., Actigraph activity monitor. Positively-framed email messages that contained peer-modelled alternatives to sitting and additional behavioural feedback were sent weekly.<br><br>**Control group** – waitlist control.	(Adaptations)<br>1. Due to schedule conflict for 1 chapter, the initial presentation and the goal setting activity took place at the same meeting instead of respective weeks. Participants received extra email and phone contact to answer any questions during the second week.<br>2. While the same visual aids were used in the initial presentation in each chapter, the depth of explanation for each chapter varied according to the participants' questions.<br>3. Proposed group activity on emotions regarding sitting and some segments of the presentation were reduced or removed because of the time limit for the sessions.<br>4. Software problems causing inaccurate estimates of SB provided to some participants.	The intervention focused on improving self-efficacy in the Social Cognitive Theory, by addressing 4 self-efficacy construct – mastery experiences, modelling, verbal and social persuasion, and emotional and physiological states. It combined the various stages of changes in the Transtheoretical Model, to reduce SB and increase PA.<br><br>In the group sessions, video and demonstrations modelled the intervention exercises. Participants set goals and rated their confidence in achieving the goal, which was intended to increase recognition of self-efficacy. The self-monitoring tools assisted the re-evaluation of SB. Tailored feedback on behaviour change facilitated mastery experiences. Group discussions, uses of behavioural cues, and positively-framed emails encouraged and prompted continuous behaviour changes.
**Albright (2015)**	**TTCW intervention** – telephone counselling sessions and a website, tailored to address a woman's specific MVPA benefits and barriers over a 12-month intervention.<br>*17 Telephone counselling:*<br>The health educator discussed MVPA goals, anticipated barriers and resolutions with participants; tracked MVPA goals (type of activity, duration, and intensity); and provided tailored suggestions on the TTCW website, by email, or mail.<br>*Schedule of counselling calls:*<br>Phase 1: weekly calls (for month 1); Phase 2: biweekly calls (2 Months and 3 Months); and Phase 3: monthly calls (4 Months to 12	(Adaptations)<br>1. In TTCW group, only 75% of participants set incremental MVPA goals with a health educator during the intervention period.<br><br>2. Some initial PA goals were set at light intensity, because the participants were relatively inactive at the beginning of the intervention.	The tailored TTCW intervention aimed to positively alter the key mediators of PA – personal, social, and environmental factors, to enhance self-efficacy and reduce barriers, using the Social Cognitive theory and Transtheoretical Model theory.<br><br>Health educators provided counselling calls, using Motivational interviewing, to encourage goals settings, problem-solving, self-monitoring, and self-reinforcement, to integrate PA into daily lives; while preparing the participants to prepare and progress through the stages of change.

Supplementary file 5. Delivery and mechanisms_27.05.21

	Months).<br>*TTCW website:*<br>Contained various resources designed to facilitate MVPA, e.g. behaviour-change tip, calendar listing "baby-friendly" exercise sessions in the community, and newsletters. Participants were informed that the website would be updated 2-3 times per month.<br><br>**SWO (control group)** – "standard" PA information was available on the SWO website, e.g., information about how to become more physically active via links to credible sources (i.e., American Heart Association, etc.). Participants in this group did not receive any telephone calls or goal-setting advice about MVPA.		The TTCW website provided information about supportive environments for the participants to exercise; and suggestions about obtaining social support for PA.
**Benedetti (2020)**	Reported as actually delivered interventions.	**BCG –** the behavioural change programme that was adapted from "Active Living Every Day" (ALED), delivered by specifically trained nutrition and exercise science professionals working at the HCs. The sessions included a series of topics related to behaviour change, aiming at a more active lifestyle.<br><br>**TEG -** received a 12-week exercise class conducted at the local HCs, led by exercise professionals employed by the HCs; 3 times per week for 60 minutes. Each session included warm-up, aerobic exercise at 50–80% of maximum aerobic power, resistance training, and cool-down. Participants' heart rate and ratings of perceived effort were tracked during each session.	The BCG was adapted from "Active Living Every Day," or ALED, from the USA (Bors 2009).<br><br>A series of behaviour change topics were delivered through 12 structured weekly meetings, aiming to achieve a more active lifestyle. The topics included finding new opportunities to be active, overcoming challenges, setting goals and rewarding, gaining confidence, enlisting support, avoiding pitfalls, step by step, positive planning, making lasting changes.
**Berendsen (2015)**	(Protocol)<br>**Supervised programme:** 6-7 individual meetings, and 26–34 group meetings with PT.	(Differences)<br>1. In both programmes the number of meetings with all HCPs was lower than planned in the protocol. Participants of the Supervised	Beweegkuur provided a wide-ranging lifestyle counselling by means of Motivational Interviewing and incorporating the concepts from Self-Determination Theory.

Supplementary file 5. Delivery and mechanisms_27.05.21

	**Start-up programme (Control):** 6 individual meetings with PT.<br><br>**Both programmes** comprised 6 individual coaching meetings LSA, 3 individual meetings with a dietitian, and 7 dietary group meetings, for 1 year.<br>The initial individual meetings with the HCPs were to set personal (exercise and nutritional) goals, and identify barriers to a healthy lifestyle through motivational interviewing, which were the basis for meetings. At the end of the programme, each participant met with the LSA to evaluate the lifestyle changes and conclude the intervention.	programme attended, compared to participants of the Start-up programme, more meetings with physiotherapists, but fewer with lifestyle advisors and dietitians.<br>2. No PT group meetings were planned in the protocol for the control Start-up group, but some PTs organised over 9 meetings. Some PT of the start-up programme only planned group meetings, instead of the intended individual meetings with each participant.<br>3. For both groups, 3 individual meetings with the dietitians were planned in the protocol, but the Start-up group received a median of 4 meetings (7 meetings at 75th percentile). On the other hand, some participants did not prefer individual meetings which added fees to participants.<br>4. Some dietitians did not plan individual meetings, and therefore felt there was no opportunity to set individual goals.<br>5. Not all participants reported that they set goals with the PA and dietitian; nor the LSA had explicitly concluded the intervention.<br>6. Not all HCPs were trained in Motivational Interviewing techniques.	All HCPs addressed goals and barriers in the different aspects of lifestyle, to promote participant's motivation for behaviour change, problem-solving skills, and thus promoting participant's sustainable self-efficacy and environment to engage in long-term PA and healthy dietary behaviour.<br><br>It has been hypothesised that the additional amount of guidance within the Supervised programme provided additional contacts and guidance, as a hypothesis that the increase in effects on physical activity would lead to bigger treatment effects.
**Biddle (2017)**	(Protocol)<br>A comprehensive health assessment, including blood tests, was conducted at the trial baseline clinic. Results were sent to all participants (intervention and control groups) and discussed in the educational workshops with each participant.<br><br>**STAND Intervention** – A 3-hour group-based educational workshop, based on the DESMOND and PREPARE structured education protocols, delivered by trained educators; plus a motivational follow-up phone call (6 Weeks) to	Delivered as intended.	STAND intervention started with a letter sent to participants at risk of T2DM and an invitation for risk tests, then discussing with an educator about the risk information and amount of SB time, by using the Commonsense Model of Illness.<br><br>The workshop was based on Commonsense Model and Dual Process Theory, in which the trained educators provided information on risk factors and complications relating to T2DM. Participants were encouraged to assess their own health risk, and to identify their modifiable

Supplementary file 5. Delivery and mechanisms_27.05.21

	review and support participants' behaviour change progress. The 'Gruve' (MUVE, Inc., USA: www.muveinc.com) was provided to participants, for self-monitoring on time spent sedentary and in PA, and prompting for break from prolonged times of inactivity. Text messages were sent to participants to encourage adherence to goals and use of the Gruve.<br><br>**Control group** – received an information leaflet focusing on key illness perceptions of being at risk of T2DM, the importance of increasing physical activity and decreasing sedentary behaviour.		risks.<br><br>Social Cognitive Theory and Behavioural Choice Theory were also employed in the workshop content, to aid participants identifying health risks associated with excess SB, strategies to reduce SB in their daily life, identifying barriers, and setting goals and action plans.<br><br>The self-monitoring tool, the Gruve, was provided to facilitate self-regulation of SB.
**Blunt (2018)**	(Protocol)<br>**The HealtheSteps™ programme** – provided individuals with a specific plan of action to improve their PA levels, healthy eating habits, and reduce sedentary behaviour.<br>*Active phase (0-6 Months):*<br>1. bi-monthly in-person coaching to set prescriptions for physical activity, exercise, and healthy eating; provided by 1 trained HealtheSteps™ coach throughout this phase.<br>2. Access to a Tyze Personal Networks (an online social network to connect with coaches and other participants); phone coaching supports; and a free HealtheSteps™ smartphone app (providing virtual coach, heart rate monitor, step counter, and tracking option to monitor progress).<br>*Maintenance phase I (7-12 Months):* in-person coaching removed, but participants had access to the full suite of eHealth technology supports.<br>*Maintenance phase II (13-18 Months):* access to the full suite of eHealth technology supports removed, and participants only had access to	(Adaptations)<br>The central research team scheduled coaching sessions for some coaches, resulting that some participants had different coaches at each session.	HealtheSteps™ was based on the Social Cognitive theory of self-regulation. The mobile app, online tools and resources, and initial supports from the coaches facilitated positive health behaviour changes and self-management of own risk factors for chronic disease.<br><br>Individualised lifestyle prescriptions were given to participants in the initial phase, using Motivational Interviewing and SMART goal setting principles (specific, measurable, attainable, realistic, and timely for the participant). These aimed to produce positive behaviour change and overcome potential barriers.

Supplementary file 5. Delivery and mechanisms_27.05.21

	publicly available resources and tools.<br><br>**Comparator group (waitlist control)** – This group continued with usual activities without intervention from the study team for the first 6-month period. After the 6 Months follow-up measurements, participants were given the opportunity to start the 6-month HealtheSteps™ programme.		
**Elramli (2017)**	Reported as actually delivered interventions.	**The WARA intervention consisted of 2 components** – *PA component:* a pedometer supported walking programme, aiming to increase participant's average daily step count by 3000 steps above their baseline value, on at least 5 days of the week by 6 months, and to maintain for up to 12 months; and to comply with the UK physical activity guidelines (2011) recommended of a total of 150 minutes per week.<br>*Educational component:* 6 weekly interactive group (up to 6 persons) sessions, each lasted 1 hour; and two booster sessions (at 3 and 6 Months) providing support to participants to evaluate their PA levels and barriers.<br>A WARA booklet was provided to participants, describing the importance of walking, strengthening exercise, reducing SB, and a healthy diet for health benefits.<br><br>**Control group –** 1-hour single education group session (up to 6 persons), included topic regarding the importance of physical activity and healthy diet.	The WARA programme was based on the Social Cognitive Theory, focusing on self-efficacy; and incorporated behaviour change techniques, particularly self-monitoring, feedback, and social support.<br><br>The group education sessions aimed to provide social support; increase the participant's awareness and knowledge of their condition, and encourage PA increase. Therefore, the participant's self-efficacy increase.<br><br>Setting goal of step-count, using pedometer and PA diary, facilitated self-monitoring with feedback from the pedometer, thus increased individual motivation to achieve behaviour change.<br><br>The WARA booklet provided health information which further increased the participant's knowledge and awareness (self-efficacy) of self-management and PA for RA.
**Harris (2018)**	(Protocol)<br>**Pedometer-plus-nurse-support group** – Pedometer and written instructions for a 12-week walking intervention, based on the participant's usual step-count provided. In addition, 3 PA consultations with a practice	(Adaptations)<br>1. Nurses and participants adapted and tailored step count target to individual circumstances, e.g., adjustments were made to the intervention to accommodate religious observances, such as Ramadan and Christmas;	The intervention resources used behaviour change techniques (BCTs).<br><br>3 PA consultations with the practice nurse were divided into 3 stages – First steps, Continuing the changes, and Building lasting habits. They

Supplementary file 5. Delivery and mechanisms_27.05.21

	nurse, individually or as a couple.<br><br>**Pedometer-alone group –** a pedometer, and a 12-week pedometer-based walking programme, posted to the participants. The programme was based on the participant's baseline step-count. On study completion (1 year from baseline), participants in this group were offered a single practice nurse PA consultation.<br><br>**Control group** – No PA intervention. They were offered to choose either receiving a pedometer and the written 12-week pedometer-based walking programme, by post, or as part of a single practice nurse consultation.	during illness; and changes in weather.<br>2. Nurses adapted participant's preferences for interventional materials when tailoring advice, e.g., counting walking by time instead of step-count; whether to use the optional handouts or not.<br>3. Not all participants altered their walking targets; some might have decreased PA level as the target.	included motivational interviewing, health information about PA, suggestions to increase PA, action planning, goal setting, self-monitoring, relapse prevention, which aimed to effect positive changes in participant's step count, PA and SB times; thus longer-term changes in walking habits and health benefits.<br><br>The patient handbook provided the same information as in the nurse consultations.<br><br>Step count diary provided suggestions and instruction for the 12 weeks walking programme. Participants could set goals, self-monitor with feedback from pedometer to increase step count.
**Lakerveld (2012)**	(Protocol)<br>**Intervention group** – Each participant was free to choose the own target lifestyle component(s) (smoking, physical activity or diet). Nurse practitioner provided the CBP to increase participant's motivation and ability to change their dietary pattern, physical activity or smoking behaviour, maximum of 6 individual 30-minute counselling sessions (weekly then reduced to every 2-3 weeks, for 2-4 months); then 3-monthly telephone booster sessions for 12 months. The total intervention period, including booster calls, will be 16 months. The MI and PST counselling methods were used.<br><br>**Control group** – Received written information about their risk of developing T2DM and CVD, and brochures of health guidelines regarding physical activity, healthy diet, and smoking cessation.	(Adaptations)<br>Actual intervention duration is unclear: The number of sessions and schedule described in the results report (Lakerveld et al., 2013) matched the protocol; but the report stated the intervention generally lasted up to 6 months.	The cognitive behavioural programme (CBP) applied the Theory of Planned Behaviour (TPB) and the theory of self-regulation, with 2 counselling techniques - Motivational interviewing (MI), and problem-solving treatment (PST).<br><br>A nurse practitioner used MI to explore the participant's attitude and intention to make lifestyle behaviour change, then resolve the ambivalence between the goal and the actual situation. Afterwards, the nurse practitioner used PST to prompt the participant to find solutions for barriers and reinforcing perceived control for behaviour change. When setting new goals was needed, the same process would be started again.<br><br>The nurse practitioner guided the participant to gradually increase the sense of mastery over difficulties and be more active in planning and

Supplementary file 5. Delivery and mechanisms_27.05.21

			implementing activities.
**Lane (2010)**	Reported as actually delivered interventions.	**Intervention group** – Participants answered a question about the stages of change at baseline. The answer determined either both intervention booklets or just one of them to be posted. The booklets provided information on physical activities and motivation to change, tailored to the participant's readiness to change.<br><br>**Control group** – Received a healthy eating and nutrition booklet, developed by the Irish Heart Foundation, An Bord Bia and the Health Promotion Unit, by post, as placebo treatment.	The tailored intervention applied the trans-theoretical model (TTM), which posits that individuals move through stages of change while learning and adopting new behaviours.<br><br>The intervention consisted of two print booklets, specific to the initial and later stages of motivational readiness. The booklets were adapted for Irish women to promote physical activity, which were broadly based on the TTM model.<br><br>The booklets contained information and structured approaches and strategies, designed to alter self-efficacy, social support, outcome expectancy and barriers to physical activity, tailored to the individual's readiness to change and may subsequently modify physical activity behaviour.
**Matson (2018)**	(Protocol)<br>**STAND intervention** – consisted of 6 health coaching sessions provided by a trained Health Coach, an educational information workbook, SB feedback charts, and a Jawbone UP band.<br>*6 health coaching Sessions:* 2 in-person sessions (first 2 weeks, 45-60 minutes each), providing and explaining the workbook, feedback chart, and Jawbone UP wristband to participants; discussing tailored reminder strategies and setting goals and action plan.<br>After that, *4 bi-weekly phone calls:* (20-40 minutes each) from the Health Coach, to review progress on goals and action plans, problem-solve barriers, use the workbook to guide participants on different types of reminder.<br><br>Based on data from participant's activPAL wear	Delivered as intended.	I-STAND intervention was based on behavioural theories, including social cognitive theory, the ecological model, and habit formation theory.<br><br>Health coaching sessions focused on using different types of reminders, building self-efficacy through motivational interviewing, problem-solving barriers, and setting personalised action plan and graded goals. (Social cognitive theory, habit formation theory)<br>The workbook and coaching sessions included social support, social environment and norms, evaluating participant's environment, to consider the possible changes. (Ecological model).<br>The wrist-worn Jawbone UP band device vibrated every 15 minutes of inactivity. This served as an outward reminder strategy for

Supplementary file 5. Delivery and mechanisms_27.05.21

	at baseline, SB feedback charts 1 Week, and 6 Week were provided to participants.<br><br>**Healthy Living Control group** – 1 in-person health coaching session: Participants were provided a health education workbook containing topics about ageing and instructed to work on 1 topic every 2 weeks using a goal-setting worksheet.<br><br>Every 2 weeks, participants received a check-in letter and asked to complete and return a review progress form.		disrupting the habitual SB, to promote behaviour change and new habits of taking breaks from sitting (habit formation theory).
**Matthews (2016)**	(Protocol)<br>**Walk Well intervention –** 12-week community-based walking programme, consisted of 3 physical activity consultations with a walking advisor; aimed to increase walking by 30-minutes on at least 5 days per week. Participants were provided with education booklets, a pedometer and step diary.<br><br>**Waiting list control group** – were advised to continue with their daily activity for 12-weeks, following which they were invited to participate in the Walk Well intervention.	(Adaptations)<br>1. Some participants experienced difficulty in reading the pedometer and recording step counts in the diary, thus adapted the diary to an alternative "tick box" to indicate having walk(s).<br>2. The physical activity consultations were refined and streamlined to focus on the core components, and flexible options of additional behaviour change techniques for adults with intellectual disability.<br>3. Walking groups were not planned, but expected by some participants, thus arranged by the care centres and carers.	Walk Well was based on the Social Cognitive theory and Trans-theoretical Model.<br><br>The PA consultations method focused on 4 core behaviour change techniques: goal setting; self-monitoring; developing self-efficacy; and mobilising social support. Furthermore, the walking advisor tailored the use of additional behaviour change techniques according to the participant's needs. The aim was autonomy and motivation of the participants to lead a more active lifestyle.<br><br>Input and engagement from carers provided social support from them directly, and their arrangement for group walks among participants.<br><br>The education booklets with visual images and appropriate text provided and reinforce health information.<br><br>Pedometer and step diary complemented the PA consultation, to motivate the participant to set goals and self-monitor step count.

Supplementary file 5. Delivery and mechanisms_27.05.21

**Poston (2013)**	Reported as actually delivered interventions.	Participants were recruited in early 2nd trimester (>15+0 weeks to <17+6 weeks' gestation) to allow adequate time for the intervention programme that was planned to end at each participant's 27+0 and 28+6 weeks' gestation.<br><br>All women attended routine antenatal care appointments and received advice regarding diet and physical activity (PA) in accordance with local policies, which draw on UK NICE guidelines.<br><br>**Intervention group –** participants attended a one-to-one appointment with the HT, provided with a pedometer, a logbook for setting goals and self-monitoring, and a DVD of exercise regime for pregnancy. After that, 8 weekly group sessions from approximately 19 weeks' gestation. The programme included dietary advice choosing low GI food and reducing saturated fats, and increasing daily PA level during pregnancy safely.<br><br>**Control group** – standard care, with additional appointments with the study midwife at 27+0 - 28+6 and 34+0-36+6 weeks', where possible coinciding with routine antenatal visits.	The intervention was based on the Control Theory, and Social Cognitive theory.<br><br>Participants were provided with a pedometer, logbook, an exercise DVD, to set, self-monitor, and achieve SMART (Specific, Measurable, Achievable, Relevant, and Time Specific) goals for diet and PA, using self-regulation techniques from the Control Theory.<br><br>The group sessions facilitated self-identification of benefits and barriers to behaviour change, which facilitated self-efficacy, and provided social support.
**SPH HKU (2017)**	Reported as actually delivered interventions.	**PA group** – received 4 group sessions: 2.5-hour interactive knowledge and motivation enhancement core session at baseline, a 1.5-hour experience sharing booster session at 3 Months, 2.5-hour tea gathering family session at 6 Months, and a Holistic Health session at 1 Year. 16 monthly/bi-weekly health-related text messages to mobile phone for knowledge enhancement and as reminders till one year after baseline.<br><br>**Control group** – received the same intervention framework and methods and the same number and duration of sessions, about Healthy diet.	The PA group intervention was guided by the Health Action Process Approach (HAPA), which proposes motivation, goal setting and planning enhance intention, thus promote its conversion to action. The intervention aimed to enhance knowledge, self-efficacy, and motivation in relation to practising ZTEx<br><br>The conceptual framework proposed that the participants pass the intervention information positively and encourage their family to practise the actions together. Through these family actions and communication, the wellbeing and

Supplementary file 5. Delivery and mechanisms_27.05.21

		Fidelity evaluated but not reported.	harmony of the family were enhanced.<br><br>The strategies included:<br>1. Introducing information on the consequences of physical inactivity, obesity and ZTEx (risk perception);<br>2. Enhancing skills and confidence in the ability to do ZTEx (exercise self-efficacy);<br>3. Associating the health behaviour to the positive outcomes of the trainees (outcome expectations); and<br>4. Introducing cognitive dissonance, i.e., a discrepancy between participants' belief (including a pledge to eat) and behaviour (failure or potential failure to act) to promote intrinsic motivation to change behaviours.<br><br>The mechanism of changes for the Healthy diet intervention (control) was the same, but focusing on healthy diet only.
**Spittaels (2007)**	Reported as actually delivered interventions.	**Tailored information and reinforcement emails group:**<br>*Tailored advice:* Participants completed a questionnaire about their PA and psychosocial determinants on the study's intervention website; subsequently, the tailored advice containing normative PA feedback and suggestions to increase PA levels were produced from it. Participants having intentions to increase PA levels were encouraged to make an action plan.<br><br>*Emails:* After receiving the first tailored advice, participants received regular emails (5 emails in 8 weeks), which asked participants to identify their current stages of change, then referred to a corresponding website with personalised information to encourage behaviour changes.	According to each individual's stage of changes, the tailored advice was provided to participants based in Transtheoretical model. The content applied the constructs of Theory of Planned Behaviour, i.e., intentions, attitudes, self-efficacy, social support, knowledge, benefits and barriers to physical activity.<br><br>Participants indicated with positive intentions to increase their PA levels in the online questionnaire were then encouraged by the website to make a personal action plan to implement behaviour changes.<br><br>Reinforcement emails assessed and followed the participant's stage of change, then directed the participant to pertinent online advice to further encourage behaviour changes.

Supplementary file 5. Delivery and mechanisms_27.05.21

		**Tailored information group:** Participants received the tailored advice online but did not receive reinforcement emails.<br><br>**Standard advice (Control):**<br>Participants received standard physical activity advice from a website, based on information presented to the other 2 groups, but not individually-tailored, e.g., the benefits of PA, current public health recommendations, the difference intensity PAs, and suggestions to be more physically active.	
**Stathi (2019)**	Reported as actually delivered interventions.	Activators attended a 2-day training course, and received an intervention delivery manual. They were trained on the protocol for types and frequency of interactions with the participants; also encouraged to be flexible according to individual needs.<br><br>Each participant was invited to attend a 6-month programme:<br>*Motivation stage (first 2 weeks)* – 2 one-to-one meetings with an activator to support motivation, build rapport, review local activities, and consider and address any barriers to participation.<br>*Action stage (1-3 Months)* – ≥3 visits to local initiatives with the activator.<br>*Maintenance stage (3-6 Months*) – Support provided by telephone, and ≥2 further visits with the activator to encourage the participant to attend local activities independently.<br>Participants could engage in a wide range of activities at the Action and Maintenance stage, e.g., bowling, ballroom dancing, lunch clubs, walking groups, and art classes. 2 social events were organised for all participants and activators to facilitate within group support and encourage more local engagement.	Intended processes of behaviour change during the three stages of the ACE intervention followed the principles of Self Determination Theory, to facilitate the participant's developing autonomous motivation, confidence, and competence for getting out and about.<br><br>In the Motivation stage, the participant engaged in social support from the activator, understood the process, and explored and enhanced motivation for actions. In Action stage, the participant made plans with the activator to try out interested activities and monitored progress. In Maintenance stage, the participant was encouraged to continue with the activities more independently, while the support from the activator was reduced.<br><br>It was shypothesised that participants in the ACE intervention would attend more out-of-house activities, and better motivation to lead an active lifestyle in the long term.

Supplementary file 5. Delivery and mechanisms_27.05.21

		The control group received written materials about local initiatives only, but were offered the intervention at the end of study period.	
**Williams (2019)**	(Protocol)<br>**Walk This Way intervention** – amended from the Walk, Address sensations, Learn about exercise, encourage exercise behaviour for persons with schizophrenia spectrum disorders ('WALC-S') programme<br>*Initial group education session:* 5-10 participants; participants were provided a pedometer for self-monitoring and calendar for recording; setting goals for increasing habitual walking level.<br>*Continuing support and coaching:* every 2 weeks (20-30 minutes), an assigned coach met the participant to review the participant's walking calendar, identify and address barriers and facilitators to increase PA and decrease SB, and provide motivational support to the participant to reach.<br>Weekly walking group: the coaches arranged and invited all participants to an optional weekly group walk (2 hours).<br><br>**Control condition** – Received written information on the benefits of increasing activity levels. This advice was given in accordance with the NHS Foundation Trust policy on physical health.	Delivered as intended.	The Walk this Way intervention employed the COM-B model of behaviour change principles to address capability, opportunity, and motivational barriers to reducing SB and increasing PA.<br><br>The Initial education session aimed to enhance motivation and self-efficacy to make behaviour change.<br><br>Health coaching sessions used the REACH© model of coaching, emphasising individual's accountability involves thinking, feeling, and doing to achieve the self-identified goals. Health information of PA, support and motivation for goal attainment were provided to facilitate the participant to increase walking into daily routine independently.<br><br>The participant's walking goal was set with SMART (Specific, Measurable, Attainable, Realistic and Timely), self-monitored by pedometer and calendar; the step count and factors affecting attainment were discussed with the coach.<br><br>Weekly regular group walk was optional, which provided social support to the participants.

Keys: * = Data from associated publications; ACE = Active, Connected, Engaged intervention; BCG = Behaviour change group; BMI = Body Mass Index; C = Control group; CBP = Cognitive behavioural programme CVD = Cardiovascular disease; DESMAND = Diabetes education and self management for ongoing and newly diagnosed; DM = Diabetes Mellitus; FU = Follow-up; GI = Glycaemic Index; GP = General practitioner; HC = Health centre; HCP = Health care provider; HT = Health trainer; I = Intervention group; LSA = Lifestyle advisor; MVPA = Moderate-to-vigorous physical activity; PA = Physical activity; PREPARE = Prediabetes risk education and physical activity recommendation and encouragement; PT = Physiotherapist; SD = Standard deviation; SMART = Specific, Measurable, Achievable,

BMJ Publishing Group Limited (BMJ) disclaims all liability and responsibility arising from any reliance placed on this supplemental material which has been supplied by the author(s)

Supplementary file 5. Delivery and mechanisms_27.05.21

Relevant, and Time Specific; STAND = Sedentary Time ANd Diabetes; SWO = Standard website-only; TEG = Traditional exercise group; TTCW = Tailored telephone counselling plus website; WARA = Walk for Rheumatoid Arthritis; ZTEx = Zero Time Exercise
