# Supplementary material for: Systematic review of process evaluations of interventions in trials investigating sedentary behaviour in adults
Source: BMJ Open. 2022 Jan 25;12(1):e053945. doi: 10.1136/bmjopen-2021-053945 (PMC8804646; doi:10.1136/bmjopen-2021-053945)
Supplement: Supplementary data [file bmjopen-2021-053945supp006.pdf]

Supplementary file 6. Implementation data_27.05.21

Study (Year)	Fidelity (delivering the intervention as per protocol)	Recruitment (recruiting participants and sites)	Retention (participants remaining in the intervention or control/usual care group)	Reach (dose received and participant engagement)
**Adams (2012)**	Only qualitative data reported.	10 clusters invited.<br>7 clusters recruited (needed active membership n≥12).	I: n= 40 (85.1%)<br>C: n= 24 (85.7%)<br><br>Primary reasons for leaving the study:<br>55% (6/11) Having to wear the activity monitors.<br>18% (2/11) Time commitment too great.<br>18% (2/11) Had not understood length of study.<br>9% (1/11) Went out of town unexpectedly.	23/40 (58%) participants always used 2 of 3 intervention elements<br><br>Overall satisfaction with the programme (Likert scale, 1= not at all, 5= very satisfied):<br>39.5% (17/43) participants rated very satisfied (highest %).<br>97.7% (42/43) participants rated at least "3= somewhat" or above.
**Albright (2015)**	5% (80/1586) recorded telephone counselling sessions evaluated against a checklist of the essential intervention components:<br><br>88% fidelity over the 12-month intervention to the essential intervention components.<br><br>96% calls covered barriers to MVPA discussion.<br>97% calls covered assessing participant's previous MVPA goal.<br>100% calls covered setting the participant's next MVPA goal.<br><br>The two components most	Community recruitment:<br>272 via adverts, e.g., magazines, radio stations;<br>170 randomised,<br><br>Kaiser Permanente recruitment:<br>3844 Postcards sent out;<br>1176 calls made;<br>419 interested in joining;<br>141 randomised.	I: n= 115 (74.7%)<br>C: n= 127 (80.9%)<br><br>Most frequent reasons for failure to complete the intervention:<br>13% Pregnancy.<br>9.5% Too busy.<br>6.1% Discontinued participation, no given reason.<br>3.5% Family/job issues.	TTCW group:<br>90.4% of the participants receiving ≥13 of the 17 scheduled calls.<br><br>78.3% of the participants viewed the website at least once.<br><br>75% of participants set incremental MVPA goals with a health educator during the counselling sessions over the 12-month intervention period.<br><br>Level of achieving set MVPA goals in the 3 phases among all participants:<br><br>High level (≥100% of MVPA goal achieved or exceeded):<br>40.6% of the time during Phase 1 (weekly calls).<br>39.9% of time during Phase 2 (biweekly

Supplementary file 6. Implementation data_27.05.21

	frequently not delivered:<br>Pedometer steps (asked in 68.8% calls).<br>MVPA resources (offered in 80% calls).			calls).<br>42.0% of time during Phase 3 (monthly calls).<br><br>Moderate level (50-99% MVPA goal achieved):<br>23.5% of the time during Phase 1.<br>28.4% of the time during Phase 2.<br>21.1% of the time during Phase 3.<br><br>Low level (0-49% MVPA goal achieved):<br>35.8% of the time during Phase 1.<br>31.7% of the time during Phase 2.<br>36.9% of the time during Phase 3.
**Benedetti (2020)**	Checklist to assess implementation, including programme fidelity, instructor knowledge, classroom, schedule, participants' attention and attendance:<br>All analysed items achieved an average of 98% fidelity.	2 of 5 health districts in Florianopolis were interested in participating, consisting 20 of 50 HCs.<br>6 HCs were interested, and had the physical structure and human resources to offer the programmes, thus were recruited.<br><br>4,071 older adults across the 6 HCs;<br>24.2% (985) individuals were considered eligible;<br>11.5% (114) of eligible participants recruited.	Post-intervention (3 months):<br>BCG: n= 18 (50%)<br>TEG: n= 33 (63.5%)<br>C: n= 23 (88.5%)<br><br>6 months:<br>BCG: n= 17 (47.2%)<br>TEG: n= 32 (61.5%)<br>C: n= 21 (80.8%)<br><br>12 months:<br>BCG: n= 13 (36.1%)<br>TEG: n= 28 (53.8%)<br>C: n= 17 (65.4%)	Overall, 49% of participants attended at least 75% of all sessions, with disengagement occurring mostly in the first three weeks of the study (42%).<br><br>Both intervention groups showed relatively high disengagement rates (BCG 50% vs. TEG 37%) with individuals in the BCG presenting lower rates of overall attendance (27% vs. 47%).
**Berendsen (2015)**	Fidelity:<br>24/25 interviewed HCPs were trained in Motivational Interviewing, and applied MI with the participants.<br><br>100% PTs made an exercise plan with the participants.	30 clusters invited.<br><br>411 participants recruited (with 2 to 30 subjects per cluster, 76.9% of participants referred by the GP).<br><br>Eligibility based on baseline data:<br>- 48.9% met the inclusion criteria.<br>- 10.0% healthy BMI/no comorbidities.	28 clusters remained<br><br>Participants:<br>I: n= 196 (79.4%)<br>C: n= 126 (76.8%)<br><br>From recorded data, the main reasons of drop-outs were health issues (31.5%),	**% = median of attended / planned number of meetings:**<br><br>LSA meetings:<br>I: 50.0%; C:66.7%<br>PT group meetings:<br>I: 47.1% to 61.5%; C: 0% (planned n= 0)<br>PT individual meetings:<br>I: 0% (planned 6 to 7); C: 33.3%

Supplementary file 6. Implementation data_27.05.21

	84.8% of the participants set exercise goals or made an exercise plan with an HCP. 79.9% Exercise plans or goals were made with PT, if participants attended any individual meeting with a PT.<br><br>5/6 dietitians made nutritional plans with the participants. 73.9% of the participants made set nutritional plan or goals with an HCP. 91.7% of the plans or goals were made with the dietitian, if participants attended any individual meeting with a dietitian.<br><br>96.9% participants reported LSA had explained the intervention clearly at the beginning.<br><br>226 participants (from both IG and CG) completed a questionnaire after 12 months:<br>40.7% Reported the LSA had explicitly concluded the intervention.<br>41.2% Reported the intervention was not concluded.<br>18.1% Did not know.<br><br>Dose Delivered:<br>1 PT in start-up programme	- 16.8% higher weight-related risk than the target population.<br>- 24.3% of participants' eligibility could not be checked.	and personal reasons (10.1%).	Dietitian group meetings:<br>I: 42.9%; C: 28.6%<br>Dietitian individual meetings:<br>I: 33.3%; C: 133.3%<br><br>Satisfaction (on scale of 1–10, 10 is best):<br>Mean range (across meeting types):<br>I: 7.1 – 8.0<br>C: 7.1 – 7.3<br>Overall programme (Mean (SD)):<br>I: 7.7 (1.5)<br>C: 7.1 (1.8)

Supplementary file 6. Implementation data_27.05.21

	only planned group meetings with all HCPs, instead of the individual meetings intended per protocol.<br><br>4 dietitians typically offered individual meetings with participants, as per protocol. The other 4 dietitians only planned individual meetings according to participant's preference.			
**Biddle (2017)**	Not reported	Not reported	*I: n= 41 (43.6%)<br>*C: n= 68 (73.1%)<br><br>Reasons for failure to complete the intervention or loss to follow-ups:<br>24.5% (23/94) Did not receive allocated intervention in the intervention group.<br>16% (30/187) No longer want to participate.<br>13.4% (25/187) Failed to attend FU appointment.	23/94 (24%) allocated to intervention group did not attend the structured education workshop.<br>45/94 (47.9%) took part in Week 6 phone progress reviews<br><br>26/31 (84%) participants used the accelerometer daily initially, but this fell to 13/31 participants at 6 weeks.<br><br>25/31 (81%) participants felt the accelerometer as helpful at 6 weeks.<br><br>Workshop feedback:<br>Behaviour change plans for future (6 weeks):<br>4/38 (11%) referred to strategies to sit less<br>17/38 (45%) planned for physical activity<br>Others referred to desired health outcomes<br><br>"Best bits" of the workshop (mentioned most frequently): 1. information on diabetes; 2. the atmosphere of the workshop; 3. Receiving personal data on

Supplementary file 6. Implementation data_27.05.21

				sitting levels and health.<br><br>Behaviour change strategies attempted as reported by participants:<br>18 strategies mentioned to sit less and 8 strategies to move more.
**Blunt (2018)**	Only qualitative data reported	***How recruited participants heard about the study:**<br>51 (45%) from posters or handouts;<br>28 (25%) received an email from the study site advertising the project;<br>15 (13%) from an in-person study recruiter;<br>12 (11%) referred by their health care provider (HCP) and/or HCP team;<br>6 (5%) by word of mouth;<br>1 (1%) other unspecified methods<br>Five did not specify how they heard about the study	*6 months:<br>I: n= 44 (74.6%)<br>C: n= 46 (78.0%)<br><br>3.4% (I: n= 2) Did not attend any session<br>6.8% (I: n= 5, C: n= 3) Personal/health reasons<br>3.4% (I: n= 3, C: n= 1) Time commitment<br>5.9% (I: n= 2, C: n= 5) No longer interested<br><br>*12 months:<br>I: n= 37 (63%)<br><br>*18 months:<br>I: n= 35 (59%)	***Attendance:**<br>5% attended no sessions;<br>17% attended 1 session;<br>10% attended 2 sessions;<br>20% attended 3 sessions;<br>48% attended all 4 sessions.<br>Across all sites, 40 participants (68%) were classified as programme completers.<br>Among participants who completed the intervention programme, 30% attended 3 in-person sessions, 70% attended all 4 sessions.
**Elramli (2017)**	Not reported	320 participants invited:<br>106 (33.1%) did not respond;<br>122 (38.1%) ineligible;<br>92 (28.8%) assessed for eligibility;<br>76 (23.8%) randomised	3 months:<br>I: n= 36 (92.3%)<br>C: n= 26 (70.3%)<br><br>6 months:<br>I: n= 37 (94.9%)<br>C: n= 22 (59.5%)	Intervention attendance:<br>26 (66.7%) participants attended all 8 education sessions (6 sessions and 2 booster sessions)<br>28 (71.8%) attended 6 sessions<br>71.8 % attended the first booster session<br>76.9% attended the second booster session<br><br>Control group attendance:<br>21 (56.8%) participants attended the single group education session
**Harris (2018)**	Nurse session attendance and session content delivered	11,015 people invited to participate;<br>6,399 did not respond;	3 months:<br>Postal: n= 335 (98.8%)	Diary returned:<br>Postal: 268/339 (79%)

Supplementary file 6. Implementation data_27.05.21

	recorded by the nurses after each session.<br><br>Fidelity to content delivered was high in all sessions; the mean number of items delivered in session one was 11 (range 10–11); six (range 5–6) in sessions 2 and 3.<br><br>Duration of sessions reported by nurses and measured from records were not very far from the recommendation (±≤30% difference maximum).	548 were excluded as a result of self-reported PA guideline achievement;<br>1,023/10,467 (10%) were randomised.	Nurse: n= 335 (96.8%)<br>C: n= 335 (99.1%)<br><br>12 months:<br>Postal: n= 319 (94.1%)<br>Nurse: n= 317 (91.6%)<br>C: n= 329 (97.3%)<br><br>4.3% (Postal: n=15/339, Nurse: n=25/346, C: n=4/338) Withdrawn<br>1.4% (Postal: n=5/339, Nurse: n=4/346, C: n=5/338) Not able to be contacted	Nurse: 281/346 (81%)<br><br>Pedometer use (every day or most days) during 12-week intervention:<br>Postal: 238/294 (81%)<br>Nurse: 269/303 (89%)<br><br>Attending nurse sessions:<br>255/346 (74%) attended all three sessions.<br>258/263 (98%) attended session 3, and reported still using the pedometer and diary every day or sometimes.
**Lakerveld (2012)**	Only qualitative data reported	8,193 people of 12 general practices were invited according the age (30-50 years) and absence of DM or CVD.<br><br>2,401 (29.3%) responded positively;<br>1,186 (14.5%) declined;<br>921 (11.2%) of those who accepted invitation met the waist circumference inclusion criterion;<br>772 (9.4%) attended screening at clinic and consented;<br>622 (7.6%) fully eligible and randomised.	End of intervention (6 months):<br>I: n= 267 (85.0%)<br>C: n= 269 (87.3%)<br><br>12 months:<br>I: n= 249 (79.3%)<br>C: n= 253 (82.1%)<br><br>24 months:<br>I: n= 236 (75.2%)<br>C: n= 244 (79.2%)<br><br>Reasons for loss to follow-up:<br>15.1% (I: n=42/308, C: n=52/314) Unable to attend<br>3.7% (I: n=9/308, C: n=14/314) Withdrew consent<br>1.1% (I: n=5/308, C: n=2/314) Became pregnant<br>1.3% (IG n=5/308, C:	*207 (66%) participants received at least 1 face-to-face session, 78% of them were content with the sessions.<br><br>The median number of attended sessions was 2 (out of a max of 6).

Supplementary file 6. Implementation data_27.05.21

			n=3/314) Unable to contact<br>0.2% (I: n=1/308, C: n=0/314) Died<br>1.4% (I: n=2/308, C: n=7/314) Diagnosed type 2 DM	
**Lane (2010)**	Not reported	11,205 women registered for the Women's Mini Marathon completed a survey about their PA habits.<br><br>Consented respondents were followed up 2 months and 6 months afterwards respectively:<br>2,020 of them provided records of PA changes at both follow-ups;<br>414 of them were identified as having relapsed to insufficient levels of PA and invited to participate in the trial;<br>176 consented to participate.	Follow-up response rate (end of trial at 6 Weeks):<br>I: n= 55 (65%)<br>C: n= 57 (63%)	76% of Intervention group participants responded at 3 Weeks:<br>97% received the booklet(s)<br>90% found the booklet(s) useful<br>50% reported increase in PA levels<br>28.5% felt greater levels of motivation which led to PA increase<br>16% felt they had more knowledge on being active which led to PA increase<br>5% attributed the PA increase to training for the Mini Marathon for the following year<br><br>At end of trial (6 Weeks), receipt and use of materials provided:<br>95% of intervention group participants<br>80% of control group participants
**Matson (2018)**	Not reported	Not reported	*I: n= 29 (100%)<br>*C: n= 25 (80.6%)	Only qualitative data reported
**Matthews (2016)**	Only qualitative data reported	Sample was deemed representative of adults with intellectual disabilities: 91% (n = 93) had mild or moderate intellectual disability.	*End of intervention (12 weeks):<br>I: n= 45 (83.3%)<br>C: n= 43 (89.6%)<br><br>*24 weeks:<br>I: n= 42 (77.8%)<br>C: n= 40 (83.3%)<br><br>Reasons for loss to follow-up:<br>32.4% (I: n=20/54, C: n=13/48) Did not want to	*54 participants were assigned to intervention, and received the intervention.<br><br>*71% took part in all 3 planned face-to-face physical activity consultations.<br><br>*26% took part in 2 consultations<br>*3% took part in 1 consultation

Supplementary file 6. Implementation data_27.05.21

			continue<br>1% (I: n=1/54) Ill-health	
**Poston (2013)**	Goals were set at all group sessions, of which 88% were considered SMART by HTs according to their diaries.	According to information from the Health and Social Care Information Centre (2013), approximately 1:5 pregnant women would be eligible for inclusion.<br><br>473/656 (72%) eligible people declined to participate (43.0% of those who declined were in the lowest quintile for Index of deprivation indicating the most severe deprivation);<br>38% participated.	End of intervention:<br>I: n= 79 (84.0%)<br>C: n= 75 (84.3%)	82/94 (88%) attended at least one group session, and 60 (64%) attended 4 or more.<br><br>42 women (45%) received material from all eight sessions, 6 by full attendance (6%) and 36 when partly/wholly covered by subsequent phone contact.<br><br>Mean of 6.1 (SD 2.6) sessions were attended or partly/wholly covered for the intervention group.
**School of Public Health, HKU (2017)**	Fidelity checks were conducted for every session of the programmes, which ensured the quality of the intervention and the implementation of the key elements in the intervention.	8 participating Integrated Family Service Centres to recruit around 600 eligible parents.<br><br>728 (121.3% of target) randomised.	Trial Core session (baseline):<br>I: n= 357 (92.5%)<br>C: n= 316 (92.4%)<br><br>3 months:<br>I: n= 335 (86.8%)<br>C: n= 306 (89.5%)<br><br>6 months:<br>I: n= 328 (85.0%)<br>C: n= 298 (87.1%)<br><br>End of intervention -12 months:<br>I: n= 309 (80.1%)<br>C: n= 284 (83.0%)<br><br>Reasons for absence from sessions included occupied with other activities, took care of family, illness, could not be contacted, and abroad; the exact number of participants dropped out for each of these reasons cannot	Physical activity group: (386 randomised)<br>357 (92.5%) attended core (1st) session<br>355 (92.0%) attended booster session at 3 months<br>313 (81.1%) attended tea gathering at 6 months<br>281 (72.8%) attended Family Holistic Health session at 1 year.<br><br>Healthy diet group: (342 randomised)<br>316 (92.4%) attended core (1st) session<br>306 (89.5%) attended booster session at 3 months<br>292 (85.4%) attended tea gathering at 6 months<br>268 (78.4%) attended Family Holistic Health session at 1 year.<br><br>Participant's feedback at end of Physical activity programme (on a scale of 0-10, 10 is best) (Mean (SD)):<br>9.0 (1.2) Quality of intervention content<br>9.0 (1.2) Level of utility of the intervention<br><br>100% participants would recommend this

Supplementary file 6. Implementation data_27.05.21

			be ascertained.	intervention programme to their friends and families
**Spittaels (2007)**	Not reported	8,000 employees targeted via 6 worksites using email messages, posters and internal newsletters;<br>570 (7.1%) responded positively;<br>562 (7.0%) returned the baseline questionnaire with the informed consent, and then randomised.<br><br>~65% of participants met the minimal recommendations for physical activity at baseline despite explicit recruitment of inactive participants<br><br>31% participants were female, males comprising the majority of employees in the two biggest worksites for recruitment<br><br>Male participants already had high baseline physical activity scores compared to the general male population (72% vs. 57% meeting the recommendations), whereas female participants were more representative of the population (47% vs. 48% meeting the recommendations).	End of intervention:<br>Tailored advice+emails: n= 116 (66.7%)<br>Tailored advice: n= 122 (69.7%)<br>C: n= 141 (79.7%)	Recalled having received the tailored advice (% participants):<br>97% Tailored advice+emails group<br>94% Tailored advice group<br>53% Control group<br><br>Tailored advice+emails group satisfaction (% participants):<br>92% Received at least 3 of the 5 reinforcement emails<br>77% Read them completely<br>87% Satisfied by number of emails<br>86% Satisfied by frequency of emails<br>45% Felt emails were useful<br>33% Reported behavioural changes
**Stathi (2019)**	Not reported	2,000 mailed invitations were delivered in the target areas resulting in 230 responses from potential participants and activators (response rate 11.5%).<br><br>ACE participants: 154 (7.7%) requests for information packs. 65 (3.3%) people returned reply forms. 40 (2.0%) recruited.<br><br>Activators: 76 (3.8%) requests for information packs. 15 (0.8%) recruited after completing the training.	End of intervention:<br>Activator: n= 15 (100.0%)<br><br>Participants:<br>I: n= 19 (86.4%)<br>C: n= 13 (76.5%)<br><br>Reasons for dropping out prior to final measures:<br>7.7% (3/39) Ill-health<br>5.1% (2/39) Carer commitments	All participants who completed the intervention engaged with their activator at least 7 times as planned.<br><br>Of the 3 participants who dropped out: 2 met their activator less than 5 times but were contacted regularly by phone.

Supplementary file 6. Implementation data_27.05.21

			2.6% (1/39) Lack of time<br>2.6% (1/39) Moving to a different city	
**Williams (2019)**	Not reported	215 eligible service users contacted by letter and phone;<br>71 not interested;<br>104 not contactable;<br>40 (18.6%) recruited.	I: n= 16 (80.0%)<br>C: n= 17 (85.0%)	13 (65%) received intervention:<br>5 did not engage with intervention;<br>2 did not engage with intervention after education session.

Keys: * = Data from associated publications; ACE = Active, Connected, Engaged intervention; BCG = Behaviour change group; BMI = Body Mass Index; C = Control group; CVD = Cardiovascular disease; DM = Diabetes Mellitus; FU = Follow-up; GP = General practitioner; HC = Health centre; HCP = Health care provider; HT = Health trainer; I = Intervention group; LSA = Lifestyle advisor; MVPA = Moderate-to-vigorous physical activity; n = number of persons; PA = Physical activity; PT = Physiotherapist; SD = Standard deviation; SMART = Specific, Measurable, Achievable, Relevant, and Time Specific; TEG = Traditional exercise group; vs = versus
