# Supplementary material for: Systematic review of process evaluations of interventions in trials investigating sedentary behaviour in adults
Source: BMJ Open. 2022 Jan 25;12(1):e053945. doi: 10.1136/bmjopen-2021-053945 (PMC8804646; doi:10.1136/bmjopen-2021-053945)
Supplement: Supplementary data [file bmjopen-2021-053945supp007.pdf]

Supplementary file 7_SB results from RCTs associated with included PEs_27.05.21

**Study (Year)** Number of participants randomised	**Unit of outcome**	**Outcome measure(s) for SB**	**Intervention group Baseline**	**Control group Baseline**	**Intervention group End of intervention[a]**	**Control group End of intervention[a]**
**Adams (2012)**<br><br>I: 47 C: 28	1. Mean % of SB time per day (SD)<br>2. Mean sitting hours per week (SD)	1. Accelerometer<br><br>2. IPAQ	(n= 40)<br>SB: 47.42% (10.77)<br><br>Sitting time: 57.99 (29.70)	(n= 24)<br>SB: 50.7% (13.78)<br><br>Sitting time: 45.18 (34.88)	(n= 40)<br>SB: 49.16% (10.23)<br><br>Sitting time: 46.00 (28.91)	(n= 24)<br>SB: 50.39% (14.92)<br><br>Sitting time: 40.33 (40.68)
**Albright (2015)**<br><br>I: 138 C:140	Mean sitting hours per day (SD)	Active Australia Survey	Traveling to/from work: 1.19 (0.71)*<br>While at work: 2.02 (2.18)<br>Watching TV: 2.05 (1.33)<br>Using a computer at home: 1.27 (0.98)<br>Other leisure time (movies, dining out): 1.38 (1.01)<br>While holding/feeding baby: 2.93 (1.78)	Traveling to/from work: 1.41 (0.82)*<br>While at work: 2.52 (2.5)<br>Watching TV: 1.91 (1.36)<br>Using a computer at home: 1.41 (1.18)<br>Other leisure time (movies, dining out): 1.31 (1.05)<br>While holding/feeding baby: 3.20 (2.08)	Not published	Not published
**Benedetti (2020)**<br><br>BCG: 36<br>TEG: 52<br>C: 26	Baseline: Mean SB minutes per week (SD)<br><br>End of trial: Mean SB minutes per day (SE)	Accelerometer	BCG: 498.5 (113.6)<br>TEG: 529.8 (107.3)	522.8 (86.7)	Change between baseline and end of intervention (3 months):<br>BCG: -14.3 (56.3) [c]<br>TEG: -4.1 (62.2) [c]<br>*Change between baseline and end of trial (12 months):*<br>*BCG: -10.9 (59.9)* [c]<br>*TEG: 4.2 (78.6)* [c]	Change between baseline and end of intervention (3 months):<br>-25.6 (77.9) [c]<br>*Change between baseline and end of trial (12 months): -26.7 (68.3)* [c]
**Berendsen (2015)**<br><br>I: 247 C: 164	Not published	Accelerometer	Not published	Not published	Not published	Not published

Supplementary file 7_SB results from RCTs associated with included PEs_27.05.21

**Biddle (2017)**<br><br>1: 94 C:93	Mean SB hours per day (95% CI)	1. Actigraph (worn on waistband)<br>2. ActivPAL (worn on thigh)	Actigraph (n= 76): 10.83 (10.50, 11.17) [c]<br>ActivPal (n= 60): 8.91 (8.59, 9.24) [c]	Actigraph (n= 80): 11.01 (10.76, 11.26) [c]<br>ActivPal (n= 57): 9.02 (8.73, 9.30) [c]	Outcomes not measured at end of intervention (6 Weeks).<br>*Change between baseline and end of trial (12 months)*<br>Actigraph *(n= 38): -0.29 (-0.75, 0.17)* [c]<br>*ActivPal (time change, n=32): 0.64 (0.13, 1.16)* [c]	Outcomes not measured at end of intervention (6 Weeks).<br>*Change between baseline and end of trial (12 months)*<br>*Actigraph n= 49): -0.23 (-0.60, 0.14)* [c]<br>*ActivPal (n=29): 0.58 (0.06, 1.09)* [c]
**Blunt (2018)**<br><br>I: 59 C:59	Mean sitting minutes per day (SD)	IPAQ	360 (315)	360 (240)	Mean difference between groups (only measured at end of active intervention phase – 6 months): -0.08 (-0.16, -0.006)* [c]	
**Elramli (2017)**<br><br>I: 39 C: 37	Mean SB hours per day (SE)	1. ActivPal<br><br>2. IPAQ	ActivPal SB: 18.0 (0.27) [c]<br>IPAQ weekday sitting: 5.3 (0.31)<br>IPAQ weekend sitting: 5.3 (0.36)	ActivPal SB: 18.5 (0.2) [c]<br>IPAQ weekday sitting: 4.7 (0.41)<br>IPAQ weekend sitting: 4.6 (0.38)	ActivPal SB: 17.2 (0.3) [c]<br>IPAQ weekday sitting: 4.2 (0.33)**<br>IPAQ weekend sitting: 3.9 (0.33)	ActivPal SB: 18.7 (0.41) [c]<br>IPAQ weekday sitting: 5.7 (0.53)**<br>IPAQ weekend sitting: 5.1 (0.63)
**Harris (2018)**<br><br>Postal: 339<br>Nurse: 346<br>C: 338	Mean SB minutes per day (SD, or 95% CI)	Accelerometer	Postal: 614 (71)<br>Nurse: 619 (78)	613 (86)	Mean difference between groups at end of intervention (3 months)<br>Postal versus control: -2 (-12, 7) [c]<br>Nurse versus control: -7 (-16, 3) [c]<br>Nurse versus Postal: -4 (-13, 5) [c]<br>Mean difference between groups at *end of trial (12 months)*<br>*Postal versus control: 1 (-8, 10)* [c]<br>*Nurse versus control: -0.2 (-9, 9)* [c]<br>*Nurse versus Postal: -1 (-10, 8)* [c]	
**Lakerveld (2012)**<br><br>I: 314 C: 308	Mean SB minutes per day (SD)	A subscale of AQuAA	253.7 (146.9) [c]	255.4 (124.5) [c]	Outcomes not measured at end of intervention<br>*End of trial (Month 24): 231.5 (122.2)* [c]	Outcomes not measured at end of intervention<br>*End of trial (Month 24): 233.0 (140.7)* [c]

Supplementary file 7_SB results from RCTs associated with included PEs_27.05.21

**Lane (2010)**<br><br>I: 85 C: 91	Mean sitting time minutes per week (SD)	Frequently used validated questions selected for the trial from other population-level PA interventions.	335.9 (194.9)	310.1 (224.7)	371.4 (170.1)	369.5 (152.6)
**Matson (2018)**<br><br>I: 29 C: 31	Mean sitting time minutes over last 7 days (SD)[b]	ActivPAL	Not published	Not published	Change between baseline and end of intervention (n= 29): -70.1 (104) [b]	Change between baseline and end of intervention (n= 25): 6.5 (69) [b]
**Matthews (2016)**<br><br>I: 54 C: 48	Mean% of time per day spent in SB (SD)	Accelerometer	64.2% (10.5)	66.9% (11.3)	(n= 42)<br>66.4% (10.0) [c]	(n= 40)<br>65.9% (12.0) [c]
**Poston (2013)**<br><br>I: 94 C: 89	Mean SB time minutes per day (SD)	1. Accelerometer<br><br>2. RPAQ	Accelerometer (n= 68): 1165 (91) [c]<br>RPAQ (n= 79): 1009 (187) [c]	Accelerometer (n= 72): 1172 (95) [c]<br>RPAQ (n= 80): 1007 (207) [c]	Accelerometer (n= 36): 1197 (77) [c]<br>RPAQ (n= 56): 1020 (226) [c]	Accelerometer (n= 39): 1175 (86) [c]<br>RPAQ (n= 54): 1068 (177) [c]
**School of Public Health HKU (2017)**<br><br>I: 357 C:316	Mean sitting hours in a working day (SD)	IPAQ-C	4.47 (2.47)*	4.11 (2.38)*	4.3	4.2
**Spittaels (2007)**<br><br>I:<br>Group 1 (tailored advice + email): 116<br>Group 2 (tailored advice): 122<br><br>C: 141	Mean sitting minutes per day (SD)	IPAQ	Group 1:<br>Weekday: 482 (183)<br>Weekend day: 308 (160)<br>Group 2:<br>Weekday: 492 (202)<br>Weekend day: 296 (160)	Weekday: 470 (217)<br>Weekend day: 309 (182)	Group 1:<br>Weekday: 443 (168)<br>Weekend day: 276 (131)<br><br>Group 2:<br>Weekday: 438 (172)<br>Weekend day: 268 (141)	Weekday: 419 (181)<br>Weekend day: 271 (139)
**Stathi (2019)**<br><br>I: 22 C: 17	Mean SB minutes per day (SD, +/ 95% CI)	Accelerometer	681.5 (74.9)	616.2 (112.3)	Change between baseline and end of intervention:	Change between baseline and end of intervention:

Supplementary file 7_SB results from RCTs associated with included PEs_27.05.21

					13.1 (77.2) (-26.6, 52.8)	-8.7 (70.7) (-57.6, 75.1)
**Williams (2019)**<br><br>I: 20 C: 20	Mean SB minutes per day (SE)	Accelerometer	577.2 (9.8)	549.2 (19.1)	End of intervention (17 weeks, n= 16): 520.9 (36.2)*<br><br>*End of trial (6 months, n= 8): 508.2 (19.4)**	End of intervention (17 weeks, n= 17): 637.9 (30.4)*<br><br>*End of trial (6 months, n= 13): 661.2 (33.5)**

Supplementary file 3: Sedentary behaviour measured (at baseline and end of the trial) in the randomised controlled trials associated with the included process evaluation studies

Keys: [a] = Results available from the assessment immediately after the intervention, unless otherwise specified; [b] = unclear if adjusted for covariates; [c] = data were adjusted for covariates; * = p value <0.05 for comparison between intervention and control groups (where reported); ** = p value <0.025 for comparison between intervention and control groups reported as accepted statistical significance ; *Italic font* = End of trial results, if available from publications; AQuAA = Activity Questionnaire for Adolescents & Adults; BCG = Behaviour Change Group; C = Control group; FU = Follow-up; I = Intervention group; IPAQ = International Physical Activity Questionnaire; IPAQ-C = International Physical Activities Questionnaire-Chinese version; n = number of persons included in the analysis; RPAQ = Recent Physical Activity Questionnaire; SB = Sedentary behaviour; SD = Standard deviation; SE = Standard error; TEG = Traditional Exercise Group
