# Supplementary material for: Systematic review of process evaluations of interventions in trials investigating sedentary behaviour in adults
Source: BMJ Open. 2022 Jan 25;12(1):e053945. doi: 10.1136/bmjopen-2021-053945 (PMC8804646; doi:10.1136/bmjopen-2021-053945)
Supplement: Supplementary data [file bmjopen-2021-053945supp008.pdf]

Supplementary file 8 characteristics of 24 excluded studies 27.05.21

Study	Reason for exclusion
Ashe 2013	Results of process evaluation not available.
Burton 1995	Ineligible study design: The study did not involve process evaluation.
Cohen 2017	Ineligible study design: The participants were not all assessed at all timepoints throughout the trial. The data from each time point were not obtained from the same sample group throughout the study.
Coll-Planas 2019	Results of process evaluation not available.
Douglas 2019	Ineligible study design: The study is not RCT.
Gray 2018	Sedentary behaviour was not measured in the RCT.
Gummelt 2017	Sedentary behaviour was not measured in the RCT.
Hammerback 2012	Sedentary behaviour was not measured in the RCT.
Harvey 2016	Ineligible study design: The study did not involve process evaluation of exploration of the intervention.
Holt 2019	Sedentary behaviour was not measured in the RCT.
Hsu 2013	Sedentary behaviour was not measured in the RCT.
Jayaprakash 2016	Sedentary behaviour was not measured in the RCT: Sedentary behaviour was measured at baseline, but not throughout the trial as an outcome.
Lai 2019	Ineligible study design: The study was not a RCT.
Maddison 2020	Sedentary behaviour was not measured in the RCT.
McAuley 2013	Ineligible study design: The study did not involve process evaluation.
Orme 2017	Ineligible study design: The evaluation of feasibility did not involve process evaluation or qualitative evaluation.
Rovniak 2014	Sedentary behaviour was not measured in the RCT.
Sazlina 2015	Results of process evaluation not available.
Seguin 2019	Sedentary behaviour was not measured in the RCT.
Sheppard 2016	Sedentary behaviour was not measured in the RCT.
Stevens 2015	Sedentary behaviour was not measured in the RCT.
Thomsen 2016	Ineligible study design: The study did not involve process evaluation or qualitative evaluation.
Thompson 2008	Results of process evaluation not available.
Thornton 2018	Ineligible comparator: The eligible intervention was assigned to the control group, not the experimental intervention group in this study.
Tiedemann 2015	Sedentary behaviour was not measured in the RCT.
van de Glind 2017	Results of process evaluation not available.
van der Wardt 2019	Sedentary behaviour was not measured in the RCT.
Varela-Mato 2016	Ineligible setting: The intervention was delivered at workplace.
Voorn 2016	Sedentary behaviour was not measured in the RCT.
Yeung 2020	Ongoing: Study not completed.
Zabaleta-Del-Olmo 2018	Results of process evaluation not available.

Keys: RCT = Ramdomised Controlled Trial
