# Supplementary material for: Systematic review of process evaluations of interventions in trials investigating sedentary behaviour in adults
Source: BMJ Open. 2022 Jan 25;12(1):e053945. doi: 10.1136/bmjopen-2021-053945 (PMC8804646; doi:10.1136/bmjopen-2021-053945)
Supplement: Supplementary data [file bmjopen-2021-053945supp009.pdf]

Supplementary file 9 _Quality assessment MMAT_27.05.21

	SCREENING QUESTIONS		1. QUALITATIVE STUDIES					2. RANDOMIZED CONTROLLED TRIALS					3. NON-RANDOMIZED STUDIES					4. QUANTITATIVE DESCRIPTIVE STUDIES					5. MIXED METHODS STUDIES				
**Authors (Year)**	**S1**	**S2**	**1.1**	**1.2**	**1.3**	**1.4**	**1.5**	**2.1**	**2.2**	**2.3**	**2.4**	**2.5**	**3.1**	**3.2**	**3.3**	**3.4**	**3.5**	**4.1**	**4.2**	**4.3**	**4.4**	**4.5**	**5.1**	**5.2**	**5.3**	**5.4**	**5.5**
Adams (2012)	Yes	Yes	Yes	Yes	Yes	Yes	Yes	Yes	Yes	Yes	No	Yes						Yes	Yes	Yes	Yes	No	Yes	Yes	Yes	Yes	Yes
Albright (2015)	No	Can't tell						Can't tell	Yes	Can't tell	No	No						Yes	Yes	Yes	Yes	Yes					
Benedetti 2015	Yes	Yes	Yes	Yes	Yes	Yes	Yes	Yes	Yes	Can't tell	Can't tell	No						Yes	Yes	Yes	Can’t tell	Yes	Yes	Yes	Yes	Yes	Yes
Berendsen (2015)	Yes	Yes	Yes	Yes	Yes	Yes	Yes	No	Yes	Can't tell	Yes	Can't tell						Yes	Yes	Yes	Yes	Yes	Yes	Yes	Yes	Yes	Yes
Biddle (2017)	Yes	Yes	Yes	Yes	Yes	Yes	Yes	Yes	Yes	No	No	No						Yes	Yes	Yes	Yes	Yes	Yes	Yes	Yes	Yes	Yes
Blunt (2018)	Yes	Yes	Yes	Yes	Yes	Yes	Yes	Yes	Yes	Yes	No	No															
Elramli (2017)	Yes	Yes	Yes	Yes	Yes	Yes	Yes	No	Yes	No	No	No															
Harris (2018)	Yes	Yes	Yes	Yes	Yes	Yes	Yes	Yes	Yes	Yes	No	Yes						Yes	Yes	Yes	Yes	Yes	Yes	Yes	Yes	Yes	Yes
Lakerveld (2012)	Yes	No						Yes	Yes	Yes	No	Yes						Yes	Yes	Yes	Yes	Yes					
Lane (2010)	Yes	Yes						Can't tell	Yes	Yes	No	Yes						Yes	Yes	Yes	Yes	Yes					
Matson (2018)	Yes	Yes	Yes	Yes	Yes	Yes	Yes	Yes	Yes	Yes	Yes	Can't tell															
Matthews (2016)	Yes	Yes	Yes	Yes	Yes	Yes	Yes	Can't tell	Yes	Yes	No	Yes						Yes	Yes	Yes	Yes	Yes	Yes	Yes	Yes	Yes	Yes
Poston (2013)	Yes	Yes	Yes	Yes	No	No	No	Yes	Yes	Yes	Can't tell	Yes						Yes	Yes	Yes	Yes	Yes	Yes	No	No	Can't tell	No
SPH HKU (2017)	Yes	Yes	Yes	Yes	No	No	No	Can't tell	No	Yes	Can't tell	No															
Spittaels (2007)	Yes	Yes						Can't tell	Yes	Yes	Can't tell	Yes						Yes	Yes	Yes	Yes	Yes					
Stathi (2019)	Yes	Yes	Yes	Yes	No	No	No	Can't tell	No	Yes	Can't tell	Yes						Yes	Yes	Yes	Yes	Yes	Yes	No	No	Can't tell	No
Williams (2019)	Yes	Yes	Yes	Yes	No	No	No	Yes	Yes	No	Yes	No						Yes	Yes	Yes	Yes	Yes	Yes	Yes	Yes	No	No

S1. Are there clear research questions? S2. Do the collected data allow to address the research questions? 1.1. Is the qualitative approach appropriate to answer the research question? 1.2. Are the qualitative data collection methods adequate to address the research question? 1.3. Are the findings adequately derived from the data? 1.4. Is the interpretation of results sufficiently substantiated by data? 1.5. Is there coherence between qualitative data sources, collection, analysis and

Supplementary file 9 _Quality assessment MMAT_27.05.21

interpretation? 2.1. Is randomization appropriately performed? 2.2. Are the groups comparable at baseline? 2.3. Are there complete outcome data? 2.3. Are there complete outcome data? 2.4. Are outcome assessors blinded to the intervention provided? 2.5 Did the participants adhere to the assigned intervention? 3.1 Are the participants representative of the target population? 3.2 Are measurements appropriate regarding both the outcome and intervention? 3.3 Are there complete outcome data? 3.4 Are the confounders accounted for in the design and analysis? 3.5 During the study period, is the intervention administered (or exposure occurred) as intended? 4.1. Is the sampling strategy relevant to address the research question? 4.2. Is the sample representative of the target population? 4.3. Are the measurements appropriate? 4.4. Is the risk of nonresponse bias low? 4.5. Is the statistical analysis appropriate to answer the research question? 5.1. Is there an adequate rationale for using a mixed methods design to address the research question? 5.2. Are the different components of the study effectively integrated to answer the research question? 5.3. Are the outputs of the integration of qualitative and quantitative components adequately interpreted? 5.4. Are divergences and inconsistencies between quantitative and qualitative results adequately addressed? 5.5. Do the different components of the study adhere to the quality criteria of each tradition of the methods involved?
