# Supplementary material for: Systematic review of process evaluations of interventions in trials investigating sedentary behaviour in adults
Source: BMJ Open. 2022 Jan 25;12(1):e053945. doi: 10.1136/bmjopen-2021-053945 (PMC8804646; doi:10.1136/bmjopen-2021-053945)
Supplement: Supplementary data [file bmjopen-2021-053945supp010.pdf]

Supplementary file 10_search strategies 27th October 2021

Our information specialist updated a few of the MeSH terms that weren't available in the previous searches e.g. sedentary behaviour/ screen time/ and sitting position/.

Database: CINAHL (EBSCOost), search modes - Boolean/Phrase:

S1 (MH "Life Style, Sedentary")

S2 TI (sedentary or sitting or sedentariness or sedentarism)

S3 TX ((sedentary or sitting or seated) N5 (behavio* or lifestyle* or life-style* or pattern* or leisure or time or bout*)))

S4 TX((inactiv* or "no exercise" or nonexercise or "non exercise") N3 (adult* or men or women or male or males or female or females or individual* or people or person or population* or senior or seniors or elderly))

S5 TX((sedentary) N3 (adult* or men or women or male or males or female or females or individual* or people or person or population* or senior or seniors or elderly))

S6 TX ((light or low) N1 "physical activ*")

S7 TX "physical* inactiv*"

S8 TX ("leisure time" N5 ("physical* activ*" or passive or inactiv*))

S9 TX "physical activity level*"

S10 (MH "Sitting")

S11 TX ((sitting or lying) N2 posture*)

S12 TX ((uninterrupted or long* or prolong* or extend* or continu* or protracted or sustain* or period* or duration* or time*) N5 (reclin* or sit or sitting or seated or lying))

S13 TX ("sit less" or "sitting less")

S14 TX ((decrease or reduc* or discourag* or lessen*) N3 (sit or sitting or stand or standing or "physical* inactiv*" or sedentar*))

S15 (MH "Screen Time")

S16 TX (time N5 (computer* or television or tv or "video game*" or videogame* or gaming or screen or media))

S17 TX ((watch* or view*) N5 (television or tv))

S18 TX(play* N5 ("video game*" or videogame* or "computer game*"))

S19 TI ((computer* or television or tv or video game* or videogame* or gaming) and (sedentary or "physical* activity" or sitting or seated or underactiv* or "under activ*")))

S20 MH randomized controlled trials

S21 MH single-blind studies

S22 MH double-blind studies

S23 MH random assignment

S24 MH pretest-posttest design

S25 MH cluster sample

S26 TI (randomised OR randomized)

S27 AB (random*)

S28 TI (trial)

S29 MH (sample size)

S30 AB (assigned OR allocated OR control)

S31 MH (placebos)

S32 PT (randomized controlled trial)

S33 AB (control W5 group)

S34 MH (crossover design)

S35 MH (comparative studies)

S36 AB (cluster W3 RCT)

S37 MH animals+

S38 MH (animal studies)

S39 TI (animal model*)

S40 S37 OR S38 OR S39

S41 MH (human)

S42 S40 not S41

S43 S20 OR S21 OR S22 OR S23 OR S24 OR S25 OR S26 OR S27 OR S28 OR S29 OR S30 OR S31 OR S32 OR S33 OR S34 OR S35 OR S36

S44 S43 NOT S42

S45 (MH "Program Evaluation")

S46 (MH "Process Assessment (Health Care)")

S47 TX "program* evaluat*"

S48 TX ((process evaluat*))

S49 S45 OR S46 OR S47 OR S48

S50 S1 OR S2 OR S3 OR S4 OR S5 OR S6 OR S7 OR S8 OR S9 OR S10 OR S11 OR S12 OR S13 OR S14 OR S15 OR S16 OR S17 OR S18 OR S19

S51 S43 AND S49 AND S50

Database: SPORTDiscus (EBSCOHost), search modes - Boolean/Phrase:

S1	SU Sedentary Lifestyle
S2	TI (sedentary or sitting or sedentariness or sedentarism)
S3	TX ((sedentary or sitting or seated) N5 (behavio* or lifestyle* or life-style* or pattern* or leisure or time or bout*))
S4	TX((inactiv* or "no exercise" or nonexercise or "non exercise") N3 (adult* or men or women or male or males or female or females or individual* or people or person or population* or senior or seniors or elderly))
S5	TX((sedentary) N3 (adult* or men or women or male or males or female or females or individual* or people or person or population* or senior or seniors or elderly))
S6	TX ((light or low) N1 "physical activ*")
S7	TX "physical* inactiv*"
S8	TX ("leisure time" N5 ("physical* activ*" or passive or inactiv*))
S9	TX "physical activity level*"
S10	SU sitting
S11	TX ((sitting or lying) N2 posture*)
S12	TX ((uninterrupted or long* or prolong* or extend* or continu* or protracted or sustain* or period* or duration* or time*) N5 (reclin* or sit or sitting or seated or lying))
S13	TX ("sit less" or "sitting less")
S14	TX ((decrease or reduc* or discourag* or lessen*) N3 (sit or sitting or stand or standing or "physical* inactiv*" or sedentar*))
S15	TX (time N5 (computer* or television or tv or "video game*" or videogame* or gaming or screen or media))
S16	TX ((watch* or view*) N5 (television or tv))
S17	TX(play* N5 ("video game*" or videogame* or "computer game*")) OR AB (play* N5 ("video game*" or videogame* or "computer game*")
S18	TI ((computer* or television or tv or "video game*" or videogame* or gaming) and (sedentary or "physical* activity" or sitting or seated or underactiv* or "under activ*"))
S19	((DE "RANDOMIZED controlled trials")))
S20	TX "allocat* random*"
S21	TX "random* assign*"
S22	TI (randomised OR randomized)
S23	TI (trial)
S24	AB (assigned OR allocated OR control)
S25	AB (control W5 group)
S26	TX placebo*
S27	TX clinic* n1 trial*

S28 S19 OR S20 OR S21 OR S22 OR S23 OR S24 OR S25 OR S26 OR S27

S29 SU program evaluation

S30 TX "program* evaluat*"

S31 TX "process evaluat*"

S32 S29 OR S30 OR S31

S33 S1 OR S2 OR S3 OR S4 OR S5 OR S6 OR S7 OR S8 OR S9 OR S10 OR S11 OR S12 OR S13 OR S14 OR S15 OR S16 OR S17 OR S18

S34 S28 AND S32 AND S33

Database: Cochrane Database of Systematic Reviews (Wiley):

#1 MeSH descriptor: [Sedentary Behavior] this term only
#2 ((sedentary or sitting or sedentariness or sedentarism)):ti (Word variations have been searched)
#3 ((sedentary or sitting or seated) near/5 (behavio* or lifestyle* or “life style*” or pattern* or leisure or time or bout*)):ti,ab,kw (Word variations have been searched)
#4 ((inactiv* or "no exercise" or nonexercise or "non exercise") near/3 (adult* or men or women or male or males or female or females or individual* or people or person or population* or senior or seniors or elderly)):ti,ab,kw (Word variations have been searched)
#5 (sedentary near/3 (adult* or men or women or male or males or female or females or individual* or people or person or population* or senior or seniors or elderly)):ti,ab,kw (Word variations have been searched)
#6 (((light or low) near/1 "physical activ*")):ti,ab,kw (Word variations have been searched)
#7 ("physical activity level*"):ti,ab,kw
#8 ("physical* inactiv*"):ti,ab,kw
#9 ("leisure time" near/5 ("physical* activ*" or passive or inactiv*)):ti,ab,kw (Word variations have been searched)
#10 MeSH descriptor: [Sitting Position] explode all trees
#11 ((sitting or lying) near/2 posture*):ti,ab,kw
#12 ((uninterrupted or long* or prolong* or extend* or continu* or protracted or sustain* or period* or duration* or time*) near/5 (reclin* or sit or sitting or seated or lying)):ti,ab,kw
#13 (("sit* less" or "sitting less")):ti,ab,kw
#14 ((light or low) near/1 "physical activ*"):ti,ab,kw
#15 ((decrease or reduc* or discourag* or lessen*) near/3 (sit or sitting or stand or standing or "physical* inactiv*" or sedentar*)):ti,ab,kw
#16 MeSH descriptor: [Screen Time] this term only
#17 (time near/5 (computer* or television or tv or "video game*" or videogame* or gaming or screen or media)):ti,ab,kw
#18 ((watch* or view*) near/5 (television or tv)):ti,ab,kw
#19 (play* near/5 ("video game*" or videogame* or computer game*)):ti,ab,kw
#20 ((computer* or television or tv or "video game*" or videogame* or gaming) and (sedentary or "physical* activity*" or sitting or seated or underactiv* or "under activ*")):ti,ab,kw
#21 {or #1-#20}
#22 (("program* evaluation*")):ti,ab,kw
#23 MeSH descriptor: [Outcome and Process Assessment, Health Care] this term only
#24 MeSH descriptor: [Process Assessment, Health Care] this term only
#25 ("process evaluation*"):ti,ab,kw
#26 {or #22-#25}
#27 #21 and #26
#28 #21 and #26 in Cochrane Reviews

Database: Cochrane Central Register of Controlled Trials (Wiley):

#1 MeSH descriptor: [Sedentary Behavior] this term only
#2 ((sedentary or sitting or sedentariness or sedentarism)):ti (Word variations have been searched)
#3 ((sedentary or sitting or seated) near/5 (behavio* or lifestyle* or “life style*” or pattern* or leisure or time or bout*)):ti,ab,kw (Word variations have been searched)

#4 ((inactiv* or "no exercise" or nonexercise or "non exercise") near/3 (adult* or men or women or male or males or female or females or individual* or people or person or population* or senior or seniors or elderly)):ti,ab,kw (Word variations have been searched)
#5 (sedentary near/3 (adult* or men or women or male or males or female or females or individual* or people or person or population* or senior or seniors or elderly)):ti,ab,kw (Word variations have been searched)
#6 (((light or low) near/1 "physical activ*")):ti,ab,kw (Word variations have been searched)
#7 ("physical activity level*"):ti,ab,kw
#8 ("physical* inactiv*"):ti,ab,kw
#9 ("leisure time" near/5 ("physical* activ*" or passive or inactiv*)):ti,ab,kw (Word variations have been searched)
#10 MeSH descriptor: [Sitting Position] explode all trees
#11 ((sitting or lying) near/2 posture*):ti,ab,kw
#12 ((uninterrupted or long* or prolong* or extend* or continu* or protracted or sustain* or period* or duration* or time*) near/5 (reclin* or sit or sitting or seated or lying)):ti,ab,kw
#13 (("sit* less" or "sitting less")):ti,ab,kw
#14 ((light or low) near/1 "physical activ*"):ti,ab,kw
#15 ((decrease or reduc* or discourag* or lessen*) near/3 (sit or sitting or stand or standing or "physical* inactiv*" or sedentar*)):ti,ab,kw
#16 MeSH descriptor: [Screen Time] this term only
#17 (time near/5 (computer* or television or tv or "video game*" or videogame* or gaming or screen or media)):ti,ab,kw
#18 ((watch* or view*) near/5 (television or tv)):ti,ab,kw
#19 (play* near/5 ("video game*" or videogame* or computer game*)):ti,ab,kw
#20 ((computer* or television or tv or "video game*" or videogame* or gaming) and (sedentary or "physical* activity*" or sitting or seated or underactiv* or "under activ*")):ti,ab,kw
#21 {or #1-#20}
#22 (("program* evaluation*")):ti,ab,kw
#23 MeSH descriptor: [Outcome and Process Assessment, Health Care] this term only
#24 MeSH descriptor: [Process Assessment, Health Care] this term only
#25 ("process evaluation*"):ti,ab,kw
#26 {or #22-#25}
#27 #21 and #26
#28 #21 and #26 in Trials

<u>AMED (Allied and Complementary Medicine) (OVID) <1985 to October 2021>:</u>

1 Sedentary Lifestyle/
2 (sedentary or sitting or sedentariness or sedentarism).ti.
3 (sedentary adj3 (adult? or men or women or male or males or female or females or individual? or people or person or population? or senior or seniors or elderly)).tw.
4 ((inactiv* or no exercise or nonexercise or non exercise) adj3 (adult? or men or women or male or males or female or females or individual? or people or person or population? or senior or seniors or elderly)).tw.
5 (sedentary adj3 (adult? or men or women or males or females or individual? or people or population? or senior or seniors or elderly)).tw.
6 ((light or low) adj physical activ*).tw.
7 physical* inactiv*.tw. (248)
8 (leisure time adj5 (physical* activ* or passive or inactiv*)).tw.
9 "physical activity level*".tw.
10 sitting/
11 ((sitting or lying) adj2 posture*).tw.
12 ((uninterrupted or long* or prolong* or extend* or continu* or protracted or sustain* or period* or duration* or time*) adj5 (reclin* or sit or sitting or seated or lying)).tw.
13 (sit less or sitting less).tw.
14 ((decrease or reduc* or discourag* or lessen*) adj3 (sit or sitting or stand or standing or physical* inactiv* or sedentar*)).tw.
15 (time adj5 (computer* or television or tv or video game? or videogame? or gaming or screen or media)).tw.
16 ((watch* or view*) adj5 (television or tv)).tw.
17 (play* adj5 (video game? or videogame? or computer game?)).tw.
18 ((computer* or television or tv or video game? or videogame? or gaming) and (sedentary or physical* activity* or sitting or seated or underactiv* or under activ*)).ti.
19 or/1-18 [sedentary behaviour]
20 process evaluat*.mp.
21 "Outcome and Process Assessment"/
22 program* evaluat*.mp.

23 or/20-22 [process evaluation]
24 19 and 23 [sedentary behaviour and process evaluation]

Database: Embase Classic+Embase (OVID) <1947 to 2021 October 22>:

1 Sedentary Lifestyle/
2 sedentary time/
3 (sedentary or sitting or sedentariness or sedentarism).ti.
4 ((sedentary or sitting or seated) adj5 (behavio* or lifestyle* or life-style* or pattern* or leisure or time or bout*)).tw,kw.
5 ((inactiv* or no exercise or nonexercise or non exercise) adj3 (adult? or men or women or male or males or female or females or individual? or people or person or population? or senior or seniors or elderly)).tw,kw.
6 (sedentary adj3 (adult? or men or women or male or males or female or females or individuals or people or person or population? or senior or seniors or elderly)).tw,kw.
7 physical* inactiv*.tw,kw.
8 (leisure time adj5 (physical* activ* or passive or inactiv*)).tw,kw.
9 physical activity level*.tw,kw.
10 ((sitting or lying) adj2 posture*).tw,kw.
11 sitting/
12 ((uninterrupted or long* or prolong* or extend* or continu* or protracted or sustain* or period* or duration* or time*) adj2 (reclin* or sit or sitting or seated or lying)).tw,kw.
13 (sit less or sitting less).tw,kw.
14 ((light or low) adj physical activ*).tw,kw.
15 ((decrease or reduc* or discourag* or lessen*) adj3 (sit or sitting or stand or standing or physical* inactiv* or sedentar*)).tw,kw.
16 screen time/
17 (time adj5 (computer* or television or tv or video game? or videogame? or gaming or screen or media)).tw,kw.
18 ((watch* or view*) adj5 (television or tv)).tw.
19 (play* adj5 (video game? or videogame? or computer game?)).tw,kw.
20 ((computer* or television or tv or video game? or videogame? or gaming) and (sedentary or physical* activity* or sitting or seated or underactiv* or under activ*)).ti.
21 or/1-20 [sedentary behaviour]
22 Randomized controlled trial/
23 Controlled clinical study/
24 22 or 23
25 Random*.tw.
26 randomization/
27 intermethod comparison/
28 placebo.tw.
29 (compare or compared or comparison).ti.
30 ((evaluated or evaluate or evaluating or assessed or assess) and (compare or compared or comparing or comparison)).ab.
31 (open adj label).tw.
32 ((double or single or doubly or singly) adj (blind or blinded or blindly)).tw.
33 double blind procedure/
34 parallel group*1.tw.
35 (crossover or cross over).tw.
36 ((assign* or match or matched or allocation) adj5 (alternate or group*1 or intervention*1 or patient*1 or subject*1 or participant*1)).tw.
37 (assigned or allocated).tw.
38 (controlled adj7 (study or design or trial)).tw.
39 (volunteer or volunteers).tw.
40 human experiment/
41 trial.ti.
42 or/25-41
43 42 or 24
44 (random* adj sampl* adj7 ("cross section*" or questionnaire*1 or survey* or database*1)).tw. not (comparative study/ or controlled study/ or randomi?ed controlled.tw. or randomly assigned.tw.)
45 Cross-sectional study/ not (randomized controlled trial/ or controlled clinical study/ or controlled study/ or randomi?ed controlled.tw. or control group*1.tw.)
46 (((case adj control*) and random*) not randomi?ed controlled).tw.
47 (Systematic review not (trial or study)).ti.
48 (nonrandom* not random*).tw.
49 "Random field*".tw.

50 (random cluster adj3 sampl*).tw.
51 (review.ab. and review.pt.) not trial.ti.
52 "we searched".ab. and (review.ti. or review.pt.)
53 "update review".ab.
54 (databases adj4 searched).ab.
55 (rat or rats or mouse or mice or swine or porcine or murine or sheep or lambs or pigs or piglets or rabbit or rabbits or cat or cats or dog or dogs or cattle or bovine or monkey or monkeys or trout or marmoset*1).ti. and animal experiment/
56 Animal experiment/ not (human experiment/ or human/)
57 or/44-56
58 43 not 57 [Cochrane Highly Sensitive Search Strategy for identifying controlled trials in Embase: (2018 revision); Ovid format (Glanville et al 2019b) Validated Search Filter]
59 program evaluat*.mp.
60 health care quality/
61 process evaluat*.mp.
62 or/59-61 [process evaluation]
63 21 and 58 and 62

Database: APA PsycInfo (OVID) <1806 to October Week 3 2021>:

1 Sedentary behavior/
2 (sedentary or sitting or sedentariness or sedentarism).ti.
3 ((sedentary or sitting or seated) adj5 (behavio* or lifestyle* or life-style* or pattern* or leisure or time or bout*)).tw.
4 ((inactiv* or no exercise or nonexercise or non exercise) adj3 (adult? or men or women or male or males or female or females or individual? or people or person or population? or senior or seniors or elderly)).tw.
5 (sedentary adj3 (adult? or men or women or male or males or female or females or individual? or people or person or population? or senior or seniors or elderly)).tw.
6 ((light or low) adj physical activ*).tw.
7 physical* inactiv*.tw.
8 (leisure time adj5 (physical* activ* or passive or inactiv*)).tw.
9 "physical activity level*".tw.
10 ((sitting or lying) adj2 posture*).tw.
11 ((uninterrupted or long* or prolong* or extend* or continu* or protracted or sustain* or period* or duration* or time*) adj5 (reclin* or sit or sitting or seated or lying)).tw.
12 (sit less or sitting less).tw.
13 ((decrease or reduc* or discourag* or lessen*) adj3 (sit or sitting or stand or standing or physical* inactiv* or sedentar*)).tw.
14 screen time/
15 (time adj5 (computer* or television or tv or video game? or videogame? or gaming or screen or media)).tw.
16 ((watch* or view*) adj5 (television or tv)).tw.
17 (play* adj5 (video game? or videogame? or computer game?)).tw.
18 ((computer* or television or tv or video game? or videogame? or gaming) and (sedentary or physical* activity* or sitting or seated or underactiv* or under activ*)).ti.
19 or/1-18 [sednetary behaviour]
20 clinical trials/ or treatment effectiveness evaluation/ or placebo/
21 (random* or RCT or RCTs).tw.
22 (clinical* adj5 trial*).tw.
23 ((control or treatment or experiment* or intervention) adj5 (group* or subject* or patient*)).tw.
24 ((control or experiment* or conservative) adj5 (treatment or therapy or procedure or manage*)).tw.
25 ((singl* or doubl* or tripl* or trebl*) adj5 (blind* or mask*)).tw.
26 (cross over or crossover).tw.
27 (placebo* or sham).tw.
28 rial.ti.
29 (assign* or allocat*).tw.
30 controls.tw.
31 or/20-30 [RCTs]
32 program evaluat*.mp.
33 process evaluat*.mp.
34 evaluation/
35 or/32-34 [process evaluation terms]
36 19 and 31 and 35 [sedentary behaviour and RCTs and process evaluations]

Database: Ovid MEDLINE(R) All <1946 to October 22, 2021>:

1 Sedentary behavior/
2 (sedentary or sitting or sedentariness or sedentarism).ti.
3 ((sedentary or sitting or seated) adj5 (behavio* or lifestyle* or life-style* or pattern* or leisure or time or bout*)).tw,kf.
4 ((inactiv* or no exercise or nonexercise or non exercise) adj3 (adult? or men or women or male or males or female or females or individual? or people or person or population? or senior or seniors or elderly)).tw,kf.
5 (sedentary adj3 (adult? or men or women or male or males or female or females or individual? or people or person or population? or senior or seniors or elderly)).tw,kf.
6 physical* inactiv*.tw,kf.
7 (leisure time adj5 (physical* activ* or passive or inactiv*)).tw,kf.
8 physical activity level*.tw,kf.
9 sitting position/
10 ((sitting or lying) adj2 posture*).tw,kf.
11 ((uninterrupted or long* or prolong* or extend* or continu* or protracted or sustain* or period* or duration* or time*) adj5 (reclin* or sit or sitting or seated or lying)).tw,kf.
12 (sit less or sitting less).tw,kf.
13 ((light or low) adj "physical activ*").tw,kf.
14 ((decrease or reduc* or discourag* or lessen*) adj3 (sit or sitting or stand or standing or physical* inactiv* or sedentar*)).tw,kf.
15 screen time/
16 (time adj5 (computer* or television or tv or video game? or videogame? or gaming or screen or media)).tw,kf.
17 ((watch* or view*) adj5 (television or tv)).tw,kf.
18 (play* adj5 (video game? or videogame? or computer game?)).tw,kf.
19 ((computer* or television or tv or video game? or videogame? or gaming) and (sedentary or physical* activity* or sitting or seated or underactiv* or under activ*)).ti.
20 or/1-19 [sedentary behaviour]
21 program* evaluat*.mp.
22 "Outcome and Process Assessment (Health Care)"/
23 "Process Assessment (Health Care)"/
24 process evaluat*.mp.
25 or/21-24 [process evaluation]
26 randomized controlled trial.pt.
27 controlled clinical trial.pt.
28 randomized.ab.
29 placebo.ab.
30 drug therapy.fs.
31 randomly.ab.
32 trial.ab.
33 groups.ab.
34 26 or 27 or 28 or 29 or 30 or 31 or 32 or 33
35 exp animals/ not humans.sh.
36 34 not 35 [Cochrane RCT filter 2008, sensitivity maximimising]
37 20 and 25 and 36 [sedentary behaviour and process evaluation and RCTs]

Database: Web of Science: Indexes=SCI-EXPANDED, SSCI, CPCI-S, CPCI-SSH, ESCI (Clarivate), Timespan= 1900-2021:

1 TI=((sedentary or sitting or sedentariness or sedentarism))

2 TS=(((sedentary or inactiv* or "no exercise" or nonexercise or "non exercise") near/3 (adult* or men or women or male or males or female or females or individual* or people or person or population* or senior or seniors or elderly)))

3 TS=(((sedentary or sitting or seated) near/5 (behavio* or lifestyle* or "life style*" or pattern* or leisure or time or bout*)))

4 TS=((light or low*) near/1 "physical activ*")

5 TS=("physical* inactiv*")

# 6	TS=("leisure time" near/5 ("physical* activ*" or passive or inactiv*))
# 7	TS=("physical activity level*")
# 8	TS=((sitting or lying) near/2 posture)
# 9	TS=(((uninterrupted or long* or prolong* or extend* or continu* or protracted or sustain* or period* or duration* or time*) near/5 (reclin* or sit or sitting or seated or lying)))
# 10	TS=("sit less" or "sitting less")
# 11	TS=(((decrease or reduc* or discourag* or lessen*) near/3 (sit or sitting or stand or standing or "physical* inactiv*" or sedentar*)))
# 12	TS=((time*) near/3 (computer* or television or tv or "video game*" or videogame* or gaming or screen or media))
# 13	TS=(((watch* or view*) near/5 (television or tv)))
# 14	TS=((play* near/5 ("video game*" or videogame* or "computer gam*")))
# 15	TI=(((computer* or television or tv or "video game*" or videogame* or gaming) and (sedentary or "physical* activit*" or sitting or seated or underactiv* or "under activ*")))
# 16	#15 OR #14 OR #13 OR #12 OR #11 OR #10 OR #9 OR #8 OR #7 OR #6 OR #5 OR #4 OR #3 OR #2 OR #1
# 17	TS=((random* or RCT or placebo or clinical Near/1 trial*))
# 18	TS=(("program* evaluat*"))
# 19	TS=(("process evaluat*"))
# 20	#19 OR #18
# 21	#20 AND #17 AND #16
