# Supplementary material for: Systematic review of process evaluations of interventions in trials investigating sedentary behaviour in adults
Source: BMJ Open. 2022 Jan 25;12(1):e053945. doi: 10.1136/bmjopen-2021-053945 (PMC8804646; doi:10.1136/bmjopen-2021-053945)
Supplement: Author's manuscript [file bmjopen-2021-053945.draft_revisions.pdf]

BMJ Open is committed to open peer review. As part of this commitment we make the peer review history of every article we publish publicly available.

When an article is published we post the peer reviewers' comments and the authors' responses online. We also post the versions of the paper that were used during peer review. These are the versions that the peer review comments apply to.

The versions of the paper that follow are the versions that were submitted during the peer review process. They are not the versions of record or the final published versions. They should not be cited or distributed as the published version of this manuscript.

BMJ Open is an open access journal and the full, final, typeset and author-corrected version of record of the manuscript is available on our site with no access controls, subscription charges or pay-per-view fees (http://bmjopen.bmj.com).

If you have any questions on BMJ Open's open peer review process please email info.bmjopen@bmj.com

BMJ Open

A SYSTEMATIC REVIEW OF PROCESS EVALUATIONS OF INTERVENTIONS IN TRIALS INVESTIGATING SEDENTARY BEHAVIOUR IN ADULTS

Journal:	*BMJ Open*
Manuscript ID	bmjopen-2021-053945
Article Type:	Original research
Date Submitted by the Author:	01-Jun-2021

Complete List of Authors:	Hall, Jessica; Bradford Institute for Health Research, Academic Unit of Ageing and Stroke Research; University of Leeds Leeds Institute of Health Sciences, Academic Unit for Ageing and Stroke Research<br>Lam, Natalie; Bradford Institute for Health Research, Academic Unit for Ageing and Stroke Research; University of Leeds Leeds Institute of Health Sciences, Academic Unit for Ageing and Stroke Research<br>Ozer, Seline; Bradford Institute for Health Research, Academic Unit for Ageing and Stroke Research; University of Leeds Leeds Institute of Health Sciences, Academic Unit for Ageing and Stroke Research<br>Hall, Jennifer; Bradford Institute for Health Research, Academic Unit for Ageing and Stroke Research; University of Bradford, Faculty of Life Sciences and Health Studies<br>Morton, Sarah; The University of Edinburgh Centre for Clinical Brain Sciences, Geriatric Medicine<br>English, Coralie; The University of Newcastle School of Health Sciences, Faculty of Health and Medicine<br>Fitzsimons, Claire F.; University of Edinburgh Physical Activity for Health Research Centre<br>Lawton, Rebecca; University of Leeds, Institute of Psychological Sciences; Bradford Institute for Health Research, Quality and Safety Research<br>Forster, Anne; University of Leeds Leeds Institute of Health Sciences, Academic Unit for Ageing and Stroke Research ; Bradford Institute for Health Research, Academic Unit for Ageing and Stroke Research<br>Clarke, David; University of Leeds Leeds Institute of Health Sciences, Academic Unit for Ageing and Stroke Research ; Bradford Institute for Health Research, Academic Unit for Ageing and Stroke Research

Keywords:	PUBLIC HEALTH, Quality in health care < HEALTH SERVICES ADMINISTRATION & MANAGEMENT, REHABILITATION MEDICINE

SCHOLARONE™
Manuscripts

I, the Submitting Author has the right to grant and does grant on behalf of all authors of the Work (as defined in the below author licence), an exclusive licence and/or a non-exclusive licence for contributions from authors who are: i) UK Crown employees; ii) where BMJ has agreed a CC-BY licence shall apply, and/or iii) in accordance with the terms applicable for US Federal Government officers or employees acting as part of their official duties; on a worldwide, perpetual, irrevocable, royalty-free basis to BMJ Publishing Group Ltd ("BMJ") its licensees and where the relevant Journal is co-owned by BMJ to the co-owners of the Journal, to publish the Work in this journal and any other BMJ products and to exploit all rights, as set out in our licence.

The Submitting Author accepts and understands that any supply made under these terms is made by BMJ to the Submitting Author unless you are acting as an employee on behalf of your employer or a postgraduate student of an affiliated institution which is paying any applicable article publishing charge ("APC") for Open Access articles. Where the Submitting Author wishes to make the Work available on an Open Access basis (and intends to pay the relevant APC), the terms of reuse of such Open Access shall be governed by a Creative Commons licence – details of these licences and which Creative Commons licence will apply to this Work are set out in our licence referred to above.

Other than as permitted in any relevant BMJ Author's Self Archiving Policies, I confirm this Work has not been accepted for publication elsewhere, is not being considered for publication elsewhere and does not duplicate material already published. I confirm all authors consent to publication of this Work and authorise the granting of this licence.

For peer review only

A SYSTEMATIC REVIEW OF PROCESS EVALUATIONS OF INTERVENTIONS IN TRIALS INVESTIGATING SEDENTARY BEHAVIOUR IN ADULTS

Authors:

[1,2] Jessica Faye Hall Jessica.Hall@bthft.nhs.uk (corresponding author); [1,2]Natalie Lam Natalie.Lam@bthft.nhs.uk; [1,2] Seline Ozer Seline.Ozer@bthft.nhs.uk; [1,3] Jennifer Hall Jennifer.Hall@bthft.nhs.uk; [4] Sarah Morton sarah.morton@ed.ac.uk ; [5]Coralie English Coralie.English@newcastle.edu.au; [6] Claire Fitzsimons Claire.Fitzsimons@ed.ac.uk; [7] Rebecca Lawton R.J.Lawton@leeds.ac.uk;[2]Anne Forster A.Forster@Leeds.ac.uk; [2] David Clarke D.J.Clarke@leeds.ac.uk.

[1]Academic Unit for Ageing and Stroke Research, Bradford Institute for Health Research, Temple Bank House, Bradford Royal Infirmary, Bradford, BD9 6RJ, UK

[2] Academic Unit for Ageing and Stroke Research, University of Leeds, Temple Bank House, Bradford Royal Infirmary, Bradford, BD9 6RJ, UK

[3] Faculties of Life Sciences and Health Studies, University of Bradford, Richmond Road, Bradford, BD7 1D7, UK

[4] Geriatric Medicine, Division of Health Sciences, Centre for Clinical Brain Sciences, Room F1424, Royal Infirmary, Edinburgh, EH16 4SB, UK

[5] Physiotherapy Program, School of Health Sciences, Faculty of Health and Medicine, HC-36 Hunter Building, The University of Newcastle, New South Wales 2308, Australia

[6] Physical Activity for Health Research Centre, The University of Edinburgh, St Leonard's Land, Holyrood Road, Edinburgh, EH8 8AQ, UK

[7] Yorkshire Quality and Safety, Bradford Institute for Health Research, Temple Bank House, Bradford Royal Infirmary, Bradford, BD9 6RJ, UK

Word count: 7285

Abstract

Objectives: To systematically review and synthesise the findings from process evaluations of interventions in trials which measured sedentary behaviour as an outcome in adults to explore: 1) how intervention content, implementation, mechanisms of impact and context influence outcomes; 2) how these interventions are experienced from different perspectives (participants, carers, and staff).

Methods: Databases searches were conducted in March 2019 and updated in May 2020 in: CINAHL; SPORTDiscus; Cochrane Database of Systematic Reviews; Cochrane Central Register of Controlled Trials; AMED; EMBASE; PsycINFO; MEDLINE; Web of Science; and ProQuest Dissertations & Theses. Studies meeting the following criteria were included: Process evaluations of trials including interventions where sedentary behaviour was measured as an outcome in adults aged 16 or over from clinical or non-clinical populations. Studies were excluded if interventions were delivered in educational settings, a workplace, or if they were laboratory-based studies focused on the immediate effects of breaking sitting. The Medical Research Council process evaluation framework underpinned the review, informing the objectives, coding framework and providing a structure for synthesising and reporting the findings.

Results: 17 process evaluations were included**.** Five interventions focused on reducing sedentary behaviour or sitting time, 12 aimed to increase physical activity or promote healthier lifestyles. The process evaluations indicated changes in sedentary behaviour outcomes were shaped by numerous factors including: barriers (e.g. staffing difficulties and scheduling problems) and facilitators (e.g. allowing for flexibility) to intervention delivery; contextual factors (e.g. usual lifestyle and religious events); and individual factors (e.g. pain, tiredness, illness, age, and individual preferences).

Discussion: Changing sedentary behaviour is complex. Intervention requires careful consideration of the different factors that could influence changes in outcomes to ensure that interventions can be appropriately tailored to suit different individuals and groups.

For peer review only - http://bmjopen.bmj.com/site/about/guidelines.xhtml

PROSPERO registration number: CRD42018087403

Key words: Sedentary behaviour, systematic review, process evaluation

Strengths and limitations of this study

- This systematic review is guided by Preferred Reporting Items for Systematic Review and Meta-analysis (PRISMA) guidance
- This is the first systematic review which has synthesised data from process evaluations evaluating interventions in trials that measure sedentary behaviour as an outcome in adults
- The Medical Research Council guidance for conducting process evaluations has been used to structure this review and has therefore provided a comprehensive way of identifying factors associated with implementation, mechanisms of impact and context which may influence the effectiveness of randomised controlled trials investigating sedentary behaviour in adults
- Non-English electronic databases were not searched. This limitation may cause language bias.
- There is some inconsistency in the quality in the reporting of the included process evaluations

INTRODUCTION

Sedentary behaviour is defined as any waking behaviour characterised by energy expenditure ≤1.5 Metabolic Equivalents (METS) while in a sitting, lying or reclining posture(1). In recent years, research exploring sedentary behaviour in adults has been expanding rapidly, documenting the potential for sedentary behaviour to have detrimental effects on health, wellbeing, and healthcare costs(2). Randomised controlled trials (RCTs) are particularly useful to examine intervention effectiveness(3). However, this approach cannot fully account for how interventions work, and the degree to which intervention components contribute to effectiveness or ineffectiveness(4).

Interventions targeting sedentary behaviour are typically complex, with multiple interacting components (5). Changes in outcomes following interventions are largely influenced by human behaviours and contextual factors as part of a complex process(6). The value of studying intervention processes, is recognised in the Medical Research Council (MRC) guidelines for developing and evaluating complex interventions(3) and detailed in the guidance for conducting process evaluations of complex interventions(4). Process evaluations are designed to help understand the theoretical assumptions underpinning an intervention, and to disentangle factors which may have contributed to the outcomes of an intervention(4).

The MRC process evaluation framework states that understanding of causal assumptions underpinning interventions and evaluation of how interventions work in practice are vital in building an evidence base that informs policy and practice. The framework outlines key functions of a process evaluation including investigating implementation, mechanisms of impact and context to understand how outcomes are interpreted(4).

To date, systematic reviews have synthesised the evidence of effectiveness of interventions aimed at reducing sedentary behaviour(7, 8). However, it is also important to synthesise findings from process evaluations to understand the complexity of factors that may influence whether interventions are effective in reducing sedentary behaviour as these will inform future interventions in this relatively new research area. This paper seeks to address the following aims and objectives (table 1):

For peer review only - http://bmjopen.bmj.com/site/about/guidelines.xhtml

Aims and objectives

1) To identify process evaluations of interventions in trials which measured sedentary behaviour as an outcome in adults, to understand the intervention content, mechanisms of impact, implementation and delivery approaches and contexts, in which interventions were reported to be effective or ineffective.
2) To explore experiences of participants, family members/carers and intervention staff in interventions that measured sedentary behaviour as an outcome in adults.

Table 1: Review objectives

1. To identify and record the trial data (e.g., design of interventions, sample sizes, duration and content of interventions, and primary and secondary outcome data (from the process evaluation publication or associated publications)).
2. Establish whether logic models or theoretical models were used to explain how interventions were intended to work.
3. Establish whether interventions were delivered as intended (as per protocol).
4. Explore intended or unintended mechanisms that influence the extent to which interventions are effective.
5. Understand barriers and facilitators to delivery of, and participation in, interventions and any recommendations made to address such barriers and facilitators.
6. To synthesise qualitative data concerning the understanding and experiences of interventions from the perspectives of participants, family members/carers and intervention staff.

Qualitative data related to exploring perceptions, views and lived experiences of sedentary behaviour, but not related to receipt or delivery of an intervention were examined in a separate systematic review(9).

The MRC process evaluation framework(4) was the underpinning framework for this review informing the aims and objectives, coding framework, providing a structure for synthesising and reporting findings.

METHODS

Protocol and registration

Reporting of this systematic review is guided by Preferred Reporting Items for Systematic Review and Meta-analysis (PRISMA) guidance,(10) (Supplementary file 1). The review was prospectively registered with PROSPERO (Prospective Register of Systematic Reviews); registration number: CRD42018087403, the protocol has been published (12).

Patient and Public Involvement

No patients involved.

Eligibility criteria

Study design

Studies explicitly identified as a process evaluation, or studies that aimed to understand the functioning of an intervention by examining implementation, mechanisms of impact, and contextual factors(11) . Only process evaluations of RCTs, cluster RCTs, and randomised cross-over trials were included.

For peer review only - http://bmjopen.bmj.com/site/about/guidelines.xhtml

Participants

Adults aged 16 or over regardless of whether they were recruited from a clinical or nonclinical population.

Interventions

Interventions in any study which measured sedentary behaviour as an outcome, even if reducing sedentary behaviour was not the primary outcome.

Interventions were excluded if: they were delivered primarily in schools, colleges, universities, or a workplace; or aimed at the acute (immediate) effects of breaking up sitting time as part of a supervised (usually laboratory-based) intervention.

Comparators

In trials, intervention groups may be compared to: no treatment, usual care, attention control, or waitlist control groups.

Information sources

Electronic sources

In collaboration with information specialist colleagues, comprehensive search strategies were developed using controlled vocabulary and free text terms (Supplementary File 2 for the search strategy for the MEDLINE database). Searches were conducted in March 2019 and updated in May 2020.

We searched the following databases: CINAHL (EBSCOHost); SPORTDiscus (EBSCOHost); Cochrane Database of Systematic Reviews (Wiley); Cochrane Central Register of Controlled Trials (Wiley): AMED (OVID); EMBASE (OVID); PsycINFO (OVID); Ovid MEDLINE(R); OVID MEDLINE(R) and Epub Ahead of Print, In-Process & Other Non-Indexed Citations; Web of Science: Sciences Citation Index Expanded (Clarivate); Web of Science: Social Sciences Citation Index Expanded (Clarivate); Web of Science: Conference Proceedings Citation Index- Science (Clarivate); Web of Science: Conference Proceedings Citation Index- Social Sciences and Humanities (Clarivate); ProQuest Dissertations & Theses.

Searching other sources

In addition to searching electronic databases, we identified process evaluations through examining included studies from a concurrent systematic review exploring the effects of interventions in reducing sedentary behaviour (Hall et al., submitted manuscript, 2021 (12)). We identified process evaluations reported in the studies included in the Hall et al.,(12) review and related process evaluations through reference list scanning, citation searching and contacting authors of the studies included from the literature searches.

Study records

Data management

References identified from electronic databases and other sources were de-duplicated and imported into Endnote X7 reference management software. References were then imported in to Covidence (www.covidence.org, 28th April 2021), a web-based systematic review tool.

Selection process

Using Covidence, two reviewers (RC, NL) independently assessed titles and abstracts of records from the electronic searches against the eligibility criteria and excluded obviously irrelevant studies. The full-text of the remaining studies were obtained; then independently assessed, by the same reviewers, against the eligibility criteria to determine which studies would be eligible for inclusion. The same process for updated literature searches was undertaken (by NL, SO). During the screening process, disagreements were resolved by a consensus-based decision between the reviewers, or if necessary, discussion with a third reviewer (DJC).

For peer review only - http://bmjopen.bmj.com/site/about/guidelines.xhtml

Data extraction and narrative synthesis

A narrative approach to synthesising the data was undertaken to provide detailed written commentary to address the research aims and objectives. Reviewers (RC, NL, and JFH) independently extracted relevant quantitative and qualitative data from the included studies. All quantitative data was checked by a second reviewer (SO). Fifty percent of the qualitative data was compared by NL and JFH.

Developing and refining the framework

To direct data extraction, a framework was produced based on this review's aims, objectives and data to be extracted as specified in the protocol (13). The six themes and relevant subthemes align with the key functions in the MRC process evaluation framework (4) (Table 2). Data extraction items (related to the trial and process evaluations) (13) were coded into the framework then summarised in a series of files focusing on: the characteristics of trials (Supplementary file 3), characteristics of process evaluations (Supplementary file 4), delivery methods and mechanisms of impact (Supplementary file 5), and implementation data including fidelity, recruitment, retention and reach (Supplementary file 6). Within file 6, we have included our definitions of these terms; informed by three key papers (4, 14, 15). Qualitative data from the framework is presented under the subheadings in the 'narrative synthesis findings' section.

To help understand the effects of each included intervention on sedentary behaviour outcomes, the sedentary behaviour measures from the associated RCTs were also extracted (Supplementary file 7). As the review focuses on the findings from the process evaluations, the treatment effects estimated in the RCTs have not been synthesised or analysed.

Two reviewers (JFH, NL) independently coded one study to pilot the framework. Following discussion, minor refinements were made before the final framework was agreed. For example, engagement was added in to barriers and facilitators to participation in the intervention, a clearer definition of context was added for clarity, and a 6th 'miscellaneous' theme was included to code data about trial procedures and qualitative methods, mainly for context where appropriate. The coding rules were also refined, then used in coding the remainder of the included studies.

Table 2: Coding framework

Themes and sub-themes	Definition / descriptions of what should be coded
**1. Implementation data**	
1a. Intended delivery	How the intervention was intended to be delivered (in main paper or protocol)
1b. Actual delivery (including when this has been adapted)	How the intervention was actually delivered , including when it has been adapted from what was intended
1c. Strategies for achieving delivery	How the intervention delivery was achieved (e.g. tailoring interventions to individuals)
1d. Measures of adherence	A measure of adherence that was used in the study (NB: may be some overlap with compliance/fidelity). Definition adopted: *"The extent to which delivered content, frequency, duration and coverage of intervention components/ material are as intended."*
**2. Mechanisms of impact**	
2a. Logic models used to explain how the intervention was intended to work	Coded when a logic model is present

For peer review only - http://bmjopen.bmj.com/site/about/guidelines.xhtml

2b. Theories underpinning the intervention	Theories underpinning the intervention e.g. trans-theoretical model , social cognitive theory and behaviour change techniques (BCTs) from the 93-item taxonomy used as part of the intervention e.g. goal setting, self-monitoring<br>NB: still coded BCTs even if authors do not make reference to a BCT taxonomy
2c. Mediators of change	Factors that explained how the intervention had an effect.
2d. Responses to and interactions with the intervention	Instances where participants or those providing the intervention talked about how they responded to, or interacted with the intervention
2e. Intended mechanisms of action influencing intervention effectiveness	How the intended mechanisms of action influenced effectiveness (e.g. intended mechanism of effect- self monitoring of daily activity)
2f. Unintended mechanisms of action influencing intervention effectiveness	Descriptions of how unintended mechanisms of action influenced effectiveness (e.g. if social support increased intervention effectiveness but the intended mechanism was self-monitoring)
**3. Contextual factors influencing effective and ineffective interventions** (Context includes anything external to the intervention that may act as a barrier or facilitator to its implementation or its effects (4)).	
3a. Influencing implementation	Anything external to the intervention that may have influenced its implementation
3b. Influencing mechanisms	Anything external to the intervention that may have influenced the mechanisms by which the intervention had an effect (or not)
3c. Influencing outcomes	Anything external to the intervention that may have influenced the outcomes of the intervention
**4. Barriers and facilitators**	
4a. Barriers to delivery of intervention	Factors that hindered the delivery of the intervention (including internal factors)
4b. Facilitators to delivery of intervention	Factors that enhanced the delivery of the intervention (including internal factors)
4c. Barriers to participation and/or engagement in intervention	Factors that hindered participation or engagement in the intervention: "The extent to which participants understand, accept and enact specific components of the programme in their daily lives."
4d. Facilitators to participation and/or engagement in intervention (e.g. incentives)	Factors that enhanced the delivery of the intervention. Definition as above.
4e. Recommendations made to address barriers and facilitators.	Recommendations made to overcome the barriers and facilitators (from either the study participants (including those delivering)) or the authors of the paper.
**5. Understanding and experiences of interventions from different perspectives**	
5a. Participants' experiences	Experiences from the perspectives of participants that cannot otherwise be coded into context, or barriers and facilitators (likely to be direct quotations)

For peer review only - http://bmjopen.bmj.com/site/about/guidelines.xhtml

5b. Family and carers' experiences	Experiences from the perspectives of family and carers that cannot otherwise be coded into context, or barriers and facilitators. Carers defined as unpaid and informal carers so includes friends and relatives but not paid carers.
5c. Staffs' experiences	Experiences from the perspectives of staff that cannot otherwise be coded into context, or barriers and facilitators. Paid carers that are involved in the intervention would be included here.
5d. Control group experiences	Experiences from control group participants if reported
**6. Miscellaneous**	
**6a. Trial procedures data**	Instances where study includes information that might be of use but is more focused on the data collection e.g. recruitment and retention, rather than the intervention. Agreed not to code any quantitative data that is otherwise captured elsewhere in the review.
**6b. Qualitative methods (to provide context)**	Reports of how qualitative data collection was undertaken e.g. 'semi-structured interviews were conducted with 10 staff.'

Coding into the framework

Using the framework, JFH independently coded all included studies. Nine studies (every other study listed alphabetically) were coded independently by NL. Coding was managed using NVivo software version 12 Plus(16).

Comparing codes

JFH and NL compared data from the nine studies coded by both researchers. To enhance the rigour of the process, JFH then re-reviewed all studies coded singly to ensure consistency(17).

Methodological quality

Methodological quality of included studies was assessed using the Mixed Methods Appraisal Tool (MMAT)(18), which is designed to concurrently assess qualitative, quantitative, and mixed methods studies. Three reviewers (NL, RC, JFH) independently assessed the quality of studies and resolved any discrepancies by making a consensus-based decision, or if necessary, by discussion with a fourth reviewer (DJC). Studies were not excluded from the synthesis based on the outcome of the quality assessment.

For peer review only - http://bmjopen.bmj.com/site/about/guidelines.xhtml

RESULTS

The PRISMA flow diagram (Figure 1), presents results from all searches. Database searches identified 3,167 records; 116 additional records were identified through other sources. After removing duplicates (n = 1,113), 2,170 titles and abstracts were screened; 2,088 records were excluded as they did not meet the pre-defined eligibility criteria. The full-text reports of the remaining 82 records were assessed for eligibility, of which 24 reports were assessed as ineligible. The results of the process evaluations of six eligible studies (seven reports) were unavailable. In total, 17 process evaluation reports were included for data synthesis. Fifty associated reports were also retained for the purpose of this review (e.g. protocols, trial results) to address objective one.

For peer review only

Figure 1: PRISMA flow diagram

For peer review only - http://bmjopen.bmj.com/site/about/guidelines.xhtml

Record of excluded studies

Supplementary file 8 provides reasons for excluding the 24 studies outlined in Figure 1.

Summary of included studies

Included randomised controlled trials

To address objective 1, and provide context for the process evaluations, supplementary file 3 presents data from the trials associated with included process evaluations, including: aims, inclusion/exclusion criteria, sample size, participant characteristics, study design, intervention and control descriptions, data collection and follow-up time-points and outcome measures used.

RCT aims

Associated trials where sedentary behaviour was measured as an outcome were published between 2007-2020. Five trials focused specifically on reducing sedentary behaviour (19-21) or sitting time(22, 23). The remaining 12 trials aimed to increase physical activity or promote healthier lifestyles but measured sedentary behaviour as an outcome.

Trial location and participant characteristics

Seven trials were conducted in the UK(20-22, 24-27), the remainder in the USA(19, 23, 28), Netherlands(29, 30), Brazil(31), Ireland(32), Canada(33), Hong Kong(34), and Belgium(35). Participants recruited into the trials varied, including: mothers or parents of infants, pregnant women, adults, older adults, overweight adults, individuals with chronic illnesses, and individuals with intellectual disabilities or serious mental illnesses. Most trials included males and females, however three included females only(19, 26, 28). Participants' ages ranged between 30 and 75; the majority of trials included participants aged between 40 and 50 years(19-21, 24, 25, 29, 30, 32, 33).

Included process evaluations

Supplementary file 4 presents data specific to the process evaluations including: aims and whether the process evaluations were pre-specified, sample size and sampling methods, study design and data collection methods, and theoretical frameworks used. These data provide further context for the narrative synthesis.

Thirteen of the 17 process evaluations were pre-specified in published protocols, or trial register records. Five studies(19, 26, 30, 32, 35) were published prior to the MRC guidance for process evaluations(4), the majority were published in the same year or after the guidance was published(20-25, 27-29, 31, 33, 34). Despite this, only four authors cited the MRC guidance(20, 22, 25, 27) and only one reported using this to guide the process evaluation(25). Fourteen out of 17 used the term ‘process evaluation’ within the publication. Three did not use this term(23, 24, 34).

Process evaluation aims

There was considerable variation in process evaluation aims. Some studies had a broad focus on participants’ experiences for example, Elramli (24) aimed to explore participants’ views regarding the effectiveness of a walking intervention for rheumatoid arthritis (RA). Others focused more specifically on barriers to achieving activity goals(28), or barriers and facilitators to the sustainability of an intervention(29). Some focused on the feasibility and/or acceptability of interventions among different participant groups, including those at risk of chronic disease (33); older adults(23); individuals with intellectual disabilities(20); and individuals with serious mental illnesses(21). Only two process evaluations were conducted with a view to refine the intervention(26, 27).

Study design and data collection methods

For peer review only - http://bmjopen.bmj.com/site/about/guidelines.xhtml

As outlined in supplementary file 4, sample sizes of participants recruited to the process evaluations varied, from five(21) to 411(29). A total of 1553 participants were included from intervention groups across the 17 studies and 340 from control groups in four studies(26, 29, 34, 35).

Nine studies(19-22, 25-27, 29, 31) used mixed-methods, most commonly combining quantitative questionnaires with semi-structured interviews (telephone and face-to-face). In five studies, questionnaires were used to ask participants about their satisfaction with the intervention, intervention fidelity, and about suggested improvements to interventions(19, 27, 29, 32, 35). In two studies, questionnaires focused on intervention providers experiences of delivering and participating in interventions(25, 30).

Semi-structured interviews explored intervention contexts, barriers and facilitators to intervention delivery , and experiences from the perspectives of intervention providers, participants, and their family members or carers(21-27, 29, 31, 33, 34). Other methods used included: non-participant observations (19), focus groups(20, 25, 27, 31, 34), healthcare professionals' registries and log books(29).

For peer review only

Methodological quality

Supplementary file 9 provides an overview of the answers to questions in the relevant categories of the MMAT(18) for all included studies. Options include, 'yes', indicating a positive judgement, 'no', indicating a negative judgement, or 'can't tell,' which is used when there is insufficient information to make a judgement. MMAT authors discourage calculating an overall score and excluding studies based on their methodological quality(18). Therefore all studies remained included in the synthesis and were not weighted. Below is a summary of the assessment of each of the six categories.

- Screening questions

The majority of studies had clear research questions or aims, and appropriate data were collected.

1. Qualitative studies

Thirteen of 17 included studies had a qualitative component. Four(21, 26, 27, 34) were rated as not meeting some of the criteria in this category, because the descriptions of the analysis process lacked detail, and it was unclear how authors arrived at their findings. In these studies, findings were commonly presented as a series of quotes, in tables or supplementary files but interpretation was considered too limited to constitute an in-depth analysis.

2. Randomised controlled trials

Each of the included studies was associated with a RCT. This category of the MMAT was used to assess the quality of the trials. The 'can't' tell' option was most commonly used in this section because authors often provided insufficient information to provide an answer, particularly regarding the randomisation process and blinding. Scoring was more mixed within this category and no studies scored yes for all questions.

3. Non-randomized studies

The associated trials were all RCTs; therefore this category was not applicable.

4. Quantitative descriptive studies

Thirteen studies had a quantitative component. Overall, they were rated positively across all questions.

5. Mixed methods studies

We considered studies which used methods meeting the criteria for both categories 1 and 4 as mixed methods studies. This category was only applicable for nine studies. When studies were rated negatively on either the qualitative or quantitative component, it was reflected in the judgement for this category.

Narrative synthesis findings

This section reports on the findings from the included 17 process evaluations coded into the framework and summarised in narrative form. Subheadings based on the key functions of a process evaluation outlined in MRC guidance by Moore et al.,(4) have been applied to organise the data.

Description of the interventions and their causal assumptions

According to Moore et al.(4) a clear description of the intervention and its causal assumptions are an important part of understanding how other factors (e.g. implementation, context and mechanisms of impact) influence outcomes.

Supplementary file 5 describes the content and delivery methods for all the interventions. Intervention delivery periods ranged between 6 weeks and 18 months. All interventions included multiple components, examples include group based educational session combined with email input and self- monitoring tools(19) or one-to-one counselling combined with tailored email input(28). In terms of intervention delivery, interventions commonly incorporated some group based input or support(19, 21, 22, 24-26, 29, 31, 34). Interventions were also delivered by a range of providers including researchers(19), health educators (22, 28), exercise professionals, including personal trainers(20, 29), coaches(21, 23, 33), advisors and nurses(25, 30).

Supplementary file 5 also includes information about the mechanisms by which the interventions are intended to have an effect, and any theoretical underpinnings. All interventions were underpinned by theory or incorporated behaviour change techniques, the most common theory being Social Cognitive Theory(36).

Implementation and delivery approaches

Moore et al.(4) recognise that interventions can have limited effects due to weaknesses in how they are designed, or because they are not properly implemented. This section outlines the extent to which interventions were reported to be delivered as intended, common approaches used in intervention delivery, and whether this reportedly translated into changes in outcomes.

As indicated in file 5, in three studies(21-23) interventions were reportedly delivered as intended. In seven studies,(19, 20, 25, 28-30, 33) adaptations were made to the interventions during the course of the trial. In the remaining seven studies,(24, 26, 27, 31, 32, 34, 35) it was difficult to determine whether there were any adaptations as authors only reported the actual delivery, not the intended delivery.

Approaches for achieving intervention delivery included: ensuring staff were appropriately trained and prepared to deliver the intervention with fidelity(19, 31); tailoring aspects of the programme to individuals and their needs (e.g. ensuring activity consultations are appropriate for those with intellectual disabilities (20)); and allowing for flexibility in delivery methods. For example, in Poston et al.(26), pregnant women were provided with the option of receiving the intervention via phone or email, rather than sessions delivered at the hospital, and in Berendsen et al.(29) coaching meetings as part of the intervention were planned with consideration of holidays and health issues.

For peer review only - http://bmjopen.bmj.com/site/about/guidelines.xhtml

Despite these adaptations for enhancing fidelity, interventions were not always effective in achieving the intended outcomes. For example, in Poston et al.(26) despite flexibility in the delivery mode, objectively measured physical activity and sedentary behaviour did not change in the intervention group. In this particular participant group (pregnant women), the potential to achieve the targeted health outcome, optimal blood glucose level, via dietary changes, was greater than changes in physical activity, including sedentary behaviour, as for some participants increasing their activity led to feelings of discomfort. Similarly, in Matthews et al.(20), although individual tailoring was used, the intervention did not have a significant effect on any of the primary or secondary outcomes including time spent in MVPA and time spent sedentary. It was suggested that the intervention may need to be longer than 12 weeks for individuals with intellectual disabilities. This highlights the importance of understanding more about how an intervention is intended to have an effect, as outlined in the following section.

Mechanisms of impact influencing intervention effectiveness

Moore et al.(4) emphasised the importance of exploring mechanisms through which interventions bring about change, to learn more about how the intervention effects may have occurred and how they may be replicated in similar future interventions. This section outlines the mechanisms were reported across the studies and the extent to which they had an impact on behaviour and outcomes.

Social Cognitive Theory was the most commonly used theory, and the following mechanisms of action were reported in several studies: enhancing self-efficacy by rating confidence in completing goals(19); using behavioural cues e.g. standing up every hour, and leaving the remote at the TV(19); using resources e.g. websites combined with counselling calls to encourage goal setting(28) providing social support in educational sessions or workshops, and input and engagement from carers(19, 20, 22, 24, 28).

However, across the studies, the extent to which these mechanisms had their intended impact on behaviour change varied. In Elramli (24) where the aim of the intervention was increasing daily step count, social support was found to be a key factor in participants who increased their physical activity. However, behaviour change techniques including social support, feedback, and self-monitoring were to a lesser extent associated with reduced sedentary behaviour in those with rheumatoid arthritis (RA). In Matthews et al.(20), where the intervention aimed to increase walking and reduce sedentary behaviour, the component of social support was not effective for adults with intellectual disabilities. In the study by Biddle et al.(22) where the intervention aimed to reduce sitting time, there was no difference in sedentary time at 12 months between intervention and control arms. Reasons for a lack of change in sedentary behaviour included: a preference for adopting physically active behaviours rather than sitting less, and motivational drift after three months. In Adams and Gill(19) which focused on reducing sedentary behaviour and increasing light physical activity, self-efficacy was not shown to be a predictor of change in sedentary behaviour. Behavioural cues, e.g. leaving the remote at the TV, did not always influence behaviours either, because some participants were already doing the cued behaviour, and some did not have a TV(19).

Studies underpinned by the Transtheoretical Model, Theory of Planned Behaviour and Self-Determination theory placed emphasis on encouraging participants to be aware of and monitor their own behaviour(20, 29, 30). Motivational interviewing was used in two studies to prompt participants to find solutions, rather than telling them how to change their behaviour (29, 30). Berendsen et al.(29) found the feasibility of changing physical activity behaviours and dietary habits was not as high as expected and was likely associated with poor adherence. Some participants were unrealistic about how much of their own effort would be required, which influenced attendance at meetings. Lakerveld et al.(30) reported that practice nurses were competent and confident in the delivery of motivational interviewing and participants' satisfaction was high, but even so, almost no effects were seen in the determinants of behaviour change in this population of individuals who were at risk of cardiovascular disease and diabetes.

For peer review only - http://bmjopen.bmj.com/site/about/guidelines.xhtml

In summary, these findings provide some insights into how mechanisms may or may not have an effect on sedentary behaviour, and highlight that it is important to fully understand the complexities of interventions.

Factors including context that facilitate or hinder implementation or how participants respond or interact with the intervention

Moore et al.(4) regard understanding context as an important part of interpreting factors influencing whether interventions are effective. They defined context as anything external to the intervention that may act as a barrier to its implementation or effects. They also considered participants responses to and interactions with the intervention as important mechanisms that could influence outcomes. Drawing on the coding framework, this section is divided into include barriers and facilitators to delivery of interventions, barriers and facilitators to participation and engagement, and understanding of participants experiences from different perspectives.

Barriers to delivery of interventions

Across the studies, there were a range of barriers to delivering interventions, including administrative or scheduling issues and organisational difficulties or challenges. In two studies, planning educational sessions around other commitments including holidays and childcare responsibilities was difficult for staff (24, 34). In Blunt et al.(33) a central research team were involved in scheduling appointments, intending to reduce the workload for coaches. However, this resulted in increasing time spent scheduling and it was recommended that coaches were best placed to take responsibility for their own scheduling(33).

Organisational difficulties were apparent across two studies(20, 31). A community health worker from one of the six health centres in Benedetti et al.(31) described the long absence of a doctor as a turbulent time in the unit, which added difficulties in trying to deliver the intervention. In Matthews et al.(20), the intervention was implemented at a time of significant change within the local learning disability service. Provision of support was affected by the closure of many day centres, which led to a low morale and increasing work pressures among the staff. In Berendsen et al.(29), there were factors that influenced adherence; additionally suspended government financial and policy support meant the programme could not continue.

Barriers to participation and engagement

Across the studies, there was a range of barriers to participation and engagement in the interventions. The most common barriers to engagement were: having a pre-existing illness or injury and associated problems e.g. pain(19, 23-29, 33), having other commitments e.g. work, caring responsibilities(23, 24, 26, 28); and being too tired(22, 26, 33). Other, less common barriers to engagement included loss of accountability for behaviour over time(33), fluctuating mental health(21), and lack of motivation(24).

Some participants also experienced difficulties with pedometers and accelerometers used as an outcome measure for the trial., in terms of understanding how to use them, side effects of wearing them e.g. skin irritation(19, 23) and lost devices(19, 22). In Biddle et al.(22) half the participants experienced problems with the software for the 'Gruve' accelerometer, including: computer synchronisation issues, incompatible computers, website navigation problems, device malfunction, short battery life, and charging issues.

Some barriers may be more applicable to specific groups. For example, in Benedetti et al.(31), a community health worker perceived some older people to be apprehensive about new things which may have been a barrier to participation. In another study, a participant thought that sitting was deserved in old age and he was looking forward to this aspect of retirement to indulge in some of his passions e.g. reading and studying, which made him resent the idea of standing more(23).

For peer review only - http://bmjopen.bmj.com/site/about/guidelines.xhtml

Some barriers were specific to particular contexts. In Elramli,(24) participants who had RA worried about using the gym because they lacked knowledge of suitable, safe exercises. Although workplace interventions were not included in this review, participants who had received educational based interventions reflected on how this applied to other parts of their lives and therefore provided some insight into how the work setting impacts upon sedentariness. For example, participants felt that it was not appropriate to be standing in a work context which could cause embarrassment, e.g. the expectation to be seated for meetings(19, 22, 23). Further barriers at work included having no access to stairs and no standing desks(22).

The context of other parts of everyday life was also influential for some participants who had developed ingrained sedentary habits, as a result of their usual activities or hobbies e.g. reading, eating, socialising, TV viewing, and knitting(23). Religious festivals had an impact on willingness to reduce sitting time at certain times of the year e.g. Christmas and Ramadan(25).

Facilitators to the delivery of interventions

Some of the approaches for achieving implementation and delivery could be regarded as facilitators, including: allowing flexibility in delivery methods, tailoring aspects of the programme to individuals, initial preparation and planning. A range of other factors facilitated intervention delivery.

For example, in Blunt et al.(33), coaches valued the simplicity and structure of the programme. They also appreciated that the programme did not require extensive background knowledge or preparation over and above their existing working requirements. Coaches had the option of referring back to the Canadian Physical Activity Guidelines to ensure they were providing the right level of support to participants. In another study, not requiring too much additional trial focused expertise, and having access to useful trial related resources was valued by social workers(34). In this study the research team prepared and organised most of the materials which facilitated delivery. As a contrast to low morale among staff(20), having a committed team was also important for facilitating delivery(34).

Facilitators to participation or engagement in intervention

There were a range of facilitators to participation and engagement in the interventions. The most common facilitator was support and encouragement from providers and peers; participants valued personal interaction and having someone to keep them on track with the intervention(20, 24, 25, 27, 31, 33).

In some studies, group environments facilitated engagement and provided opportunities for sharing experiences and meeting other peers in a similar situation(21, 24, 27). In Matthews et al.(20), many participants liked one-to-one engagement with intervention providers. This was particularly beneficial to the group who had intellectual disabilities, partly because the conflicting needs of participants in group activities were occasionally disruptive. This group faced challenges to engagement with the intervention, compared to the general population. Matthews et al. suggested the need for providing interventions to people with intellectual disabilities for longer than 12 weeks, so that consultations with providers can address more barriers(20).

Being accountable to someone, e.g. a health coach, also facilitated engagement in three studies because the participants felt being monitored provided motivation(20, 23, 25, 33). Whilst use of a step count monitor was a barrier for some, others found this was a good motivator(23, 24). Adams and Gill(19) recommended that in order for pedometers to be beneficial they need to be more accurate. It was also suggested that technology should be tailored to detect movement in older adults which may be different from younger adults (23).

Participants valued textual resources that were considered attractive through using appropriate text and images (20, 31). Adams and Gill(19) made recommendations for making resources more accessible including

For peer review only - http://bmjopen.bmj.com/site/about/guidelines.xhtml

embedding videos in emails rather than asking participants to use YouTube, and printing cue cards out rather than asking participants to do so themselves. Less common facilitators were: already being involved in health programmes (33), and becoming more aware of the extent of their own sedentary behaviour(23).

Understanding experiences of interventions from different perspectives

Participants

There was some overlap in data coded into barriers and facilitators and participant experiences. The experiences can be divided into positive and negative. Examples of common positive experiences included enjoyment or satisfaction with the intervention programme (19, 21, 31). In some studies, participants described this as life-changing(23, 25) or a new opportunity for learning about how to reduce sedentary behaviour and exercise safely(24). As a result of engaging in the intervention, some participants recognised they had become more aware of the importance of reducing sedentary behaviour(19, 24, 31) and associated benefits e.g. weight loss(21, 23), and reduced stress(23, 34),less fatigue(23), less pain(24), and lower blood sugar(19).

Examples of negative experiences included: feeling stressed or nervous due to wearing a pedometer and a need to check it frequently(24); disliking a type of counselling session because they expected to follow suggestions(30); and feeling nagged by carers to participate(20).

Family/carers

Only two studies included data regarding the experiences of families or carers(20, 34). There was a distinction between the carers' or family members' perceptions of participants' experience and their own experiences as part of an intervention or supporting the intervention. In Matthews et al.(20) family carers talked about how much the participants enjoyed their experiences due to reaching their goals and getting a certificate.

The dynamic was different in another study which included a family-based exercise intervention(34). Participants valued reminding each other as a family to do their exercises.

Staff

There was also some overlap in data coded into barriers and facilitators and staff experiences. Most of staff's perceptions of the participants' experiences were positive. In two studies, staff perceived participants enjoyed using pedometers and diaries(20, 25). Staff voiced their positive perceptions of the programme, e.g. encouraging others and themselves to fit physical activities into their everyday lives(33), and enhancing the participants' family cohesiveness(34). Being involved in delivering the programme also had benefits for some staff . It helped them understand the complexities associated with having a healthy lifestyle(33); and reminded them to stand and move more in their own roles(34).

Some negative experiences overlapped with the barriers to delivering the interventions. These included difficulties with staffing when they were already overcommitted (20, 31); limited venue space for delivering the programme(31); and lack of psychological training to be able to deliver the intervention(29).

DISCUSSION

Summary of findings

For peer review only - http://bmjopen.bmj.com/site/about/guidelines.xhtml

This review aimed to synthesise process evaluations of interventions in trials where sedentary behaviour was measured as an outcome to: develop an understanding of intervention content, mechanisms of impact, implementation and delivery approaches and contexts, in which interventions were reported to be effective or ineffective and explore the experiences of participants, family/carers and intervention staff in such interventions. To address these aims, we synthesised data from 17 studies including a range of participant groups e.g. mothers or parents of infants, pregnant women, adults, older adults, overweight adults, individuals with chronic illnesses including rheumatoid arthritis, intellectual disabilities and serious mental illnesses. Systematic reviews of process evaluations have been conducted in other areas of research e.g. primary care(37) and workplace health promotion programmes(38). However, to our knowledge this review is the first to synthesise data from process evaluations of interventions in trials which measured sedentary behaviour as an outcome in adults.

The review has highlighted the complexity of factors that contribute to implementing interventions with fidelity, and how this links to outcome effects. Common barriers to delivery were those that may be expected in delivery of complex interventions of any kind, not just reducing sedentary behaviour. These included structural changes and staffing pressures within an organisation, and limited funding for providing interventions. Many interventions required some level of input from providers (e.g. researchers, health educators, exercise professionals, coaches and health professionals) to deliver the programme, e.g. scheduled exercise or education sessions. On the other hand, this limited flexibility of a structured intervention posed difficulties amongst some participants who had busy schedules and other priorities. In such cases, delivery was facilitated by providing different options for how the intervention is delivered e.g. via phone or email. However, flexible intervention delivery did not guarantee adherence to the intervention, because participants faced other barriers e.g. discomfort during pregnancy, cognitive difficulties; these factors ultimately impacted on sedentariness.

Whilst it was not our primary intention to synthesise the quantitative findings from the RCTs; the quantitative findings (summarised in supplementary file 7), indicate only three studies reported a statistically significant reduction in sedentary behaviour at the end of the intervention (21, 24, 33). The review identified commonalities across these three interventions that were effective in reducing sedentary behaviour; they all included elements of goal setting and access to support or coaching from a professional. All three were underpinned by theories (social cognitive theory of self-regulation, social cognitive theory and the COM-B model, including a focus on self-efficacy) which in part explain how these interventions may have had their effects (file 5). However other studies also had similar features, were underpinned by similar social cognitive principles including self-efficacy (19, 22, 26, 28)but reported no statistically significant reduction in sedentary behaviour.

This suggests that the process of changing outcomes e.g. sedentary behaviour is complex and influenced by other factors. Complex interventions were traditionally understood as those comprised of multiple components(3). However, context is becoming increasingly recognised as a source of complexity with acknowledgement that interventions are not a discrete package of components, but also a process of changing what complex systems do, including the interactions between individuals (e.g. providers and recipients)(39). Our findings support this notion because whilst all interventions were underpinned by psychological theories focused on individual-level change e.g. social cognitive theory(36), trans-theoretical model(40), theory of planned behaviour(41), self-determination theory(42) and habit formation theory(43); it was evident that a range of wider, contextual factors in addition to individual factors also influenced the implementation and delivery of the intervention as part of complex systems. However, within the included process evaluations, programme theories (including logic models) depicting how the intervention would operate in a particular context were rarely reported. Only one process evaluation reported a logic model(25). Given the complex nature of the delivery and engagement associated with complex interventions, it is important that influences on outcomes such as reduced sedentary behaviour are understood as individual-level behaviour change

For peer review only - http://bmjopen.bmj.com/site/about/guidelines.xhtml

processes, and in context, taking into account the complexities of experiences(44). Ensuring logic models are developed and reported would aid in understanding these complexities.

The identified barriers and facilitators to participation and engagement provide important insights into participants' experiences of interventions and explain what makes interventions more acceptable to some individuals compared to others. The review indicates that social support was important for participants. Some participants valued elements of groups such as meeting others and sharing experiences among similar peers. Others, particularly those with intellectual disabilities, valued one-to one input from providers. Level of motivation was also influential in engagement. Some felt motivated due to being accountable to someone; whilst others felt motivated as a result of tracking activity using a pedometer. However, others disliked pedometers because they struggled to understand the device or experienced skin irritation whilst wearing them. Previous studies have found satisfaction being important for compliance and engagement with tracking devices e.g. pedometers(45, 46) . Results of a national cross sectional survey conducted in Australia suggested that interventions should make sure the devices align with the preferences of the target groups(47) . Our review suggests that individuals with particular conditions could benefit from interventions that are tailored to their symptoms e.g. pain, tiredness and illness.

Changes across the lifespan should also be considered so that interventions can take into account what is appropriate and acceptable for older adults. Our review findings indicate that older people may be more likely to think that sitting down is deserved, or associated with enjoyable hobbies e.g. reading. A recent review by Compernolle et al.(48) focused on older adults perceptions of sedentary behaviour similarly found that sedentariness was motivated by finding enjoyment, and comfort. Their experiences are also shaped by their capabilities, the social opportunities, and motivations in addition to societal expectations that often dictate that for older people sitting is their main mode of living.

Current lifestyles, regardless of age or other characteristics also influence the extent to which participants are likely to engage in behaviours that reduce sedentary behaviour. Our review evidence adds to, and supports findings from another review exploring qualitative experiences of participating in non-workplace interventions(9). Sedentary behaviour is further complicated by seasons and events e.g. celebrations such as Christmas or Ramadan which disrupt normal behaviour patterns, and perhaps lead to less concern with healthy behaviours, even with interventions. A systematic review of factors that influence physical activity and sedentary behaviour in ethnic minority groups in Europe also identified cultural and religious factors as influential in the extent to which individuals were sedentary(49). However, they highlighted that aside from the celebrations and events, some parts of religious activity e.g. walking to religious sites for prayers actually facilitated reduced sedentary behaviour and increased physical activity.

Looking across the barriers and facilitators identified in this review and the wider literature, a range of factors need to be considered, highlighting how difficult it is to develop interventions that are suitable for participants, even those with apparently similar characteristics. The consolidated framework for implementation research (CFIR) is an example of a taxonomy of constructs, organised into five domains (intervention, inner setting, outer setting, individual characteristics, and process) that has been devised to understand what influences implementation that could be applied to further understand such complexities(50). Interventions require some level of adaptation to the context and may need to be tailored to participants, including those share similar characteristics, e.g. those with rheumatoid arthritis or intellectual disabilities. They also need to consider the dynamic between staff, participants and families as part of working towards a shared goal (e.g. reducing sedentary behaviour). However, tailoring interventions can be challenging. It can be expensive while material and staffing resources are often limited. If we are to reach a point where reducing sedentary behaviour becomes habitual once interventions cease, participants will need simple strategies and support to take ownership of their own behaviour so they can sustain the lifestyle changes within the context of their lives and their preferences.

For peer review only - http://bmjopen.bmj.com/site/about/guidelines.xhtml

Strengths and limitations

This is the first systematic review to synthesise data from process evaluations evaluating interventions in trials that measure sedentary behaviour as an outcome in adults. Robust methods were used throughout the conduct of the review. A comprehensive search strategy was developed with input from an information specialist; two reviewers independently screened search results and assessed the quality of included studies.

Although a large proportion of the trials on which the process evaluations were based were conducted in the UK, the inclusion of studies from other countries (e.g. USA, Netherlands, Brazil, and Hong Kong) mean these findings are relevant for researchers internationally. The inclusion of males and females enhances the applicability of the findings in terms of gender. However with regards to age, the majority of studies included participants between 40 and 50 years; therefore not all findings are applicable to other age groups. The inclusion of participants from various groups can be regarded as both a strength and limitation of this review. Findings may be of interest to experts in different research areas; however it is difficult to draw firm conclusions for particular population groups, especially where sample sizes are small.

There was an overall lack of consistency in how process evaluations were reported. Fourteen out of 17 used the term 'process evaluation' within the publication. Three did not use this term(23, 24, 34), although they met the criteria for inclusion in that they aimed to explore participants' views on the factors that influence intervention effectiveness (24, 34), including the feasibility and acceptability of the intervention. Others have similarly critiqued process evaluations in primary care(37), suggesting a need for more consistency to produce higher quality evaluations that would inform practice and be more comparable in future reviews of this nature.

The assessments using the MMAT also indicated some variation in the quality of the process evaluations. The four studies that were considered lowest quality had poorer qualitative components(21, 26, 27, 34) that lacked detail and depth, and had limited interpretation. When studies were rated negatively on the qualitative component, it was reflected in the judgement in the mixed methods category in the MMAT. Only four studies(20, 22, 25, 27) cited the MRC guidance for process evaluations (4) but this did not equate to better quality. Only one study(25) used the framework to guide the evaluation whereas the other three only made reference to it in the introduction.

More than 24 tools are available to assess the quality of systematic reviews; however, there remains no clear guidance for which tool to use for assessing the quality of process evaluations (51). The MMAT(18) was a logical choice as it is appropriate for mixed methods studies and those using either qualitative or quantitative data. However, it has not been designed to require detailed commentary about judgements of quality. Therefore a simplified account of quality is presented. Yet, it is difficult to compare studies without looking across all the domains because the authors do not recommend calculating an overall score(18). It was also recommended that studies should not be excluded based on their quality(18), accordingly all studies were included in the synthesis. Researchers could benefit from considering the strengths and limitations of the MMAT when interpreting findings from this review. In our view there is also a need to develop guidelines specific to systematically reviewing process evaluations of complex interventions.

CONCLUSIONS

There is a wealth of existing evidence which synthesises the findings from trials evaluating interventions that have measured sedentary behaviour as an outcome in adults. To our knowledge this review is the first to synthesise data from the process evaluations of such interventions. This review complements existing trial evidence because it highlights a range of factors associated with implementation, context, and participants experiences that can contribute to whether an intervention is effective or not.

For peer review only - http://bmjopen.bmj.com/site/about/guidelines.xhtml

It is promising that all interventions were underpinned by theory as part of understanding how they were intended to have an effect, however it is important to acknowledge how different contexts and individual level factors e.g. health status, illness, age, and lifestyles can shape levels of engagement and behaviour change. Researchers could benefit from using a process evaluation framework such as Moore et al's,(4) for conducting and reporting process evaluations to ensure all factors are considered. Including logic model as part of the process evaluation would also assist in mapping the range of factors that contribute to changes in intervention outcomes.

FIGURES:

Figure 1: PRISMA flow diagram

ADDITIONAL

Acknowledgements: We acknowledge the help and support of our Information Scientist, Deirdre Andre, University of Leeds. We also thank Dr Rekesh Corepal (RC) for his contributions when the original search was conducted in 2019. We are grateful for the funding provided by the National Institute for Health Research (NIHR).

Author Contributions: This systematic review was conceived and designed by members of the RECREATE Programme Management Group (AF, CE, CF, RL, DJC) and researchers (JFH, NL, JH, SM). The systematic review process was conducted by JFH, NL, SO with oversight and input from DJC. JFH drafted the initial manuscript with input from NL and DJC. All authors have critically reviewed and revised different versions of the manuscript (JFH, NL, SO, JH, SM, CE, CF, RL, AF, DJC).

Competing interests: None declared

Funding: This report is independent research funded by the National Institute for Health Research (Programme Grants for Applied Research, Development and evaluation of strategies to reduce sedentary behaviour in patients after stroke and improve outcomes, RP-PG-0615-20019).

Data availability statement: All data relevant to the study are included in the article or uploaded as supplementary information

Patient consent for publication: Not required

Ethics approval: None required as this is a systematic review which synthesises data from previously published research.

For peer review only

For peer review only - http://bmjopen.bmj.com/site/about/guidelines.xhtml

REFERENCES

1. Tremblay MS, Aubert S, Barnes JD, Saunders TJ, Carson V, Latimer-Cheung AE, et al. Sedentary behavior research network (SBRN)–terminology consensus project process and outcome. International Journal of Behavioral Nutrition and Physical Activity. 2017;14(1):75.
2. Owen N, Healy GN, Matthews CE, Dunstan DW. Too much sitting: the population-health science of sedentary behavior. Exercise and sport sciences reviews. 2010;38(3):105.
3. Craig P, Dieppe P, Macintyre S, Michie S, Nazareth I, Petticrew M. Developing and evaluating complex interventions: the new Medical Research Council guidance. 2013.
4. Moore GF, Audrey S, Barker M, Bond L, Bonell C, Hardeman W, et al. Process evaluation of complex interventions: Medical Research Council guidance. bmj. 2015;350.
5. Petticrew M. When are complex interventions 'complex'? When are simple interventions 'simple'? : Oxford University Press; 2011.
6. Shepperd S, Lewin S, Straus S, Clarke M, Eccles MP, Fitzpatrick R, et al. Can we systematically review studies that evaluate complex interventions? PLoS Med. 2009;6(8):e1000086.
7. Prince S, Saunders T, Gresty K, Reid R. A comparison of the effectiveness of physical activity and sedentary behaviour interventions in reducing sedentary time in adults: a systematic review and meta-analysis of controlled trials. Obesity Reviews. 2014;15(11):905-19.
8. Shrestha N, Grgic J, Wiesner G, Parker A, Podnar H, Bennie JA, et al. Effectiveness of interventions for reducing non-occupational sedentary behaviour in adults and older adults: a systematic review and meta-analysis. British journal of sports medicine. 2019;53(19):1206-13.
9. Rawlings G, Williams R, Clarke D, English C, Fitzsimons C, Holloway I, et al. Exploring adults' experiences of sedentary behaviour and participation in non-workplace interventions designed to reduce sedentary behaviour: a thematic synthesis of qualitative studies. BMC public health. 2019;19(1):1099.
10. Moher D, Shamseer L, Clarke M, Ghersi D, Liberati A, Petticrew M, et al. Preferred reporting items for systematic review and meta-analysis protocols (PRISMA-P) 2015 statement. Systematic reviews. 2015;4(1):1.
11. Moore G, Audrey S, Barker M, Bond L, Bonell C, Hardeman W, et al. Process evaluation of complex interventions: UK Medical Research Council (MRC) guidance. London: MRC. 2014.
12. Hall J.F, Corepal R, Crocker T.F, Lam N, L B, Birch K, et al. A systematic review and meta-analysis of non-workplace interventions to reduce time spent sedentary in adults. 2021 Submitted to medRxiv. https://www.medrxiv.org/2021.
13. Corepal R, Hall JF, English C, Farrin A, Fitzsimons CF, Forster A, et al. A protocol for a systematic review of process evaluations of interventions investigating sedentary behaviour in adults. BMJ open. 2019;9(9):e031291.
14. Robbins LB, Ling J, Toruner EK, Bourne KA, Pfeiffer KA. Examining reach, dose, and fidelity of the "Girls on the Move" after-school physical activity club: A process evaluation. BMC public health. 2016;16(1):671.
15. Walton H, Spector A, Williamson M, Tombor I, Michie S. Developing quality fidelity and engagement measures for complex health interventions. British journal of health psychology. 2020;25(1):39-60.
16. International Q. NVivo Qualitative Data Analysis Software (Version 12). . 2019.
17. Lincoln YS, Guba EG. But is it rigorous? Trustworthiness and authenticity in naturalistic evaluation. New directions for program evaluation. 1986;1986(30):73-84.
18. Hong QN, Pluye P, Fàbregues S, Bartlett G, Boardman F, Cargo M, et al. Mixed methods appraisal tool (MMAT), version 2018. Registration of copyright. 2018;1148552.
19. Adams MM, Gill DL. On our feet: Feasibility trial of an intervention to reduce sedentary behavior and increase physical activity. 2012(3511147):332.
20. Matthews L, Mitchell F, Stalker K, McConnachie A, Murray H, Melling C, et al. Process evaluation of the Walk Well study: a cluster-randomised controlled trial of a community based walking programme for adults with intellectual disabilities. BMC Public Health. 2016;16:527.
21. Williams J, Stubbs B, Richardson S, Flower C, Barr-Hamilton L, Grey B, et al. 'Walk this way': results from a pilot randomised controlled trial of a health coaching intervention to reduce sedentary behaviour and increase physical activity in people with serious mental illness. BMC Psychiatry. 2019;19(1):287.
22. Biddle SJ, Edwardson CL, Gorely T, Wilmot EG, Yates T, Nimmo MA, et al. Reducing sedentary time in adults at risk of type 2 diabetes: process evaluation of the STAND (Sedentary Time ANd Diabetes) RCT. BMC Public Health. 2017;17(1):80.
23. Matson TE, Renz AD, Takemoto ML, McClure JB, Rosenberg DE. Acceptability of a sitting reduction intervention for older adults with obesity. BMC public health. 2018;18(1):706-.

For peer review only - http://bmjopen.bmj.com/site/about/guidelines.xhtml

24. Elramli A. Effectiveness of community based physical activity on step count and sedentary behaviour in people with rheumatoid arthritis within the first five years of diagnosis [PhD]. Glasgow: University of Glasgow; 2017.
25. Harris TK, S; Victor, C; Iliffe, S; Ussher, M; Fox-Rushby, J; Whincup, P; Ekelund, U; Furness, C; Limb, E; Anokye, N; Ibison, J; DeWilde, S; David, L; Howard, E; Dale, R; Smith, J; Normansell, R; Beighton, C; Morgan, K; Wahlich, C; Sanghera, S; Cook, D. A pedometer-based walking intervention in 45- to 75-year-olds, with and without practice nurse support: the PACE-UP three-arm cluster RCT. ASSESSMENT HT; 2018. Contract No.: ISSN 1366-5278.
26. Poston L, Briley AL, Barr S, Bell R, Croker H, Coxon K, et al. Developing a complex intervention for diet and activity behaviour change in obese pregnant women (the UPBEAT trial); assessment of behavioural change and process evaluation in a pilot randomised controlled trial. BMC Pregnancy Childbirth. 2013;13:148.
27. Stathi A, Withall J, Thompson JL, Davis MG, Gray S, De Koning J, et al. Feasibility Trial Evaluation of a Peer Volunteering Active Aging Intervention: ACE (Active, Connected, Engaged). Gerontologist. 2019.
28. Albright CL, Saiki K, Steffen AD, Woekel E. What barriers thwart postpartum women's physical activity goals during a 12-month intervention? A process evaluation of the Na Mikimiki Project. Women Health. 2015;55(1):1-21.
29. Berendsen BA, Kremers SP, Savelberg HH, Schaper NC, Hendriks MR. The implementation and sustainability of a combined lifestyle intervention in primary care: mixed method process evaluation. BMC Family Practice. 2015;16:37.
30. Lakerveld J, Bot S, Chinapaw M, van Tulder M, Kingo L, Nijpels G. Process evaluation of a lifestyle intervention to prevent diabetes and cardiovascular diseases in primary care. Health Promot Pract. 2012;13(5):696-706.
31. Benedetti TRB, Rech CR, Konrad LM, Almeida FA, Brito FA, Chodzko-Zajko W, et al. Re-thinking Physical Activity Programs for Older Brazilians and the Role of Public Health Centers: A Randomized Controlled Trial Using the RE-AIM Model. Front Public Health. 2020;8:48-.
32. Lane A, Murphy N, Bauman A, Chey T. Randomized controlled trial to increase physical activity among insufficiently active women following their participation in a mass event. Health education journal. 2010;69(3):287-96.
33. Blunt W, Gill DP, Riggin B, Brown JB, Petrella RJ. Process evaluation of the HealtheStepsTM lifestyle prescription program. Translational behavioral medicine. 2019;9(1):32-40.
34. School of Public Health TUoHK. Be Healthy, So Easy: Family Education Project. Hong Kong: The University of Hong Kong; 2017.
35. Spittaels H, De Bourdeaudhuij I, Brug J, Vandelanotte C. Effectiveness of an online computer-tailored physical activity intervention in a real-life setting. Health Education Research. 2007;22(3):385-96.
36. Bandura A. Social foundations of thought and action. Englewood Cliffs, NJ. 1986;1986(23-28).
37. Liu H, Mohammed A, Shanthosh J, Laba T-L, Hackett ML, Peiris D, et al. Process evaluations of primary care interventions addressing chronic disease: a systematic review. BMJ open. 2019;9(8):e025127.
38. Wierenga D, Engbers LH, Van Empelen P, Duijts S, Hildebrandt VH, Van Mechelen W. What is actually measured in process evaluations for worksite health promotion programs: a systematic review. BMC public health. 2013;13(1):1190.
39. Moore GF, Evans RE, Hawkins J, Littlecott H, Melendez-Torres G, Bonell C, et al. From complex social interventions to interventions in complex social systems: future directions and unresolved questions for intervention development and evaluation. Evaluation. 2019;25(1):23-45.
40. Prochaska JO, Marcus BH. The transtheoretical model: Applications to exercise. 1994.
41. Ajzen I. From intentions to actions: A theory of planned behavior. Action control: Springer; 1985. p. 11-39.
42. Ryan RM, Deci EL. Self-determination theory and the facilitation of intrinsic motivation, social development, and well-being. American psychologist. 2000;55(1):68.
43. Lally P, Wardle J, Gardner B. Experiences of habit formation: a qualitative study. Psychology, health & medicine. 2011;16(4):484-9.
44. Moore GF, Evans RE, Hawkins J, Littlecott HJ, Turley R. All interventions are complex, but some are more complex than others: using iCAT_SR to assess complexity. Cochrane Database of Systematic Reviews. 2017(7).
45. Fairclough S, Noonan R, Rowlands A, Van Hees V, Knowles Z, Boddy L. Wear compliance and activity in children wearing wrist and hip mounted accelerometers. Medicine & Science in Sports & Exercise. 2016;48(2):245-53.

For peer review only - http://bmjopen.bmj.com/site/about/guidelines.xhtml

46. Hassani M, Kivimaki M, Elbaz A, Shipley M, Singh-Manoux A, Sabia S. Non-consent to a wrist-worn accelerometer in older adults: the role of socio-demographic, behavioural and health factors. PLoS One. 2014;9(10):e110816.
47. Alley S, Schoeppe S, Guertler D, Jennings C, Duncan MJ, Vandelanotte C. Interest and preferences for using advanced physical activity tracking devices: results of a national cross-sectional survey. BMJ open. 2016;6(7):e011243.
48. Compernolle S, De Cocker K, Cardon G, De Bourdeaudhuij I, Van Dyck D. Older adults' perceptions of sedentary behavior: A systematic review and thematic synthesis of qualitative studies. The Gerontologist. 2020;60(8):e572-e82.
49. Langøien LJ, Terragni L, Rugseth G, Nicolaou M, Holdsworth M, Stronks K, et al. Systematic mapping review of the factors influencing physical activity and sedentary behaviour in ethnic minority groups in Europe: a DEDIPAC study. International Journal of Behavioral Nutrition and Physical Activity. 2017;14(1):1-24.
50. Damschroder LJ, Aron DC, Keith RE, Kirsh SR, Alexander JA, Lowery JC. Fostering implementation of health services research findings into practice: a consolidated framework for advancing implementation science. Implementation science. 2009;4(1):1-15.
51. Shea B, Dubé C, Moher D. Assessing the quality of reports of systematic reviews: the QUOROM statement compared to other tools. Systematic Reviews in Health Care: Meta-Analysis in Context. 2001:122-39.

For peer review only

For peer review only - http://bmjopen.bmj.com/site/about/guidelines.xhtml

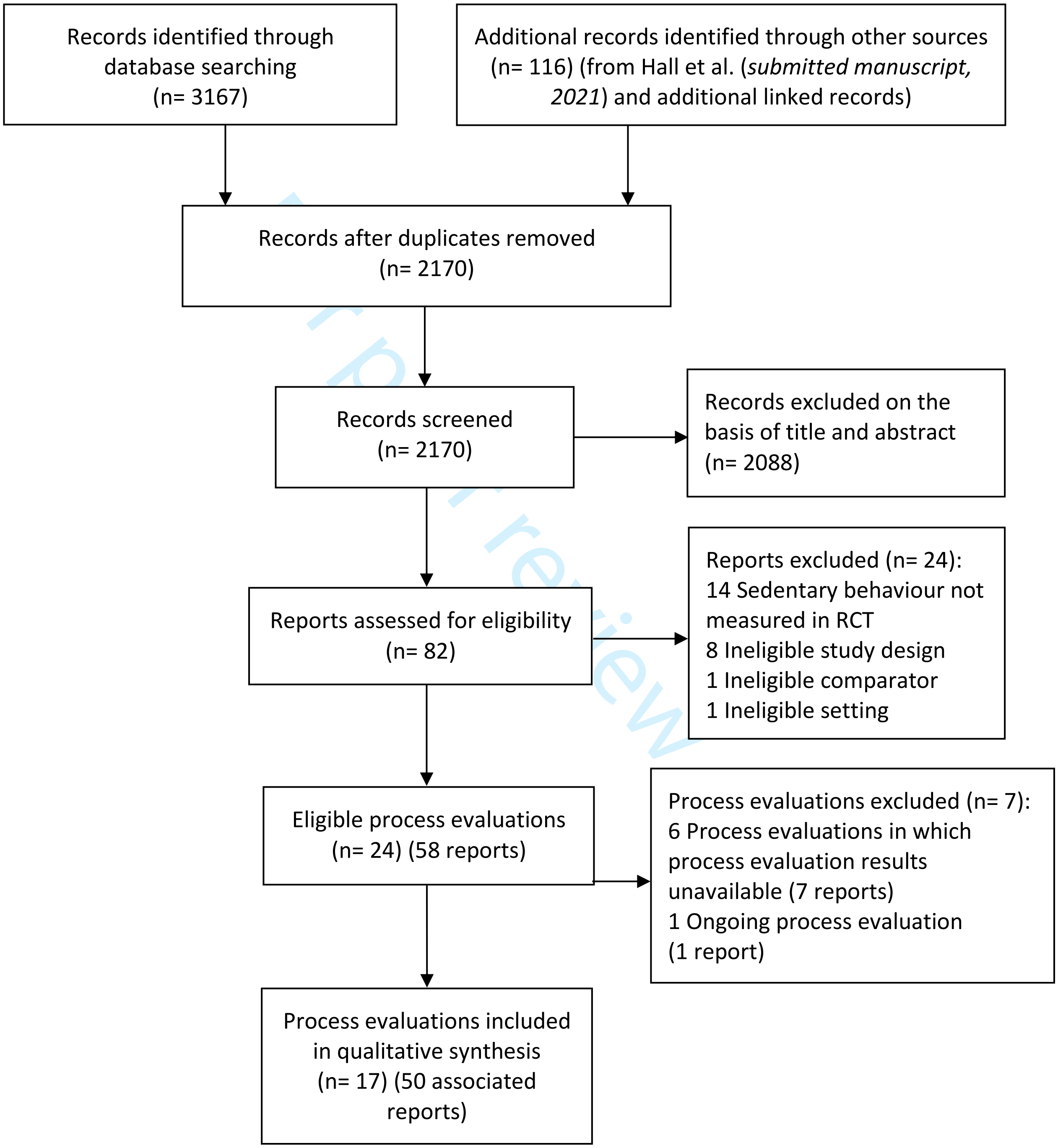
Records identified through database searching (n= 3167)
Additional records identified through other sources (n= 116) (from Hall et al. (*submitted manuscript, 2021*) and additional linked records)
Records after duplicates removed (n= 2170)
Records screened (n= 2170)
Records excluded on the basis of title and abstract (n= 2088)
Reports assessed for eligibility (n= 82)
Reports excluded (n= 24):
14 Sedentary behaviour not measured in RCT
8 Ineligible study design
1 Ineligible comparator
1 Ineligible setting
Eligible process evaluations (n= 24) (58 reports)
Process evaluations excluded (n= 7):
6 Process evaluations in which process evaluation results unavailable (7 reports)
1 Ongoing process evaluation (1 report)
Process evaluations included in qualitative synthesis (n= 17) (50 associated reports)

Supplementary file 1: PRISMA 2020 Checklist 27.05.21

Section and Topic	Item #	Checklist item	Location where item is reported
**TITLE**			
Title	1	Identify the report as a systematic review.	Title, page 1
**ABSTRACT**			
Abstract	2	See the PRISMA 2020 for Abstracts checklist.	Pg. 1
**INTRODUCTION**			
Rationale	3	Describe the rationale for the review in the context of existing knowledge.	Pg. 2/3
Objectives	4	Provide an explicit statement of the objective(s) or question(s) the review addresses.	Pg. 2/3
**METHODS**			
Eligibility criteria	5	Specify the inclusion and exclusion criteria for the review and how studies were grouped for the syntheses.	Pg. 3
Information sources	6	Specify all databases, registers, websites, organisations, reference lists and other sources searched or consulted to identify studies. Specify the date when each source was last searched or consulted.	Pg. 4
Search strategy	7	Present the full search strategies for all databases, registers and websites, including any filters and limits used.	Supplementary file 2
Selection process	8	Specify the methods used to decide whether a study met the inclusion criteria of the review, including how many reviewers screened each record and each report retrieved, whether they worked independently, and if applicable, details of automation tools used in the process.	Pg. 4
Data collection process	9	Specify the methods used to collect data from reports, including how many reviewers collected data from each report, whether they worked independently, any processes for obtaining or confirming data from study investigators, and if applicable, details of automation tools used in the process.	Pgs. 4-7
Data items	10a	List and define all outcomes for which data were sought. Specify whether all results that were compatible with each outcome domain in each study were sought (e.g. for all measures, time points, analyses), and if not, the methods used to decide which results to collect.	n/a
	10b	List and define all other variables for which data were sought (e.g. participant and intervention characteristics, funding sources). Describe any assumptions made about any missing or unclear information.	n/a
Study risk of bias assessment	11	Specify the methods used to assess risk of bias in the included studies, including details of the tool(s) used, how many reviewers assessed each study and whether they worked independently, and if applicable, details of automation tools used in the process.	Methodological quality pg. 7
Effect measures	12	Specify for each outcome the effect measure(s) (e.g. risk ratio, mean difference) used in the synthesis or presentation of results.	n/a
Synthesis methods	13a	Describe the processes used to decide which studies were eligible for each synthesis (e.g. tabulating the study intervention characteristics and comparing against the planned groups for each synthesis (item #5)).	Pgs. 4-7
	13b	Describe any methods required to prepare the data for presentation or synthesis, such as handling of missing summary statistics, or data conversions.	n/a
	13c	Describe any methods used to tabulate or visually display results of individual studies and syntheses.	Pgs. 4-7
	13d	Describe any methods used to synthesize results and provide a rationale for the choice(s). If meta-analysis was performed, describe the model(s), method(s) to identify the presence and extent of statistical heterogeneity, and software package(s) used.	Pgs. 4-7
	13e	Describe any methods used to explore possible causes of heterogeneity among study results (e.g. subgroup analysis, meta-regression).	n/a
	13f	Describe any sensitivity analyses conducted to assess robustness of the synthesized results.	n/a
Reporting bias assessment	14	Describe any methods used to assess risk of bias due to missing results in a synthesis (arising from reporting biases).	Supplementary file 9

For peer review only - http://bmjopen.bmj.com/site/about/guidelines.xhtml

Supplementary file 1: PRISMA 2020 Checklist 27.05.21

Section and Topic	Item #	Checklist item	Location where item is reported
Certainty assessment	15	Describe any methods used to assess certainty (or confidence) in the body of evidence for an outcome.	n/a
**RESULTS**			
Study selection	16a	Describe the results of the search and selection process, from the number of records identified in the search to the number of studies included in the review, ideally using a flow diagram.	Pg. 8
	16b	Cite studies that might appear to meet the inclusion criteria, but which were excluded, and explain why they were excluded.	Supplementary file 4
Study characteristics	17	Cite each included study and present its characteristics.	Supplementary files 3 and 4
Risk of bias in studies	18	Present assessments of risk of bias for each included study.	Supplementary file 9
Results of individual studies	19	For all outcomes, present, for each study: (a) summary statistics for each group (where appropriate) and (b) an effect estimate and its precision (e.g. confidence/credible interval), ideally using structured tables or plots.	n/a
Results of syntheses	20a	For each synthesis, briefly summarise the characteristics and risk of bias among contributing studies.	n/a
	20b	Present results of all statistical syntheses conducted. If meta-analysis was done, present for each the summary estimate and its precision (e.g. confidence/credible interval) and measures of statistical heterogeneity. If comparing groups, describe the direction of the effect.	n/a
	20c	Present results of all investigations of possible causes of heterogeneity among study results.	n/a
	20d	Present results of all sensitivity analyses conducted to assess the robustness of the synthesized results.	n/a
Reporting biases	21	Present assessments of risk of bias due to missing results (arising from reporting biases) for each synthesis assessed.	Supplementary file 9
Certainty of evidence	22	Present assessments of certainty (or confidence) in the body of evidence for each outcome assessed.	n/a
**DISCUSSION**			
Discussion	23a	Provide a general interpretation of the results in the context of other evidence.	Pgs. 15-17
	23b	Discuss any limitations of the evidence included in the review.	Pg. 18
	23c	Discuss any limitations of the review processes used.	Pg. 18
	23d	Discuss implications of the results for practice, policy, and future research.	Pgs. 15-19
**OTHER INFORMATION**			
Registration and protocol	24a	Provide registration information for the review, including register name and registration number, or state that the review was not registered.	Pg. 3
	24b	Indicate where the review protocol can be accessed, or state that a protocol was not prepared.	Pg. 3
	24c	Describe and explain any amendments to information provided at registration or in the protocol.	n/a
Support	25	Describe sources of financial or non-financial support for the review, and the role of the funders or sponsors in the review.	Pg. 19
Competing interests	26	Declare any competing interests of review authors.	n/a
Availability of data, code and	27	Report which of the following are publicly available and where they can be found: template data collection forms; data extracted from included studies; data used for all analyses; analytic code; any other materials used in the review.	Pg. 19

For peer review only

Supplementary file 1: PRISMA 2020 Checklist 27.05.21

Section and Topic	Item #	Checklist item	Location where item is reported
other materials			

From: Page MJ, McKenzie JE, Bossuyt PM, Boutron I, Hoffmann TC, Mulrow CD, et al. The PRISMA 2020 statement: an updated guideline for reporting systematic reviews. BMJ 2021;372:n71. doi: 10.1136/bmj.n71

For more information, visit: http://www.prisma-statement.org/

For peer review only

Supplementary file 2_search strategy MEDLINE_27.05.21

Database: Ovid MEDLINE(R) <1946 to May 2020>

Search Strategy:

----

1 Sedentary Lifestyle/ (7525)

2 (sedentary or sitting or sedentariness or sedentarism).ti. (6452)

3 ((sedentary or sitting or seated) adj5 (behavio* or lifestyle or life-style)).tw. (7249)

4 ((inactiv* or no exercise or nonexercise or non exercise) adj3 (adult? or men or women or males or females or individuals or people)).tw. (2515)

5 (sedentary adj3 (adult? or men or women or males or females or individuals or people or population?)).tw. (4859)

6 ((sitting or sit or seated or stationary or standing) adj3 (task* or time or bout* or work* or break*)).tw. (4603)

7 low energy expenditure.tw. (144)

8 physical* inactiv*.tw. (6591)

9 (leisure time adj5 (physical* activ* or passive or inactiv*)).tw. (3445)

10 "physical activity level*".tw. (6404)

11 ((sitting or lying) adj2 posture*).tw. (998)

12 (prolong* adj2 (reclin* or sit or sitting or seated)).tw. (564)

13 chair rise?.tw. (323)

14 "sit* less".tw. (601)

15 ((light or low) adj "physical activ*").tw. (1853)

16 ((decrease or reduc* or discourag* or lessen*) adj3 (sit or sitting or stand or standing or physical* inactiv*)).tw. (1377)

17 (time adj5 (computer* or television or tv or video game? or videogame? or gaming or screen or media)).tw. (8936)

18 ((watch* or view*) adj5 (television or tv)).tw. (4240)

19 (play* adj5 (video game? or videogame? or computer game?)).tw. (1305)

20 ((computer* or television or tv or video game? or videogame? or gaming) and (sedentary or physical* activity* or sitting or seated or underactiv* or under activ*)).ti. (351)

21 or/1-20 [sedentary behaviour terms] (50991)

22 Program Evaluat*.mp. (62861)

23 "Outcome and Process Assessment (Health Care)"/ (25572)

For peer review only

24 "Process Assessment (Health Care)"/ (4358)

25 process evaluat*.mp. (2608)

26 or/22-25 [process evaluation] (91311)

27 randomized controlled trial.pt. (476630)

28 controlled clinical trial.pt. (92914)

29 randomized.ab. (377791)

30 placebo.ab. (177752)

31 drug therapy.fs. (2086845)

32 randomly.ab. (262246)

33 trial.ab. (392148)

34 groups.ab. (1631334)

35 27 or 28 or 29 or 30 or 31 or 32 or 33 or 34 (4041965)

36 exp animals/ not humans.sh. (4552221)

37 35 not 36 [Cochrane RCT filter 2008, sensitivity maximimising] (3448772)

38 21 and 26 and 37 [sedentary behaviour and process evaluation and RCTs] (420)

..

For peer review only

Supplementary file 3. Characteristics of 17 included RCTs_27.05.21

Randomised Control Trials									
**Study** *(Authors (Year),* **Country** *(of process evaluation report)*	**Study aims**	**Inclusion/exclusion criteria**	**Sample size, n assigned to intervention /control**	**Participant characteristics** *(Age (mean (SD) or %), Gender (% female), Ethnicity)*	**Study design, RCT type, group, setting**	**Intervention description** *(Content, duration)*	**Control description**	**Data collection and follow ups** *(time-points)*	**Outcome measures for treatment effects** *(pre-specified or those only reported)*
**Adams (2012)**<br><br>USA	Reduce sedentary behaviour, increase light physical activity.<br><br>(Feasibility trial)	**Inclusion:**<br>1. Women between the ages of 35-85;<br>2. BMI >25;<br>3. Be willing to receive intervention materials and messages by email;<br>4. Plan to attend all program and data collection sessions.<br>**Exclusion:**<br>Any reported conditions that prohibited standing or walking.	75<br><br>I: 47<br>C: 28	**Age:**<br>I: 56.73 (12.64)<br>C: 61.38 (12.1)<br><br>**Gender:**<br>100%<br><br>**Ethnicity:**<br>89% Caucasian<br>11% African-American	Cluster randomised controlled<br><br>Weight-loss support club (cluster unit)	On Our Feet intervention – combination of 2 face-to-face interactive group sessions, and 6 weekly email messages.<br><br>6 weeks	Waiting list	Baseline<br>6 weeks	1. Time spent in SB; light and moderate PA (accelerometer; IPAQ, Godin Leisure-Time Activity Questionnaire);<br>2. Participant's self-rated level of confidence for reducing sitting and increasing PA behaviours;<br>3. BMI and waist circumference.
**Albright (2015)**<br><br>USA	Increase moderate to vigorous physical activity.	**Inclusion:**<br>1. Mother of infant aged 2-12 months;<br>2. Inactive (<30 minutes of MVPA/week);<br>3. Healthy, able to do moderate intensity physical activity;<br>4. BMI =18.5-40;<br>5. Not planning to become pregnant in the next 12 months;<br>6. Aged 18-45;<br>7. Had health insurance;<br>8. Read/understood English;	311<br><br>I: 154<br>C: 157	**Age:**<br>I: 31.6 (5.5)<br>C: 32.1 (5.9)<br><br>**Gender:**<br>100%<br><br>**Ethnicity:**<br>31.5% Native Hawaiian/ Pacific Islander<br>33.8% Asian (Japanese, Filipino, other Asian)	Randomised controlled<br><br>Parallel groups<br><br>Community	Tailored telephone counselling, information on website, and pedometer.<br><br>12 months	Information in print or standard website.	Baseline<br>1 month<br>3 months<br>6 months<br>12 months (immediately after intervention)<br>18 months	1. Time spent in MVPA (Active Australia Survey; accelerometer; exercise log);<br>2. Time spent sitting while travelling; at work; watching TV, etc. (Active Australia Survey);<br>3. Body mass index;<br>4. Self-efficacy for PA (instrument designed to assess self-confidence to overcome barriers to PA, modified with questions tailored to new mothers);

For peer review only

Supplementary file 3. Characteristics of 17 included RCTs_27.05.21

		9. Physician's written approval if history of contraindicated conditions.<br>**Exclusion:**<br>1. Pregnant;<br>2. Planning to leave Oahu, Hawaii in the next year (permanently move away);<br>3. Diagnosis of cancer, coronary heart disease (including atrial fibrillation), insulin-dependent diabetes mellitus (IDDM), and other atherosclerotic cardiovascular diseases (e.g., stroke).		16.4% Mixed race<br>15.1% White<br>2.6% Black/ Native American<br>0.6% Unknown					5. Psychosocial mediators survey.
**Benedetti (2020)**<br><br>Brazil	Improve physical activity level.	**Inclusion:**<br>1. Aged ≥60;<br>2. No severe physical and/or mental health impairments;<br>3. Had not participated in physical activity programs in the past 6 months.<br>**Exclusion:**<br>History of heart attack and/or stroke in the past 6 months, cancer diagnosis and/or other severe medical conditions.	114<br><br>BCG: 36<br>TEG: 52<br>C: 26	**Age:**<br>BCG: 69.7 (6.9)<br>TEG: 71.3 (7.3)<br>C: 67.2 (5.8)<br><br>**Gender:** 80.7%<br><br>**Ethnicity:**<br>Not reported	Cluster randomised controlled<br><br>Public health centres (cluster unit)	BCG: 12 weekly meetings behavioural change programme that was adapted from "Active Living Every Day" from USA.<br><br>TEG: 12-week (3 times per week) exercise class conducted at local HCs.	No intervention	Baseline<br>3 months<br>6 months<br>12 months	1. Time spent in SB; light PA; and MVPA (accelerometers);<br>2. BMI;<br>3. Quality of life (WHOQOL-BREF and WHOQOL-OLD).
**Berendsen (2015)**<br><br>The Netherlands	Improve physical activity and dietary behaviour.	**Inclusion:**<br>1. Weight-related health risk;<br>2. Inactive lifestyle (not doing 30 minutes moderate physical activity for at least 5 days per week);<br>3. Motivated for behavioural change;	411<br><br>I: 247<br>C: 164	**Age:**<br>I: 55.9 (12.3)<br>C: 53.8 (12.4)<br><br>**Gender:**<br>64.7%<br><br>**Nationality:**	Cluster randomised controlled<br><br>GP practices (Cluster	Supervised exercise programme based on BeweegKuur – individual and group meetings with lifestyle advisor, dietitian,	Start-up exercise programme based on BeweegKuur – same number of meetings	Activity monitor, physiological measures:<br>Baseline<br>12 months<br>24 months	1. Time spent PA (accelerometer; IPAQ), sedentary, standing or active (accelerometer);<br>2. Dietary habits;<br>3. Quality of Life (EQ-6D);<br>4. Medication;<br>5. Side-effects;

For peer review only

Supplementary file 3. Characteristics of 17 included RCTs_27.05.21

		4. BMI= 25-30, with a large waist circumference (men greater than 102 cm, women greater than 88 cm) with comorbidity (cardiovascular disease and/or T2DM, arthrosis and sleep apnoea), or<br>5. BMI= 30-35, with a normal or large waist circumference with comorbidity, or<br>6. BMI= 35-40, with a normal or large waist circumference with risk factors for cardiovascular disease or T2DM and without other comorbidities.<br>**Exclusion:**<br>1. Serious mobility limitations precluding participation;<br>2. Pregnancy.		88.8% Dutch	unit)	and intensive support from physical therapist.<br><br>12 months	with lifestyle advisor and dietitian as the intervention group, few numbers of meeting with physical therapist.<br><br>12 months	IPAQ, dietary habits:<br>Baseline<br>6 months<br>12 months<br>18 months<br>24 months<br><br>EQ-6D, healthcare costs:<br>Baseline, then every 3 months until 24 months	6. Direct and indirect costs;<br>7. Health risk, e.g. waist circumference, body composition, blood pressure, resting heart rate, blood biochemistry, and physical fitness.
**Biddle (2017)**<br><br>UK	Reduce sitting time.	**Inclusion:**<br>1. Age 18-40, BMI ≥30 (≥27.5 for South Asians).<br>2. Age 18-40, BMI ≥25 (≥23 for South Asians), with ≥1 additional risk factor for diabetes.<br>**Exclusion:**<br>Significant illness, steroid use, diabetes, pregnancy or an inability to communicate in English.	187<br><br>I: 94<br><br>C: 93	**Age:**<br>I: 32.4 (5.4)<br>C: 33.3 (5.8)<br><br>**Gender:**<br>68.5%<br><br>**Ethnicity:**<br>19.8% black and minority ethnic groups	Randomised controlled<br><br>Parallel groups<br><br>Community	STAND – A group-based structured education workshop.<br><br>6 weeks	Information leaflet focusing on T2DM, the importance of increasing physical activity and decreasing sedentary behaviour.	Baseline<br>3 months<br>12 months	1. Time spent in SB;<br>2. Number of breaks in SB (SB to upright movement) per day (Both by IPAQ and accelerometer);<br>3. Biochemical variables (glucose control, insulin sensitivity, cholesterol levels);<br>4. Anthropometric data (BP, weight, body composition, waist circumference);<br>5. Quality of life (EQ-5D);<br>6. Self-efficacy for SB change;

For peer review only

Supplementary file 3. Characteristics of 17 included RCTs_27.05.21

									7. Anxiety and depressions (HADS).
**Blunt (2018)**<br><br>Canada	Increase physical activity levels.	**Inclusion:**<br>1. Age 18-85;<br>2. ≥1 self-reported or measured risk factor for chronic disease including: BMI >25, <150 min of exercise/week, ≥3 hours sitting/day, <8 fruit and vegetable servings/day, diagnosis of metabolic syndrome or T2DM.<br>**Exclusion:**<br>Unable to comprehend the letter of information and consent documentation.	118<br><br>I: 59<br>C: 59	**Age:**<br>I: 56.8 (12.3)<br>C: 58.6 (14.7)<br><br>**Gender:**<br>78.8%<br><br>**Ethnicity:**<br>97.5% White	Randomised controlled<br><br>Parallel group<br><br>Primary care health centres	3-phases HealtheSteps™ program – in-person lifestyle coaching, and access to a suite of eHealth technology support.<br><br>18 months	Usual-care wait-list control to begin HealtheSteps™ 6 months after baseline.	Baseline<br>6 months (end of active phase intervention)<br><br>Additional for intervention group in minimally-support phase:<br>12 months<br>18 months	1. Mean daily steps (pedometer; self-report);<br>2. Time spent in PA; sitting (IPAQ);<br>3. Eating habits (STC; modified DINE);<br>4. Quality of life (EQ-5D; EQ-VAS);<br>5. Weight and body composition<br>6. Blood pressure;<br>7. Adverse events.
**Elramli (2017)**<br><br>UK	Increase average daily step count.	**Inclusion:**<br>1. Aged ≥18;<br>2. Confirmed diagnosis of Rheumatoid Arthritis (RA) according to ACR/EULAR 2010 criteria, within 5 years of diagnosis.<br>**Exclusion:**<br>1. Pregnant, severe hypertension, joint replacement within last 6 months, unstable cardiac conditions, or other serious pathology which would affect ability to take part in physical activity;	76<br><br>I: 39<br>C: 37	**Age:**<br>I: 58.2 (13.5)<br>C: 58.6 (15.8)<br><br>**Gender:**<br>83.9%<br><br>**Ethnicity:**<br>Not reported	Randomised controlled<br><br>Parallel groups<br><br>Community	Walk for Rheumatoid Arthritis (WARA) – 6 group sessions in first 7 weeks, 2 booster group sessions in week 14 and 28, personal support from physiotherapist on week 7, 9, and 11. Pedometers and PA diaries were given with instructions.	1 group education session on importance of exercise and healthy diet; and written educational material. At end of trial (12-month), provided pedometer and PA diaries, with	Baseline<br>13 weeks<br>26 weeks<br>52 weeks	1. Daily step count (accelerometer);<br>2. Time spent in SB (accelerometer);<br>3. Time spent in sitting; PA (IPAQ);<br>4. Disease activity (SDAI);<br>5. RA Quality of life (RAQoL);<br>6. Functional capacity (6MWT; MHAQ; hand grip test);<br>7. Cardiovascular risk factors (Blood biochemical variables; ASSIGN score Version

For peer review only

For peer review only - http://bmjopen.bmj.com/site/about/guidelines.xhtml

Supplementary file 3. Characteristics of 17 included RCTs_27.05.21

		2. Unable to understand written and spoken English or had cognitive impairment.				28 weeks	advice on use.		1.5.1; BMI; waist and hip circumferences);<br>8. Dietary assessment (DINE);<br>9. PA self-efficacy.
**Harris (2018)**<br><br>UK	Increase physical activity.	**Inclusion:**<br>1. Aged 45-75;<br>2. Registered at 1 of the 6 participating general practices;<br>3. Able to walk outside the home and with no contraindications to increasing their moderate intensity physical activity levels.<br>**Exclusion:**<br>1. Achieving at least 150 minutes of at least moderate intensity physical activity weekly;<br>2. Living in residential or nursing home, or housebound;<br>3. ≥3 falls, or ≥1 fall required attention, within last year;<br>4. Terminal illness, dementia, significant cognitive impairment, blind, new onset chest pain, MI, pregnant, conditions which GP judged for exclusion.	1,023<br><br>I:<br>Postal: 339<br>Nurse: 346<br><br>C: 338	**Age:**<br>45-54: 33.2%<br>55-64: 37.8%<br>65-75: 28.9%<br><br>**Gender:**<br>64.1%<br><br>**Ethnicity:**<br>80.3% White<br>10.3% Black<br>6.9% Asian<br>2.5% Other	Randomised controlled<br><br>Parallel groups by household<br><br>Community	1. Postal – pedometer, physical activity diary, and instructions for a 12-week walking programme sent by post.<br><br>2. Nurse support – provided pedometer, physical activity diary, and instructions by a practice nurse, who also provided 3 meetings over 3 months to facilitate participants to be more active.	Usual physical activity, provided a pedometer and guidance on a 12-week walking programme at end of trial.	Baseline<br>3 months<br>12 months	1. Daily step count (accelerometer);<br>2. Time spent in at least moderate PA (accelerometer);<br>3. Time spent in SB (accelerometer);<br>4. Self-reported PA (GPPAQ; IPAQ);<br>5. Cost-effectiveness to health services;<br>6. Exercise self-efficacy;<br>7. Anxiety, depression;<br>8. Quality of life (EQ-5D);<br>9. BMI; waist circumference; body fat;<br>10. Adverse events;<br>11. Health service use.
**Lakerveld (2012)**<br><br>The Netherlands	Improve lifestyle behaviour (dietary, physical activity,	**Inclusion:**<br>1. Aged 30-50;<br>2. Moderate or high risk of CVD (according to SCORE), or a high risk of T2DM (according to ARIC Study).	622<br><br>I: 314<br>C: 308	**Age:**<br>I: 43.6 (5.1)<br>C: 43.4 (5.5)<br><br>**Gender:**<br>58%	Randomised controlled<br><br>Parallel groups	Cognitive behavioural programme aimed at modifying dietary, and/or physical activity,	Provision of health brochures only	Baseline<br>6 months<br>12 months<br>24 months	1. Cardiovascular risk score;<br>2. Diabetes risk score;<br>3. Dietary behaviour (Food Frequency Questionnaire);

For peer review only

Supplementary file 3. Characteristics of 17 included RCTs_27.05.21

	and/or smoking).	**Exclusion:**<br>1. Having diabetes;<br>2. Previous CVD;<br>3. Pregnancy;<br>4. Current malignant disease;<br>5. (Severe) mobility problems.		**Ethnicity:**<br>Not reported	General Practices	and/or smoking behaviour, maximum of six individual counselling sessions of 30 minutes, followed by 3-monthly booster sessions by phone.<br><br>Intervention duration unclear			4. Time spent in PA and SB (SQUASH; a subscale of AQuAA);<br>5. Smoking behaviour;<br>6. Determinants of behavioural change;<br>7. Medical care utilisation;<br>8. BMI, waist-hip circumferences;<br>9. Cost-effectiveness and cost-utility in the societal perspective;<br>10. Quality of life (EQ-5D);<br>11. Blood pressure;<br>12. Blood biochemistry.
**Lane (2010)**<br><br>Ireland	To assess the impact of a community based, low-contact intervention on the physical activity habits of insufficiently active women.	**Inclusion:**<br>1. A population sample of women participating in a mass 10 km event;<br>2.Consented to follow-ups 2 and 6 months afterwards;<br>3. Those who had relapsed to insufficient levels of physical activity were invited.	176<br><br>I: 85<br>C: 91	**Age:**<br>21-49: 84%<br><br>**Gender:**<br>100%<br><br>**Ethnicity:**<br>Not reported	Randomised controlled<br><br>Parallel groups<br><br>Community	2 booklets delivered by post – Booklet 1 targeted the earliest stages of motivational readiness, and step-by-step guide to increase motivation. Booklet 2 targeted already motivated and active stage with information about moderate intensity PA, and staying active.	Placebo treatment – a healthy eating and nutrition booklet, delivered by post.	Baseline<br>6 weeks	1. Time spent in sitting;<br>2. Time spent in sufficient PA levels;<br>3. Time spent in total PA (All of above by bespoke self-report questionnaire);<br>4. Readiness to change (exercise motivational stage).
**Matson (2018)**<br><br>USA	To decrease sitting; increase standing	**Inclusion:**<br>1. Kaiser Permanente Washington (KPWA) members;	60<br><br>I: 29<br>C: 31	Age:<br>I: 69.0 (4.7)<br>C: 67.8 (5.2)	Randomised controlled	2 health coaching sessions; 4 follow-up health coaching phone	Healthy living intervention usually	Baseline<br>12 weeks	1. Time spent in sitting (total time, and number of periods of sitting for ≥30 minutes

For peer review only

Supplementary file 3. Characteristics of 17 included RCTs_27.05.21

	time and light physical activity.<br><br>(Pilot trial)	2. Age >60;<br>3. BMI 30–50 kg/m^2;<br>4. Not residing in long-term care or skilled nursing, no diagnosis of dementia, and no serious mental or a potentially terminal illness.<br>**Exclusion:**<br>1. Unable to stand, were not able to walk one block;<br>2. Participating in another intervention study;<br>3. Reported sitting time of less than 7 hours per day;<br>4. Could not communicate by phone, or speak and read English.		Gender:<br>68.3%<br><br>Ethnicity:<br>95.0% Not Hispanic or Latino<br>1.7% Hispanic or Latino<br>3.3% Unknown	Parallel groups<br><br>KPWA primary care clinics	calls; and written materials, and email reminders.<br>A wrist-worn device programmed to serve as an outward reminder strategy for taking breaks from sitting.<br><br>12 weeks	available to the KPWA members<br><br>12 weeks		continuously);<br>2. Daily number of sit-to-stand transitions (breaks from sitting) (Both of above by accelerometer);<br>3. Short Physical Performance Battery;<br>4. Blood pressure;<br>5. Fasting glucose level;<br>6. Total cholesterol level;<br>7. Depressive symptoms (PHQ-8);<br>8.Adverse events.
**Matthews (2016)**<br><br>UK	Increase walking, reduce sedentary behaviour.	**Inclusion:**<br>1. Aged 18-65;<br>2. Ambulatory and able to walk unaided for 10 minutes at a time, based on self/carer report;<br>3. Any level of intellectual disabilities;<br>4. Not currently taking part in any other research study.<br>**Exclusion:**<br>1. Wheelchair user or significant mobility problems;<br>2. Severe challenging behaviour, or other needs requiring constant one-to-one support from staff;<br>3. Involved in regular physical activity - meeting current public health recommendations for	102<br><br>I: 54<br>C: 48	**Age:**<br>I: 44.9 (13.5)<br>C: 47.7 (12.3)<br><br>**Gender:**<br>44.1%<br><br>**Ethnicity:**<br>Not reported	Cluster randomised controlled<br><br>Intellectual disabilities community-based organisations (cluster unit)	Walk Well programme – 3 face-to-face physical activity consultations, written resources for participants and carers, and an individualised, structured walking programme<br><br>12 weeks	12-week waiting list control	Baseline<br>12 weeks<br>24 weeks	1. Daily step count (accelerometer);<br>2. Time spent in SB; MVPA; total PA (accelerometer; IPAQ-S);<br>3. BMI; waist circumference;<br>4. Quality of life (EQ-5D; Subjective Vitality Scale);<br>5. Self-Efficacy for Activity for Persons with Intellectual Disability and Self-Efficacy for Exercise Scale.

For peer review only

Supplementary file 3. Characteristics of 17 included RCTs_27.05.21

		physical activity, for six months or more.							
**Poston (2013)**<br><br>UK	Behavioural intervention comprising dietary and physical activity changes to improve glycaemic control in obese pregnant women.<br><br>(Feasibility trial)	**Inclusion:**<br>1. Pregnant with booking BMI ≥30;<br>2. Singleton pregnancy, gestational age >15^{+0} weeks and <17^{+6} weeks' gestation.<br>**Exclusion:**<br>1. Gestation <15^{+0} weeks and >17^{+6} weeks;<br>2. Pre-existing diabetes;<br>3. Pre-existing essential hypertension (treated);<br>4. Pre-existing renal disease, multiple pregnancies, systemic lupus erythematosus (SLE), antiphospholipid syndrome, sickle cell disease; thalassemia; celiac disease, currently prescribed metformin; thyroid disease or current psychosis.	183<br><br>I: 94<br>C: 89	**Age:**<br>I: 30.4 (5.7)<br>C: 30.7 (4.9)<br><br>**Gender:**<br>100%<br><br>**Ethnicity:**<br>56.3% White<br>38.3% Black<br>1.6% Asian<br>3.8% Other	Randomised controlled<br><br>Parallel groups<br><br>Antenatal clinics	One-to-one appointment with the health trainer; weekly group sessions for 8 consecutive weeks from approximately 19 weeks' gestation; dietary advice, and physical activity level advice; plus usual antenatal care.<br><br>8 weeks	Usual antenatal care	Baseline (15^{+0} -18^{+6} weeks' gestation)<br>27^{+0} -28^{+6} weeks' gestation<br>34^{+0} -36^{+0} weeks' gestation	1. Attitudinal assessment questionnaire - perceived benefits and barriers and confidence to carry out the dietary and PA behaviours;<br>2. Quality of life (EQ-5D);<br>3. Edinburgh Post Natal Depression Score (EPDS);<br>4. Dietary assessment;<br>5. Time spent in SB; light PA; MVPA (accelerometer; RPAQ);<br>6. Maternal outcomes: diagnosis of GDM and pre-eclampsia, gestational weight gain, mode of delivery, blood loss at delivery, inpatient nights, detailed clinical and family history, health in current pregnancy, early pregnancy data (ultrasound scan, nuchal screening), blood pressure, routine blood results;<br>7. Neonatal outcomes: birthweight, anthropometry, inpatient nights.
**School of Public Health, HKU (2017)**	Healthier lifestyle by adopting Zero Time Exercise	**Inclusion:**<br>1. Aged ≥18 years;<br>2. Parents/grandparents with ≥1 child/grandchild aged 3–17;	728<br><br>I: 386<br>C:342	**Age:**<br>Majority aged 30-49<br>I: 87%<br>C: 84%	Cluster randomised controlled	Physical activity intervention – 4 group sessions over 12 months; biweekly/	Healthy eating intervention –similar structural	Baseline<br>3 months<br>6 months<br>12 months	1. Time spent in SB; PA (IPAQ-C);<br>2. Physical fitness performance (hand grip strength; time spent

For peer review only - http://bmjopen.bmj.com/site/about/guidelines.xhtml

Supplementary file 3. Characteristics of 17 included RCTs_27.05.21

Hong Kong	(ZTEx), and enhance positive family communication and personal and family wellbeing.	3. Primary education or higher; and able to read and write Chinese;<br>**Exclusion:**<br>Serious health conditions that might prevent from participating in low intensity physical activity.		**Gender:**<br>92.1%<br><br>**Ethnicity:**<br>Not reported	Integrated Family Service Centres (cluster unit)	monthly mobile messages to improve physical activity habit.<br><br>12 months	design as intervention group.<br><br>12 months		standing on 1 leg; foot pedalling duration);<br>3. Dietary habits;<br>4. Self-reported wellbeing (personal-health; happiness; family harmony).
**Spittaels (2007)**<br><br>Belgium	Increase physical activity.	**Inclusion:**<br>1. Aged 25-55;<br>2. No history of cardiovascular disease;<br>3. Internet access (including email access) either at home or at work.<br><br>**Exclusion:**<br>Not specified.	526<br><br>I:<br>Group 1: 174<br>Group 2: 175<br><br>C: 177	**Age:**<br>I:<br>Group 1: 39.7 (8.9)<br>Group 2: 39.3 (8.7)<br><br>C: 40.9 (8.0)<br><br>Gender: 30.6%<br><br>**Ethnicity:**<br>Not reported	Randomised controlled<br><br>Parallel groups<br><br>Internet	Group 1. Online-tailored physical activity advice + 8-week stage-based reinforcement emails.<br><br>Group 2. Online-tailored physical activity advice.<br><br>6 months	Online non-tailored standard physical activity advice – based on information present in the computer-tailored programme.	Baseline<br>6 months	1. Time spent in PA; SB (IPAQ).<br><br>In addition, in 1 of 6 worksites (n= 57):<br>2. Time spent in MVPA (accelerometer);<br>3. BMI; body fat; blood pressure; heart rate at rest.
**Stathi (2019)**<br><br>UK	Promote active ageing in socially disengaged, inactive older adults.<br><br>(Feasibility trial)	**Inclusion:**<br>1. Sedentary retired adults aged ≥65, reported spending <20 min per week in the past month in MVPA;<br>2. Capable of walking at least 200m.<br><br>**Exclusion:**<br>1. Disease or disability that seriously precluded participation in out-of-house activities, diagnosis of dementia;<br>2. Already meeting current PA recommendations, and	39 Participants:<br><br>I: 22<br>C: 17<br><br>(15 voluntary Activators)	**Age:**<br>I: 72.9 (7.3)<br>C: 75 (6.4)<br><br>**Gender:**<br>43.6%<br><br>**Ethnicity:**<br>97% White	Randomised controlled<br><br>Parallel groups<br><br>Community	ACE (Active, Connected, Engaged) intervention – One-to-one support from a peer volunteer (activator) to attend local activities continuously.<br><br>6 months	Waiting-list control group, and received written materials about local initiatives.	Baseline<br>6 months	1. Number of out of house activities;<br>2. Time spent in SB; lifestyle PA (accelerometer);<br>3. Lower limb function (SPPB);<br>4. Wellbeing (life-satisfaction; subjective wellbeing; resilience; and vitality);<br>5. Self-perceived barriers to activity in the neighbourhood.

For peer review only

Supplementary file 3. Characteristics of 17 included RCTs_27.05.21

		regularly engaging with local groups and Activities.							
**Williams 2019**<br><br>UK	Reduce sedentary behaviour, increase physical activity.<br><br>(Pilot study)	Inclusion:<br>1. A diagnosis of any serious mental illness;<br>2. Meeting any one of the following criteria: i) overweight, ii) at risk of or have diabetes, iii) in the clinician's view, have a sedentary lifestyle, iv) or smoke tobacco;<br>3. Ability to provide informed consent and understands English;<br>4. Aged ≥18 years.	40<br><br>I: 20<br>C: 20	Age:<br>I+C: 43 years (20–56)<br><br>Gender:<br>45%<br><br>Ethnicity:<br>50% Black<br>27.5% White<br>12.5% Mixed<br>7.5 Asian<br>2.5 Other	Randomised controlled<br><br>Parallel groups<br><br>3 community mental health teams	WTW intervention including an initial education session, fortnightly coaching, provision of pedometers and access to a weekly walking group.<br><br>17 weeks	Treatment as usual which consisted of care coordination plus written information on the benefits of increasing activity levels.	Baseline<br>17 weeks<br>6 months	1. Time spent in SB; light PA; MVPA (accelerometer);<br>2. Self-report SB and PA (IPAQ);<br>3. Motivation to engage in PA (BREQ-2);<br>4. Blood biochemistry;<br>5. Blood pressure;<br>6. BMI; waist circumference;<br>7. Mental Wellbeing (WEMWBS);<br>8. Functional mobility (TUG test).

Keys:
6MWT = 6-minute Walk Test; ACR/EULAR 2010 criteria = American College of Rheumatology/ European League Against Rheumatism 2010 criteria; ARIC = Atherosclerosis Risk in Communities; AQuAA = Activity Questionnaire for Adolescents & Adults; ASSIGN score = a cardiovascular risk score developed by Dundee University (2006); BCG = Behaviour Change Group; BMI = Body Mass Index; BP = blood pressure; BREQ-2 = Behavioural Regulation in Exercise Questionnaire-2; C = Control group; CVD = Cardiovascular disease; DINE = Dietary Instrument for Nutrition Education; EPDS = Edinburgh Post Natal Depression Score; EQ-5D/6D = European Quality of Life-5 dimensions/6 dimensions; EQ-VAS = European Quality of Life-Visual Analogue Scale; GI = glycaemic index; GP = General practitioner; GPPAQ = General Practice PA Questionnaire; HADS = Hospital Anxiety and Depression Scale; HCP = Health care provider; I = Intervention group; IDDM = insulin-dependent diabetes mellitus; IPAQ = International Physical Activity Questionnaire; IPAQ-C = International Physical Activities Questionnaire-Chinese version; IPAQ-S = International Physical Activity Questionnaire-Short version; KPWA = Kaiser Permanente Washington; MHAQ = Modified Stanford Health Assessment Questionnaire; MI = myocardial infarction; MVPA = Moderate to vigorous physical activity; n = Number of persons; PA = Physical activity; PHQ-8 = Patient Health Questionnaire; RA = Rheumatoid Arthritis; RAQoL = RA Quality of Life; RCT = Randomised Controlled Trial; RPAQ = Recent Physical Activity Questionnaire; SB = Sedentary behaviour; SCORE = Systematic Coronary Risk Evaluation; SD = standard deviation; SDAI = Simple disease activity index; SMART = Specific, Measurable, Achievable, Relevant and Time specific; SPPB = Short Physical Performance Battery; SQUASH = Short Questionnaire to Assess Health Enhancing Physical Activity; STC = Starting the Conversation questionnaire; T2DM = Type 2 Diabetes Mellitus; TEG = Traditional Exercise Group; TUG test = Timed Get Up and Go Test; WEMWBS = Warwick-Edinburgh Mental Wellbeing Scale; WHOQOL = World Health Organization Quality of Life; WTW = Walk this Way

For peer review only

Supplementary file 4.Characteristics of 17 included process evaluations_27.05.21

**Process Evaluation studies**				
**Study** *(Author (Year)*, **Country**	**Aims** *(whether process evaluation was pre-specified before commencing RCT)*	**Sample size and sampling method**	**Study Design (Data collection methods, e.g., mixed methods)**	**Frameworks for process evaluation**
**Adams (2012)**<br><br>USA	To explore overweight and obese women's perceptions of benefits, challenges and effectiveness of the intervention to reduce SB and increase PA. (Pre-specified)	I: n= 47<br>All participants in the intervention group were asked to complete the questionnaires at the mid-point of the intervention, and intervention end or withdrawing.<br><br>1 researcher<br>The researcher leading the PhD project.	Mixed methods:<br>1. By completing online questionnaires in different weeks during the intervention period, the participants evaluated their perceived benefits and barriers, frequency of using the intervention materials, and the effectiveness and ease of use of the intervention elements; and were asked to provide suggestions for improvement.<br>2. The researcher recorded her observations of the challenges, benefits, and costs in implementing the intervention.<br>3. Attendance and retention data were collected to determine attrition.	Not specified
**Albright (2015)**<br><br>USA	To quantify and compare the barriers to MVPA, frequency of achieving MVPA goals, and the relation of persistent barriers to achievement of goals. (Uncertain whether pre-specified or not)	I: n= 115<br>Study records of all participants in intervention group were used.<br><br>Staff conducted the telephone counselling sessions<br>Sessions were recorded, then selected for evaluation (Selection method and number of staff included were unclear – assuming random selection of the records).	1. Checklist to assess fidelity in 80 of the 1,586 recorded telephone counselling sessions.<br>2. Quantified information from telephone counselling sessions to evaluate goals set and achieved, and barriers.<br>3. Study records for assessing the use of intervention materials and attritions.	Not specified
**Benedetti (2020)**<br><br>Brazil	To conduct a comprehensive programme evaluation including all dimensions of RE-AIM using quantitative and qualitative data. (Uncertain whether pre-specified or not)	Participants in the programme<br>Sample size and sampling method not specified, assuming the BCG group only.<br><br>Staff<br>Professionals delivering the programmes, community health workers, and local and city administrators overseeing public health centers. Sample size and sampling method not specified.	Mixed methods:<br>1. 12 focus groups and 32 interviews with participants in the programme, staff delivering the intervention, or those overseeing the venues at the end of the trial.<br>2. Quantitative data in study records about participation, treatment effects, and fidelity.<br>3. Checklist for assessing implementation.	Framework: RE-AIM Framework (Glasgow et al., 1999)

For peer review only - http://bmjopen.bmj.com/site/about/guidelines.xhtml

Supplementary file 4.Characteristics of 17 included process evaluations_27.05.21

**Berendsen (2015)**<br><br>The Netherlands	To provide an insight into possible barriers and facilitators in execution and sustainability of lifestyle interventions in primary care. (Pre-specified)	I: n= 247, C: n= 164<br>All participants in intervention and control groups.<br><br>25 Health Care Providers<br>8 physiotherapists, 7 dietitians, 10 lifestyle advisors (who were practice nurses/ dietitian/ physiotherapists) were selected for the interviews (sampling method not specified).	Mixed methods:<br>1. Face-to-face, semi-structured interviews with HCPs at the end of the trial on fidelity, dose, context and strategy for implementation, and sustainability.<br>2. Questionnaires to participants every 3 months about dose and satisfaction.<br>3. HCP registries and logbooks completed during the trial about dose, fidelity, and attrition.	Frameworks: RE-AIM Framework (Glasgow et al., 1999); Steckler & Linnan (2002); Saunders et al. (2005); Grant et al. (2013)
**Biddle (2017)**<br><br>UK	To understand the trial outcome findings from the delivery of the workshop and participant behaviour change strategies. (Pre-specified)	I: n= 71 (then n= 45 at 6 weeks after the workshop; n=10 at 12 months)<br>All participants provided feedback immediately after the workshop, and were contacted at 6 weeks afterwards. Invitations sent to 28 participants at the end of the trial (12 months).<br><br>2 Educator/ Facilitator<br>All the workshop educator and facilitator were interviewed at the end of the trial.	Mixed methods:<br>1. Evaluation sheet completed by participants immediately after the educational workshop.<br>2. Phone interviews 6 weeks after the workshop.<br>3. Phone interviews at the end of the trial on following the intervention, awareness of risk, and suggestions for improvement.<br>4. Face-to-face interview with each workshop educator/ facilitator at the end of the trial on intervention delivery, anticipated effectiveness of the intervention, and suggestions for improvement.	Framework: MRC Guidance (Craig et al., 2008)
**Blunt (2018)**<br><br>Canada	To examine the acceptability of the intervention programme. (Pre-specified)	I: n= 13<br>All participants (n= 39) who attended the follow-up assessment at 12 months were invited to participate in an interview; 13/32 agreed participants purposefully chosen, according to baseline measures, e.g., average step count, and self-rated health.<br><br>12 Coaches<br>All coaches delivered the intervention, except 1 was unavailable due to scheduling conflicts.	1. Semi-structured interviews with coaches upon programme completion at 6 months, exploring experiences, barriers, and facilitators in delivering the intervention, and suggestions for improvement.<br>2. Semi-structured interview with participants at 12 months about the experience making health behaviour changes, programme successes and challenges, and suggestions for improving intervention.	Not specified
**Elramli (2017)**<br><br>UK	To explore participant views regarding the effectiveness of WARA intervention. (Pre-specified)	I: n= 10<br>Participants were chosen from the 3 recruiting hospitals, including both genders, who did and did not change PA level and step counts.	Semi-structured 30-minute phone interview at 6 months to explore participant's views about the effectiveness and overall views of the intervention.	Not specified
**Harris (2018)**	To examine the mechanisms of change by under-standing of	Nurse-supported group I: 295 completed by participants, 251 completed by nurses for participants	Mixed methods:<br>1. Semi-structured phone interviews with participants at the end of the trial, to explore their	Framework: MRC Guidance (Moore et al., 2015)

For peer review only - http://bmjopen.bmj.com/site/about/guidelines.xhtml

Supplementary file 4.Characteristics of 17 included process evaluations_27.05.21

UK	how the intervention was delivered and received, and how this may have affected the outcomes.<br>(Pre-specified)	All participants (n= 346) and nurses asked to complete the alliance questionnaires.<br><br>Nurse-supported group: n= 21, Postal group: n= 22<br>Semi-structured interviews: Participants consented at baseline, completed intervention at 12 months, selected according to step-count change, and baseline characteristics.<br><br>7 Nurses<br>All 8 nurses were invited to focus group/ interviews; 1 was unavailable and did not participate.	experiences.<br>2. Semi-structured focus groups/ interviews with nurses at the end of the trial to explore experiences of delivering PA consultations.<br>3. Patient alliance questionnaire and nurse alliance questionnaire on quality of delivery and participant responsiveness, covering different intervention aspects (e.g., working together and goal-setting, number of appointments).<br>4. Intervention session audio-records and checklists for fidelity and dose.<br>5. Return of participant's PA diary for participation, fidelity, and dose.<br>6. Trial administrative records about participation, dose, and fidelity.	
**Lakerveld (2012)**<br><br>The Netherlands	To describe the intervention's reach, effectiveness in terms of process outcomes, adoption, and implementation of intervention.<br>(Pre-specified)	I: n= 267<br>All participants (n =314) were asked to complete the questionnaire.<br><br>8 Practice nurses<br>All the nurses delivering the intervention.	1. Trial records for participations, dose, and treatment effects.<br>2. Questionnaires to participants at 6 months to evaluate satisfaction and effects on determinants of lifestyle behavioural change.<br>3. Questionnaires to nurses at 6 months to evaluate the training and their confidence in delivering the intervention.<br>4. 2 counselling sessions conducted by each nurse was tape-recorded to assess the nurse's competence.	Framework: RE-AIM Framework (Dzewaltowski et al., 2004)
**Lane (2010)**<br><br>Ireland	To explore the effectiveness and acceptability of intervention booklets.<br>(Aim is not specified, but assumed according to the reported results; and process evaluation is assumed to be pre-specified)	I: n= 85<br>Participants in the intervention group were contacted.	3 weeks and 6 weeks after baseline data were recorded:<br>Questionnaires were mailed or emailed to participants.	Not specified
**Matson (2018)**	Collecting qualitative results to further inform the feasibility	I: n= 22<br>The health coaches reported that 23 of all 29 participants were available, interested, or	Semi-structured exit interviews with participants within 10 days of the final follow-up, to explore their experiences and perceived health impact of the	Not specified

For peer review only - http://bmjopen.bmj.com/site/about/guidelines.xhtml

Supplementary file 4.Characteristics of 17 included process evaluations_27.05.21

USA	and acceptability of the interventions. (Pre-specified)	appropriate for the interview, thus the 23 participants were invited, but 1 participant declined.	intervention.	
**Matthews (2016)**<br><br>UK	To explore the feasibility of a 12-week walking intervention for adults with intellectual disabilities, in relation to context, recruitment and retention, reach, implementation and fidelity.<br>(Pre-specified)	<u>I: n= 20</u><br>Participants who had and did not have successful outcomes.<br><br><u>6 Key stakeholders</u><br>The health professional delivering the intervention; the researcher responsible for intervention delivery and management; 1 participant with positive study outcomes; 1 participant with no significant outcomes; 1 carer; a day centre manager	Mixed methods:<br>All conducted after the end of intervention:<br>1. Semi-structured interviews or focus groups with participants to explore their attitudes towards physical activity and walking, perceived benefits, drawbacks and impact of increased activity, subjective feelings of wellbeing, and any changes in view during the intervention period.<br>2. Interviews with key stakeholders to gain insight from a variety of individuals involved in the study.<br>3. Data input spreadsheet which recorded multiple elements including attendance, reasons for withdrawal from the study, for gaining insight regarding recruitment, retention and reach of the intervention.	Frameworks: MRC Guidance (Moore et al., 2015), WHO (2001); RE-AIM Framework (Glasgow et al., 2012); Steckler & Linnan (2002)
**Poston (2013)**<br><br>UK	To refine the intervention protocol through process evaluation of intervention fidelity.<br>(Pre-specified)	<u>I: n= 9, C: n= 12</u><br>Participants recruited from each study site, using a maximum diversity sampling approach, following an informed consent procedure.<br><br><u>130 audio diaries from Health trainers</u><br>Number of Health trainers completed included, or sampling method not specified.	Mixed methods:<br>All conducted after the end of intervention:<br>1. 17 face-to-face and 4 telephone semi-structured interviews with participants during their pregnancy, to capture their experiences and perceptions of the trial and intervention.<br>2. Audio diaries of health trainers in which they reflected on the fidelity and feasibility of the intervention delivery.<br>3. Study database for evaluating attendance.	Framework: Steckler & Linnan (2002)
**School of Public Health, HKU (2017)**<br><br>Hong Kong	To explore the opinions and experiences of the programme; to evaluate the effectiveness of the programme.<br>(Pre-specified)	<u>I: n= 24, C: n= 8</u><br>Participants who attended all the 4 sessions were invited.<br><br><u>8 Social workers and 1 Clerical staff</u><br>Sampling method not specified.	All conducted at the end of the trial:<br>1. Focus groups with participants to explore their experiences, and the impact of the intervention on their living habits and wellbeing.<br>2. Interviews with staff to collect comments about this study, and suggestions for future improvement.<br>3. Fidelity checks conducted for every session to ensure the quality and implementation of the intervention. Methods and results not reported.	Not specified.
**Spittaels (2007)**	To investigate the effectiveness of	<u>Tailored advice+emails group: n= 128,</u><br><u>Tailored advice group: n= 139, C: n= 156</u>	All completed at the end of intervention:<br>1. Questionnaire to all participants to investigate	Not specified

For peer review only - http://bmjopen.bmj.com/site/about/guidelines.xhtml

Supplementary file 4.Characteristics of 17 included process evaluations_27.05.21

Belgium	intervention outside laboratory.<br>(Uncertain whether pre-specified or not)	All participants were asked to complete the questionnaire; included participants were those responded.	whether participants remembered the advice, read the advice, and considered the advice had had a positive impact on their physical activity behaviour.<br>2. Further questions to the Tailored advice+emails intervention group to investigate the number of emails received and read, and their opinion on the provision of emails.	
**Stathi (2019)**<br><br>UK	To determine the relative usefulness of different intervention components, to identify ways to refine or improve the intervention.<br>(Pre-specified)	I: n= 20<br>Sampling method not specified.<br><br>13 Activators<br>Sampling method not specified.<br><br>2 Coordinators<br>Sampling method not specified.	Mixed methods:<br>All conducted at the end of intervention:<br>1. Quantitative process evaluation via a self-administered questionnaire which assessed changes in confidence to get out and about, social support, autonomy, competence, and relatedness.<br>2. 14 semi-structured exit interviews and 7 focus groups conduced with participants, activators and coordinators, to evaluate the effectiveness and suggestions of intervention elements.<br>3. Trial records for evaluating recruitment rate, attendance, completion rate, and acceptability of the intervention.	Framework: MRC Guidance (Moore et al., 2015)
**Williams 2019**<br><br>UK	To establish the feasibility and acceptability of the Walk this Way (WTW) intervention<br>(Pre-specified)	I: n= 5<br>Participants who agreed to be interviewed; sampling method unclear.	Mixed methods:<br>1. Semi-structured interviews to evaluate how participants experienced the intervention, and suggestions for improving the intervention.<br>2. Trial records for calculating recruitment rate, attendance, number of participants completed the intervention and refused outcomes measurements.	Not specified.

Keys: ACR/EULAR 2010 criteria = American College of Rheumatology/ European League Against Rheumatism 2010 criteria; ARIC = Atherosclerosis Risk in Communities; BCG = Behaviour Change Group; BMI = Body Mass Index; C = Control group; CVD = Cardiovascular disease; GP = General practitioner; HCP = Health care provider; I = Intervention group; IPAQ = International Physical Activity Questionnaire; MRC: Medical Research Council; MVPA = Moderate to vigorous physical activity; n = number of persons; PA = Physical activity; PhD = Doctor of Philosophy; RCT = Randomised controlled trial; SB = Sedentary behaviour; SCORE = Systematic Coronary Risk Evaluation; T2DM = Type 2 Diabetes Mellitus; sTEG = Traditional Exercise Group; WHO: World Health Organisation

References for process evaluation theoretical frameworks:

Dzewaltowski, D. A., Glasgow, R. E., Klesges, L. M., Estabrooks, P. A., & Brock, E. (2004). RE-AIM: Evidence-based standards and a web resource to improve translation of research into practice. Annals of Behavioral Medicine, 28(2), 75-80.

For peer review only - http://bmjopen.bmj.com/site/about/guidelines.xhtml

Supplementary file 4.Characteristics of 17 included process evaluations_27.05.21

Glasgow RE, Vogt TM, Boles SM. Evaluating the public health impact of health promotion interventions: the RE-AIM framework. Am J Public Health. 1999;89(9):1322–7.

Glasgow R, Boles S, Vogt T: Reach Effectiveness Adoption Implementation Maintenance (RE-AIM). [19-Feb-2012]; 2012. www.re-aim.org.

Grant A, Treweek S, Dreischulte T, Foy R, Guthrie B. Process evaluations for cluster-randomised trials of complex interventions: a proposed framework for design and reporting. Trials. 2013;14:15.

Moore GF, Audrey S, Baerker M, Bond L, Bonell C, Hardeman W, et al. Process evaluation of complex interventions: Medical Research Council guidance. BMJ. 2015;350:h1258.

Saunders RP, Evans MH, Joshi P. Developing a process-evaluation plan for assessing health promotion program implementation: a how-to guide. Health Promot Pract. 2005;6(2):134–47.

Steckler A, Linnan L. Process Evaluation for Public Health Interventions and Research. San Francisco: Jossey-Bass; 2002.

World Health Organisation [WHO]. Process Evaluation Workbook. Geneva: WHO; 2001.

For peer review only

Supplementary file 5. Delivery and mechanisms_27.05.21

Study (Year)	Intended delivery (aim/ intervention description)	Actual delivery (difference from the intended delivery)	Intended mechanism (theoretical model/ logic model)
**Adams (2012)**	**On Our Feet intervention** – combination of 2 face-to-face interactive group sessions, and 6 weekly email messages. 1-2 Weeks were led in-person by the researcher. 3-6 Weeks were conducted over the internet, mainly by email.<br><br>Participants were given feedback on their initial levels of SB and PA, were led through a goal setting activity and provided with self-monitoring tools, e.g., Actigraph activity monitor. Positively-framed email messages that contained peer-modelled alternatives to sitting and additional behavioural feedback were sent weekly.<br><br>**Control group** – waitlist control.	(Adaptations)<br>1. Due to schedule conflict for 1 chapter, the initial presentation and the goal setting activity took place at the same meeting instead of respective weeks. Participants received extra email and phone contact to answer any questions during the second week.<br>2. While the same visual aids were used in the initial presentation in each chapter, the depth of explanation for each chapter varied according to the participants' questions.<br>3. Proposed group activity on emotions regarding sitting and some segments of the presentation were reduced or removed because of the time limit for the sessions.<br>4. Software problems causing inaccurate estimates of SB provided to some participants.	The intervention focused on improving self-efficacy in the Social Cognitive Theory, by addressing 4 self-efficacy construct – mastery experiences, modelling, verbal and social persuasion, and emotional and physiological states. It combined the various stages of changes in the Transtheoretical Model, to reduce SB and increase PA.<br><br>In the group sessions, video and demonstrations modelled the intervention exercises. Participants set goals and rated their confidence in achieving the goal, which was intended to increase recognition of self-efficacy. The self-monitoring tools assisted the re-evaluation of SB. Tailored feedback on behaviour change facilitated mastery experiences. Group discussions, uses of behavioural cues, and positively-framed emails encouraged and prompted continuous behaviour changes.
**Albright (2015)**	**TTCW intervention** – telephone counselling sessions and a website, tailored to address a woman's specific MVPA benefits and barriers over a 12-month intervention.<br>*17 Telephone counselling:*<br>The health educator discussed MVPA goals, anticipated barriers and resolutions with participants; tracked MVPA goals (type of activity, duration, and intensity); and provided tailored suggestions on the TTCW website, by email, or mail.<br>*Schedule of counselling calls:*<br>Phase 1: weekly calls (for month 1); Phase 2: biweekly calls (2 Months and 3 Months); and Phase 3: monthly calls (4 Months to 12	(Adaptations)<br>1. In TTCW group, only 75% of participants set incremental MVPA goals with a health educator during the intervention period.<br><br>2. Some initial PA goals were set at light intensity, because the participants were relatively inactive at the beginning of the intervention.	The tailored TTCW intervention aimed to positively alter the key mediators of PA – personal, social, and environmental factors, to enhance self-efficacy and reduce barriers, using the Social Cognitive theory and Transtheoretical Model theory.<br><br>Health educators provided counselling calls, using Motivational interviewing, to encourage goals settings, problem-solving, self-monitoring, and self-reinforcement, to integrate PA into daily lives; while preparing the participants to prepare and progress through the stages of change.

For peer review only

Supplementary file 5. Delivery and mechanisms_27.05.21

	Months).<br>*TTCW website:*<br>Contained various resources designed to facilitate MVPA, e.g. behaviour-change tip, calendar listing "baby-friendly" exercise sessions in the community, and newsletters. Participants were informed that the website would be updated 2-3 times per month.<br><br>**SWO (control group)** – "standard" PA information was available on the SWO website, e.g., information about how to become more physically active via links to credible sources (i.e., American Heart Association, etc.). Participants in this group did not receive any telephone calls or goal-setting advice about MVPA.		The TTCW website provided information about supportive environments for the participants to exercise; and suggestions about obtaining social support for PA.
**Benedetti (2020)**	Reported as actually delivered interventions.	**BCG –** the behavioural change programme that was adapted from "Active Living Every Day" (ALED), delivered by specifically trained nutrition and exercise science professionals working at the HCs. The sessions included a series of topics related to behaviour change, aiming at a more active lifestyle.<br><br>**TEG** - received a 12-week exercise class conducted at the local HCs, led by exercise professionals employed by the HCs; 3 times per week for 60 minutes. Each session included warm-up, aerobic exercise at 50–80% of maximum aerobic power, resistance training, and cool-down. Participants' heart rate and ratings of perceived effort were tracked during each session.	The BCG was adapted from "Active Living Every Day," or ALED, from the USA (Bors 2009).<br><br>A series of behaviour change topics were delivered through 12 structured weekly meetings, aiming to achieve a more active lifestyle. The topics included finding new opportunities to be active, overcoming challenges, setting goals and rewarding, gaining confidence, enlisting support, avoiding pitfalls, step by step, positive planning, making lasting changes.
**Berendsen (2015)**	(Protocol)<br>**Supervised programme:** 6-7 individual meetings, and 26–34 group meetings with PT.	(Differences)<br>1. In both programmes the number of meetings with all HCPs was lower than planned in the protocol. Participants of the Supervised	Beweegkuur provided a wide-ranging lifestyle counselling by means of Motivational Interviewing and incorporating the concepts from Self-Determination Theory.

For peer review only

Supplementary file 5. Delivery and mechanisms_27.05.21

	**Start-up programme (Control):** 6 individual meetings with PT.<br><br>**Both programmes** comprised 6 individual coaching meetings LSA, 3 individual meetings with a dietitian, and 7 dietary group meetings, for 1 year.<br>The initial individual meetings with the HCPs were to set personal (exercise and nutritional) goals, and identify barriers to a healthy lifestyle through motivational interviewing, which were the basis for meetings. At the end of the programme, each participant met with the LSA to evaluate the lifestyle changes and conclude the intervention.	programme attended, compared to participants of the Start-up programme, more meetings with physiotherapists, but fewer with lifestyle advisors and dietitians.<br>2. No PT group meetings were planned in the protocol for the control Start-up group, but some PTs organised over 9 meetings. Some PT of the start-up programme only planned group meetings, instead of the intended individual meetings with each participant.<br>3. For both groups, 3 individual meetings with the dietitians were planned in the protocol, but the Start-up group received a median of 4 meetings (7 meetings at 75th percentile). On the other hand, some participants did not prefer individual meetings which added fees to participants.<br>4. Some dietitians did not plan individual meetings, and therefore felt there was no opportunity to set individual goals.<br>5. Not all participants reported that they set goals with the PA and dietitian; nor the LSA had explicitly concluded the intervention.<br>6. Not all HCPs were trained in Motivational Interviewing techniques.	All HCPs addressed goals and barriers in the different aspects of lifestyle, to promote participant's motivation for behaviour change, problem-solving skills, and thus promoting participant's sustainable self-efficacy and environment to engage in long-term PA and healthy dietary behaviour.<br><br>It has been hypothesised that the additional amount of guidance within the Supervised programme provided additional contacts and guidance, as a hypothesis that the increase in effects on physical activity would lead to bigger treatment effects.
**Biddle (2017)**	(Protocol)<br>A comprehensive health assessment, including blood tests, was conducted at the trial baseline clinic. Results were sent to all participants (intervention and control groups) and discussed in the educational workshops with each participant.<br><br>**STAND Intervention** – A 3-hour group-based educational workshop, based on the DESMOND and PREPARE structured education protocols, delivered by trained educators; plus a motivational follow-up phone call (6 Weeks) to	Delivered as intended.	STAND intervention started with a letter sent to participants at risk of T2DM and an invitation for risk tests, then discussing with an educator about the risk information and amount of SB time, by using the Commonsense Model of Illness.<br><br>The workshop was based on Commonsense Model and Dual Process Theory, in which the trained educators provided information on risk factors and complications relating to T2DM. Participants were encouraged to assess their own health risk, and to identify their modifiable

For peer review only - http://bmjopen.bmj.com/site/about/guidelines.xhtml

Supplementary file 5. Delivery and mechanisms_27.05.21

	review and support participants' behaviour change progress. The 'Gruve' (MUVE, Inc., USA: www.muveinc.com) was provided to participants, for self-monitoring on time spent sedentary and in PA, and prompting for break from prolonged times of inactivity. Text messages were sent to participants to encourage adherence to goals and use of the Gruve.<br><br>**Control group** – received an information leaflet focusing on key illness perceptions of being at risk of T2DM, the importance of increasing physical activity and decreasing sedentary behaviour.		risks.<br><br>Social Cognitive Theory and Behavioural Choice Theory were also employed in the workshop content, to aid participants identifying health risks associated with excess SB, strategies to reduce SB in their daily life, identifying barriers, and setting goals and action plans.<br><br>The self-monitoring tool, the Gruve, was provided to facilitate self-regulation of SB.
**Blunt (2018)**	(Protocol)<br>**The HealtheSteps™ programme** – provided individuals with a specific plan of action to improve their PA levels, healthy eating habits, and reduce sedentary behaviour.<br>*Active phase (0-6 Months):*<br>1. bi-monthly in-person coaching to set prescriptions for physical activity, exercise, and healthy eating; provided by 1 trained HealtheSteps™ coach throughout this phase.<br>2. Access to a Tyze Personal Networks (an online social network to connect with coaches and other participants); phone coaching supports; and a free HealtheSteps™ smartphone app (providing virtual coach, heart rate monitor, step counter, and tracking option to monitor progress).<br>*Maintenance phase I (7-12 Months):* in-person coaching removed, but participants had access to the full suite of eHealth technology supports.<br>*Maintenance phase II (13-18 Months):* access to the full suite of eHealth technology supports removed, and participants only had access to	(Adaptations)<br>The central research team scheduled coaching sessions for some coaches, resulting that some participants had different coaches at each session.	HealtheSteps™ was based on the Social Cognitive theory of self-regulation. The mobile app, online tools and resources, and initial supports from the coaches facilitated positive health behaviour changes and self-management of own risk factors for chronic disease.<br><br>Individualised lifestyle prescriptions were given to participants in the initial phase, using Motivational Interviewing and SMART goal setting principles (specific, measurable, attainable, realistic, and timely for the participant). These aimed to produce positive behaviour change and overcome potential barriers.

Supplementary file 5. Delivery and mechanisms_27.05.21

	publicly available resources and tools.<br><br>**Comparator group (waitlist control)** – This group continued with usual activities without intervention from the study team for the first 6-month period. After the 6 Months follow-up measurements, participants were given the opportunity to start the 6-month HealtheSteps™ programme.		
**Elramli (2017)**	Reported as actually delivered interventions.	**The WARA intervention consisted of 2 components** – *PA component:* a pedometer supported walking programme, aiming to increase participant's average daily step count by 3000 steps above their baseline value, on at least 5 days of the week by 6 months, and to maintain for up to 12 months; and to comply with the UK physical activity guidelines (2011) recommended of a total of 150 minutes per week.<br>*Educational component:* 6 weekly interactive group (up to 6 persons) sessions, each lasted 1 hour; and two booster sessions (at 3 and 6 Months) providing support to participants to evaluate their PA levels and barriers.<br>A WARA booklet was provided to participants, describing the importance of walking, strengthening exercise, reducing SB, and a healthy diet for health benefits.<br><br>**Control group –** 1-hour single education group session (up to 6 persons), included topic regarding the importance of physical activity and healthy diet.	The WARA programme was based on the Social Cognitive Theory, focusing on self-efficacy; and incorporated behaviour change techniques, particularly self-monitoring, feedback, and social support.<br><br>The group education sessions aimed to provide social support; increase the participant's awareness and knowledge of their condition, and encourage PA increase. Therefore, the participant's self-efficacy increase.<br><br>Setting goal of step-count, using pedometer and PA diary, facilitated self-monitoring with feedback from the pedometer, thus increased individual motivation to achieve behaviour change.<br><br>The WARA booklet provided health information which further increased the participant's knowledge and awareness (self-efficacy) of self-management and PA for RA.
**Harris (2018)**	(Protocol)<br>**Pedometer-plus-nurse-support group** – Pedometer and written instructions for a 12-week walking intervention, based on the participant's usual step-count provided. In addition, 3 PA consultations with a practice	(Adaptations)<br>1. Nurses and participants adapted and tailored step count target to individual circumstances, e.g., adjustments were made to the intervention to accommodate religious observances, such as Ramadan and Christmas;	The intervention resources used behaviour change techniques (BCTs).<br><br>3 PA consultations with the practice nurse were divided into 3 stages – First steps, Continuing the changes, and Building lasting habits. They

Supplementary file 5. Delivery and mechanisms_27.05.21

	nurse, individually or as a couple.<br><br>**Pedometer-alone group –** a pedometer, and a 12-week pedometer-based walking programme, posted to the participants. The programme was based on the participant's baseline step-count. On study completion (1 year from baseline), participants in this group were offered a single practice nurse PA consultation.<br><br>**Control group** – No PA intervention. They were offered to choose either receiving a pedometer and the written 12-week pedometer-based walking programme, by post, or as part of a single practice nurse consultation.	during illness; and changes in weather.<br>2. Nurses adapted participant's preferences for interventional materials when tailoring advice, e.g., counting walking by time instead of step-count; whether to use the optional handouts or not.<br>3. Not all participants altered their walking targets; some might have decreased PA level as the target.	included motivational interviewing, health information about PA, suggestions to increase PA, action planning, goal setting, self-monitoring, relapse prevention, which aimed to effect positive changes in participant's step count, PA and SB times; thus longer-term changes in walking habits and health benefits.<br><br>The patient handbook provided the same information as in the nurse consultations.<br><br>Step count diary provided suggestions and instruction for the 12 weeks walking programme. Participants could set goals, self-monitor with feedback from pedometer to increase step count.
**Lakerveld (2012)**	(Protocol)<br>**Intervention group** – Each participant was free to choose the own target lifestyle component(s) (smoking, physical activity or diet). Nurse practitioner provided the CBP to increase participant's motivation and ability to change their dietary pattern, physical activity or smoking behaviour, maximum of 6 individual 30-minute counselling sessions (weekly then reduced to every 2-3 weeks, for 2-4 months); then 3-monthly telephone booster sessions for 12 months. The total intervention period, including booster calls, will be 16 months. The MI and PST counselling methods were used.<br><br>**Control group** – Received written information about their risk of developing T2DM and CVD, and brochures of health guidelines regarding physical activity, healthy diet, and smoking cessation.	(Adaptations)<br>Actual intervention duration is unclear: The number of sessions and schedule described in the results report (Lakerveld et al., 2013) matched the protocol; but the report stated the intervention generally lasted up to 6 months.	The cognitive behavioural programme (CBP) applied the Theory of Planned Behaviour (TPB) and the theory of self-regulation, with 2 counselling techniques - Motivational interviewing (MI), and problem-solving treatment (PST).<br><br>A nurse practitioner used MI to explore the participant's attitude and intention to make lifestyle behaviour change, then resolve the ambivalence between the goal and the actual situation. Afterwards, the nurse practitioner used PST to prompt the participant to find solutions for barriers and reinforcing perceived control for behaviour change. When setting new goals was needed, the same process would be started again.<br><br>The nurse practitioner guided the participant to gradually increase the sense of mastery over difficulties and be more active in planning and

For peer review only

Supplementary file 5. Delivery and mechanisms_27.05.21

			implementing activities.
**Lane (2010)**	Reported as actually delivered interventions.	**Intervention group** – Participants answered a question about the stages of change at baseline. The answer determined either both intervention booklets or just one of them to be posted. The booklets provided information on physical activities and motivation to change, tailored to the participant's readiness to change.<br><br>**Control group** – Received a healthy eating and nutrition booklet, developed by the Irish Heart Foundation, An Bord Bia and the Health Promotion Unit, by post, as placebo treatment.	The tailored intervention applied the trans-theoretical model (TTM), which posits that individuals move through stages of change while learning and adopting new behaviours.<br><br>The intervention consisted of two print booklets, specific to the initial and later stages of motivational readiness. The booklets were adapted for Irish women to promote physical activity, which were broadly based on the TTM model.<br><br>The booklets contained information and structured approaches and strategies, designed to alter self-efficacy, social support, outcome expectancy and barriers to physical activity, tailored to the individual's readiness to change and may subsequently modify physical activity behaviour.
**Matson (2018)**	(Protocol)<br>**STAND intervention** – consisted of 6 health coaching sessions provided by a trained Health Coach, an educational information workbook, SB feedback charts, and a Jawbone UP band.<br>*6 health coaching Sessions:* 2 in-person sessions (first 2 weeks, 45-60 minutes each), providing and explaining the workbook, feedback chart, and Jawbone UP wristband to participants; discussing tailored reminder strategies and setting goals and action plan.<br>After that, *4 bi-weekly phone calls:* (20-40 minutes each) from the Health Coach, to review progress on goals and action plans, problem-solve barriers, use the workbook to guide participants on different types of reminder.<br><br>Based on data from participant's activPAL wear	Delivered as intended.	I-STAND intervention was based on behavioural theories, including social cognitive theory, the ecological model, and habit formation theory.<br><br>Health coaching sessions focused on using different types of reminders, building self-efficacy through motivational interviewing, problem-solving barriers, and setting personalised action plan and graded goals. (Social cognitive theory, habit formation theory)<br>The workbook and coaching sessions included social support, social environment and norms, evaluating participant's environment, to consider the possible changes. (Ecological model).<br>The wrist-worn Jawbone UP band device vibrated every 15 minutes of inactivity. This served as an outward reminder strategy for

For peer review only

Supplementary file 5. Delivery and mechanisms_27.05.21

	at baseline, SB feedback charts 1 Week, and 6 Week were provided to participants.<br><br>**Healthy Living Control group** – 1 in-person health coaching session: Participants were provided a health education workbook containing topics about ageing and instructed to work on 1 topic every 2 weeks using a goal-setting worksheet.<br><br>Every 2 weeks, participants received a check-in letter and asked to complete and return a review progress form.		disrupting the habitual SB, to promote behaviour change and new habits of taking breaks from sitting (habit formation theory).
**Matthews (2016)**	(Protocol)<br>**Walk Well intervention –** 12-week community-based walking programme, consisted of 3 physical activity consultations with a walking advisor; aimed to increase walking by 30-minutes on at least 5 days per week. Participants were provided with education booklets, a pedometer and step diary.<br><br>**Waiting list control group** – were advised to continue with their daily activity for 12-weeks, following which they were invited to participate in the Walk Well intervention.	(Adaptations)<br>1. Some participants experienced difficulty in reading the pedometer and recording step counts in the diary, thus adapted the diary to an alternative "tick box" to indicate having walk(s).<br>2. The physical activity consultations were refined and streamlined to focus on the core components, and flexible options of additional behaviour change techniques for adults with intellectual disability.<br>3. Walking groups were not planned, but expected by some participants, thus arranged by the care centres and carers.	Walk Well was based on the Social Cognitive theory and Trans-theoretical Model.<br><br>The PA consultations method focused on 4 core behaviour change techniques: goal setting; self-monitoring; developing self-efficacy; and mobilising social support. Furthermore, the walking advisor tailored the use of additional behaviour change techniques according to the participant's needs. The aim was autonomy and motivation of the participants to lead a more active lifestyle.<br><br>Input and engagement from carers provided social support from them directly, and their arrangement for group walks among participants.<br><br>The education booklets with visual images and appropriate text provided and reinforce health information.<br><br>Pedometer and step diary complemented the PA consultation, to motivate the participant to set goals and self-monitor step count.

For peer review only

Supplementary file 5. Delivery and mechanisms_27.05.21

**Poston (2013)**	Reported as actually delivered interventions.	Participants were recruited in early 2nd trimester (>15^{+0} weeks to <17^{+6} weeks' gestation) to allow adequate time for the intervention programme that was planned to end at each participant's 27^{+0} and 28^{+6} weeks' gestation.<br><br>All women attended routine antenatal care appointments and received advice regarding diet and physical activity (PA) in accordance with local policies, which draw on UK NICE guidelines.<br><br>**Intervention group –** participants attended a one-to-one appointment with the HT, provided with a pedometer, a logbook for setting goals and self-monitoring, and a DVD of exercise regime for pregnancy. After that, 8 weekly group sessions from approximately 19 weeks' gestation. The programme included dietary advice choosing low GI food and reducing saturated fats, and increasing daily PA level during pregnancy safely.<br><br>**Control group** – standard care, with additional appointments with the study midwife at 27+0 - 28+6 and 34+0-36+6 weeks', where possible coinciding with routine antenatal visits.	The intervention was based on the Control Theory, and Social Cognitive theory.<br><br>Participants were provided with a pedometer, logbook, an exercise DVD, to set, self-monitor, and achieve SMART (Specific, Measurable, Achievable, Relevant, and Time Specific) goals for diet and PA, using self-regulation techniques from the Control Theory.<br><br>The group sessions facilitated self-identification of benefits and barriers to behaviour change, which facilitated self-efficacy, and provided social support.
**SPH HKU (2017)**	Reported as actually delivered interventions.	**PA group** – received 4 group sessions: 2.5-hour interactive knowledge and motivation enhancement core session at baseline, a 1.5-hour experience sharing booster session at 3 Months, 2.5-hour tea gathering family session at 6 Months, and a Holistic Health session at 1 Year. 16 monthly/bi-weekly health-related text messages to mobile phone for knowledge enhancement and as reminders till one year after baseline.<br><br>**Control group** – received the same intervention framework and methods and the same number and duration of sessions, about Healthy diet.	The PA group intervention was guided by the Health Action Process Approach (HAPA), which proposes motivation, goal setting and planning enhance intention, thus promote its conversion to action. The intervention aimed to enhance knowledge, self-efficacy, and motivation in relation to practising ZTEx<br><br>The conceptual framework proposed that the participants pass the intervention information positively and encourage their family to practise the actions together. Through these family actions and communication, the wellbeing and

For peer review only

Supplementary file 5. Delivery and mechanisms_27.05.21

		Fidelity evaluated but not reported.	harmony of the family were enhanced.<br><br>The strategies included:<br>1. Introducing information on the consequences of physical inactivity, obesity and ZTEx (risk perception);<br>2. Enhancing skills and confidence in the ability to do ZTEx (exercise self-efficacy);<br>3. Associating the health behaviour to the positive outcomes of the trainees (outcome expectations); and<br>4. Introducing cognitive dissonance, i.e., a discrepancy between participants' belief (including a pledge to eat) and behaviour (failure or potential failure to act) to promote intrinsic motivation to change behaviours.<br><br>The mechanism of changes for the Healthy diet intervention (control) was the same, but focusing on healthy diet only.
**Spittaels (2007)**	Reported as actually delivered interventions.	**Tailored information and reinforcement emails group:**<br>*Tailored advice:* Participants completed a questionnaire about their PA and psychosocial determinants on the study's intervention website; subsequently, the tailored advice containing normative PA feedback and suggestions to increase PA levels were produced from it. Participants having intentions to increase PA levels were encouraged to make an action plan.<br><br>*Emails:* After receiving the first tailored advice, participants received regular emails (5 emails in 8 weeks), which asked participants to identify their current stages of change, then referred to a corresponding website with personalised information to encourage behaviour changes.	According to each individual's stage of changes, the tailored advice was provided to participants based in Transtheoretical model. The content applied the constructs of Theory of Planned Behaviour, i.e., intentions, attitudes, self-efficacy, social support, knowledge, benefits and barriers to physical activity.<br><br>Participants indicated with positive intentions to increase their PA levels in the online questionnaire were then encouraged by the website to make a personal action plan to implement behaviour changes.<br><br>Reinforcement emails assessed and followed the participant's stage of change, then directed the participant to pertinent online advice to further encourage behaviour changes.

For peer review only

Supplementary file 5. Delivery and mechanisms_27.05.21

		**Tailored information group:** Participants received the tailored advice online but did not receive reinforcement emails.<br><br>**Standard advice (Control):**<br>Participants received standard physical activity advice from a website, based on information presented to the other 2 groups, but not individually-tailored, e.g., the benefits of PA, current public health recommendations, the difference intensity PAs, and suggestions to be more physically active.	
**Stathi (2019)**	Reported as actually delivered interventions.	Activators attended a 2-day training course, and received an intervention delivery manual. They were trained on the protocol for types and frequency of interactions with the participants; also encouraged to be flexible according to individual needs.<br><br>Each participant was invited to attend a 6-month programme:<br>*Motivation stage (first 2 weeks)* – 2 one-to-one meetings with an activator to support motivation, build rapport, review local activities, and consider and address any barriers to participation.<br>*Action stage (1-3 Months)* – ≥3 visits to local initiatives with the activator.<br>*Maintenance stage (3-6 Months*) – Support provided by telephone, and ≥2 further visits with the activator to encourage the participant to attend local activities independently.<br>Participants could engage in a wide range of activities at the Action and Maintenance stage, e.g., bowling, ballroom dancing, lunch clubs, walking groups, and art classes. 2 social events were organised for all participants and activators to facilitate within group support and encourage more local engagement.	Intended processes of behaviour change during the three stages of the ACE intervention followed the principles of Self Determination Theory, to facilitate the participant's developing autonomous motivation, confidence, and competence for getting out and about.<br><br>In the Motivation stage, the participant engaged in social support from the activator, understood the process, and explored and enhanced motivation for actions. In Action stage, the participant made plans with the activator to try out interested activities and monitored progress. In Maintenance stage, the participant was encouraged to continue with the activities more independently, while the support from the activator was reduced.<br><br>It was shypothesised that participants in the ACE intervention would attend more out-of-house activities, and better motivation to lead an active lifestyle in the long term.

For peer review only

Supplementary file 5. Delivery and mechanisms_27.05.21

		The control group received written materials about local initiatives only, but were offered the intervention at the end of study period.	
**Williams (2019)**	(Protocol)<br>**Walk This Way intervention** – amended from the Walk, Address sensations, Learn about exercise, encourage exercise behaviour for persons with schizophrenia spectrum disorders ('WALC-S') programme<br>*Initial group education session:* 5-10 participants; participants were provided a pedometer for self-monitoring and calendar for recording; setting goals for increasing habitual walking level.<br>*Continuing support and coaching:* every 2 weeks (20-30 minutes), an assigned coach met the participant to review the participant's walking calendar, identify and address barriers and facilitators to increase PA and decrease SB, and provide motivational support to the participant to reach.<br>Weekly walking group: the coaches arranged and invited all participants to an optional weekly group walk (2 hours).<br><br>**Control condition** – Received written information on the benefits of increasing activity levels. This advice was given in accordance with the NHS Foundation Trust policy on physical health.	Delivered as intended.	The Walk this Way intervention employed the COM-B model of behaviour change principles to address capability, opportunity, and motivational barriers to reducing SB and increasing PA.<br><br>The Initial education session aimed to enhance motivation and self-efficacy to make behaviour change.<br><br>Health coaching sessions used the REACH© model of coaching, emphasising individual's accountability involves thinking, feeling, and doing to achieve the self-identified goals. Health information of PA, support and motivation for goal attainment were provided to facilitate the participant to increase walking into daily routine independently.<br><br>The participant's walking goal was set with SMART (Specific, Measurable, Attainable, Realistic and Timely), self-monitored by pedometer and calendar; the step count and factors affecting attainment were discussed with the coach.<br><br>Weekly regular group walk was optional, which provided social support to the participants.

Keys: * = Data from associated publications; ACE = Active, Connected, Engaged intervention; BCG = Behaviour change group; BMI = Body Mass Index; C = Control group; CBP = Cognitive behavioural programme CVD = Cardiovascular disease; DESMAND = Diabetes education and self management for ongoing and newly diagnosed; DM = Diabetes Mellitus; FU = Follow-up; GI = Glycaemic Index; GP = General practitioner; HC = Health centre; HCP = Health care provider; HT = Health trainer; I = Intervention group; LSA = Lifestyle advisor; MVPA = Moderate-to-vigorous physical activity; PA = Physical activity; PREPARE = Prediabetes risk education and physical activity recommendation and encouragement; PT = Physiotherapist; SD = Standard deviation; SMART = Specific, Measurable, Achievable,

For peer review only

Supplementary file 5. Delivery and mechanisms_27.05.21

Relevant, and Time Specific; STAND = Sedentary Time ANd Diabetes; SWO = Standard website-only; TEG = Traditional exercise group; TTCW = Tailored telephone counselling plus website; WARA = Walk for Rheumatoid Arthritis; ZTEx = Zero Time Exercise

For peer review only

Supplementary file 6. Implementation data_27.05.21

Study (Year)	Fidelity (delivering the intervention as per protocol)	Recruitment (recruiting participants and sites)	Retention (participants remaining in the intervention or control/usual care group)	Reach (dose received and participant engagement)
**Adams (2012)**	Only qualitative data reported.	10 clusters invited.<br>7 clusters recruited (needed active membership n≥12).	I: n= 40 (85.1%)<br>C: n= 24 (85.7%)<br><br>Primary reasons for leaving the study:<br>55% (6/11) Having to wear the activity monitors.<br>18% (2/11) Time commitment too great.<br>18% (2/11) Had not understood length of study.<br>9% (1/11) Went out of town unexpectedly.	23/40 (58%) participants always used 2 of 3 intervention elements<br><br>Overall satisfaction with the programme (Likert scale, 1= not at all, 5= very satisfied):<br>39.5% (17/43) participants rated very satisfied (highest %).<br>97.7% (42/43) participants rated at least "3= somewhat" or above.
**Albright (2015)**	5% (80/1586) recorded telephone counselling sessions evaluated against a checklist of the essential intervention components:<br><br>88% fidelity over the 12-month intervention to the essential intervention components.<br><br>96% calls covered barriers to MVPA discussion.<br>97% calls covered assessing participant's previous MVPA goal.<br>100% calls covered setting the participant's next MVPA goal.<br><br>The two components most	Community recruitment:<br>272 via adverts, e.g., magazines, radio stations;<br>170 randomised,<br><br>Kaiser Permanente recruitment:<br>3844 Postcards sent out;<br>1176 calls made;<br>419 interested in joining;<br>141 randomised.	I: n= 115 (74.7%)<br>C: n= 127 (80.9%)<br><br>Most frequent reasons for failure to complete the intervention:<br>13% Pregnancy.<br>9.5% Too busy.<br>6.1% Discontinued participation, no given reason.<br>3.5% Family/job issues.	TTCW group:<br>90.4% of the participants receiving ≥13 of the 17 scheduled calls.<br><br>78.3% of the participants viewed the website at least once.<br><br>75% of participants set incremental MVPA goals with a health educator during the counselling sessions over the 12-month intervention period.<br><br>Level of achieving set MVPA goals in the 3 phases among all participants:<br><br>High level (≥100% of MVPA goal achieved or exceeded):<br>40.6% of the time during Phase 1 (weekly calls).<br>39.9% of time during Phase 2 (biweekly

For peer review only

Supplementary file 6. Implementation data_27.05.21

	frequently not delivered:<br>Pedometer steps (asked in 68.8% calls).<br>MVPA resources (offered in 80% calls).			calls).<br>42.0% of time during Phase 3 (monthly calls).<br><br>Moderate level (50-99% MVPA goal achieved):<br>23.5% of the time during Phase 1.<br>28.4% of the time during Phase 2.<br>21.1% of the time during Phase 3.<br><br>Low level (0-49% MVPA goal achieved):<br>35.8% of the time during Phase 1.<br>31.7% of the time during Phase 2.<br>36.9% of the time during Phase 3.
**Benedetti (2020)**	Checklist to assess implementation, including programme fidelity, instructor knowledge, classroom, schedule, participants' attention and attendance:<br>All analysed items achieved an average of 98% fidelity.	2 of 5 health districts in Florianopolis were interested in participating, consisting 20 of 50 HCs.<br>6 HCs were interested, and had the physical structure and human resources to offer the programmes, thus were recruited.<br><br>4,071 older adults across the 6 HCs; 24.2% (985) individuals were considered eligible;<br>11.5% (114) of eligible participants recruited.	Post-intervention (3 months):<br>BCG: n= 18 (50%)<br>TEG: n= 33 (63.5%)<br>C: n= 23 (88.5%)<br><br>6 months:<br>BCG: n= 17 (47.2%)<br>TEG: n= 32 (61.5%)<br>C: n= 21 (80.8%)<br><br>12 months:<br>BCG: n= 13 (36.1%)<br>TEG: n= 28 (53.8%)<br>C: n= 17 (65.4%)	Overall, 49% of participants attended at least 75% of all sessions, with disengagement occurring mostly in the first three weeks of the study (42%).<br><br>Both intervention groups showed relatively high disengagement rates (BCG 50% vs. TEG 37%) with individuals in the BCG presenting lower rates of overall attendance (27% vs. 47%).
**Berendse n (2015)**	Fidelity:<br>24/25 interviewed HCPs were trained in Motivational Interviewing, and applied MI with the participants.<br><br>100% PTs made an exercise plan with the participants.	30 clusters invited.<br><br>411 participants recruited (with 2 to 30 subjects per cluster, 76.9% of participants referred by the GP).<br><br>Eligibility based on baseline data:<br>- 48.9% met the inclusion criteria.<br>- 10.0% healthy BMI/no comorbidities.	28 clusters remained<br><br>Participants:<br>I: n= 196 (79.4%)<br>C: n= 126 (76.8%)<br><br>From recorded data, the main reasons of drop-outs were health issues (31.5%),	**% = median of attended / planned number of meetings:**<br><br>LSA meetings:<br>I: 50.0%; C:66.7%<br>PT group meetings:<br>I: 47.1% to 61.5%; C: 0% (planned n= 0)<br>PT individual meetings:<br>I: 0% (planned 6 to 7); C: 33.3%

For peer review only - http://bmjopen.bmj.com/site/about/guidelines.xhtml

Supplementary file 6. Implementation data_27.05.21

	84.8% of the participants set exercise goals or made an exercise plan with an HCP. 79.9% Exercise plans or goals were made with PT, if participants attended any individual meeting with a PT.<br><br>5/6 dietitians made nutritional plans with the participants. 73.9% of the participants made set nutritional plan or goals with an HCP. 91.7% of the plans or goals were made with the dietitian, if participants attended any individual meeting with a dietitian.<br><br>96.9% participants reported LSA had explained the intervention clearly at the beginning.<br><br>226 participants (from both IG and CG) completed a questionnaire after 12 months:<br>40.7% Reported the LSA had explicitly concluded the intervention.<br>41.2% Reported the intervention was not concluded.<br>18.1% Did not know.<br><br>Dose Delivered:<br>1 PT in start-up programme	- 16.8% higher weight-related risk than the target population.<br>- 24.3% of participants' eligibility could not be checked.	and personal reasons (10.1%).	Dietitian group meetings:<br>I: 42.9%; C: 28.6%<br>Dietitian individual meetings:<br>I: 33.3%; C: 133.3%<br><br>Satisfaction (on scale of 1–10, 10 is best):<br>Mean range (across meeting types):<br>I: 7.1 – 8.0<br>C: 7.1 – 7.3<br>Overall programme (Mean (SD)):<br>I: 7.7 (1.5)<br>C: 7.1 (1.8)

For peer review only

Supplementary file 6. Implementation data_27.05.21

	only planned group meetings with all HCPs, instead of the individual meetings intended per protocol.<br><br>4 dietitians typically offered individual meetings with participants, as per protocol. The other 4 dietitians only planned individual meetings according to participant's preference.			
**Biddle (2017)**	Not reported	Not reported	*I: n= 41 (43.6%)<br>*C: n= 68 (73.1%)<br><br>Reasons for failure to complete the intervention or loss to follow-ups:<br>24.5% (23/94) Did not receive allocated intervention in the intervention group.<br>16% (30/187) No longer want to participate.<br>13.4% (25/187) Failed to attend FU appointment.	23/94 (24%) allocated to intervention group did not attend the structured education workshop.<br>45/94 (47.9%) took part in Week 6 phone progress reviews<br><br>26/31 (84%) participants used the accelerometer daily initially, but this fell to 13/31 participants at 6 weeks.<br><br>25/31 (81%) participants felt the accelerometer as helpful at 6 weeks.<br><br>Workshop feedback:<br>Behaviour change plans for future (6 weeks):<br>4/38 (11%) referred to strategies to sit less<br>17/38 (45%) planned for physical activity<br>Others referred to desired health outcomes<br><br>"Best bits" of the workshop (mentioned most frequently): 1. information on diabetes; 2. the atmosphere of the workshop; 3. Receiving personal data on

Supplementary file 6. Implementation data_27.05.21

				sitting levels and health.<br><br>Behaviour change strategies attempted as reported by participants:<br>18 strategies mentioned to sit less and 8 strategies to move more.
**Blunt (2018)**	Only qualitative data reported	***How recruited participants heard about the study:**<br>51 (45%) from posters or handouts;<br>28 (25%) received an email from the study site advertising the project;<br>15 (13%) from an in-person study recruiter;<br>12 (11%) referred by their health care provider (HCP) and/or HCP team;<br>6 (5%) by word of mouth;<br>1 (1%) other unspecified methods<br>Five did not specify how they heard about the study	*6 months:<br>I: n= 44 (74.6%)<br>C: n= 46 (78.0%)<br><br>3.4% (I: n= 2) Did not attend any session<br>6.8% (I: n= 5, C: n= 3) Personal/health reasons<br>3.4% (I: n= 3, C: n= 1) Time commitment<br>5.9% (I: n= 2, C: n= 5) No longer interested<br><br>*12 months:<br>I: n= 37 (63%)<br><br>*18 months:<br>I: n= 35 (59%)	***Attendance:**<br>5% attended no sessions;<br>17% attended 1 session;<br>10% attended 2 sessions;<br>20% attended 3 sessions;<br>48% attended all 4 sessions.<br>Across all sites, 40 participants (68%) were classified as programme completers. Among participants who completed the intervention programme, 30% attended 3 in-person sessions, 70% attended all 4 sessions.
**Elramli (2017)**	Not reported	320 participants invited:<br>106 (33.1%) did not respond;<br>122 (38.1%) ineligible;<br>92 (28.8%) assessed for eligibility;<br>76 (23.8%) randomised	3 months:<br>I: n= 36 (92.3%)<br>C: n= 26 (70.3%)<br><br>6 months:<br>I: n= 37 (94.9%)<br>C: n= 22 (59.5%)	Intervention attendance:<br>26 (66.7%) participants attended all 8 education sessions (6 sessions and 2 booster sessions)<br>28 (71.8%) attended 6 sessions<br>71.8 % attended the first booster session<br>76.9% attended the second booster session<br><br>Control group attendance:<br>21 (56.8%) participants attended the single group education session
**Harris (2018)**	Nurse session attendance and session content delivered	11,015 people invited to participate;<br>6,399 did not respond;	3 months:<br>Postal: n= 335 (98.8%)	Diary returned:<br>Postal: 268/339 (79%)

For peer review only

Supplementary file 6. Implementation data_27.05.21

	recorded by the nurses after each session.<br><br>Fidelity to content delivered was high in all sessions; the mean number of items delivered in session one was 11 (range 10–11); six (range 5–6) in sessions 2 and 3.<br><br>Duration of sessions reported by nurses and measured from records were not very far from the recommendation (±≤30% difference maximum).	548 were excluded as a result of self-reported PA guideline achievement;<br>1,023/10,467 (10%) were randomised.	Nurse: n= 335 (96.8%)<br>C: n= 335 (99.1%)<br><br>12 months:<br>Postal: n= 319 (94.1%)<br>Nurse: n= 317 (91.6%)<br>C: n= 329 (97.3%)<br><br>4.3% (Postal: n=15/339, Nurse: n=25/346, C: n=4/338) Withdrawn<br>1.4% (Postal: n=5/339, Nurse: n=4/346, C: n=5/338) Not able to be contacted	Nurse: 281/346 (81%)<br><br>Pedometer use (every day or most days) during 12-week intervention:<br>Postal: 238/294 (81%)<br>Nurse: 269/303 (89%)<br><br>Attending nurse sessions:<br>255/346 (74%) attended all three sessions.<br>258/263 (98%) attended session 3, and reported still using the pedometer and diary every day or sometimes.
**Lakerveld (2012)**	Only qualitative data reported	8,193 people of 12 general practices were invited according the age (30-50 years) and absence of DM or CVD.<br><br>2,401 (29.3%) responded positively;<br>1,186 (14.5%) declined;<br>921 (11.2%) of those who accepted invitation met the waist circumference inclusion criterion;<br>772 (9.4%) attended screening at clinic and consented;<br>622 (7.6%) fully eligible and randomised.	End of intervention (6 months):<br>I: n= 267 (85.0%)<br>C: n= 269 (87.3%)<br><br>12 months:<br>I: n= 249 (79.3%)<br>C: n= 253 (82.1%)<br><br>24 months:<br>I: n= 236 (75.2%)<br>C: n= 244 (79.2%)<br><br>Reasons for loss to follow-up:<br>15.1% (I: n=42/308, C: n=52/314) Unable to attend<br>3.7% (I: n=9/308, C: n=14/314) Withdrew consent<br>1.1% (I: n=5/308, C: n=2/314) Became pregnant<br>1.3% (IG n=5/308, C:	*207 (66%) participants received at least 1 face-to-face session, 78% of them were content with the sessions.<br><br>The median number of attended sessions was 2 (out of a max of 6).

For peer review only

Supplementary file 6. Implementation data_27.05.21

			n=3/314) Unable to contact<br>0.2% (I: n=1/308, C: n=0/314) Died<br>1.4% (I: n=2/308, C: n=7/314) Diagnosed type 2 DM	
**Lane (2010)**	Not reported	11,205 women registered for the Women's Mini Marathon completed a survey about their PA habits.<br><br>Consented respondents were followed up 2 months and 6 months afterwards respectively:<br>2,020 of them provided records of PA changes at both follow-ups;<br>414 of them were identified as having relapsed to insufficient levels of PA and invited to participate in the trial;<br>176 consented to participate.	Follow-up response rate (end of trial at 6 Weeks):<br>I: n= 55 (65%)<br>C: n= 57 (63%)	76% of Intervention group participants responded at 3 Weeks:<br>97% received the booklet(s)<br>90% found the booklet(s) useful<br>50% reported increase in PA levels<br>28.5% felt greater levels of motivation which led to PA increase<br>16% felt they had more knowledge on being active which led to PA increase<br>5% attributed the PA increase to training for the Mini Marathon for the following year<br><br>At end of trial (6 Weeks), receipt and use of materials provided:<br>95% of intervention group participants<br>80% of control group participants
**Matson (2018)**	Not reported	Not reported	*I: n= 29 (100%)<br>*C: n= 25 (80.6%)	Only qualitative data reported
**Matthews (2016)**	Only qualitative data reported	Sample was deemed representative of adults with intellectual disabilities: 91% (n = 93) had mild or moderate intellectual disability.	*End of intervention (12 weeks):<br>I: n= 45 (83.3%)<br>C: n= 43 (89.6%)<br><br>*24 weeks:<br>I: n= 42 (77.8%)<br>C: n= 40 (83.3%)<br><br>Reasons for loss to follow-up:<br>32.4% (I: n=20/54, C: n=13/48) Did not want to	*54 participants were assigned to intervention, and received the intervention.<br><br>*71% took part in all 3 planned face-to-face physical activity consultations.<br><br>*26% took part in 2 consultations<br>*3% took part in 1 consultation

For peer review only

Supplementary file 6. Implementation data_27.05.21

			continue<br>1% (I: n=1/54) Ill-health	
**Poston (2013)**	Goals were set at all group sessions, of which 88% were considered SMART by HTs according to their diaries.	According to information from the Health and Social Care Information Centre (2013), approximately 1:5 pregnant women would be eligible for inclusion.<br><br>473/656 (72%) eligible people declined to participate (43.0% of those who declined were in the lowest quintile for Index of deprivation indicating the most severe deprivation);<br>38% participated.	End of intervention:<br>I: n= 79 (84.0%)<br>C: n= 75 (84.3%)	82/94 (88%) attended at least one group session, and 60 (64%) attended 4 or more.<br><br>42 women (45%) received material from all eight sessions, 6 by full attendance (6%) and 36 when partly/wholly covered by subsequent phone contact.<br><br>Mean of 6.1 (SD 2.6) sessions were attended or partly/wholly covered for the intervention group.
**School of Public Health, HKU (2017)**	Fidelity checks were conducted for every session of the programmes, which ensured the quality of the intervention and the implementation of the key elements in the intervention.	8 participating Integrated Family Service Centres to recruit around 600 eligible parents.<br><br>728 (121.3% of target) randomised.	Trial Core session (baseline):<br>I: n= 357 (92.5%)<br>C: n= 316 (92.4%)<br><br>3 months:<br>I: n= 335 (86.8%)<br>C: n= 306 (89.5%)<br><br>6 months:<br>I: n= 328 (85.0%)<br>C: n= 298 (87.1%)<br><br>End of intervention -12 months:<br>I: n= 309 (80.1%)<br>C: n= 284 (83.0%)<br><br>Reasons for absence from sessions included occupied with other activities, took care of family, illness, could not be contacted, and abroad; the exact number of participants dropped out for each of these reasons cannot	Physical activity group: (386 randomised)<br>357 (92.5%) attended core (1st) session<br>355 (92.0%) attended booster session at 3 months<br>313 (81.1%) attended tea gathering at 6 months<br>281 (72.8%) attended Family Holistic Health session at 1 year.<br><br>Healthy diet group: (342 randomised)<br>316 (92.4%) attended core (1st) session<br>306 (89.5%) attended booster session at 3 months<br>292 (85.4%) attended tea gathering at 6 months<br>268 (78.4%) attended Family Holistic Health session at 1 year.<br><br>Participant's feedback at end of Physical activity programme (on a scale of 0-10, 10 is best) (Mean (SD)):<br>9.0 (1.2) Quality of intervention content<br>9.0 (1.2) Level of utility of the intervention<br><br>100% participants would recommend this

For peer review only

Supplementary file 6. Implementation data_27.05.21

			be ascertained.	intervention programme to their friends and families
**Spittaels (2007)**	Not reported	8,000 employees targeted via 6 worksites using email messages, posters and internal newsletters;<br>570 (7.1%) responded positively;<br>562 (7.0%) returned the baseline questionnaire with the informed consent, and then randomised.<br><br>~65% of participants met the minimal recommendations for physical activity at baseline despite explicit recruitment of inactive participants<br><br>31% participants were female, males comprising the majority of employees in the two biggest worksites for recruitment<br><br>Male participants already had high baseline physical activity scores compared to the general male population (72% vs. 57% meeting the recommendations), whereas female participants were more representative of the population (47% vs. 48% meeting the recommendations).	End of intervention:<br>Tailored advice+emails: n= 116 (66.7%)<br>Tailored advice: n= 122 (69.7%)<br>C: n= 141 (79.7%)	Recalled having received the tailored advice (% participants):<br>97% Tailored advice+emails group<br>94% Tailored advice group<br>53% Control group<br><br>Tailored advice+emails group satisfaction (% participants):<br>92% Received at least 3 of the 5 reinforcement emails<br>77% Read them completely<br>87% Satisfied by number of emails<br>86% Satisfied by frequency of emails<br>45% Felt emails were useful<br>33% Reported behavioural changes
**Stathi (2019)**	Not reported	2,000 mailed invitations were delivered in the target areas resulting in 230 responses from potential participants and activators (response rate 11.5%).<br><br>ACE participants: 154 (7.7%) requests for information packs. 65 (3.3%) people returned reply forms. 40 (2.0%) recruited.<br><br>Activators: 76 (3.8%) requests for information packs. 15 (0.8%) recruited after completing the training.	End of intervention:<br>Activator: n= 15 (100.0%)<br><br>Participants:<br>I: n= 19 (86.4%)<br>C: n= 13 (76.5%)<br><br>Reasons for dropping out prior to final measures:<br>7.7% (3/39) Ill-health<br>5.1% (2/39) Carer commitments	All participants who completed the intervention engaged with their activator at least 7 times as planned.<br><br>Of the 3 participants who dropped out: 2 met their activator less than 5 times but were contacted regularly by phone.

For peer review only - http://bmjopen.bmj.com/site/about/guidelines.xhtml

Supplementary file 6. Implementation data_27.05.21

			2.6% (1/39) Lack of time<br>2.6% (1/39) Moving to a different city	
**Williams (2019)**	Not reported	215 eligible service users contacted by letter and phone;<br>71 not interested;<br>104 not contactable;<br>40 (18.6%) recruited.	I: n= 16 (80.0%)<br>C: n= 17 (85.0%)	13 (65%) received intervention:<br>5 did not engage with intervention;<br>2 did not engage with intervention after education session.

Keys: * = Data from associated publications; ACE = Active, Connected, Engaged intervention; BCG = Behaviour change group; BMI = Body Mass Index; C = Control group; CVD = Cardiovascular disease; DM = Diabetes Mellitus; FU = Follow-up; GP = General practitioner; HC = Health centre; HCP = Health care provider; HT = Health trainer; I = Intervention group; LSA = Lifestyle advisor; MVPA = Moderate-to-vigorous physical activity; n = number of persons; PA = Physical activity; PT = Physiotherapist; SD = Standard deviation; SMART = Specific, Measurable, Achievable, Relevant, and Time Specific; TEG = Traditional exercise group; vs = versus

For peer review only

Supplementary file 7_SB results from RCTs associated with included PEs_27.05.21

**Study (Year)** Number of participants randomised	**Unit of outcome**	**Outcome measure(s) for SB**	**Intervention group Baseline**	**Control group Baseline**	**Intervention group End of intervention[a]**	**Control group End of intervention[a]**
**Adams (2012)**<br><br>I: 47 C: 28	1. Mean % of SB time per day (SD)<br>2. Mean sitting hours per week (SD)	1. Accelerometer<br><br>2. IPAQ	(n= 40)<br>SB: 47.42% (10.77)<br><br>Sitting time: 57.99 (29.70)	(n= 24)<br>SB: 50.7% (13.78)<br><br>Sitting time: 45.18 (34.88)	(n= 40)<br>SB: 49.16% (10.23)<br><br>Sitting time: 46.00 (28.91)	(n= 24)<br>SB: 50.39% (14.92)<br><br>Sitting time: 40.33 (40.68)
**Albright (2015)**<br><br>I: 138 C:140	Mean sitting hours per day (SD)	Active Australia Survey	Traveling to/from work: 1.19 (0.71)*<br>While at work: 2.02 (2.18)<br>Watching TV: 2.05 (1.33)<br>Using a computer at home: 1.27 (0.98)<br>Other leisure time (movies, dining out): 1.38 (1.01)<br>While holding/feeding baby: 2.93 (1.78)	Traveling to/from work: 1.41 (0.82)*<br>While at work: 2.52 (2.5)<br>Watching TV: 1.91 (1.36)<br>Using a computer at home: 1.41 (1.18)<br>Other leisure time (movies, dining out): 1.31 (1.05)<br>While holding/feeding baby: 3.20 (2.08)	Not published	Not published
**Benedetti (2020)**<br><br>BCG: 36<br>TEG: 52<br>C: 26	Baseline: Mean SB minutes per week (SD)<br><br>End of trial: Mean SB minutes per day (SE)	Accelerometer	BCG: 498.5 (113.6)<br>TEG: 529.8 (107.3)	522.8 (86.7)	Change between baseline and end of intervention (3 months):<br>BCG: -14.3 (56.3) [c]<br>TEG: -4.1 (62.2) [c]<br>*Change between baseline and end of trial (12 months):*<br>*BCG: -10.9 (59.9)* [c]<br>*TEG: 4.2 (78.6)* [c]	Change between baseline and end of intervention (3 months):<br>-25.6 (77.9) [c]<br>*Change between baseline and end of trial (12 months): -26.7 (68.3)* [c]
**Berendsen (2015)**<br><br>I: 247 C: 164	Not published	Accelerometer	Not published	Not published	Not published	Not published

For peer review only

Supplementary file 7_SB results from RCTs associated with included PEs_27.05.21

**Biddle (2017)**<br><br>1: 94 C:93	Mean SB hours per day (95% CI)	1. Actigraph (worn on waistband)<br>2. ActivPAL (worn on thigh)	Actigraph (n= 76): 10.83 (10.50, 11.17) [c]<br>ActivPal (n= 60): 8.91 (8.59, 9.24) [c]	Actigraph (n= 80): 11.01 (10.76, 11.26) [c]<br>ActivPal (n= 57): 9.02 (8.73, 9.30) [c]	Outcomes not measured at end of intervention (6 Weeks).<br>*Change between baseline and end of trial (12 months)*<br>Actigraph *(n= 38): -0.29 (-0.75, 0.17)* [c]<br>*ActivPal (time change, n=32): 0.64 (0.13, 1.16)* [c]	Outcomes not measured at end of intervention (6 Weeks).<br>*Change between baseline and end of trial (12 months)*<br>*Actigraph n= 49): -0.23 (-0.60, 0.14)* [c]<br>*ActivPal (n=29): 0.58 (0.06, 1.09)* [c]
**Blunt (2018)**<br><br>I: 59 C:59	Mean sitting minutes per day (SD)	IPAQ	360 (315)	360 (240)	Mean difference between groups (only measured at end of active intervention phase – 6 months): -0.08 (-0.16, -0.006)* [c]	
**Elramli (2017)**<br><br>I: 39 C: 37	Mean SB hours per day (SE)	1. ActivPal<br><br>2. IPAQ	ActivPal SB: 18.0 (0.27) [c]<br>IPAQ weekday sitting: 5.3 (0.31)<br>IPAQ weekend sitting: 5.3 (0.36)	ActivPal SB: 18.5 (0.2) [c]<br>IPAQ weekday sitting: 4.7 (0.41)<br>IPAQ weekend sitting: 4.6 (0.38)	ActivPal SB: 17.2 (0.3) [c]<br>IPAQ weekday sitting: 4.2 (0.33)**<br>IPAQ weekend sitting: 3.9 (0.33)	ActivPal SB: 18.7 (0.41) [c]<br>IPAQ weekday sitting: 5.7 (0.53)**<br>IPAQ weekend sitting: 5.1 (0.63)
**Harris (2018)**<br><br>Postal: 339<br>Nurse: 346<br>C: 338	Mean SB minutes per day (SD, or 95% CI)	Accelerometer	Postal: 614 (71)<br>Nurse: 619 (78)	613 (86)	Mean difference between groups at end of intervention (3 months)<br>Postal versus control: -2 (-12, 7) [c]<br>Nurse versus control: -7 (-16, 3) [c]<br>Nurse versus Postal: -4 (-13, 5) [c]<br>Mean difference between groups at *end of trial (12 months)*<br>*Postal versus control: 1 (-8, 10)* [c]<br>*Nurse versus control: -0.2 (-9, 9)* [c]<br>*Nurse versus Postal: -1 (-10, 8)* [c]	
**Lakerveld (2012)**<br><br>I: 314 C: 308	Mean SB minutes per day (SD)	A subscale of AQuAA	253.7 (146.9) [c]	255.4 (124.5) [c]	Outcomes not measured at end of intervention<br>*End of trial (Month 24): 231.5 (122.2)* [c]	Outcomes not measured at end of intervention<br>*End of trial (Month 24): 233.0 (140.7)* [c]

For peer review only

Supplementary file 7_SB results from RCTs associated with included PEs_27.05.21

**Lane (2010)**<br><br>I: 85 C: 91	Mean sitting time minutes per week (SD)	Frequently used validated questions selected for the trial from other population-level PA interventions.	335.9 (194.9)	310.1 (224.7)	371.4 (170.1)	369.5 (152.6)
**Matson (2018)**<br><br>I: 29 C: 31	Mean sitting time minutes over last 7 days (SD)[b]	ActivPAL	Not published	Not published	Change between baseline and end of intervention (n= 29): -70.1 (104) [b]	Change between baseline and end of intervention (n= 25): 6.5 (69) [b]
**Matthews (2016)**<br><br>I: 54 C: 48	Mean% of time per day spent in SB (SD)	Accelerometer	64.2% (10.5)	66.9% (11.3)	(n= 42)<br>66.4% (10.0) [c]	(n= 40)<br>65.9% (12.0) [c]
**Poston (2013)**<br><br>I: 94 C: 89	Mean SB time minutes per day (SD)	1. Accelerometer<br><br>2. RPAQ	Accelerometer (n= 68): 1165 (91) [c]<br>RPAQ (n= 79): 1009 (187) [c]	Accelerometer (n= 72): 1172 (95) [c]<br>RPAQ (n= 80): 1007 (207) [c]	Accelerometer (n= 36): 1197 (77) [c]<br>RPAQ (n= 56): 1020 (226) [c]	Accelerometer (n= 39): 1175 (86) [c]<br>RPAQ (n= 54): 1068 (177) [c]
**School of Public Health HKU (2017)**<br><br>I: 357 C:316	Mean sitting hours in a working day (SD)	IPAQ-C	4.47 (2.47)*	4.11 (2.38)*	4.3	4.2
**Spittaels (2007)**<br><br>I:<br>Group 1 (tailored advice + email): 116<br>Group 2 (tailored advice): 122<br><br>C: 141	Mean sitting minutes per day (SD)	IPAQ	Group 1:<br>Weekday: 482 (183)<br>Weekend day: 308 (160)<br>Group 2:<br>Weekday: 492 (202)<br>Weekend day: 296 (160)	Weekday: 470 (217)<br>Weekend day: 309 (182)	Group 1:<br>Weekday: 443 (168)<br>Weekend day: 276 (131)<br><br>Group 2:<br>Weekday: 438 (172)<br>Weekend day: 268 (141)	Weekday: 419 (181)<br>Weekend day: 271 (139)
**Stathi (2019)**<br><br>I: 22 C: 17	Mean SB minutes per day (SD, +/ 95% CI)	Accelerometer	681.5 (74.9)	616.2 (112.3)	Change between baseline and end of intervention:	Change between baseline and end of intervention:

For peer review only

Supplementary file 7_SB results from RCTs associated with included PEs_27.05.21

					13.1 (77.2) (-26.6, 52.8)	-8.7 (70.7) (-57.6, 75.1)
**Williams (2019)**<br><br>I: 20 C: 20	Mean SB minutes per day (SE)	Accelerometer	577.2 (9.8)	549.2 (19.1)	End of intervention (17 weeks, n= 16): 520.9 (36.2)*<br><br>*End of trial (6 months, n= 8): 508.2 (19.4)**	End of intervention (17 weeks, n= 17): 637.9 (30.4)*<br><br>*End of trial (6 months, n= 13): 661.2 (33.5)**

Supplementary file 3: Sedentary behaviour measured (at baseline and end of the trial) in the randomised controlled trials associated with the included process evaluation studies

Keys: [a] = Results available from the assessment immediately after the intervention, unless otherwise specified; [b] = unclear if adjusted for covariates; [c] = data were adjusted for covariates; * = p value <0.05 for comparison between intervention and control groups (where reported); ** = p value <0.025 for comparison between intervention and control groups reported as accepted statistical significance ; *Italic font* = End of trial results, if available from publications; AQuAA = Activity Questionnaire for Adolescents & Adults; BCG = Behaviour Change Group; C = Control group; FU = Follow-up; I = Intervention group; IPAQ = International Physical Activity Questionnaire; IPAQ-C = International Physical Activities Questionnaire-Chinese version; n = number of persons included in the analysis; RPAQ = Recent Physical Activity Questionnaire; SB = Sedentary behaviour; SD = Standard deviation; SE = Standard error; TEG = Traditional Exercise Group

For peer review only

Supplementary file 8_characteristics of 24 excluded studies_27.05.21

Study	Reason for exclusion
Ashe 2013	Results of process evaluation not available.
Burton 1995	Ineligible study design: The study did not involve process evaluation.
Cohen 2017	Ineligible study design: The participants were not all assessed at all timepoints throughout the trial. The data from each time point were not obtained from the same sample group throughout the study.
Coll-Planas 2019	Results of process evaluation not available.
Douglas 2019	Ineligible study design: The study is not RCT.
Gray 2018	Sedentary behaviour was not measured in the RCT.
Gummelt 2017	Sedentary behaviour was not measured in the RCT.
Hammerback 2012	Sedentary behaviour was not measured in the RCT.
Harvey 2016	Ineligible study design: The study did not involve process evaluation of exploration of the intervention.
Holt 2019	Sedentary behaviour was not measured in the RCT.
Hsu 2013	Sedentary behaviour was not measured in the RCT.
Jayaprakash 2016	Sedentary behaviour was not measured in the RCT: Sedentary behaviour was measured at baseline, but not throughout the trial as an outcome.
Lai 2019	Ineligible study design: The study was not a RCT.
Maddison 2020	Sedentary behaviour was not measured in the RCT.
McAuley 2013	Ineligible study design: The study did not involve process evaluation.
Orme 2017	Ineligible study design: The evaluation of feasibility did not involve process evaluation or qualitative evaluation.
Rovniak 2014	Sedentary behaviour was not measured in the RCT.
Sazlina 2015	Results of process evaluation not available.
Seguin 2019	Sedentary behaviour was not measured in the RCT.
Sheppard 2016	Sedentary behaviour was not measured in the RCT.
Stevens 2015	Sedentary behaviour was not measured in the RCT.
Thomsen 2016	Ineligible study design: The study did not involve process evaluation or qualitative evaluation.
Thompson 2008	Results of process evaluation not available.
Thornton 2018	Ineligible comparator: The eligible intervention was assigned to the control group, not the experimental intervention group in this study.
Tiedemann 2015	Sedentary behaviour was not measured in the RCT.
van de Glind 2017	Results of process evaluation not available.
van der Wardt 2019	Sedentary behaviour was not measured in the RCT.
Varela-Mato 2016	Ineligible setting: The intervention was delivered at workplace.
Voorn 2016	Sedentary behaviour was not measured in the RCT.
Yeung 2020	Ongoing: Study not completed.
Zabaleta-Del-Olmo 2018	Results of process evaluation not available.

Keys: RCT = Ramdomised Controlled Trial

For peer review only

	SCREENING QUESTIONS		1. QUALITATIVE STUDIES					2. RANDOMIZED CONTROLLED TRIALS					3. NON-RANDOMIZED STUDIES					4. QUANTITATIVE DESCRIPTIVE STUDIES					5. MIXED METHODS STUDIES				
**Authors (Year)**	**S1**	**S2**	**1.1**	**1.2**	**1.3**	**1.4**	**1.5**	**2.1**	**2.2**	**2.3**	**2.4**	**2.5**	**3.1**	**3.2**	**3.3**	**3.4**	**3.5**	**4.1**	**4.2**	**4.3**	**4.4**	**4.5**	**5.1**	**5.2**	**5.3**	**5.4**	**5.5**
Adams (2012)	Yes	Yes	Yes	Yes	Yes	Yes	Yes	Yes	Yes	Yes	No	Yes						Yes	Yes	Yes	Yes	No	Yes	Yes	Yes	Yes	Yes
Albright (2015)	No	Can't tell						Can't tell	Yes	Can't tell	No	No						Yes	Yes	Yes	Yes	Yes					
Benedetti 2015	Yes	Yes	Yes	Yes	Yes	Yes	Yes	Yes	Yes	Can't tell	Can't tell	No						Yes	Yes	Yes	Can’t tell	Yes	Yes	Yes	Yes	Yes	Yes
Berendsen (2015)	Yes	Yes	Yes	Yes	Yes	Yes	Yes	No	Yes	Can't tell	Yes	Can't tell						Yes	Yes	Yes	Yes	Yes	Yes	Yes	Yes	Yes	Yes
Biddle (2017)	Yes	Yes	Yes	Yes	Yes	Yes	Yes	Yes	Yes	No	No	No						Yes	Yes	Yes	Yes	Yes	Yes	Yes	Yes	Yes	Yes
Blunt (2018)	Yes	Yes	Yes	Yes	Yes	Yes	Yes	Yes	Yes	Yes	No	No															
Elramli (2017)	Yes	Yes	Yes	Yes	Yes	Yes	Yes	No	Yes	No	No	No															
Harris (2018)	Yes	Yes	Yes	Yes	Yes	Yes	Yes	Yes	Yes	Yes	No	Yes						Yes	Yes	Yes	Yes	Yes	Yes	Yes	Yes	Yes	Yes
Lakerveld (2012)	Yes	No						Yes	Yes	Yes	No	Yes						Yes	Yes	Yes	Yes	Yes					
Lane (2010)	Yes	Yes						Can't tell	Yes	Yes	No	Yes						Yes	Yes	Yes	Yes	Yes					
Matson (2018)	Yes	Yes	Yes	Yes	Yes	Yes	Yes	Yes	Yes	Yes	Yes	Can't tell															
Matthews (2006)	Yes	Yes	Yes	Yes	Yes	Yes	Yes	Can't tell	Yes	Yes	No	Yes						Yes	Yes	Yes	Yes	Yes	Yes	Yes	Yes	Yes	Yes
Poston (2013)	Yes	Yes	Yes	Yes	No	No	No	Yes	Yes	Yes	Can't tell	Yes						Yes	Yes	Yes	Yes	Yes	Yes	No	No	Can't tell	No
SPH HKU (2017)	Yes	Yes	Yes	Yes	No	No	No	Can't tell	No	Yes	Can't tell	No															
Spittaels (2007)	Yes	Yes						Can't tell	Yes	Yes	Can't tell	Yes						Yes	Yes	Yes	Yes	Yes					
Stathi (2019)	Yes	Yes	Yes	Yes	No	No	No	Can't tell	No	Yes	Can't tell	Yes						Yes	Yes	Yes	Yes	Yes	Yes	No	No	Can't tell	No
Williams (2019)	Yes	Yes	Yes	Yes	No	No	No	Yes	Yes	No	Yes	No						Yes	Yes	Yes	Yes	Yes	Yes	Yes	Yes	No	No

S1. Are there clear research questions? S2. Do the collected data allow to address the research questions? 1.1. Is the qualitative approach appropriate to answer the research question? 1.2. Are the qualitative data collection methods adequate to address the research question? 1.3. Are the findings adequately derived from the data? 1.4. Is the interpretation of results sufficiently substantiated by data? 1.5. Is there coherence between qualitative data sources, collection, analysis and

interpretation? 2.1. Is randomization appropriately performed? 2.2. Are the groups comparable at baseline? 2.3. Are there complete outcome data? 2.3. Are there complete outcome data? 2.4. Are outcome assessors blinded to the intervention provided? 2.5 Did the participants adhere to the assigned intervention? 3.1 Are the participants representative of the target population? 3.2 Are measurements appropriate regarding both the outcome and intervention? 3.3 Are there complete outcome data? 3.4 Are the confounders accounted for in the design and analysis? 3.5 During the study period, is the intervention administered (or exposure occurred) as intended? 4.1. Is the sampling strategy relevant to address the research question? 4.2. Is the sample representative of the target population? 4.3. Are the measurements appropriate? 4.4. Is the risk of nonresponse bias low? 4.5. Is the statistical analysis appropriate to answer the research question? 5.1. Is there an adequate rationale for using a mixed methods design to address the research question? 5.2. Are the different components of the study effectively integrated to answer the research question? 5.3. Are the outputs of the integration of qualitative and quantitative components adequately interpreted? 5.4. Are divergences and inconsistencies between quantitative and qualitative results adequately addressed? 5.5. Do the different components of the study adhere to the quality criteria of each tradition of the methods involved?

For peer review only

BMJ Open

A SYSTEMATIC REVIEW OF PROCESS EVALUATIONS OF INTERVENTIONS IN TRIALS INVESTIGATING SEDENTARY BEHAVIOUR IN ADULTS

Journal:	*BMJ Open*
Manuscript ID	bmjopen-2021-053945.R1
Article Type:	Original research
Date Submitted by the Author:	24-Nov-2021
Complete List of Authors:	Johansson, Jessica; Bradford Institute for Health Research, Academic Unit of Ageing and Stroke Research; University of Leeds Leeds Institute of Health Sciences, Academic Unit for Ageing and Stroke Research<br>Lam, Natalie; Bradford Institute for Health Research, Academic Unit for Ageing and Stroke Research; University of Leeds Leeds Institute of Health Sciences, Academic Unit for Ageing and Stroke Research<br>Ozer, Seline; Bradford Institute for Health Research, Academic Unit for Ageing and Stroke Research; University of Leeds Leeds Institute of Health Sciences, Academic Unit for Ageing and Stroke Research<br>Hall, Jennifer; Bradford Institute for Health Research, Academic Unit for Ageing and Stroke Research; University of Bradford, Faculty of Life Sciences and Health Studies<br>Morton, Sarah; The University of Edinburgh Centre for Clinical Brain Sciences, Geriatric Medicine<br>English, Coralie; The University of Newcastle School of Health Sciences, Faculty of Health and Medicine<br>Fitzsimons, Claire F.; University of Edinburgh Physical Activity for Health Research Centre<br>Lawton, Rebecca; University of Leeds, Institute of Psychological Sciences; Bradford Institute for Health Research, Quality and Safety Research<br>Forster, Anne; University of Leeds Leeds Institute of Health Sciences, Academic Unit for Ageing and Stroke Research ; Bradford Institute for Health Research, Academic Unit for Ageing and Stroke Research<br>Clarke, David; University of Leeds Leeds Institute of Health Sciences, Academic Unit for Ageing and Stroke Research ; Bradford Institute for Health Research, Academic Unit for Ageing and Stroke Research
**Primary Subject Heading**:	Public health
Secondary Subject Heading:	Health services research
Keywords:	PUBLIC HEALTH, Quality in health care < HEALTH SERVICES ADMINISTRATION & MANAGEMENT, REHABILITATION MEDICINE

SCHOLARONE™
Manuscripts

I, the Submitting Author has the right to grant and does grant on behalf of all authors of the Work (as defined in the below author licence), an exclusive licence and/or a non-exclusive licence for contributions from authors who are: i) UK Crown employees; ii) where BMJ has agreed a CC-BY licence shall apply, and/or iii) in accordance with the terms applicable for US Federal Government officers or employees acting as part of their official duties; on a worldwide, perpetual, irrevocable, royalty-free basis to BMJ Publishing Group Ltd ("BMJ") its licensees and where the relevant Journal is co-owned by BMJ to the co-owners of the Journal, to publish the Work in this journal and any other BMJ products and to exploit all rights, as set out in our licence.

The Submitting Author accepts and understands that any supply made under these terms is made by BMJ to the Submitting Author unless you are acting as an employee on behalf of your employer or a postgraduate student of an affiliated institution which is paying any applicable article publishing charge ("APC") for Open Access articles. Where the Submitting Author wishes to make the Work available on an Open Access basis (and intends to pay the relevant APC), the terms of reuse of such Open Access shall be governed by a Creative Commons licence – details of these licences and which Creative Commons licence will apply to this Work are set out in our licence referred to above.

Other than as permitted in any relevant BMJ Author's Self Archiving Policies, I confirm this Work has not been accepted for publication elsewhere, is not being considered for publication elsewhere and does not duplicate material already published. I confirm all authors consent to publication of this Work and authorise the granting of this licence.

For peer review only

A SYSTEMATIC REVIEW OF PROCESS EVALUATIONS OF INTERVENTIONS IN TRIALS INVESTIGATING SEDENTARY BEHAVIOUR IN ADULTS

Authors:

[1,2] Jessica Faye Johansson Jessica.Johansson@bthft.nhs.uk (corresponding author); [1,2]Natalie Lam Natalie.Lam@bthft.nhs.uk; [1,2] Seline Ozer Seline.Ozer@bthft.nhs.uk; [1,3] Jennifer Hall Jennifer.Hall@bthft.nhs.uk; [4] Sarah Morton sarah.morton@ed.ac.uk ; [5]Coralie English Coralie.English@newcastle.edu.au; [6] Claire Fitzsimons Claire.Fitzsimons@ed.ac.uk; [7] Rebecca Lawton R.J.Lawton@leeds.ac.uk;[2]Anne Forster A.Forster@Leeds.ac.uk; [2] David Clarke D.J.Clarke@leeds.ac.uk.

[1]Academic Unit for Ageing and Stroke Research, Bradford Institute for Health Research, Temple Bank House, Bradford Royal Infirmary, Bradford, BD9 6RJ, UK

[2] Academic Unit for Ageing and Stroke Research, University of Leeds, Temple Bank House, Bradford Royal Infirmary, Bradford, BD9 6RJ, UK

[3] Faculties of Life Sciences and Health Studies, University of Bradford, Richmond Road, Bradford, BD7 1D7, UK

[4] Geriatric Medicine, Division of Health Sciences, Centre for Clinical Brain Sciences, Room F1424, Royal Infirmary, Edinburgh, EH16 4SB, UK

[5] Physiotherapy Program, School of Health Sciences, Faculty of Health and Medicine, HC-36 Hunter Building, The University of Newcastle, New South Wales 2308, Australia

[6] Physical Activity for Health Research Centre, The University of Edinburgh, St Leonard's Land, Holyrood Road, Edinburgh, EH8 8AQ, UK

[7] Yorkshire Quality and Safety, Bradford Institute for Health Research, Temple Bank House, Bradford Royal Infirmary, Bradford, BD9 6RJ, UK

Word count: 8365

Abstract

Objectives: To systematically review and synthesise findings from process evaluations of interventions in trials which measured sedentary behaviour as an outcome in adults to explore: 1) how intervention content, implementation, mechanisms of impact and context influence outcomes; 2) how these interventions are experienced from different perspectives (participants, carers, staff).

Design: Systematic review and narrative synthesis underpinned by the Medical Research Council (MRC) process evaluation framework.

Data sources: Databases searches were conducted in March 2019 then updated in May 2020 and October 2021 in: CINAHL; SPORTDiscus; Cochrane Database of Systematic Reviews; Cochrane Central Register of Controlled Trials; AMED; EMBASE; PsycINFO; MEDLINE; Web of Science; and ProQuest Dissertations & Theses.

Eligibility criteria: We included: Process evaluations of trials including interventions where sedentary behaviour was measured as an outcome in adults aged 16 or over from clinical or non-clinical populations. We excluded studies if interventions were delivered in educational or workplace settings, , or if they were laboratory studies focused on immediate effects of breaking sitting.

Data extraction and synthesis: Two independent reviewers extracted and coded data into a framework and assessed the quality of studies using the Mixed Methods Appraisal Tool. We synthesised findings using a narrative approach.

Results: 17 process evaluations were included. Five interventions focused on reducing sedentary behaviour or sitting time, 12 aimed to increase physical activity or promote healthier lifestyles. Process evaluations indicated changes in sedentary behaviour outcomes were shaped by numerous factors including: barriers (e.g. staffing difficulties and scheduling problems) and facilitators (e.g. allowing for flexibility) to intervention delivery; contextual factors (e.g. usual lifestyle and religious events); and individual factors (e.g. pain, tiredness, illness, age, and individual preferences).

For peer review only - http://bmjopen.bmj.com/site/about/guidelines.xhtml

Discussion: Intervention requires careful consideration of different factors that could influence changes in sedentary behaviour outcomes to ensure that interventions can be tailored to suit different individuals and groups.

PROSPERO registration number: CRD42018087403

Key words: Sedentary behaviour, systematic review, process evaluation

Strengths and limitations of this study

- This systematic review is guided by Preferred Reporting Items for Systematic Review and Meta-analysis (PRISMA) guidance.
- This is the first systematic review which has synthesised data from process evaluations evaluating interventions in trials that measure sedentary behaviour as an outcome in adults.
- The Medical Research Council guidance for conducting process evaluations has been used to structure this review and provided a comprehensive way of identifying factors associated with implementation, mechanisms of impact and context which may influence the effectiveness of randomised controlled trials investigating sedentary behaviour in adults.
- Non-English electronic databases were not searched. This limitation may cause language bias.
- There is some inconsistency in the quality of the reporting of the process evaluations included in the review .

INTRODUCTION

Sedentary behaviour is defined as any waking behaviour characterised by energy expenditure ≤1.5 Metabolic Equivalents (METS) while in a sitting, lying or reclining posture(1). In recent years, research exploring sedentary behaviour in adults has been expanding rapidly, documenting the potential for sedentary behaviour to have detrimental effects on health, wellbeing, and healthcare costs(2). Randomised controlled trials (RCTs) are particularly useful to examine intervention effectiveness(3). However, this approach cannot fully account for how interventions work, and the degree to which intervention components contribute to effectiveness or ineffectiveness(4).

Interventions targeting sedentary behaviour are typically complex, with multiple interacting components (5). Changes in outcomes following interventions are largely influenced by human behaviours and contextual factors as part of a complex process(6). The value of studying intervention processes, is recognised in the Medical Research Council (MRC) guidelines for developing and evaluating complex interventions(3) and detailed in the guidance for conducting process evaluations of complex interventions(4). Process evaluations are designed to help understand the theoretical assumptions underpinning an intervention, and to disentangle factors which may have contributed to the outcomes of an intervention(4).

The MRC process evaluation framework states that understanding of causal assumptions underpinning interventions and evaluation of how interventions work in practice are vital in building an evidence base that informs policy and practice. The framework outlines key functions of a process evaluation including investigating implementation, mechanisms of impact and context to understand how outcomes are interpreted(4).

To date, systematic reviews have synthesised the evidence of effectiveness of interventions aimed at reducing sedentary behaviour(7, 8). However, it is also important to synthesise findings from process evaluations to

For peer review only - http://bmjopen.bmj.com/site/about/guidelines.xhtml

understand the complexity of factors that may influence whether interventions are effective in reducing sedentary behaviour as these will inform future interventions in this relatively new research area. This paper seeks to address the following aims and objectives (table 1):

Aims and objectives

1) To identify process evaluations of interventions in trials which measured sedentary behaviour as an outcome in adults, to understand the intervention content, mechanisms of impact, implementation and delivery approaches and contexts, in which interventions were reported to be effective or ineffective.
2) To explore experiences of participants, family members/carers and intervention staff in interventions that measured sedentary behaviour as an outcome in adults.

Table 1: Review objectives

1. To identify and record the trial data (e.g., design of interventions, sample sizes, duration and content of interventions, and primary and secondary outcome data (from the process evaluation publication or associated publications).
2. Establish whether logic models or theoretical models were used to explain how interventions were intended to work.
3. Establish whether interventions were delivered as intended (as per protocol).
4. Explore intended or unintended mechanisms that influence the extent to which interventions are effective.
5. Understand barriers and facilitators to delivery of, and participation in, interventions and any recommendations made to address such barriers and facilitators.
6. To synthesise qualitative data concerning the understanding and experiences of interventions from the perspectives of participants, family members/carers and intervention staff.

Qualitative data related to exploring perceptions, views and lived experiences of sedentary behaviour, but not related to receipt or delivery of an intervention were examined in a separate systematic review(9).

The MRC process evaluation framework(4) was the underpinning framework for this review informing the aims and objectives, coding framework, providing a structure for synthesising and reporting findings.

METHODS

Protocol and registration

Reporting of this systematic review is guided by Preferred Reporting Items for Systematic Review and Meta-analysis (PRISMA) guidance,(10) (Supplementary file 1). The review was prospectively registered with PROSPERO (Prospective Register of Systematic Reviews); registration number: CRD42018087403, the protocol has been published (11).

Patient and Public Involvement

No patients involved.

Eligibility criteria

Study design

Studies explicitly identified by authors as a process evaluation, or studies that aimed to understand the functioning of an intervention by examining implementation, mechanisms of impact, and contextual

For peer review only - http://bmjopen.bmj.com/site/about/guidelines.xhtml

factors(12). Only process evaluations of RCTs, cluster RCTs, and randomised cross-over trials were included. Cohort and uncontrolled before- and- after studies were excluded.

Participants

Adults aged 16 or over regardless of whether they were recruited from a clinical or nonclinical population.

Interventions

Interventions which measured sedentary behaviour as an outcome, even if reducing sedentary behaviour was not the primary outcome.

Interventions were excluded if: they were delivered primarily in schools, colleges, universities, or a workplace; or aimed at the acute (immediate) effects of breaking up sitting time as part of a supervised (usually laboratory-based) intervention.

Comparators

In trials, intervention groups may be compared to: no treatment, usual care, attention control, waitlist control groups, or alternative treatments.

Information sources

Electronic sources

In collaboration with information specialist colleagues, comprehensive search strategies were developed using controlled vocabulary and free text terms (Supplementary File 2 for the search strategy for the MEDLINE database). Searches were conducted in March 2019 then updated in May 2020 and October 2021.
We searched the following databases: CINAHL (EBSCOHost); SPORTDiscus (EBSCOHost); Cochrane Database of Systematic Reviews (Wiley); Cochrane Central Register of Controlled Trials (Wiley): AMED (OVID); EMBASE (OVID); PsycINFO (OVID); Ovid MEDLINE(R); OVID MEDLINE(R) and Epub Ahead of Print, In-Process & Other Non-Indexed Citations; Web of Science: Sciences Citation Index Expanded (Clarivate); Web of Science: Social Sciences Citation Index Expanded (Clarivate); Web of Science: Conference Proceedings Citation Index- Science (Clarivate); Web of Science: Conference Proceedings Citation Index- Social Sciences and Humanities (Clarivate); ProQuest Dissertations & Theses.

Searching other sources

In addition to searching electronic databases, we identified process evaluations through examining included studies from a concurrent systematic review and meta-analysis of RCTs that explored the effects of interventions in reducing sedentary behaviour, using the same eligibility criteria for participants, interventions and comparators (Hall et al., , 2021 (13)). For each included study in the systematic review and meta-analysis of RCTs , we identified whether a process evaluation was conducted alongside the RCT and included all those identified. If the process evaluation results were not available, we contacted study authors for results.

Study records

Data management

References identified from electronic databases and other sources were de-duplicated and imported into Endnote X7 reference management software. References were then imported in to Covidence (www.covidence.org, 28th April 2021), a web-based systematic review tool.

Selection process

Using Covidence, two reviewers (RC, NL) independently assessed titles and abstracts of records from the electronic searches against the eligibility criteria and excluded obviously irrelevant studies. The full-text of the remaining studies were obtained; then independently assessed, by the same reviewers, against the eligibility

For peer review only - http://bmjopen.bmj.com/site/about/guidelines.xhtml

criteria to determine which studies would be eligible for inclusion. The same process for updated literature searches was undertaken (by NL, SO). During the screening process, disagreements were resolved by a consensus-based decision between the reviewers, or if necessary, discussion with a third reviewer (DJC).

Data extraction and narrative synthesis

A narrative approach to synthesising data was undertaken to provide detailed written commentary to address the research aims and objectives. Reviewers (RC, NL, and JFJ) independently extracted relevant quantitative and qualitative data from included studies. All quantitative data was checked by a second reviewer (SO). Fifty percent of the qualitative data was compared by NL and JFJ.

Developing and refining the framework

To direct data extraction, a framework was produced based on this review's aims, objectives and data to be extracted as specified in the protocol (11). The six themes and relevant subthemes align with the key functions in the MRC process evaluation framework (4) (Table 2). Data extraction items (related to the trial and process evaluations) (11) were coded into the framework then summarised in a series of files focusing on: the characteristics of trials (Supplementary file 3), characteristics of process evaluations (Supplementary file 4), delivery methods and mechanisms of impact (Supplementary file 5), and implementation data including fidelity, recruitment, retention and reach (Supplementary file 6). Within file 6, we have included definitions of these terms; informed by three key papers (4, 14, 15). Qualitative data from the framework is presented in the 'narrative synthesis findings' section.

To help understand the effects of each included intervention on sedentary behaviour outcomes, the sedentary behaviour measures from the associated RCTs were also extracted (Supplementary file 7). As the review focuses on the findings from the process evaluations, the treatment effects estimated in the RCTs have not been synthesised or analysed.

Two reviewers (JFJ, NL) independently coded one study to pilot the framework. Following discussion, minor refinements were made before the final framework was agreed. For example, engagement was added in to barriers and facilitators to participation in the intervention, a clearer definition of context was added and a 6th 'miscellaneous' theme was included to code data about trial procedures and qualitative methods, mainly for context where appropriate. The coding rules were also refined, then used in coding the remainder of the included studies.

Table 2: Coding framework

Themes and sub-themes	Definition / descriptions of what should be coded
**1. Implementation data**	
1a. Intended delivery	How the intervention was intended to be delivered (in main paper or protocol)
1b. Actual delivery (including when adapted)	How the intervention was actually delivered , including when it has been adapted from what was intended
1c. Strategies for achieving delivery	How the intervention delivery was achieved (e.g. tailoring interventions to individuals)
1d. Measures of adherence	A measure of adherence that was used in the study (NB: may be some overlap with compliance/fidelity). Definition adopted: *"The extent to which delivered content, frequency, duration and coverage of intervention components/ material are as intended."*
**2. Mechanisms of impact**	
2a. Logic models used to explain	Coded when a logic model is present

For peer review only - http://bmjopen.bmj.com/site/about/guidelines.xhtml

how the intervention was intended to work	
2b. Theories underpinning the intervention	Theories underpinning the intervention e.g. trans-theoretical model , social cognitive theory and behaviour change techniques (BCTs) from the 93-item taxonomy used as part of the intervention e.g. goal setting, self-monitoring NB: still coded BCTs even if authors do not make reference to a BCT taxonomy
2c. Mediators of change	Factors that explained how the intervention had an effect.
2d. Responses to and interactions with the intervention	Instances where participants or those providing the intervention talked about how they responded to, or interacted with the intervention
2e. Intended mechanisms of action influencing intervention effectiveness	How the intended mechanisms of action influenced effectiveness (e.g. intended mechanism of effect- self monitoring of daily activity)
2f. Unintended mechanisms of action influencing intervention effectiveness	Descriptions of how unintended mechanisms of action influenced effectiveness (e.g. if social support increased intervention effectiveness but the intended mechanism was self-monitoring)
**3. Contextual factors influencing effective and ineffective interventions** (Context includes anything external to the intervention that may act as a barrier or facilitator to its implementation or its effects (4)).	
3a. Influencing implementation	Anything external to the intervention that may have influenced its implementation
3b. Influencing mechanisms	Anything external to the intervention that may have influenced the mechanisms by which the intervention had an effect (or not)
3c. Influencing outcomes	Anything external to the intervention that may have influenced the outcomes of the intervention
**4. Barriers and facilitators**	
4a. Barriers to delivery of intervention	Factors that hindered the delivery of the intervention (including internal factors)
4b. Facilitators to delivery of intervention	Factors that enhanced the delivery of the intervention (including internal factors)
4c. Barriers to participation and/or engagement in intervention	Factors that hindered participation or engagement in the intervention: "The extent to which participants understand, accept and enact specific components of the programme in their daily lives."
4d. Facilitators to participation and/or engagement in intervention (e.g. incentives)	Factors that enhanced the delivery of the intervention. Definition as above.
4e. Recommendations made to address barriers and facilitators.	Recommendations made to overcome the barriers and facilitators (from either the study participants (including those delivering)) or the authors of the paper.
**5. Understanding and experiences of interventions from different perspectives**	
5a. Participants' experiences	Experiences from the perspectives of participants that cannot otherwise be coded into context, or barriers and facilitators (likely to be direct quotations)
5b. Family and carers' experiences	Experiences from the perspectives of family and carers that cannot otherwise be coded into context, or barriers and facilitators. Carers defined as unpaid and informal carers so includes friends and relatives but not paid carers.
5c. Staffs' experiences	Experiences from the perspectives of staff that cannot otherwise be coded into context, or barriers and facilitators. Paid carers that are involved in the

For peer review only - http://bmjopen.bmj.com/site/about/guidelines.xhtml

	intervention would be included here.
5d. Control group experiences	Experiences from control group participants if reported
**6. Miscellaneous**	
**6a. Trial procedures data**	Instances where study includes information that is more focused on the data collection e.g. recruitment and retention, rather than the intervention. Agreed not to code any quantitative data that is otherwise captured elsewhere in the review.
**6b. Qualitative methods (to provide context)**	Reports of how qualitative data collection was undertaken e.g. 'semi-structured interviews were conducted with 10 staff.'

Coding into the framework

Using the framework, JFJ independently coded all included studies. Nine studies (every other study listed alphabetically) were coded independently by NL. Coding was managed using NVivo software version 12 Plus(16).

Comparing codes

JFJ and NL compared data from the nine studies coded by both researchers. To enhance the rigour of the process, JFJ then re-reviewed all studies coded singly to ensure consistency(17).

Methodological quality

Methodological quality of included studies was assessed using the Mixed Methods Appraisal Tool (MMAT)(18), which is designed to concurrently assess qualitative, quantitative, and mixed methods studies. Three reviewers (NL, RC, JFJ) independently assessed the quality of studies and resolved any discrepancies by making a consensus-based decision, or if necessary, by discussion with a fourth reviewer (DJC). Studies were not excluded from the synthesis based on the outcome of the quality assessment.

For peer review only

For peer review only - http://bmjopen.bmj.com/site/about/guidelines.xhtml

RESULTS

The PRISMA flow diagram (Figure 1), presents results from all searches. Database searches identified 3,167 records; 116 additional records were identified through other sources. After removing duplicates (n = 1,113), 2,170 titles and abstracts were screened; 2,088 records were excluded as they did not meet the pre-defined eligibility criteria. The full-text reports of the remaining 82 records were assessed for eligibility, of which 24 reports were assessed as ineligible. The results of process evaluations of six eligible studies (seven reports) were unavailable. In total, 17 process evaluation reports were included for data synthesis. Fifty associated reports were also retained to address objective one.

For peer review only

Figure 1: PRISMA flow diagram

For peer review only - http://bmjopen.bmj.com/site/about/guidelines.xhtml

Record of excluded studies

Supplementary file 8 provides reasons for excluding the 24 studies outlined in Figure 1.

Summary of included studies

Included randomised controlled trials

To address objective 1, and provide context for the process evaluations, supplementary file 3 presents data from trials with included process evaluations, including: aims, inclusion/exclusion criteria, sample size, participant characteristics, study design, intervention and control descriptions, data collection and follow-up time-points and outcome measures used.

RCT aims

Associated trials where sedentary behaviour was measured as an outcome were published between 2007-2020. Five trials focused specifically on reducing sedentary behaviour (19-21) or sitting time(22, 23). The remaining 12 trials aimed to increase physical activity or promote healthier lifestyles but measured sedentary behaviour as an outcome (Supplementary file 3).

Trial location and participant characteristics

Seven trials were conducted in the UK(20-22, 24-27), the remainder in the USA(19, 23, 28), Netherlands(29, 30), Brazil(31), Ireland(32), Canada(33), Hong Kong(34), and Belgium(35). Participants recruited into the trials varied, including: mothers or parents of infants, pregnant women, adults, older adults, overweight adults, individuals with chronic illnesses, and individuals with intellectual disabilities or serious mental illnesses. Most trials included males and females, however three included females only(19, 26, 28). Participants' ages ranged between 30 and 75; the majority of trials included participants aged between 40 and 50 years(19-21, 24, 25, 29, 30, 32, 33). Only nine trials reported ethnicity, the most ethnically diverse study was by Albright et al., (28) which reported the following ethnicities: Native Hawaiian, Pacific Islander, Asian, mixed race, white, black-Native American.

Included process evaluations

Supplementary file 4 presents data specific to the process evaluations including: aims and whether process evaluations were pre-specified, sample size and sampling methods, study design and data collection methods, and theoretical frameworks used. These data provide further context for the narrative synthesis.

Thirteen process evaluations were pre-specified in published protocols, or trial register records. Five studies(19, 26, 30, 32, 35) were published prior to the MRC guidance (4), which was developed to provide a more systematic approach for planning and conducting process evaluations. The majority were published in the same year or after the guidance was published(20-25, 27-29, 31, 33, 34). Despite this, nine studies did not report using a framework or guidance (19, 21, 23, 24, 28, 32-35) only four authors cited the MRC guidance(20, 22, 25, 27) and only one reported using this to guide the process evaluation(25). As shown in supplementary file 4, five studies cited other frameworks (20, 26, 29-31) the most common alternative to the MRC framework being the RE-AIM framework (36). Fourteen used the term 'process evaluation' within the publication. Three did not use this term(23, 24, 34).

Process evaluation aims

There was considerable variation in process evaluation aims. Some studies had a broad focus on participants' experiences for example, Elramli (24) aimed to explore participants' views regarding the effectiveness of a walking intervention for rheumatoid arthritis (RA). Others focused more specifically on barriers to achieving activity goals(28), or barriers and facilitators to the sustainability of an intervention(29). Some focused on the feasibility and/or acceptability of interventions among different participant groups, including those at risk of

For peer review only - http://bmjopen.bmj.com/site/about/guidelines.xhtml

chronic disease (33); older adults(23); individuals with intellectual disabilities(20); and individuals with serious mental illnesses(21). Only two process evaluations were conducted with a view to refine the intervention(26, 27).

Study design and data collection methods

As outlined in supplementary file 4, sample sizes of participants recruited to the process evaluations varied, from five(21) to 411(29). A total of 1553 participants were included from intervention groups across the 17 studies and 340 from control groups in four studies(26, 29, 34, 35).

Nine studies(19-22, 25-27, 29, 31) used mixed-methods, most commonly combining quantitative questionnaires with semi-structured interviews (telephone and face-to-face). In five studies, questionnaires were used to ask participants about their satisfaction with the intervention, intervention fidelity, and about suggested improvements to interventions(19, 27, 29, 32, 35). In two studies, questionnaires focused on intervention providers' experiences of delivering and participating in interventions(25, 30).

Semi-structured interviews explored intervention contexts, barriers and facilitators to intervention delivery , and experiences from the perspective of intervention providers, participants, and their family members or carers(21-27, 29, 31, 33, 34). Other methods used included: non-participant observations (19), focus groups(20, 25, 27, 31, 34), healthcare professionals' registries and log books(29).

Methodological quality

Supplementary file 9 provides an overview of answers to questions in relevant categories of the MMAT(18) for all included studies. Options include, 'yes', indicating a positive judgement, 'no', indicating a negative judgement, or 'can't tell,' which is used when there is insufficient information to make a judgement. MMAT authors discourage calculating an overall score and excluding studies based on their methodological quality(18). Therefore all studies remained included in the synthesis and were not weighted. Below is a summary of the assessment of each of the six categories.

- Screening questions

The majority of studies had clear research questions or aims, and appropriate data were collected.

1. Qualitative studies

Thirteen of 17 included studies had a qualitative component. Four(21, 26, 27, 34) were rated as not meeting some of the criteria in this category, because descriptions of the analysis process lacked detail, and it was unclear how authors arrived at their findings. In these studies, findings were commonly presented as a series of quotes, in tables or supplementary files but interpretation was considered too limited to constitute an in-depth analysis.

2. Randomised controlled trials

Each of the included studies was associated with a RCT. This category of the MMAT was used to assess the quality of the trials. The 'can't' tell' option was most commonly used in this section because authors often provided insufficient information to provide an answer, particularly regarding the randomisation process and blinding. Scoring was more mixed within this category and no studies scored yes for all questions.

3. Non-randomized studies

The associated trials were all RCTs; therefore this category was not applicable.

4. Quantitative descriptive studies

For peer review only - http://bmjopen.bmj.com/site/about/guidelines.xhtml

Thirteen studies had a quantitative component. Overall, they were rated positively across all questions.

5. Mixed methods studies

We considered studies which used methods meeting the criteria for both categories 1 and 4 as mixed methods studies. This category was only applicable for nine studies. When studies were rated negatively on either the qualitative or quantitative component, it was reflected in the judgement for this category.

Narrative synthesis findings

This section reports on the findings from the 17 process evaluations coded into the framework and summarised in narrative form. Subheadings based on the key functions of a process evaluation outlined in MRC guidance by Moore et al.,(4) have been applied to organise the data. Figure 2 (based on Moore et al (4)) outlines summary findings for each subheading in the synthesis and identifies some key findings.

Description of the interventions and their causal assumptions

According to Moore et al.(4) a clear description of the intervention and its causal assumptions are an important part of understanding how other factors (e.g. implementation, context and mechanisms of impact) influence outcomes.

Supplementary file 5 describes the content and delivery methods for all interventions. Intervention delivery periods ranged between 6 weeks and 18 months. All interventions included multiple components, examples include group based educational sessions combined with email input and self- monitoring tools(19) or one-to-one counselling combined with tailored email input(28). In terms of delivery, interventions commonly incorporated some group based input or support(19, 21, 22, 24-26, 29, 31, 34). Interventions were delivered by a range of providers including researchers(19), health educators (22, 28), exercise professionals, including personal trainers(20, 29), coaches(21, 23, 33), advisors and nurses(25, 30).

Supplementary file 5 also includes information about the mechanisms by which the interventions are intended to have an effect, and any theoretical underpinnings. All interventions were underpinned by theory or incorporated behaviour change techniques, the most common theory being Social Cognitive Theory(37).

Implementation and delivery approaches

Moore et al.(4) recognise that interventions can have limited effects due to weaknesses in how they are designed, or because they are not properly implemented. This section outlines the extent to which interventions were reported to be delivered as intended, common approaches used in intervention delivery, and whether this reportedly translated into changes in outcomes.

As indicated in file 5, in three studies(21-23) interventions were reportedly delivered as intended. In seven studies,(19, 20, 25, 28-30, 33) adaptations were made to the interventions during the course of the trial. In the remaining seven studies,(24, 26, 27, 31, 32, 34, 35) it was difficult to determine whether there were any adaptations as authors only reported the actual delivery, not the intended delivery.

Approaches for achieving intervention delivery included: ensuring staff were appropriately trained and prepared to deliver the intervention with fidelity(19, 31); tailoring aspects of the programme to individuals and their needs (e.g. ensuring activity consultations are appropriate for those with intellectual disabilities (20)); and allowing for flexibility in delivery methods. For example, in Poston et al.(26), pregnant women were provided with the option of receiving the intervention via phone or email, rather than sessions delivered at the

For peer review only - http://bmjopen.bmj.com/site/about/guidelines.xhtml

hospital, and in Berendsen et al.(29) coaching meetings as part of the intervention were planned with consideration of holidays and health issues.

Despite these adaptations for enhancing fidelity, interventions were not always effective in achieving the intended outcomes. For example, in Poston et al.(26) despite flexibility in the delivery mode, objectively measured physical activity and sedentary behaviour did not change in the intervention group. In this particular participant group (pregnant women), the potential to achieve the targeted health outcome, optimal blood glucose level, via dietary changes, was greater than changes in physical activity, including sedentary behaviour, as for some participants increasing their activity led to feelings of discomfort. Similarly, in Matthews et al.(20), although individual tailoring was used, the intervention did not have a significant effect on any of the primary or secondary outcomes including time spent in MVPA and time spent sedentary. It was suggested that this intervention may need to be longer than 12 weeks for individuals with intellectual disabilities. This highlights the importance of understanding more about how an intervention is intended to have an effect, as outlined in the following section.

Mechanisms of impact influencing intervention effectiveness

Moore et al.(4) emphasised the importance of exploring mechanisms through which interventions bring about change, to learn more about how the intervention effects may have occurred and how they may be replicated in similar future interventions. This section outlines the mechanisms reported across the studies and the extent to which they impacted on behaviour and outcomes.

Social Cognitive Theory was the most commonly used theory, and the following mechanisms of action were reported in several studies: enhancing self-efficacy by rating confidence in completing goals(19); using behavioural cues e.g. standing up every hour, and leaving the remote at the TV(19); using resources e.g. websites combined with counselling calls to encourage goal setting(28) providing social support in educational sessions or workshops, and input and engagement from carers(19, 20, 22, 24, 28).

However, across the studies, the extent to which these mechanisms had their intended impact on behaviour change varied. In Elramli (24) the intervention aim was increasing daily step count, social support was found to be a key factor in participants who increased their physical activity. However, behaviour change techniques including social support, feedback, and self-monitoring were to a lesser extent associated with reduced sedentary behaviour in those with rheumatoid arthritis (RA). In Matthews et al.(20), where the intervention aimed to increase walking and reduce sedentary behaviour, the social support component was not effective for adults with intellectual disabilities. In Biddle et al.(22) where the intervention aimed to reduce sitting time, there was no difference in sedentary time at 12 months between intervention and control arms. Reasons for a lack of change in sedentary behaviour included: a preference for adopting physically active behaviours rather than sitting less, and motivational drift after three months. In Adams and Gill(19) which focused on reducing sedentary behaviour and increasing light physical activity, self-efficacy was not shown to be a predictor of change in sedentary behaviour. Behavioural cues, e.g. leaving the remote at the TV, did not always influence behaviours either, because some participants were already doing the cued behaviour, and some did not have a TV(19).

Studies underpinned by the Transtheoretical Model, Theory of Planned Behaviour and Self-Determination theory placed emphasis on encouraging participants to be aware of and monitor their own behaviour(20, 29, 30). Motivational interviewing was used in two studies to prompt participants to find solutions, rather than telling them how to change their behaviour (29, 30). Berendsen et al.(29) found the feasibility of changing physical activity behaviours and dietary habits was not as high as expected and was likely associated with poor adherence. Some participants were unrealistic about how much of their own effort would be required, which influenced attendance at meetings. Lakerveld et al.(30) reported that practice nurses were competent and confident in the delivery of motivational interviewing and participants' satisfaction was high, but even so,

For peer review only - http://bmjopen.bmj.com/site/about/guidelines.xhtml

almost no effects were seen in the determinants of behaviour change in this population of individuals who were at risk of cardiovascular disease and diabetes.

In summary, these findings provide some insights into how mechanisms may or may not have an effect on sedentary behaviour, highlighting that it is important to fully understand the complexities of interventions.

Factors including context that facilitate or hinder implementation or how participants respond or interact with the intervention

Moore et al.(4) regard understanding context as an important part of interpreting factors influencing whether interventions are effective. They defined context as anything external to the intervention that may act as a barrier to its implementation or effects. They also considered participants' responses to and interactions with the intervention as important mechanisms that could influence outcomes. Drawing on the coding framework, this section is divided into include barriers and facilitators to delivery of interventions, barriers and facilitators to participation and engagement, and understanding of participants experiences from different perspectives.

Barriers to delivery of interventions

Across the studies, there were a range of barriers to delivering interventions, including administrative or scheduling issues and organisational difficulties or challenges. In two studies, planning educational sessions around other commitments including holidays and childcare responsibilities was difficult for staff (24, 34). In Blunt et al.(33) a central research team were involved in scheduling appointments, intending to reduce the workload for coaches. However, this resulted in increasing time spent scheduling and it was recommended that coaches were best placed to take responsibility for their own scheduling(33).

Organisational difficulties were apparent across two studies(20, 31). A community health worker from one of the six health centres in Benedetti et al.(31) described the long absence of a doctor as a turbulent time in the unit, which added difficulties in trying to deliver the intervention. In Matthews et al.(20), the intervention was implemented at a time of significant change within the local learning disability service. Provision of support was affected by the closure of many day centres, which led to a low morale and increasing work pressures among the staff. In Berendsen et al.(29), there were factors that influenced adherence; additionally suspended government financial and policy support meant the programme could not continue.

Barriers to participation and engagement

Across the studies, there was a range of barriers to participation and engagement in the interventions. The most common barriers to engagement were: having a pre-existing illness or injury and associated problems e.g. pain(19, 23-29, 33), having other commitments e.g. work, caring responsibilities(23, 24, 26, 28); and being too tired(22, 26, 33). Other, less common barriers to engagement included loss of accountability for behaviour over time(33), fluctuating mental health(21), and lack of motivation(24).

Some participants also experienced difficulties with pedometers and accelerometers used as an outcome measure for the trial., in terms of understanding how to use them, side effects of wearing them e.g. skin irritation(19, 23) and lost devices(19, 22). In Biddle et al.(22) half the participants experienced problems with the software for the 'Gruve' accelerometer, including: computer synchronisation issues, incompatible computers, website navigation problems, device malfunction, short battery life, and charging issues.

Some barriers may be more applicable to specific groups. For example, in Benedetti et al.(31), a community health worker perceived some older people to be apprehensive about new things which may have been a barrier to participation. In another study, a participant thought that sitting was deserved in old age and he was

For peer review only - http://bmjopen.bmj.com/site/about/guidelines.xhtml

looking forward to this aspect of retirement to indulge in some of his passions e.g. reading and studying, which made him resent the idea of standing more(23).

Some barriers were specific to particular contexts. In Elramli,(24) participants who had RA worried about using the gym because they lacked knowledge of suitable, safe exercises. Although workplace interventions were not included in this review, participants who had received educational based interventions reflected on how this applied to other parts of their lives and therefore provided some insight into how the work setting impacts upon sedentariness. For example, participants felt that it was not appropriate to be standing in a work context which could cause embarrassment, e.g. the expectation to be seated for meetings(19, 22, 23). Further barriers at work included having no access to stairs and no standing desks(22).

The context of other parts of everyday life was also influential for some participants who had developed ingrained sedentary habits, as a result of their usual activities or hobbies e.g. reading, eating, socialising, TV viewing, and knitting(23). Religious festivals had an impact on willingness to reduce sitting time at certain times of the year e.g. Christmas and Ramadan(25).

Facilitators to the delivery of interventions

Some of the approaches for achieving implementation and delivery could be regarded as facilitators, including: allowing flexibility in delivery methods, tailoring aspects of the programme to individuals, initial preparation and planning. A range of other factors facilitated intervention delivery.

For example, in Blunt et al.(33), coaches valued the simplicity and structure of the programme. They also appreciated that the programme did not require extensive background knowledge or preparation over and above their existing working requirements. Coaches had the option of referring back to the Canadian Physical Activity Guidelines to ensure they were providing the right level of support to participants. In another study, not requiring too much additional trial focused expertise, and having access to useful trial related resources was valued by social workers(34). In this study the research team prepared and organised most of the materials which facilitated delivery. As a contrast to low morale among staff(20), having a committed team was also important for facilitating delivery(34).

Facilitators to participation or engagement in intervention

There were a range of facilitators to participation and engagement in the interventions. The most common facilitator was support and encouragement from providers and peers; participants valued personal interaction and having someone to keep them on track with the intervention(20, 24, 25, 27, 31, 33).

In some studies, group environments facilitated engagement and provided opportunities for sharing experiences and meeting other peers in a similar situation(21, 24, 27). In Matthews et al.(20), many participants liked one-to-one engagement with intervention providers. This was particularly beneficial to the group who had intellectual disabilities, partly because the conflicting needs of participants in group activities were occasionally disruptive. This group faced challenges to engagement with the intervention, compared to the general population. Matthews et al. suggested the need for providing interventions to people with intellectual disabilities for longer than 12 weeks, so that consultations with providers can address more barriers(20).

Being accountable to someone, e.g. a health coach, also facilitated engagement in three studies because the participants felt being monitored provided motivation(20, 23, 25, 33). Whilst use of a step count monitor was a barrier for some, others found this was a good motivator(23, 24). Adams and Gill(19) recommended that in order for pedometers to be beneficial they need to be more accurate. It was also suggested that technology should be tailored to detect movement in older adults which may be different from younger adults (23).

For peer review only - http://bmjopen.bmj.com/site/about/guidelines.xhtml

Participants valued textual resources that were considered attractive through using appropriate text and images (20, 31). Adams and Gill(19) made recommendations for making resources more accessible including embedding videos in emails rather than asking participants to use YouTube, and printing cue cards out rather than asking participants to do so themselves. Less common facilitators were: already being involved in health programmes (33), and becoming more aware of the extent of their own sedentary behaviour(23).

Understanding experiences of interventions from different perspectives

Participants

There was some overlap in data coded into barriers and facilitators and participant experiences. The experiences can be divided into positive and negative. Examples of common positive experiences included enjoyment or satisfaction with the intervention programme (19, 21, 31). In some studies, participants described this as life-changing(23, 25) or a new opportunity for learning about how to reduce sedentary behaviour and exercise safely(24). As a result of engaging in the intervention, some participants recognised they had become more aware of the importance of reducing sedentary behaviour(19, 24, 31) and associated benefits e.g. weight loss(21, 23), and reduced stress(23, 34),less fatigue(23), less pain(24), and lower blood sugar(19).

Examples of negative experiences included: feeling stressed or nervous due to wearing a pedometer and a need to check it frequently(24); disliking a type of counselling session because they expected to follow suggestions(30); and feeling nagged by carers to participate(20).

Family/carers

Only two studies included data regarding the experiences of families or carers(20, 34). There was a distinction between the carers' or family members' perceptions of participants' experience and their own experiences as part of an intervention or supporting the intervention. In Matthews et al.(20) family carers talked about how much the participants enjoyed their experiences due to reaching their goals and getting a certificate.

The dynamic was different in another study which included a family-based exercise intervention(34). Participants valued reminding each other as a family to do their exercises.

Staff

There was also some overlap in data coded into barriers and facilitators and staff experiences. Most staffs' perceptions of participants' experiences were positive. In two studies, staff perceived participants enjoyed using pedometers and diaries(20, 25). Staff voiced positive perceptions of the programme, e.g. encouraging others and themselves to fit physical activities into their everyday lives(33), and enhancing the participants' family cohesiveness(34). Being involved in delivering the programme also had benefits for some staff . It helped them understand the complexities associated with having a healthy lifestyle(33); and reminded them to stand and move more in their own roles(34).

Some negative experiences overlapped with the barriers to delivering the interventions. These included difficulties with staffing when they were already overcommitted (20, 31); limited venue space for delivering the programme(31); and lack of psychological training to be able to deliver the intervention(29).

For peer review only - http://bmjopen.bmj.com/site/about/guidelines.xhtml

DISCUSSION

Summary of findings

This review aimed to synthesise process evaluations of interventions in trials where sedentary behaviour was measured as an outcome to: develop an understanding of intervention content, mechanisms of impact, implementation and delivery approaches and contexts, in which interventions were reported to be effective or ineffective and explore the experiences of participants, family/carers and intervention staff in such interventions. To address these aims, we synthesised data from 17 studies including a range of participant groups e.g. mothers or parents of infants, pregnant women, adults, older adults, overweight adults, individuals with chronic illnesses including rheumatoid arthritis, intellectual disabilities and serious mental illnesses. Systematic reviews of process evaluations have been conducted in other areas of research e.g. primary care(38) and workplace health promotion programmes(39). However, to our knowledge this review is the first to synthesise data from process evaluations of interventions in trials which measured sedentary behaviour as an outcome in adults.

The review has highlighted the complexity of factors that contribute to implementing interventions with fidelity, and how this links to outcome effects. Common barriers to delivery were those that may be expected in delivery of complex interventions of any kind, not just reducing sedentary behaviour. These included structural changes and staffing pressures within an organisation, and limited funding for providing interventions. Many interventions required some level of input from providers (e.g. researchers, health educators, exercise professionals, coaches and health professionals) to deliver the programme, e.g. scheduled exercise or education sessions. On the other hand, this limited flexibility of a structured intervention posed difficulties amongst some participants who had busy schedules and other priorities. In such cases, delivery was facilitated by providing different options for how the intervention is delivered e.g. via phone or email. However, flexible intervention delivery did not guarantee adherence to the intervention, because participants faced other barriers e.g. discomfort during pregnancy, cognitive difficulties; these factors ultimately impacted on sedentariness.

Whilst it was not our primary intention to synthesise the quantitative findings from the RCTs; the quantitative findings (summarised in supplementary file 7), indicate only three studies reported a statistically significant reduction in sedentary behaviour at the end of the intervention (21, 24, 33). The review identified commonalities across these three interventions that were effective in reducing sedentary behaviour; they all included elements of goal setting and access to support or coaching from a professional. All three were underpinned by theories (social cognitive theory of self-regulation, social cognitive theory and the COM-B model, including a focus on self-efficacy) which in part explain how these interventions may have had their effects (file 5). However other studies also had similar features, were underpinned by similar social cognitive principles including self-efficacy (19, 22, 26, 28)but reported no statistically significant reduction in sedentary behaviour. In three of these four studies, control group participants still commonly received some form of information e.g. a leaflet or workbook which could be regarded as informational support. This may account for not finding a statistically significant effect when compared to the interventions, if their mechanisms of effect are quite similar. These findings identify that the process of changing outcomes e.g. sedentary behaviour is complex and influenced by other factors, aside from intervention components.

Complex interventions were traditionally understood as those comprised of multiple components(3). However, context is becoming increasingly recognised as a source of complexity with acknowledgement that interventions are not a discrete package of components, but also a process of changing what complex systems do, including the interactions between individuals (e.g. providers and recipients)(40). Our findings support this notion because whilst all interventions were underpinned by psychological theories focused on individual-level change e.g. social cognitive theory(37), trans-theoretical model(41), theory of planned behaviour(42), self-determination theory(43) and habit formation theory(44); it was evident that a range of wider, contextual

For peer review only - http://bmjopen.bmj.com/site/about/guidelines.xhtml

factors in addition to individual factors also influenced the implementation and delivery of the intervention as part of complex systems. However, within the included process evaluations, programme theories (including logic models) depicting how the intervention would operate in a particular context were rarely reported. Only one process evaluation reported a logic model(25). Given the complex nature of the delivery and engagement associated with complex interventions, it is important that influences on outcomes such as reduced sedentary behaviour are understood as individual-level behaviour change processes, and in context, taking into account the complexities of experiences(45). Ensuring logic models are developed and reported would aid in understanding these complexities.

The identified barriers and facilitators to participation and engagement provide important insights into participants' experiences of interventions and explain what makes interventions more acceptable to some individuals compared to others. The review indicates that social support was important. Some participants valued elements of groups such as meeting others and sharing experiences among similar peers. Others, particularly those with intellectual disabilities, valued one-to one input from providers. Level of motivation was also influential in engagement. Some felt motivated due to being accountable to someone; whilst others felt motivated as a result of tracking activity using a pedometer. However, others disliked pedometers because they struggled to understand the device or experienced skin irritation whilst wearing them. Previous studies have found satisfaction being important for compliance and engagement with tracking devices e.g. pedometers(46, 47) . Results of a national cross sectional survey conducted in Australia suggested that interventions should make sure the devices align with the preferences of the target groups(48) . Our review suggests that individuals with particular conditions could benefit from interventions that are tailored to their symptoms e.g. pain, tiredness and illness.

Changes across the lifespan should also be considered so that interventions can take into account what is appropriate and acceptable for older adults. Our review findings indicate that older people may be more likely to think that sitting down is deserved, or associated with enjoyable hobbies e.g. reading. A recent review by Compernolle et al.(49) focused on older adults perceptions of sedentary behaviour similarly found that sedentariness was motivated by finding enjoyment, and comfort. Their experiences are also shaped by their capabilities, the social opportunities, and motivations in addition to societal expectations that often dictate that for older people sitting is their main mode of living.

Current lifestyles, regardless of age or other characteristics also influence the extent to which participants are likely to engage in behaviours that reduce sedentary behaviour. Our review evidence adds to, and supports findings from another review exploring qualitative experiences of participating in non-workplace interventions(9). Sedentary behaviour is further complicated by seasons and events e.g. celebrations such as Christmas or Ramadan which disrupt normal behaviour patterns, and perhaps lead to less concern with healthy behaviours, even with interventions. A systematic review of factors that influence physical activity and sedentary behaviour in ethnic minority groups in Europe also identified cultural and religious factors as influential in the extent to which individuals were sedentary(50). However, they highlighted that aside from the celebrations and events, some parts of religious activity e.g. walking to religious sites for prayers actually facilitated reduced sedentary behaviour and increased physical activity. It is possible that people from different ethnicities may also experience sedentary behaviour and physical activity differently, however it is difficult to determine based on the data available in this review given that only nine of the 17 studies reported ethnicity, and only three of those nine provided commentary on ethnicity. Albright et al. (28) identified that non-white racial or ethnic groups were less likely to meet their goals compared to white participants. Poston et al. (26) and Harris et al., (25) both included commentary on ethnicity in the context of recruitment to the trials and process evaluations. In Poston et al. (26) the process evaluation included women in urban hospitals in areas where socio-economic deprivation was high, they also highlighted that obesity rates are higher among those with lower socio-economic status, less qualifications and African and black Caribbean groups. The relatively low uptake (1 third approached for recruitment) was consistent with other studies with low uptake in

For peer review only - http://bmjopen.bmj.com/site/about/guidelines.xhtml

healthcare. Harris et al. (25) reported that participation was only 11% among adults and older adults in a socially and ethnically diverse population, with lower rates in more deprived Asian subgroups. This limited the ability to investigate differential effects in important subgroups. These authors have not drawn firm conclusions about how ethnicity and race may effect outcomes but Harris et al. (25) highlighted that differential uptake of interventions that are found to be successful in trials could lead to increases in inequalities in physical activity levels so this needs to be monitored.

Looking across the barriers and facilitators identified in this review and the wider literature, a range of factors need to be considered, highlighting how difficult it is to develop interventions that are suitable for participants, even those with apparently similar characteristics. The Consolidated Framework for Implementation Research (CFIR) is an example of a taxonomy of constructs, organised into five domains (intervention, inner setting, outer setting, individual characteristics, and process) that has been devised to understand what influences implementation that could be applied to further understand such complexities(51). Interventions require some level of adaptation to the context and may need to be tailored to participants, including those share similar characteristics, e.g. those with rheumatoid arthritis or intellectual disabilities. They also need to consider the dynamic between staff, participants and families as part of working towards a shared goal (e.g. reducing sedentary behaviour).

Proctor et al., (52) outlined a conceptual framework to understand interrelated outcomes in implementation research including: 1) implementation outcomes e.g. appropriateness, sustainability and costs; 2) service outcomes e.g. safety and timelines; and 3) client outcomes e.g. satisfaction. This is another example of a framework which incorporates outcomes that are not already included in the MRC framework for process evaluations. This framework could be applied as part of understanding the complex dynamics of implementing and tailoring interventions and would assist in highlighting some of the challenges associated with tailoring interventions e.g. material and staffing resource limitations, and what might be required for sustainability.

Based on the current findings, if we are to reach a point where reducing sedentary behaviour becomes habitual once interventions cease, participants will need simple strategies and support to take ownership of their own behaviour so they can sustain the lifestyle changes within the context of their lives and their preferences.

Strengths and limitations

This is the first systematic review to synthesise data from process evaluations evaluating interventions in trials that measure sedentary behaviour as an outcome in adults. Robust methods were used throughout the conduct of the review. A comprehensive search strategy was developed with input from an information specialist; two reviewers independently screened search results and assessed the quality of included studies.

Although a large proportion of the trials on which the process evaluations were based were conducted in the UK, the inclusion of studies from other countries (e.g. USA, Netherlands, Brazil, and Hong Kong) mean these findings are relevant for researchers internationally. The inclusion of males and females enhances the applicability of the findings in terms of gender. However, with regards to age, the majority of studies included participants between 40 and 50 years; therefore not all findings are applicable to other age groups. The inclusion of participants from various groups can be regarded as both a strength and limitation of this review. Findings may be of interest to experts in different research areas; however, it is difficult to draw firm conclusions for particular population groups, especially where sample sizes are small.

There was an overall lack of consistency in how process evaluations were reported, this was also the case in a review of process evaluations in primary care (38). Fourteen out of 17 used the term ‘process evaluation’ within the publication. Three did not use this term(23, 24, 34), although they met the criteria for inclusion in

For peer review only - http://bmjopen.bmj.com/site/about/guidelines.xhtml

that they aimed to explore participants' views on the factors that influence intervention effectiveness (24, 34), including the feasibility and acceptability of the intervention.

The assessments using the MMAT also indicated some variation in the quality of the process evaluations. The four studies that were considered lowest quality had poorer qualitative components(21, 26, 27, 34) that lacked detail and depth, and had limited interpretation. When studies were rated negatively on the qualitative component, it was reflected in the judgement in the mixed methods category in the MMAT. Only four studies(20, 22, 25, 27) cited the MRC guidance for process evaluations (4) but this did not always equate to better quality. Only one study by Harris (25) used the framework to guide the evaluation, whereas the other three only made reference to it in the introduction. Harris et al. (25) was one of the higher quality studies overall, suggesting that using a framework to guide the whole process evaluation can be beneficial. However, the quality of the other studies that included frameworks such as RE-AIM (36) and Steckler and Linnan's (53) process evaluation framework was variable.

Figure 2 indicates that the studies reported a lot of data about the factors including context that influence implementation and how participants respond or interact with the intervention. However, only one process evaluation included a logic model outlining how the intervention intended to have an effect (25). This means the theoretical understandings are more limited, making learning from previous evaluations more difficult. The importance of programme theories and logic models have been emphasised in recent MRC guidance (54), researchers should incorporate this in future evaluations of complex interventions.

More than 24 tools are available to assess the quality of systematic reviews; however, there remains no clear guidance for which tool to use for assessing the quality of process evaluations (55). The MMAT(18) was a logical choice as it is appropriate for mixed methods studies and those using either qualitative or quantitative data. However, it has not been designed to require detailed commentary about judgements of quality. Therefore a simplified account of quality is presented. Yet, it is difficult to compare studies without looking across all the domains because the authors do not recommend calculating an overall score(18). It was also recommended that studies should not be excluded based on their quality(18), accordingly all studies were included in the synthesis. In our view there is also a need to develop guidelines specific to systematically reviewing process evaluations of complex interventions.

The initial searches for this review were conducted in May 2019 and were repeated in May 2020. We acknowledge that this area of research is experiencing considerable growth in numbers of publications. Studies published since May 2020 were not included in the current synthesis. Recognising this limitation, we repeated the searches in October 2021 using the same parameters as previously. We have presented these new searches in supplementary file 10.

Overall 464 unique articles were identified once 14 duplicates were removed. Two reviewers completed title and abstract screening identifiying 29 for full text screening; of these, 21 met our criteria, eight are ongoing studies (56-63), eight are completed trials where a process evaluation was conducted but results are not available(64-71), and five are completed studies with process evaluation results available (72-76). As with the studies that were synthesised in our review, these included participants from a range of different ages and health conditions e.g. insomnia disorder, diabetes, heart disease, hip fracture, and obesity and generally focused on increasing physical activity, reducing sedentary behaviour or were lifestyle or weight loss interventions.

Of the five eligible studies where process evaluation results are available, one study (72) was guided by the MRC framework (4), none of the other studies used this or other frameworks to guide their evaluation. This study by Blackburn et al. (2021) was the only one where the intervention (SITLESS) aimed to reduce sedentary behaviour in addition to increasing physical activity and physical function. The other four included a measure of sedentary behaviour but the intervention primarily aimed to increase physical activity (75, 76)or promote

For peer review only - http://bmjopen.bmj.com/site/about/guidelines.xhtml

lifestyle changes including weight loss (73, 74). These process evaluations have different aims, one explored older adults experiences of an intervention (SITLESS) which combined an exercise referral scheme plus self-management strategies (72), one explored factors that support older people to increase their physical activity levels in a primary care based intervention (PACE-Lift) (76); one explored how participants of different ages with a range of conditions experienced and engaged with the e-coachER intervention which combined support and an exercise referral scheme (75); one focused on the feasibility and satisfaction of an email lifestyle intervention aimed at minority breast cancer survivors (74); another explored engagement and compliance with a community weight loss intervention for obese males (SHED-IT) (73).

The findings from our updated search demonstrate the growing literature on testing and evaluating interventions including understand the factors that influence experiences, engagement, compliance, satisfaction and how interventions are implemented. Whilst the reported findings of these studies appear to be largely consistent with those included in our narrative synthesis, the iterative nature of coding data into the framework that was undertaken as part of this process means that it would not be appropriate to attempt to merge these findings into our already completed analysis. However, it is important to be aware of these recent studies when considering factors that influence how interventions focused on reducing sedentary behaviour are implemented, and how they are experienced.

CONCLUSIONS

There is a wealth of existing evidence which synthesises the findings from trials evaluating interventions that have measured sedentary behaviour as an outcome in adults. This review complements existing trial evidence because it highlights a range of factors associated with implementation, context, and participants experiences that can impact on whether an intervention is effective or not.

It is promising that all interventions were underpinned by theory as part of understanding how they were intended to have an effect, however it is important to acknowledge how different contexts and individual level factors e.g. health status, illness, age, and lifestyles can shape levels of engagement and behaviour change. Researchers could benefit from using a process evaluation framework such as Moore et al's,(4) for conducting and reporting process evaluations to ensure all factors are considered. Including logic model as part of the process evaluation would also assist in mapping the range of factors that contribute to changes in intervention outcomes.

FIGURES:

Figure 1: PRISMA flow diagram
Figure 2: Key findings mapped to the diagram from the MRC guidance for process evaluations

ADDITIONAL

Acknowledgements: We acknowledge the help and support of our Information Scientist, Deirdre Andre, University of Leeds. We also thank Dr Rekesh Corepal (RC) for his contributions when the original search was conducted in 2019. We are grateful for the funding provided by the National Institute for Health Research (NIHR).

Author Contributions: This systematic review was conceived and designed by members of the RECREATE Programme Management Group (AF, CE, CF, RL, DJC) and researchers (JFJ, NL, JH, SM). The systematic review process was conducted by JFJ, NL, SO with oversight and input from DJC. JFJ drafted the initial manuscript with

For peer review only - http://bmjopen.bmj.com/site/about/guidelines.xhtml

input from NL and DJC. All authors have critically reviewed and revised different versions of the manuscript (JFJ, NL, SO, JH, SM, CE, CF, RL, AF, DJC).

Competing interests: None declared

Funding: This report is independent research funded by the National Institute for Health Research (Programme Grants for Applied Research, Development and evaluation of strategies to reduce sedentary behaviour in patients after stroke and improve outcomes, RP-PG-0615-20019).

Data availability statement: All data relevant to the study are included in the article or uploaded as supplementary information

Patient consent for publication: Not required

Ethics approval: None required as this is a systematic review which synthesises data from previously published research.

For peer review only

For peer review only - http://bmjopen.bmj.com/site/about/guidelines.xhtml

REFERENCES

1. Tremblay MS, Aubert S, Barnes JD, Saunders TJ, Carson V, Latimer-Cheung AE, et al. Sedentary behavior research network (SBRN)–terminology consensus project process and outcome. International Journal of Behavioral Nutrition and Physical Activity. 2017;14(1):75.
2. Owen N, Healy GN, Matthews CE, Dunstan DW. Too much sitting: the population-health science of sedentary behavior. Exercise and sport sciences reviews. 2010;38(3):105.
3. Craig P, Dieppe P, Macintyre S, Michie S, Nazareth I, Petticrew M. Developing and evaluating complex interventions: the new Medical Research Council guidance. 2013.
4. Moore GF, Audrey S, Barker M, Bond L, Bonell C, Hardeman W, et al. Process evaluation of complex interventions: Medical Research Council guidance. bmj. 2015;350.
5. Petticrew M. When are complex interventions 'complex'? When are simple interventions 'simple'? : Oxford University Press; 2011.
6. Shepperd S, Lewin S, Straus S, Clarke M, Eccles MP, Fitzpatrick R, et al. Can we systematically review studies that evaluate complex interventions? PLoS Med. 2009;6(8):e1000086.
7. Prince S, Saunders T, Gresty K, Reid R. A comparison of the effectiveness of physical activity and sedentary behaviour interventions in reducing sedentary time in adults: a systematic review and meta-analysis of controlled trials. Obesity Reviews. 2014;15(11):905-19.
8. Shrestha N, Grgic J, Wiesner G, Parker A, Podnar H, Bennie JA, et al. Effectiveness of interventions for reducing non-occupational sedentary behaviour in adults and older adults: a systematic review and meta-analysis. British journal of sports medicine. 2019;53(19):1206-13.
9. Rawlings G, Williams R, Clarke D, English C, Fitzsimons C, Holloway I, et al. Exploring adults' experiences of sedentary behaviour and participation in non-workplace interventions designed to reduce sedentary behaviour: a thematic synthesis of qualitative studies. BMC public health. 2019;19(1):1099.
10. Moher D, Shamseer L, Clarke M, Ghersi D, Liberati A, Petticrew M, et al. Preferred reporting items for systematic review and meta-analysis protocols (PRISMA-P) 2015 statement. Systematic reviews. 2015;4(1):1.
11. Corepal R, Hall JF, English C, Farrin A, Fitzsimons CF, Forster A, et al. A protocol for a systematic review of process evaluations of interventions investigating sedentary behaviour in adults. BMJ open. 2019;9(9):e031291.
12. Moore G, Audrey S, Barker M, Bond L, Bonell C, Hardeman W, et al. Process evaluation of complex interventions: UK Medical Research Council (MRC) guidance. London: MRC. 2014.
13. Hall J.F, Corepal R, Crocker T.F, Lam N, L B, Birch K, et al. A systematic review and meta-analysis of non-workplace interventions to reduce time spent sedentary in adults. 2021 Submitted to medRxiv. https://www.medrxiv.org/2021.
14. Robbins LB, Ling J, Toruner EK, Bourne KA, Pfeiffer KA. Examining reach, dose, and fidelity of the "Girls on the Move" after-school physical activity club: A process evaluation. BMC public health. 2016;16(1):671.
15. Walton H, Spector A, Williamson M, Tombor I, Michie S. Developing quality fidelity and engagement measures for complex health interventions. British journal of health psychology. 2020;25(1):39-60.
16. International Q. NVivo Qualitative Data Analysis Software (Version 12). . 2019.
17. Lincoln YS, Guba EG. But is it rigorous? Trustworthiness and authenticity in naturalistic evaluation. New directions for program evaluation. 1986;1986(30):73-84.
18. Hong QN, Pluye P, Fàbregues S, Bartlett G, Boardman F, Cargo M, et al. Mixed methods appraisal tool (MMAT), version 2018. Registration of copyright. 2018;1148552.
19. Adams MM, Gill DL. On our feet: Feasibility trial of an intervention to reduce sedentary behavior and increase physical activity. 2012(3511147):332.
20. Matthews L, Mitchell F, Stalker K, McConnachie A, Murray H, Melling C, et al. Process evaluation of the Walk Well study: a cluster-randomised controlled trial of a community based walking programme for adults with intellectual disabilities. BMC Public Health. 2016;16:527.
21. Williams J, Stubbs B, Richardson S, Flower C, Barr-Hamilton L, Grey B, et al. 'Walk this way': results from a pilot randomised controlled trial of a health coaching intervention to reduce sedentary behaviour and increase physical activity in people with serious mental illness. BMC Psychiatry. 2019;19(1):287.
22. Biddle SJ, Edwardson CL, Gorely T, Wilmot EG, Yates T, Nimmo MA, et al. Reducing sedentary time in adults at risk of type 2 diabetes: process evaluation of the STAND (Sedentary Time ANd Diabetes) RCT. BMC Public Health. 2017;17(1):80.

For peer review only - http://bmjopen.bmj.com/site/about/guidelines.xhtml

23. Matson TE, Renz AD, Takemoto ML, McClure JB, Rosenberg DE. Acceptability of a sitting reduction intervention for older adults with obesity. BMC public health. 2018;18(1):706-.
24. Elramli A. Effectiveness of community based physical activity on step count and sedentary behaviour in people with rheumatoid arthritis within the first five years of diagnosis [PhD]. Glasgow: University of Glasgow; 2017.
25. Harris TK, S; Victor, C; Iliffe, S; Ussher, M; Fox-Rushby, J; Whincup, P; Ekelund, U; Furness, C; Limb, E; Anokye, N; Ibison, J; DeWilde, S; David, L; Howard, E; Dale, R; Smith, J; Normansell, R; Beighton, C; Morgan, K; Wahlich, C; Sanghera, S; Cook, D. A pedometer-based walking intervention in 45- to 75-year-olds, with and without practice nurse support: the PACE-UP three-arm cluster RCT. ASSESSMENT HT; 2018. Contract No.: ISSN 1366-5278.
26. Poston L, Briley AL, Barr S, Bell R, Croker H, Coxon K, et al. Developing a complex intervention for diet and activity behaviour change in obese pregnant women (the UPBEAT trial); assessment of behavioural change and process evaluation in a pilot randomised controlled trial. BMC Pregnancy Childbirth. 2013;13:148.
27. Stathi A, Withall J, Thompson JL, Davis MG, Gray S, De Koning J, et al. Feasibility Trial Evaluation of a Peer Volunteering Active Aging Intervention: ACE (Active, Connected, Engaged). Gerontologist. 2019.
28. Albright CL, Saiki K, Steffen AD, Woekel E. What barriers thwart postpartum women's physical activity goals during a 12-month intervention? A process evaluation of the Na Mikimiki Project. Women Health. 2015;55(1):1-21.
29. Berendsen BA, Kremers SP, Savelberg HH, Schaper NC, Hendriks MR. The implementation and sustainability of a combined lifestyle intervention in primary care: mixed method process evaluation. BMC Family Practice. 2015;16:37.
30. Lakerveld J, Bot S, Chinapaw M, van Tulder M, Kingo L, Nijpels G. Process evaluation of a lifestyle intervention to prevent diabetes and cardiovascular diseases in primary care. Health Promot Pract. 2012;13(5):696-706.
31. Benedetti TRB, Rech CR, Konrad LM, Almeida FA, Brito FA, Chodzko-Zajko W, et al. Re-thinking Physical Activity Programs for Older Brazilians and the Role of Public Health Centers: A Randomized Controlled Trial Using the RE-AIM Model. Front Public Health. 2020;8:48-.
32. Lane A, Murphy N, Bauman A, Chey T. Randomized controlled trial to increase physical activity among insufficiently active women following their participation in a mass event. Health education journal. 2010;69(3):287-96.
33. Blunt W, Gill DP, Riggin B, Brown JB, Petrella RJ. Process evaluation of the HealtheStepsTM lifestyle prescription program. Translational behavioral medicine. 2019;9(1):32-40.
34. School of Public Health TUoHK. Be Healthy, So Easy: Family Education Project. Hong Kong: The University of Hong Kong; 2017.
35. Spittaels H, De Bourdeaudhuij I, Brug J, Vandelanotte C. Effectiveness of an online computer-tailored physical activity intervention in a real-life setting. Health Education Research. 2007;22(3):385-96.
36. Glasgow RE, Vogt TM, Boles SM. Evaluating the public health impact of health promotion interventions: the RE-AIM framework. American journal of public health. 1999;89(9):1322-7.
37. Bandura A. Social foundations of thought and action. Englewood Cliffs, NJ. 1986;1986(23-28).
38. Liu H, Mohammed A, Shanthosh J, Laba T-L, Hackett ML, Peiris D, et al. Process evaluations of primary care interventions addressing chronic disease: a systematic review. BMJ open. 2019;9(8):e025127.
39. Wierenga D, Engbers LH, Van Empelen P, Duijts S, Hildebrandt VH, Van Mechelen W. What is actually measured in process evaluations for worksite health promotion programs: a systematic review. BMC public health. 2013;13(1):1190.
40. Moore GF, Evans RE, Hawkins J, Littlecott H, Melendez-Torres G, Bonell C, et al. From complex social interventions to interventions in complex social systems: future directions and unresolved questions for intervention development and evaluation. Evaluation. 2019;25(1):23-45.
41. Prochaska JO, Marcus BH. The transtheoretical model: Applications to exercise. 1994.
42. Ajzen I. From intentions to actions: A theory of planned behavior. Action control: Springer; 1985. p. 11-39.
43. Ryan RM, Deci EL. Self-determination theory and the facilitation of intrinsic motivation, social development, and well-being. American psychologist. 2000;55(1):68.
44. Lally P, Wardle J, Gardner B. Experiences of habit formation: a qualitative study. Psychology, health & medicine. 2011;16(4):484-9.
45. Moore GF, Evans RE, Hawkins J, Littlecott HJ, Turley R. All interventions are complex, but some are more complex than others: using iCAT_SR to assess complexity. Cochrane Database of Systematic Reviews. 2017(7).

For peer review only - http://bmjopen.bmj.com/site/about/guidelines.xhtml

46. Fairclough S, Noonan R, Rowlands A, Van Hees V, Knowles Z, Boddy L. Wear compliance and activity in children wearing wrist and hip mounted accelerometers. Medicine & Science in Sports & Exercise. 2016;48(2):245-53.
47. Hassani M, Kivimaki M, Elbaz A, Shipley M, Singh-Manoux A, Sabia S. Non-consent to a wrist-worn accelerometer in older adults: the role of socio-demographic, behavioural and health factors. PLoS One. 2014;9(10):e110816.
48. Alley S, Schoeppe S, Guertler D, Jennings C, Duncan MJ, Vandelanotte C. Interest and preferences for using advanced physical activity tracking devices: results of a national cross-sectional survey. BMJ open. 2016;6(7):e011243.
49. Compernolle S, De Cocker K, Cardon G, De Bourdeaudhuij I, Van Dyck D. Older adults' perceptions of sedentary behavior: A systematic review and thematic synthesis of qualitative studies. The Gerontologist. 2020;60(8):e572-e82.
50. Langøien LJ, Terragni L, Rugseth G, Nicolaou M, Holdsworth M, Stronks K, et al. Systematic mapping review of the factors influencing physical activity and sedentary behaviour in ethnic minority groups in Europe: a DEDIPAC study. International Journal of Behavioral Nutrition and Physical Activity. 2017;14(1):1-24.
51. Damschroder LJ, Aron DC, Keith RE, Kirsh SR, Alexander JA, Lowery JC. Fostering implementation of health services research findings into practice: a consolidated framework for advancing implementation science. Implementation science. 2009;4(1):1-15.
52. Proctor E, Silmere H, Raghavan R, Hovmand P, Aarons G, Bunger A, et al. Outcomes for implementation research: conceptual distinctions, measurement challenges, and research agenda. Administration and policy in mental health and mental health services research. 2011;38(2):65-76.
53. Linnan L, Steckler A. Process evaluation for public health interventions and research. 2002.
54. Skivington K, Matthews L, Craig P, Simpson S, Moore L. Developing and evaluating complex interventions: updating Medical Research Council guidance to take account of new methodological and theoretical approaches. The Lancet. 2018;392:S2.
55. Shea B, Dubé C, Moher D. Assessing the quality of reports of systematic reviews: the QUOROM statement compared to other tools. Systematic Reviews in Health Care: Meta-Analysis in Context. 2001:122-39.
56. Bailey DP, Edwardson CL, Pappas Y, Dong F, Hewson DJ, Biddle SJH, et al. A randomised-controlled feasibility study of the REgulate your SItting Time (RESIT) intervention for reducing sitting time in individuals with type 2 diabetes: study protocol. Pilot & Feasibility Studies. 2021;7(1):76.
57. Cheung NW, Redfern J, Thiagalingam A, Hng T-M, Islam SMS, Haider R, et al. Text messaging support for patients with diabetes or coronary artery disease (SupportMe): protocol for a pragmatic randomised controlled trial. BMJ Open. 2019;9(6):e025923.
58. ISRCTN82280581. Reducing sedentary behaviour after stroke. http://wwwwhoint/trialsearch/Trial2aspx?TrialID=ISRCTN82280581. 2020.
59. McGregor G, Sandhu H, Bruce J, Sheehan B, McWilliams D, Yeung J, et al. Rehabilitation Exercise and psycholoGical support After covid-19 InfectioN' (REGAIN): a structured summary of a study protocol for a randomised controlled trial. Trials. 2021;22(1).
60. NL9263. Sitting interruption treatment as a personalized secondary prevention strategy in patients with coronary artery disease: a randomized clinical trial. http://wwwwhoint/trialsearch/Trial2aspx?TrialID=NL9263. 2021.
61. Roland CB, Knudsen SP, Alomairah SA, Andersen AD, Bendix J, Clausen TD, et al. Structured supervised exercise training or motivational counselling during pregnancy on physical activity level and health of mother and offspring: FitMum study protocol. BMJ Open. 2021;11(3):e043671.
62. Sharman MJ, Ball K, Greaves S, Jose KA, Morse M, Blizzard CL, et al. trips4health: Protocol of a single-blinded randomised controlled trial incentivising adults to use public transport for physical activity gain. Contemporary Clinical Trials Communications. 2020;19:100619.
63. Taylor NF, O'Halloran PD, Watts JJ, Morris R, Peiris CL, Porter J, et al. Motivational interviewing with community-dwelling older adults after hip fracture (MIHip): Protocol for a randomised controlled trial. BMJ Open. 2021;11(6) (no pagination).
64. Halse RE, Shoneye CL, Pollard CM, Jancey J, Scott JA, Pratt IS, et al. Improving Nutrition and Activity Behaviors Using Digital Technology and Tailored Feedback: Protocol for the LiveLighter Tailored Diet and Activity (ToDAy) Randomized Controlled Trial. JMIR Res Protoc. 2019;8(2):e12782.
65. Høj K, Skriver MV, Hansen A-LS, Christensen B, Maindal HT, Sandbæk A. Effect of including fitness testing in preventive health checks on cardiorespiratory fitness and motivation: study protocol of a randomized controlled trial. BMC Public Health. 2014;14(1):1057.

For peer review only - http://bmjopen.bmj.com/site/about/guidelines.xhtml

66. Murawski B, Plotnikoff RC, Rayward AT, Vandelanotte C, Brown WJ, Duncan MJ. Randomised controlled trial using a theory-based m-health intervention to improve physical activity and sleep health in adults: the Synergy Study protocol. BMJ Open. 2018;8(2):e018997.
67. O'Regan A, Glynn L, Garcia Bengoechea E, Casey M, Clifford A, Donnelly A, et al. An evaluation of an intervention designed to help inactive adults become more active with a peer mentoring component: a protocol for a cluster randomised feasibility trial of the Move for Life programme. Pilot and Feasibility Studies. 2019;5(1):88.
68. Sandal LF, Bach K, Overas CK, Svendsen MJ, Dalager T, Stejnicher Drongstrup Jensen J, et al. Effectiveness of App-Delivered, Tailored Self-management Support for Adults With Lower Back Pain-Related Disability: A selfBACK Randomized Clinical Trial. JAMA Internal Medicine. 2021;181(10):1288-96.
69. Scott SE, Duarte C, Encantado J, Evans EH, Harjumaa M, Heitmann BL, et al. The NoHoW protocol: a multicentre 2×2 factorial randomised controlled trial investigating an evidence-based digital toolkit for weight loss maintenance in European adults. BMJ Open. 2019;9(9):e029425.
70. Williams LT, Hollis JL, Collins CE, Morgan PJ. The 40-Something randomized controlled trial to prevent weight gain in mid-age women. BMC Public Health. 2013;13(1):1007.
71. Young MD, Collins CE, Callister R, Plotnikoff RC, Doran CM, Morgan PJ. The SHED-IT Weight Loss Maintenance trial protocol: A randomised controlled trial of a weight loss maintenance program for overweight and obese men. Contemporary Clinical Trials. 2014;37(1):84-97.
72. Blackburn NE, Skjodt M, Tully MA, Mc Mullan I, Giné-Garriga M, Caserotti P, et al. Older Adults' Experiences of a Physical Activity and Sedentary Behaviour Intervention: A Nested Qualitative Study in the SITLESS Multi-Country Randomised Clinical Trial. International Journal of Environmental Research and Public Health. 2021;18(9).
73. Morgan PJ, Scott HA, Young MD, Plotnikoff RC, Collins CE, Callister R. Associations between program outcomes and adherence to Social Cognitive Theory tasks: process evaluation of the SHED-IT community weight loss trial for men. International Journal of Behavioral Nutrition and Physical Activity. 2014;11(1):89.
74. Paxton RJ, Hajek R, Newcomb P, Dobhal M, Borra S, Taylor WC, et al. A Lifestyle Intervention via Email in Minority Breast Cancer Survivors: Randomized Parallel-Group Feasibility Study. JMIR Cancer. 2017;3(2):e13.
75. Taylor AH, Taylor RS, Ingram WM, Anokye N, Dean S, Jolly K, et al. Adding web-based behavioural support to exercise referral schemes for inactive adults with chronic health conditions: the e-coachER RCT. Health Technology Assessment (Winchester, England). 2020;24(63):1-106.
76. Victor CR, Rogers A, Woodcock A, Beighton C, Cook DG, Kerry SM, et al. What factors support older people to increase their physical activity levels? An exploratory analysis of the experiences of PACE-Lift trial participants. Arch Gerontol Geriatr. 2016;67:1-6.

For peer review only - http://bmjopen.bmj.com/site/about/guidelines.xhtml

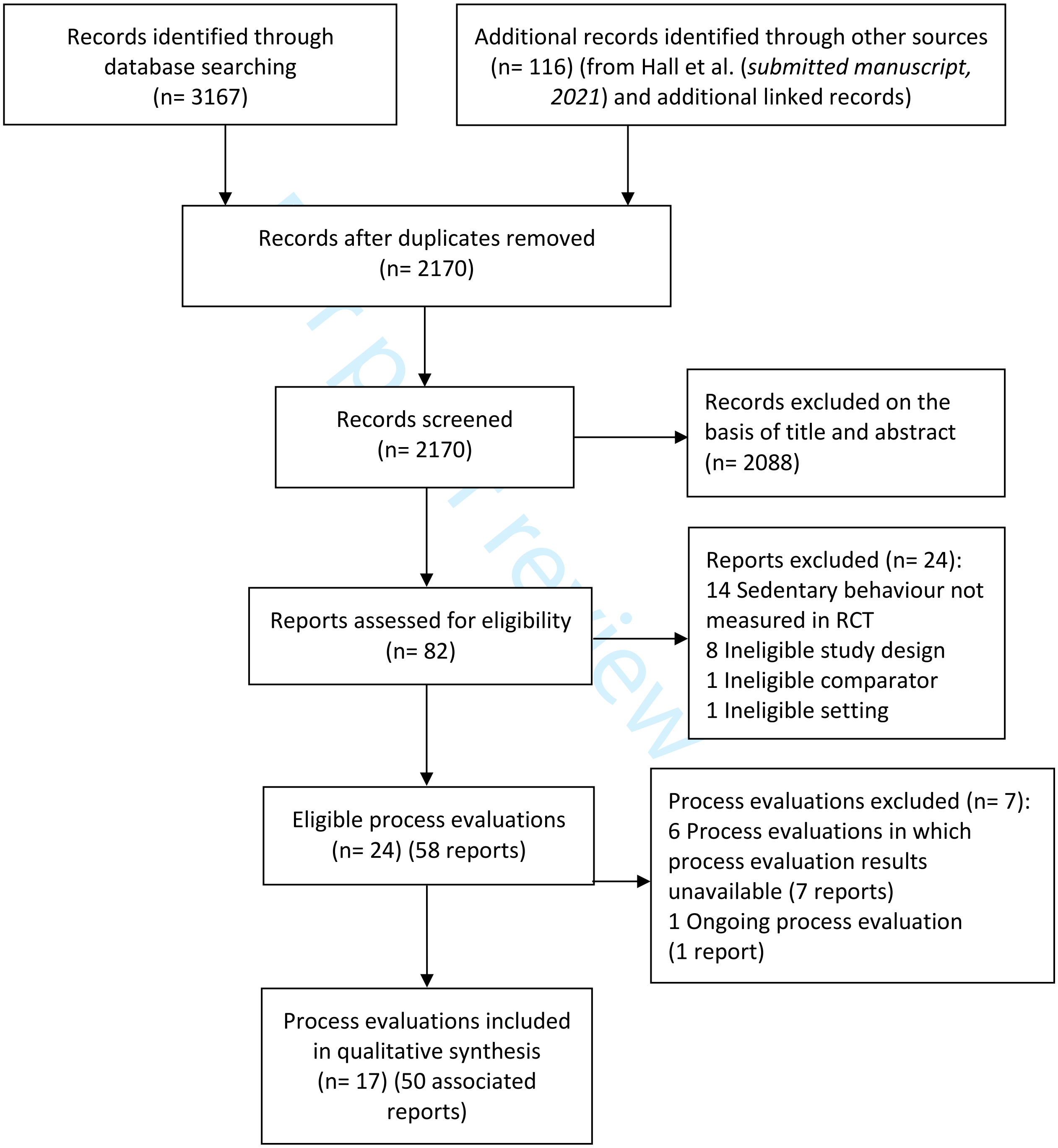
Records identified through database searching (n= 3167)
Additional records identified through other sources (n= 116) (from Hall et al. (*submitted manuscript, 2021*) and additional linked records)
Records after duplicates removed (n= 2170)
Records screened (n= 2170)
Records excluded on the basis of title and abstract (n= 2088)
Reports assessed for eligibility (n= 82)
Reports excluded (n= 24):
14 Sedentary behaviour not measured in RCT
8 Ineligible study design
1 Ineligible comparator
1 Ineligible setting
Eligible process evaluations (n= 24) (58 reports)
Process evaluations excluded (n= 7):
6 Process evaluations in which process evaluation results unavailable (7 reports)
1 Ongoing process evaluation (1 report)
Process evaluations included in qualitative synthesis (n= 17) (50 associated reports)

Factors influencing context that facilitate or hinder implementation or how participants respond or interact with the intervention (Review objectives 5 & 6)

- **Barriers to delivery of interventions:** administrative or scheduling issues, organisational challenges, absence of staff , closure of services, work pressures for staff, financial **difficulties**
- **Barriers to participation and engagement:** most common examples included: pre-existing illness or injury e.g. pain, other commitments e.g. work or caring responsibilities, being too tired. Other examples included difficulties using accelerometers or pedometers, technology problems, context specific expectations or norms, being worried about specific environments e.g. gyms due to safety risks, influence of usual everyday life routines, religious festivals at certain times of year
- **Facilitators to the delivery of interventions:** simplicity in structure of the programme, little need for preparation in addition to usual working requirements, having access to trial related resources, having a committed team
- **Facilitators to participation and engagement:** most common example- support and encouragement from providers and peers to keep on track – mixed preference for one to one vs face to face interactions. Other examples- being accountable to someone, having accurate step monitors, access to textual resources
- **Experiences of interventions from different perspectives:** positive experiences included opportunities for learning (participants and staff), becoming more aware of sedentary behaviour (participants and staff), negative experiences included disliking particular part of intervention (participants), or limited space for delivering programmes (staff)

KEY FINDING – A range of contextual and other factors influenced whether the interventions could be implemented or how participants responded. These need to be considered so that interventions can be adapted to different contexts and participant groups

Description of the interventions and their causal assumptions (review objectives 1 and 2)

- All interventions have multiple components (See supplementary file 5)
- Group based input or support common
- Delivered by a range of providers e.g. researchers, health educators, exercise professionals, coaches, advisors and nurses
- All underpinned by theory or incorporated BCTS- most common theory – Social Cognitive Theory (SCT)

KEY FINDING: Only one study included a logic model outlining causal assumptions

Implementation and delivery approaches (review objective 3)

- Interventions delivered as intended in 3 studies
- 7 interventions were adapted, 7 difficult to determine
- Approaches for achieving intervention delivery:
    - Staff training
    - Tailoring interventions to individual needs
    - Allowing for flexibility in delivery methods

KEY FINDING- enhancing fidelity with adaptations did not always lead to interventions being effective in achieving the intended outcomes. Need to understand more about the mechanisms of intended effect

Mechanisms of impact influencing intervention effectiveness (Review objectives 2 and 4)

- Mechanisms of action were reported, examples related to the most commonly used theory (SCT) included: self-efficacy, behavioural cues, using resources e.g. websites and counselling calls, social support
- Variation in how much these intended mechanisms had an effect on reducing sedentary behaviour. For example in one study, social support did not have the intended effect for those with intellectual disabilities as it was not appropriate for this population

KEY FINDING – it is important to fully understand the context and complexities associated with how interventions will work to understand more about the mechanisms of effect

Outcomes
Reducing sedentary behaviour

Figure 2: Key findings mapped to the diagram from the MRC guidance for process evaluations

Supplementary file 1: PRISMA 2020 Checklist 27.05.21

Section and Topic	Item #	Checklist item	Location where item is reported
**TITLE**			
Title	1	Identify the report as a systematic review.	Title, page 1
**ABSTRACT**			
Abstract	2	See the PRISMA 2020 for Abstracts checklist.	Pg. 1
**INTRODUCTION**			
Rationale	3	Describe the rationale for the review in the context of existing knowledge.	Pg. 2/3
Objectives	4	Provide an explicit statement of the objective(s) or question(s) the review addresses.	Pg. 3
**METHODS**			
Eligibility criteria	5	Specify the inclusion and exclusion criteria for the review and how studies were grouped for the syntheses.	Pg. 4
Information sources	6	Specify all databases, registers, websites, organisations, reference lists and other sources searched or consulted to identify studies. Specify the date when each source was last searched or consulted.	Pg. 4
Search strategy	7	Present the full search strategies for all databases, registers and websites, including any filters and limits used.	Supplementary file 2
Selection process	8	Specify the methods used to decide whether a study met the inclusion criteria of the review, including how many reviewers screened each record and each report retrieved, whether they worked independently, and if applicable, details of automation tools used in the process.	Pg. 4/5
Data collection process	9	Specify the methods used to collect data from reports, including how many reviewers collected data from each report, whether they worked independently, any processes for obtaining or confirming data from study investigators, and if applicable, details of automation tools used in the process.	Pgs. 4-7
Data items	10a	List and define all outcomes for which data were sought. Specify whether all results that were compatible with each outcome domain in each study were sought (e.g. for all measures, time points, analyses), and if not, the methods used to decide which results to collect.	n/a
	10b	List and define all other variables for which data were sought (e.g. participant and intervention characteristics, funding sources). Describe any assumptions made about any missing or unclear information.	n/a
Study risk of bias assessment	11	Specify the methods used to assess risk of bias in the included studies, including details of the tool(s) used, how many reviewers assessed each study and whether they worked independently, and if applicable, details of automation tools used in the process.	Methodological quality pg. 7
Effect measures	12	Specify for each outcome the effect measure(s) (e.g. risk ratio, mean difference) used in the synthesis or presentation of results.	n/a
Synthesis methods	13a	Describe the processes used to decide which studies were eligible for each synthesis (e.g. tabulating the study intervention characteristics and comparing against the planned groups for each synthesis (item #5)).	Pgs. 5-7
	13b	Describe any methods required to prepare the data for presentation or synthesis, such as handling of missing summary statistics, or data conversions.	n/a
	13c	Describe any methods used to tabulate or visually display results of individual studies and syntheses.	Pgs. 5-7
	13d	Describe any methods used to synthesize results and provide a rationale for the choice(s). If meta-analysis was performed, describe the model(s), method(s) to identify the presence and extent of statistical heterogeneity, and software package(s) used.	Pgs. 5-7
	13e	Describe any methods used to explore possible causes of heterogeneity among study results (e.g. subgroup analysis, meta-regression).	n/a
	13f	Describe any sensitivity analyses conducted to assess robustness of the synthesized results.	n/a
Reporting bias assessment	14	Describe any methods used to assess risk of bias due to missing results in a synthesis (arising from reporting biases).	Supplementary file 9
Certainty	15	Describe any methods used to assess certainty (or confidence) in the body of evidence for an outcome.	n/a

For peer review only - http://bmjopen.bmj.com/site/about/guidelines.xhtml

Supplementary file 1: PRISMA 2020 Checklist 27.05.21

Section and Topic	Item #	Checklist item	Location where item is reported
assessment			
**RESULTS**			
Study selection	16a	Describe the results of the search and selection process, from the number of records identified in the search to the number of studies included in the review, ideally using a flow diagram.	Pg. 9
	16b	Cite studies that might appear to meet the inclusion criteria, but which were excluded, and explain why they were excluded.	Supplementary file 4
Study characteristics	17	Cite each included study and present its characteristics.	Supplementary files 3 and 4
Risk of bias in studies	18	Present assessments of risk of bias for each included study.	Supplementary file 9
Results of individual studies	19	For all outcomes, present, for each study: (a) summary statistics for each group (where appropriate) and (b) an effect estimate and its precision (e.g. confidence/credible interval), ideally using structured tables or plots.	n/a
Results of syntheses	20a	For each synthesis, briefly summarise the characteristics and risk of bias among contributing studies.	n/a
	20b	Present results of all statistical syntheses conducted. If meta-analysis was done, present for each the summary estimate and its precision (e.g. confidence/credible interval) and measures of statistical heterogeneity. If comparing groups, describe the direction of the effect.	n/a
	20c	Present results of all investigations of possible causes of heterogeneity among study results.	n/a
	20d	Present results of all sensitivity analyses conducted to assess the robustness of the synthesized results.	n/a
Reporting biases	21	Present assessments of risk of bias due to missing results (arising from reporting biases) for each synthesis assessed.	Supplementary file 9
Certainty of evidence	22	Present assessments of certainty (or confidence) in the body of evidence for each outcome assessed.	n/a
**DISCUSSION**			
Discussion	23a	Provide a general interpretation of the results in the context of other evidence.	Pgs. 17-21
	23b	Discuss any limitations of the evidence included in the review.	Pg. 19/20
	23c	Discuss any limitations of the review processes used.	Pg. 19/20
	23d	Discuss implications of the results for practice, policy, and future research.	Pgs. 17/21
**OTHER INFORMATION**			
Registration and protocol	24a	Provide registration information for the review, including register name and registration number, or state that the review was not registered.	Pg. 2
	24b	Indicate where the review protocol can be accessed, or state that a protocol was not prepared.	Pg. 2
	24c	Describe and explain any amendments to information provided at registration or in the protocol.	n/a
Support	25	Describe sources of financial or non-financial support for the review, and the role of the funders or sponsors in the review.	Pg. 22
Competing interests	26	Declare any competing interests of review authors.	n/a
Availability of data, code and other materials	27	Report which of the following are publicly available and where they can be found: template data collection forms; data extracted from included studies; data used for all analyses; analytic code; any other materials used in the review.	Pg. 22

For peer review only

For peer review only - http://bmjopen.bmj.com/site/about/guidelines.xhtml

Supplementary file 1: PRISMA 2020 Checklist 27.05.21

From: Page MJ, McKenzie JE, Bossuyt PM, Boutron I, Hoffmann TC, Mulrow CD, et al. The PRISMA 2020 statement: an updated guideline for reporting systematic reviews. BMJ 2021;372:n71. doi: 10.1136/bmj.n71

For more information, visit: http://www.prisma-statement.org/

For peer review only

Supplementary file 2_search strategies May 11th 2020

Database: CINAHL (EBSCOhost), search modes - Boolean/Phrase, 1982-:

S1 (MH "Life Style, Sedentary")
S2 TI (sedentary or sitting or sedentariness or sedentarism)
S3 TX ((sedentary or sitting or seated) N5 (behavio* or lifestyle or life-style)))
S4 TX ((sedentary N3 (adult* or men or women or males or females or individuals or people or population*))
S5 TI (((sitting or sit or seated or stationary or standing) N3 (task* or time or bout* or work* or break*))) OR AB (((sitting or sit or seated or stationary or standing) N3 (task* or time or bout* or work* or break*)))
S6 TX((inactiv* or no exercise or nonexercise or non exercise) N3 (adult* or men or women or males or females or individuals or people))
S7 TX "low energy expenditure"
S8 TX "physical* inactiv*"
S9 TX (leisure time N5 ("physical* activ*" or passive or inactiv*)))
S10 TX "physical activity level*"
S11 TX ((sitting or lying) N2 posture*)
S12 TX (prolong* N2 (reclin* or sit or sitting or seated))
S13 TI((computer* or television or tv or video game? or videogame? or gaming) and (sedentary or "physical* activity*" or sitting or seated or underactiv* or under activ*))
S14 TX "chair rise*"
S15 TX "sit* less"
S16 TX ((light or low) N1 "physical activ*")
S17 TX ((decrease or reduc* or discourag* or lessen*) N3 (sit or sitting or stand or standing or "physical* inactiv*"))
S18 TX (time N5 (computer* or television or tv or "video game*" or videogame* or gaming or screen or media))
S19 TX ((watch* or view*) N5 (television or tv))
S20 TI (play* N5 ("video game*" or videogame* or "computer game*")) OR AB (play* N5 ("video game*" or videogame* or "computer game*"))
S21 TX allocat* random*
S22 (MH "Placebos")
S23 TX placebo*
S24 TX random* allocat*
S25 TX randomi* control* trial*
S26 TX clinic* n1 trial*
S27 PT Clinical trial
S28 (MH "Clinical Trials+")
S29 AB randomized
S30 AB randomly
S31 MH "Random Assignment"
S32 S21 or S22 or S23 or S24 or S25 or S26 or S27 or S28 or S29 or S30 or S31
S33 (MH "Program Evaluation")
S34 Tx ((process* evaluat*))
S35 TX ((program* evaluat*))
S36 (MH "Process Assessment (Health Care)")
S37 S1 OR S2 OR S3 OR S4 OR S5 OR S6 OR S7 OR S8 OR S9 OR S10 OR S11 OR S12 OR S13 OR S14 OR S15 OR S16 OR S17 OR S18 OR S19 OR S20
S38 S21 OR S22 OR S23 OR S24 OR S25 OR S26 OR S27 OR S28 OR S29 OR S30 OR S31
S39 S33 OR S34 OR S35 or S36
S40 S37 AND S38 AND S39

Database: SPORTDiscus (EBSCOhost), search modes - Boolean/Phrase:

S1 SU Sedentary Lifestyle
S2 TI (sedentary or sitting or sedentariness or sedentarism)
S3 TI ((sedentary or sitting or seated) N5 (behavio* or lifestyle or life-style))) OR AB (seated) N5 (behavio* or lifestyle or life-style)) OR AB ((sedentary or sitting or seated) N5 (behavio* or lifestyle or life-style))) OR AB (seated) N5 (behavio* or lifestyle or life-style))
S4 TI ((sedentary N3 (adult* or men or women or males or females or individuals or people or population*))) OR AB (

For peer review only

(sedentary N3 (adult* or men or women or males or females or individuals or people or population*)))
S5 TI (((sitting or sit or seated or stationary or standing) N3 (task* or time or bout* or work* or break*))) OR AB (((sitting or sit or seated or stationary or standing) N3 (task* or time or bout* or work* or break*)))
S6 TX(inactiv* or no exercise or nonexercise or non exercise) N3 (adult* or men or women or males or females or individuals or people))
S7 TI "low energy expenditure" OR AB "low energy expenditure"
S8 TI "physical* inactiv*" OR AB "physical* inactiv*"
S9 TI (leisure time N5 ("physical* activ*" or passive or inactiv*))) OR AB (leisure time N5 ("physical* activ*" or passive or inactiv*))
S10 TI "physical activity level*" OR AB "physical activity level*"
S11 TI ((sitting or lying) N2 posture*) OR AB ((sitting or lying) N2 posture*)
S12 TI (prolong* N2 (reclin* or sit or sitting or seated)) OR AB (prolong* N2 (reclin* or sit or sitting or seated))
S13 TX((computer* or television or tv or video game? or videogame? or gaming) and (sedentary or physical* activity* or sitting or seated or underactiv* or under activ*))
S14 TI "chair rise*" OR AB "chair rise*"
S15 TI "sit* less" OR AB "sit* less"
S16 TI ((light or low) N1 "physical activ*") OR AB ((light or low) N1 "physical activ*")
S17 TI ((decrease or reduc* or discourag* or lessen*) N3 (sit or sitting or stand or standing or "physical* inactiv*")) OR AB ((decrease or reduc* or discourag* or lessen*) N3 (sit or sitting or stand or standing or "physical* inactiv*"))
S18 TI (time N5 (computer* or television or tv or "video game*" or videogame* or gaming or screen or media)) OR AB (time N5 (computer* or television or tv or "video game*" or videogame* or gaming or screen or media))
S19 TI ((watch* or view*) N5 (television or tv)) OR AB ((watch* or view*) N5 (television or tv))
S20 TI (play* N5 ("video game*" or videogame* or "computer game*")) OR AB (play* N5 ("video game*" or videogame* or "computer game*"))
S21 ((DE "RANDOMIZED controlled trials")))
S22 TX allocat* random*
S23 DE "QUANTITATIVE research"
S24 DE "PLACEBOS (Medicine)"
S25 TX placebo*
S26 TX random* allocat*
S27 TX random* assign*
S28 TX randomi* control* trial*
S29 TX clinic* n1 trial*
S30 DE "CLINICAL trials"
S31 AB randomly
S32 AB randomized
S33 S21 OR S22 OR S23 OR S24 OR S25 OR S26 OR S27 OR S28 OR S29 OR S30 OR S31 OR S32
S34 SU program evaluation
S35 TX program* evaluat*
S36 TI process* evaluat*
S37 S34 OR S35 OR S36
S38 S1 OR S2 OR S3 OR S4 OR S5 OR S6 OR S7 OR S8 OR S9 OR S10 OR S11 OR S12 OR S13 OR S14 OR S15 OR S16 OR S17 OR S18 OR S19 OR S20
S39 S33 AND S37 AND S38

<u>Database: Cochrane Database of Systematic Reviews (Wiley):</u>

#1 MeSH descriptor: [Sedentary Behavior] this term only
#2 sedentary or sitting or sedentariness or sedentarism:ti
#3 (sedentary or sitting or seated) near/5 (behavio* or lifestyle or life-style):ti,ab,kw (Word variations have been searched)
#4 sedentary near/3 (adult* or men or women or males or females or individuals or people or population*):ti,ab,kw (Word variations have been searched)
#5 (sitting or sit or seated or stationary or standing) near/3 (task* or time or bout* or work* or break*):ti,ab,kw (Word variations have been searched)
#6 ((inactiv* or no exercise or nonexercise or non exercise) near/3 (adult* or men or women or males or females or individuals or people)):ti,ab,kw
#7 "low energy expenditure":ti,ab,kw (Word variations have been searched)
#8 ("physical* inactive" or "physical inactivity"):ti,ab,kw (Word variations have been searched)
#9 "leisure time" near/5 ("physical* activ*" or passive or inactiv*):ti,ab,kw (Word variations have been searched)

For peer review only

#10 "physical activity level*":ti,ab,kw (Word variations have been searched)
#11 (sitting or lying) near/2 posture*:ti,ab,kw (Word variations have been searched)
#12 prolong* near/2 (reclin* or sit or sitting or seated):ti,ab,kw (Word variations have been searched)
#13 "chair rise*":ti,ab,kw (Word variations have been searched)
#14 "sit* less":ti,ab,kw (Word variations have been searched)
#15 (light or low) near/1 "physical activ*":ti,ab,kw (Word variations have been searched)
#16 time near/5 (computer* or television or tv or "video game*" or videogame* or gaming or screen or media):ti,ab,kw (Word variations have been searched)
#17 (watch* or view*) near/5 (television or tv):ti,ab,kw (Word variations have been searched)
#18 play* near/5 ("video game*" or videogame* or "computer game*"):ti,ab,kw (Word variations have been searched)
#19 (decrease or reduc* or discourag* or lessen*) near/3 (sit or sitting or stand or standing or "physical* inactiv*"):ti,ab,kw (Word variations have been searched)
#20 ((computer* or television or tv or video game* or videogame* or gaming) and (sedentary or physical* activity* or sitting or seated or underactiv* or under activ*)):ti
#21 {or #1-#20}
#22 MeSH descriptor: [Program Evaluation] this term only
#23 ("program* evaluation*"):ti,ab,kw
#24 "process* evaluation*":ti,ab,kw
#25 MeSH descriptor: [Process Assessment, Health Care] this term only
#26 {or #22-#25}
#27 #21 and #26

For peer review only

<u>Database: Cochrane Central Register of Controlled Trials (Wiley):</u>

#1 MeSH descriptor: [Sedentary Behavior] this term only
#2 sedentary or sitting or sedentariness or sedentarism:ti
#3 (sedentary or sitting or seated) near/5 (behavio* or lifestyle or life-style):ti,ab,kw (Word variations have been searched)
#4 sedentary near/3 (adult* or men or women or males or females or individuals or people or population*):ti,ab,kw (Word variations have been searched)
#5 (sitting or sit or seated or stationary or standing) near/3 (task* or time or bout* or work* or break*):ti,ab,kw (Word variations have been searched)
#6 ((inactiv* or no exercise or nonexercise or non exercise) near/3 (adult* or men or women or males or females or individuals or people)):ti,ab,kw
#7 "low energy expenditure":ti,ab,kw (Word variations have been searched)
#8 ("physical* inactive" or "physical inactivity"):ti,ab,kw (Word variations have been searched)
#9 "leisure time" near/5 ("physical* activ*" or passive or inactiv*):ti,ab,kw (Word variations have been searched)
#10 "physical activity level*":ti,ab,kw (Word variations have been searched)
#11 (sitting or lying) near/2 posture*:ti,ab,kw (Word variations have been searched)
#12 prolong* near/2 (reclin* or sit or sitting or seated):ti,ab,kw (Word variations have been searched)
#13 "chair rise*":ti,ab,kw (Word variations have been searched)
#14 "sit* less":ti,ab,kw (Word variations have been searched)
#15 (light or low) near/1 "physical activ*":ti,ab,kw (Word variations have been searched)
#16 time near/5 (computer* or television or tv or "video game*" or videogame* or gaming or screen or media):ti,ab,kw (Word variations have been searched)
#17 (watch* or view*) near/5 (television or tv):ti,ab,kw (Word variations have been searched)
#18 play* near/5 ("video game*" or videogame* or "computer game*"):ti,ab,kw (Word variations have been searched) 478
#19 (decrease or reduc* or discourag* or lessen*) near/3 (sit or sitting or stand or standing or "physical* inactiv*"):ti,ab,kw (Word variations have been searched)
#20 ((computer* or television or tv or video game* or videogame* or gaming) and (sedentary or physical* activity* or sitting or seated or underactiv* or under activ*)):ti
#21 {or #1-#20}
#22 MeSH descriptor: [Program Evaluation] this term only
#23 ("program* evaluation*"):ti,ab,kw
#24 "process* evaluation*":ti,ab,kw
#25 MeSH descriptor: [Process Assessment, Health Care] this term only
#26 {or #22-#25}
#27 #21 and #26

<u>AMED (Allied and Complementary Medicine) (OVID) <1985 to May 2020>:</u>

1 Sedentary Lifestyle/
2 (sedentary or sitting or sedentariness or sedentarism).ti.
3 ((sedentary or sitting or seated) adj5 (behavio* or lifestyle or life-style)).tw.
4 ((inactiv* or no exercise or nonexercise or non exercise) adj3 (adult? or men or women or males or females or individuals or people)).tw.
5 (sedentary adj3 (adult? or men or women or males or females or individuals or people or population?)).tw.
6 ((sitting or sit or seated or stationary or standing) adj3 (task* or time or bout* or work* or break*)).tw.
7 low energy expenditure.tw.
8 physical* inactiv*.tw.
9 (leisure time adj5 (physical* activ* or passive or inactiv*)).tw.
10 "physical activity level*".tw.
11 ((sitting or lying) adj2 posture*).tw.
12 (prolong* adj2 (reclin* or sit or sitting or seated)).tw.
13 chair rise?.tw.
14 "sit* less".tw.
15 ((light or low) adj "physical activ*").tw.
16 ((decrease or reduc* or discourag* or lessen*) adj3 (sit or sitting or stand or standing or physical* inactiv*)).tw.
17 (time adj5 (computer* or television or tv or video game? or videogame? or gaming or screen or media)).tw.
18 ((watch* or view*) adj5 (television or tv)).tw.
19 (play* adj5 (video game? or videogame? or computer game?)).tw.
20 ((computer* or television or tv or video game? or videogame? or gaming) and (sedentary or physical* activity* or sitting or seated or underactiv* or under activ*)).ti.
21 or/1-20 [sedentary behaviour terms]
22 process evaluat*.mp.
23 "Outcome and Process Assessment"/
24 program evaluat*.mp.
25 or/22-24 [process evaluation]
26 21 and 25 [sedentary behaviour and process evaluation]

<u>Database: Embase Classic+Embase (OVID) <1947 to 2020 May 08>:</u>

1 Sedentary Lifestyle/
2 (sedentary or sitting or sedentariness or sedentarism).ti.
3 ((sedentary or sitting or seated) adj5 (behavio* or lifestyle or life-style)).tw.
4 ((inactiv* or no exercise or nonexercise or non exercise) adj3 (adult? or men or women or males or females or individuals or people)).tw.
5 (sedentary adj3 (adult? or men or women or males or females or individuals or people or population?)).tw.
6 ((sitting or sit or seated or stationary or standing) adj3 (task* or time or bout* or work* or break*)).tw.
7 low energy expenditure.tw.
8 physical* inactiv*.tw.
9 (leisure time adj5 (physical* activ* or passive or inactiv*)).tw.
10 "physical activity level*".tw.
11 ((sitting or lying) adj2 posture*).tw.
12 (prolong* adj2 (reclin* or sit or sitting or seated)).tw.
13 chair rise?.tw.
14 "sit* less".tw.
15 ((light or low) adj "physical activ*").tw.
16 ((decrease or reduc* or discourag* or lessen*) adj3 (sit or sitting or stand or standing or physical* inactiv*)).tw.
17 (time adj5 (computer* or television or tv or video game? or videogame? or gaming or screen or media)).tw.
18 ((watch* or view*) adj5 (television or tv)).tw.
19 (play* adj5 (video game? or videogame? or computer game?)).tw.
20 ((computer* or television or tv or video game? or videogame? or gaming) and (sedentary or physical* activity* or sitting or seated or underactiv* or under activ*)).ti.
21 or/1-20 [sedentary behaviour terms]
22 Randomized controlled trial/
23 Controlled clinical study/
24 22 or 23
25 Random*.tw.
26 randomization/
27 intermethod comparison/
28 placebo.tw.

For peer review only

29 (compare or compared or comparison).ti.
30 ((evaluated or evaluate or evaluating or assessed or assess) and (compare or compared or comparing or comparison)).ab.
31 (open adj label).tw.
32 ((double or single or doubly or singly) adj (blind or blinded or blindly)).tw.
33 double blind procedure/
34 parallel group*1.tw.
35 (crossover or cross over).tw.
36 ((assign* or match or matched or allocation) adj5 (alternate or group*1 or intervention*1 or patient*1 or subject*1 or participant*1)).tw.
37 (assigned or allocated).tw.
38 (controlled adj7 (study or design or trial)).tw.
39 (volunteer or volunteers).tw.
40 human experiment/
41 trial.ti.
42 or/25-41
43 42 or 24
44 (random* adj sampl* adj7 ("cross section*" or questionnaire*1 or survey* or database*1)).tw. not (comparative study/ or controlled study/ or randomi?ed controlled.tw. or randomly assigned.tw.)
45 Cross-sectional study/ not (randomized controlled trial/ or controlled clinical study/ or controlled study/ or randomi?ed controlled.tw. or control group*1.tw.)
46 (((case adj control*) and random*) not randomi?ed controlled).tw.
47 (Systematic review not (trial or study)).ti.
48 (nonrandom* not random*).tw.
49 "Random field*".tw.
50 (random cluster adj3 sampl*).tw.
51 (review.ab. and review.pt.) not trial.ti.
52 "we searched".ab. and (review.ti. or review.pt.)
53 "update review".ab.
54 (databases adj4 searched).ab.
55 (rat or rats or mouse or mice or swine or porcine or murine or sheep or lambs or pigs or piglets or rabbit or rabbits or cat or cats or dog or dogs or cattle or bovine or monkey or monkeys or trout or marmoset*1).ti. and animal experiment/ (1058538)
56 Animal experiment/ not (human experiment/ or human/)
57 or/44-56
58 43 not 57 [Cochrane Embase RTC search filter Jan 2015]
59 program evaluat*.mp.
60 health care quality/
61 process* evaluat*.mp.
62 or/59-61 [process evaluation]
63 21 and 58 and 62 [sedentary behaviour and RCTs and process evaluations]
64 remove duplicates from 63

<u>Database: APA PsycInfo (OVID) <1806 to May Week 1 2020>:</u>

1 SEDENTARY BEHAVIOR/
2 (sedentary or sitting or sedentariness or sedentarism).ti.
3 ((sedentary or sitting or seated) adj5 (behavio* or lifestyle or life-style)).tw.
4 ((inactiv* or no exercise or nonexercise or non exercise) adj3 (adult? or men or women or males or females or individuals or people)).tw.
5 (sedentary adj3 (adult? or men or women or males or females or individuals or people or population?)).tw.
6 ((sitting or sit or seated or stationary or standing) adj3 (task* or time or bout* or work* or break*)).tw.
7 low energy expenditure.tw.
8 physical* inactiv*.tw.
9 (leisure time adj5 (physical* activ* or passive or inactiv*)).tw.
10 "physical activity level*".tw.
11 ((sitting or lying) adj2 posture*).tw.
12 (prolong* adj2 (reclin* or sit or sitting or seated)).tw.
13 chair rise?.tw.
14 "sit* less".tw.
15 ((light or low) adj "physical activ*").tw.
16 ((decrease or reduc* or discourag* or lessen*) adj3 (sit or sitting or stand or standing or physical* inactiv*)).tw.
17 (time adj5 (computer* or television or tv or video game? or videogame? or gaming or screen or media)).tw.

For peer review only

18 ((watch* or view*) adj5 (television or tv)).tw.
19 (play* adj5 (video game? or videogame? or computer game?)).tw.
20 ((computer* or television or tv or video game? or videogame? or gaming) and (sedentary or physical* activity* or sitting or seated or underactiv* or under activ*)).ti.
21 or/1-20 [sedentary behaivour]
22 Treatment Effectiveness Evaluation/
23 exp Treatment Outcomes/
24 Psychotherapeutic Outcomes/
25 PLACEBO/
26 exp Followup Studies/
27 placebo*.tw.
28 random*.tw.
29 comparative stud*.tw.
30 (clinical adj3 trial*).tw.
31 (research adj3 design).tw.
32 (evaluat* adj3 stud*).tw.
33 (prospectiv* adj3 stud*).tw.
34 ((singl* or doubl*or trebl* or tripl*) adj3 (blind* or mask*)).tw.
35 or/22-34 [RCT filter adapted from Watson RJ, Richardson PH 1999]
36 program evaluat*.mp.
37 process* evaluat*.mp.
38 evaluation/
39 or/36-38 [process evaluation terms]
40 21 and 35 and 39 [sedentary behaviour and rcts and process evalutions]

Database: Ovid MEDLINE(R) and Epub Ahead of Print, In-Process & Other Non-Indexed Citations and Daily <1946 to May 08, 2020>:

1 Sedentary Lifestyle/
2 (sedentary or sitting or sedentariness or sedentarism).ti.
3 ((sedentary or sitting or seated) adj5 (behavio* or lifestyle or life-style)).tw.
4 ((inactiv* or no exercise or nonexercise or non exercise) adj3 (adult? or men or women or males or females or individuals or people)).tw.
5 (sedentary adj3 (adult? or men or women or males or females or individuals or people or population?)).tw.
6 ((sitting or sit or seated or stationary or standing) adj3 (task* or time or bout* or work* or break*)).tw.
7 low energy expenditure.tw.
8 physical* inactiv*.tw.
9 (leisure time adj5 (physical* activ* or passive or inactiv*)).tw.
10 "physical activity level*".tw.
11 ((sitting or lying) adj2 posture*).tw.
12 (prolong* adj2 (reclin* or sit or sitting or seated)).tw.
13 chair rise?.tw.
14 "sit* less".tw.
15 ((light or low) adj "physical activ*").tw.
16 ((decrease or reduc* or discourag* or lessen*) adj3 (sit or sitting or stand or standing or physical* inactiv*)).tw.
17 (time adj5 (computer* or television or tv or video game? or videogame? or gaming or screen or media)).tw.
18 ((watch* or view*) adj5 (television or tv)).tw.
19 (play* adj5 (video game? or videogame? or computer game?)).tw.
20 ((computer* or television or tv or video game? or videogame? or gaming) and (sedentary or physical* activity* or sitting or seated or underactiv* or under activ*)).ti.
21 or/1-20 [sedentary behaviour terms]
22 Program Evaluat*.mp. (62861)
23 "Outcome and Process Assessment (Health Care)"/
24 "Process Assessment (Health Care)"/
25 process evaluat*.mp.
26 or/22-25 [process evaluation]
27 randomized controlled trial.pt.
28 controlled clinical trial.pt.
29 randomized.ab.
30 placebo.ab.
31 drug therapy.fs.

For peer review only

32 randomly.ab.
33 trial.ab.
34 groups.ab.
35 27 or 28 or 29 or 30 or 31 or 32 or 33 or 34
36 exp animals/ not humans.sh.
37 35 not 36 [Cochrane RCT filter 2008, sensitivity maximimising]
38 21 and 26 and 37 [sedentary behaviour and process evaluation and RCTs]

Database: Web of Science: Indexes=SCI-EXPANDED, SSCI, CPCI-S, CPCI-SSH, ESCI (Clarivate), Timespan= 1900-2020:

1 TI=((sedentary or sitting or sedentariness or sedentarism))
2 TS=(((sedentary or sitting or seated) NEAR/5 (behavio* or lifestyle or life-style)))
3 TS=((inactive* or "non exercise" or "nonexercise" or "no exercise") near/3 (adult* or men or women or males or females or individuals or people))
4 TS=((sedentary) near/3 (adult* or men or women or males or females or individuals or people or population*))
5 TS=(("leisure time" NEAR/5 ("physical* activ*" or passive or inactiv*)))
6 TS=("physical activity level*" or "physical* inactiv*")
7 TOPIC: (((sitting or lying) near/2 posture*))
8 TOPIC: ((nonexercis* or "non exercis*" or "no exercis*")
9 TOPIC: ("chair rise")
10 TS=((sitting or sit or seated or stationary or standing) NEAR/3 (task* or time or bout* or work* or break*))
11 TS=("sit* less")
12 TOPIC: (((light or low) near/1 "physical activ*"))
13 TS=((decrease or reduc* or discourag* or lessen*) NEAR/3((sit or sitting or stand or standing or "physical* inactiv*")))
14 TS=(time NEAR/5 (computer* or television or tv or "video game*" or videogame* or gaming or screen or media))
15 TS=((watch* or view*) NEAR/5 (television or tv))
16 TS= (play* NEAR/5 ("video game*" or "videogame*" or "computer game*"))
17 TI=((computer* or television or tv or "video game?" or videogame? or gaming) and (sedentary or "physical* activity*" or sitting or seated or underactiv* or under activ*))
18 TOPIC: (random* or RCT or placebo or clinical Near/1 trial*)
19 TS=("program* evaluat*")
20 TS=("process evaluat*")
21 #20 OR #19
22 #1 or #2 or #3 or #4 or #5 or #6 or #7 or #8 or #9 or #10 or #11 or #12 or #13 or #14 or #15 or #16 or #17
23 #22 AND #21 AND #18

Databases: ProQuest Dissertations & Theses A&I, from January 01, 1990 to March 15, 2019:

ti((computer* OR television OR tv OR "video game" OR "videogame*" OR gaming) AND (sedentary OR physical* activity* OR sitting OR seated OR underactiv* OR under activ*)) OR ti(sedentary OR sitting OR elementariness OR sedentary OR (sedentary OR sitting OR seated) N5 (behavio* OR lifestyle OR life-style)) OR ti((sitting OR sit OR seated OR stationary OR standing) N3 (task* OR time OR bout* OR work* OR break*)) OR ti("physical* inactiv*" OR "chair rise*" OR "low energy expenditure" OR "sit less") OR ti((watch* OR view*) N5 (television OR tv)) OR ti(play* N5 ("video game*" OR videogame* OR "computer game*")) OR ti(time N5 (computer* OR television OR tv OR "video game*" OR videogame* OR gaming OR screen OR media)) OR ti((computer* OR television OR tv OR "video game" OR "videogame*" OR gaming) AND (sedentary OR physical* activity* OR sitting OR seated OR underactiv* OR "under activ*")) AND ti("process* evaluation*" OR "program* evaluation*") AND ti(Random* OR RCT OR clinical N1 trial*)

For peer review only

Supplementary file 3. Characteristics of 17 included RCTs_27.05.21

Randomised Control Trials

**Study** *(Authors (Year),* **Country** *(of process evaluation report)*	**Study aims**	**Inclusion/exclusion criteria**	**Sample size, n assigned to intervention /control**	**Participant characteristic** *(Age (mean (SD) or %), Gender (% female), Ethnicity)*	**Study design, RCT type, group, setting**	**Intervention description** *(Content, duration)*	**Control description**	**Data collection and follow ups** *(time-points)*	**Outcome measures for treatment effects** *(~~pre-specified or those only reported~~identified in the study reports)*
**Adams (2012)**<br>USA	Reduce sedentary behaviour, increase light physical activity.<br><br>(Feasibility trial)	**Inclusion:**<br>1. Women between the ages of 35-85;<br>2. BMI >25;<br>3. Be willing to receive intervention materials and messages by email;<br>4. Plan to attend all program and data collection sessions.<br>**Exclusion:**<br>Any reported conditions that prohibited standing or walking.	75<br><br>I: 47<br>C: 28	**Age:**<br>I: 56.73 (12.64)<br>C: 61.38 (12.1)<br><br>**Gender:**<br>100%<br><br>**Ethnicity:**<br>89% Caucasian<br>11% African-American	Cluster randomised controlled<br><br>Weight-loss support club (cluster unit)	On Our Feet intervention – combination of 2 face-to-face interactive group sessions, and 6 weekly email messages.<br><br>6 weeks	Waiting list	Baseline<br>6 weeks	1. Time spent in SB; light and moderate PA (accelerometer; IPAQ, Godin Leisure-Time Activity Questionnaire);<br>2. Participant's self-rated level of confidence for reducing sitting and increasing PA behaviours;<br>3. BMI and waist circumference.
**Albright (2015)**<br>USA	Increase moderate to vigorous physical activity.	**Inclusion:**<br>1. Mother of infant aged 2-12 months;<br>2. Inactive (<30 minutes of MVPA/week);<br>3. Healthy, able to do moderate intensity physical activity;<br>4. BMI =18.5-40;<br>5. Not planning to become pregnant in the next 12 months;<br>6. Aged 18-45;<br>7. Had health insurance;<br>8. Read/understood	311<br><br>I: 154<br>C: 157	**Age:**<br>I: 31.6 (5.5)<br>C: 32.1 (5.9)<br><br>**Gender:**<br>100%<br><br>**Ethnicity:**<br>31.5% Native Hawaiian/ Pacific Islander<br>33.8% Asian (Japanese, Filipino, other	Randomised controlled<br><br>Parallel groups<br><br>Community	Tailored telephone counselling, information on website, and pedometer.<br><br>12 months	Information in print or standard website.	Baseline<br>1 month<br>3 months<br>6 months<br>12 months (immediatel after intervention<br>18 months	1. Time spent in MVPA (Active Australia Survey; accelerometer; exercise log);<br>2. Time spent sitting while travelling; at work; watching TV, etc. (Active Australia Survey);<br>3. Body mass index;<br>4. Self-efficacy for PA (instrument designed to assess self-confidence to overcome barriers to PA, modified with questions tailored to new mothers);<br>5. Psychosocial mediators

For peer review only

For peer review only - http://bmjopen.bmj.com/site/about/guidelines.xhtml

Supplementary file 3. Characteristics of 17 included RCTs_27.05.21

		English;<br>9. Physician's written approval if history of contraindicated conditions.<br>**Exclusion:**<br>1. Pregnant;<br>2. Planning to leave Oahu, Hawaii in the next year (permanently move away);<br>3. Diagnosis of cancer, coronary heart disease (including atrial fibrillation), insulin-dependent diabetes mellitus (IDDM), and other atherosclerotic cardiovascular diseases (e.g., stroke).		Asian)<br>16.4% Mixed race<br>15.1% White<br>2.6% Black/ Native American<br>0.6% Unknown					survey.
**Benedetti (2020)**<br><br>Brazil	Improve physical activity level.	**Inclusion:**<br>1. Aged ≥60;<br>2. No severe physical and/or mental health impairments;<br>3. Had not participated in physical activity programs in the past 6 months.<br>**Exclusion:**<br>History of heart attack and/or stroke in the past 6 months, cancer diagnosis and/or other severe medical conditions.	114<br><br>BCG: 36<br>TEG: 52<br>C: 26	**Age:**<br>BCG: 69.7 (6.9)<br>TEG: 71.3 (7.3)<br>C: 67.2 (5.8)<br><br>**Gender:**<br>80.7%<br><br>**Ethnicity:**<br>Not reported	Cluster randomised controlled<br><br>Public health centres (cluster unit)	BCG: 12 weekly meetings behavioural change programme that was adapted from "Active Living Every Day" from USA.<br><br>TEG: 12-week (3 times per week) exercise class conducted at	No intervention	Baseline<br>3 months<br>6 months<br>12 months	1. Time spent in SB; light PA; and MVPA (accelerometers);<br>2. BMI;<br>3. Quality of life (WHOQOL-BREF and WHOQOL-OLD).

For peer review only

Supplementary file 3. Characteristics of 17 included RCTs_27.05.21

						local HCs.			
**Berendsen (2015)**<br><br>The Netherlands	Improve physical activity and dietary behaviour.	**Inclusion:**<br>1. Weight-related health risk;<br>2. Inactive lifestyle (not doing 30 minutes moderate physical activity for at least 5 days per week);<br>3. Motivated for behavioural change;<br>4. BMI= 25-30, with a large waist circumference (men greater than 102 cm, women greater than 88 cm) with comorbidity (cardiovascular disease and/or T2DM, arthrosis and sleep apnoea), or<br>5. BMI= 30-35, with a normal or large waist circumference with comorbidity, or<br>6. BMI= 35-40, with a normal or large waist circumference with risk factors for cardiovascular disease or T2DM and without other comorbidities.<br>**Exclusion:**<br>1. Serious mobility limitations precluding participation;<br>2. Pregnancy.	411<br><br>I: 247<br>C: 164	**Age:**<br>I: 55.9 (12.3)<br>C: 53.8 (12.4)<br><br>**Gender:**<br>64.7%<br><br>**Nationality:**<br>88.8% Dutch	Cluster randomised controlled<br><br>GP practices (Cluster unit)	Supervised exercise programme based on BeweegKuur – individual and group meetings with lifestyle advisor, dietitian, and intensive support from physical therapist.<br><br>12 months	Start-up exercise programme based on BeweegKuur – same number of meetings with lifestyle advisor and dietitian as the intervention group, few numbers of meeting with physical therapist.<br><br>12 months	Activity monitor, physiological measures:<br>Baseline<br>12 months<br>24 months<br><br>IPAQ, dietary habits:<br>Baseline<br>6 months<br>12 months<br>18 months<br>24 months<br><br>EQ-6D, healthcare costs:<br>Baseline, then every 3 months until 24 months	1. Time spent PA (accelerometer; IPAQ), sedentary, standing or active (accelerometer);<br>2. Dietary habits;<br>3. Quality of Life (EQ-6D);<br>4. Medication;<br>5. Side-effects;<br>6. Direct and indirect costs;<br>7. Health risk, e.g. waist circumference, body composition, blood pressure, resting heart rate, blood biochemistry, and physical fitness.
**Biddle (2017)**	Reduce sitting time.	**Inclusion:**<br>1. Age 18-40, BMI ≥30	187	**Age:**<br>I: 32.4 (5.4)	Randomised controlled	STAND – A group-based	Information leaflet focusing	Baseline<br>3 months	1. Time spent in SB;<br>2. Number of breaks in SB

For peer review only

Supplementary file 3. Characteristics of 17 included RCTs_27.05.21

UK		(≥27.5 for South Asians).<br>2. Age 18-40, BMI ≥25 (≥23 for South Asians), with ≥1 additional risk factor for diabetes.<br>**Exclusion:**<br>Significant illness, steroid use, diabetes, pregnancy or an inability to communicate in English.	I: 94<br><br>C: 93	C: 33.3 (5.8)<br><br>**Gender:**<br>68.5%<br><br>**Ethnicity:**<br>19.8% black and minority ethnic groups	Parallel groups<br><br>Community	structured education workshop.<br><br>6 weeks	on T2DM, the importance of increasing physical activity and decreasing sedentary behaviour.	12 months	(SB to upright movement) per day (Both by IPAQ and accelerometer);<br>3. Biochemical variables (glucose control, insulin sensitivity, cholesterol levels);<br>4. Anthropometric data (BP, weight, body composition, waist circumference);<br>5. Quality of life (EQ-5D);<br>6. Self-efficacy for SB change;<br>7. Anxiety and depressions (HADS).
**Blunt (2018)**<br><br>Canada	Increase physical activity levels.	**Inclusion:**<br>1. Age 18-85;<br>2. ≥1 self-reported or measured risk factor for chronic disease including: BMI >25, <150 min of exercise/week, ≥3 hours sitting/day, <8 fruit and vegetable servings/day, diagnosis of metabolic syndrome or T2DM.<br>**Exclusion:**<br>Unable to comprehend the letter of information and consent documentation.	118<br><br>I: 59<br>C: 59	**Age:**<br>I: 56.8 (12.3)<br>C: 58.6 (14.7)<br><br>**Gender:**<br>78.8%<br><br>**Ethnicity:**<br>97.5% White	Randomised controlled<br><br>Parallel group<br><br>Primary care health centres	3-phases HealtheSteps™ program – in-person lifestyle coaching, and access to a suite of eHealth technology support.<br><br>18 months	Usual-care wait-list control to begin HealtheSteps™ 6 months after baseline.	Baseline<br>6 months (end of active phase intervention)<br><br>Additional for intervention group in minimally-support phase:<br>12 months<br>18 months	1. Mean daily steps (pedometer; self-report);<br>2. Time spent in PA; sitting (IPAQ);<br>3. Eating habits (STC; modified DINE);<br>4. Quality of life (EQ-5D; EQ-VAS);<br>5. Weight and body composition<br>6. Blood pressure;<br>7. Adverse events.
**Elramli (2017)**<br><br>UK	Increase average daily step count.	**Inclusion:**<br>1. Aged ≥18;<br>2. Confirmed diagnosis of Rheumatoid Arthritis (RA) according to ACR/EULAR 2010 criteria, within 5	76<br><br>I: 39<br>C: 37	**Age:**<br>I: 58.2 (13.5)<br>C: 58.6 (15.8)<br><br>**Gender:**<br>83.9%	Randomised controlled<br><br>Parallel groups	Walk for Rheumatoid Arthritis (WARA) – 6 group sessions in	1 group education session on importance of exercise and healthy diet;	Baseline<br>13 weeks<br>26 weeks<br>52 weeks	1. Daily step count (accelerometer);<br>2. Time spent in SB (accelerometer);<br>3. Time spent in sitting; PA (IPAQ);

For peer review only

Supplementary file 3. Characteristics of 17 included RCTs_27.05.21

		years of diagnosis.<br>**Exclusion:**<br>1. Pregnant, severe hypertension, joint replacement within last 6 months, unstable cardiac conditions, or other serious pathology which would affect ability to take part in physical activity;<br>2. Unable to understand written and spoken English or had cognitive impairment.		**Ethnicity:**<br>Not reported	Community	first 7 weeks, 2 booster group sessions in week 14 and 28, personal support from physiotherapist on week 7, 9, and 11. Pedometers and PA diaries were given with instructions.<br><br>28 weeks	and written educational material. At end of trial (12-month), provided pedometer and PA diaries, with advice on use.		4. Disease activity (SDAI);<br>5. RA Quality of life (RAQoL);<br>6. Functional capacity (6MWT; MHAQ; hand grip test);<br>7. Cardiovascular risk factors (Blood biochemical variables; ASSIGN score Version 1.5.1; BMI; waist and hip circumferences);<br>8. Dietary assessment (DINE);<br>9. PA self-efficacy.
**Harris (2018)**<br><br>UK	Increase physical activity.	**Inclusion:**<br>1. Aged 45-75;<br>2. Registered at 1 of the 6 participating general practices;<br>3. Able to walk outside the home and with no contraindications to increasing their moderate intensity physical activity levels.<br>**Exclusion:**<br>1. Achieving at least 150 minutes of at least moderate intensity physical activity weekly;<br>2. Living in residential or nursing home, or housebound;<br>3. ≥3 falls, or ≥1 fall required attention, within	1,023<br><br>I:<br>Postal: 339<br>Nurse: 346<br><br>C: 338	**Age:**<br>45-54: 33.2%<br>55-64: 37.8%<br>65-75: 28.9%<br><br>**Gender:**<br>64.1%<br><br>**Ethnicity:**<br>80.3% White<br>10.3% Black<br>6.9% Asian<br>2.5% Other	Randomised controlled<br><br>Parallel groups by household<br><br>Community	1. Postal – pedometer, physical activity diary, and instructions for a 12-week walking programme sent by post.<br><br>2. Nurse support – provided pedometer, physical activity diary, and instructions by a practice nurse, who	Usual physical activity, provided a pedometer and guidance on a 12-week walking programme at end of trial.	Baseline<br>3 months<br>12 months	1. Daily step count (accelerometer);<br>2. Time spent in at least moderate PA (accelerometer);<br>3. Time spent in SB (accelerometer);<br>4. Self-reported PA (GPPAQ; IPAQ);<br>5. Cost-effectiveness to health services;<br>6. Exercise self-efficacy;<br>7. Anxiety, depression;<br>8. Quality of life (EQ-5D);<br>9. BMI; waist circumference; body fat;<br>10. Adverse events;<br>11. Health service use.

For peer review only

Supplementary file 3. Characteristics of 17 included RCTs_27.05.21

		last year;<br>4. Terminal illness, dementia, significant cognitive impairment, blind, new onset chest pain, MI, pregnant, conditions which GP judged for exclusion.				also provided 3 meetings over 3 months to facilitate participants to be more active.			
**Lakerveld (2012)**<br><br>The Netherlands	Improve lifestyle behaviour (dietary, physical activity, and/or smoking).	**Inclusion:**<br>1. Aged 30-50;<br>2. Moderate or high risk of CVD (according to SCORE), or a high risk of T2DM (according to ARIC Study).<br>**Exclusion:**<br>1. Having diabetes;<br>2. Previous CVD;<br>3. Pregnancy;<br>4. Current malignant disease;<br>5. (Severe) mobility problems.	622<br><br>I: 314<br>C: 308	**Age:**<br>I: 43.6 (5.1)<br>C: 43.4 (5.5)<br><br>**Gender:**<br>58%<br><br>**Ethnicity:**<br>Not reported	Randomised controlled<br><br>Parallel groups<br><br>General Practices	Cognitive behavioural programme aimed at modifying dietary, and/or physical activity, and/or smoking behaviour, maximum of six individual counselling sessions of 30 minutes, followed by 3-monthly booster sessions by phone.<br><br>Intervention duration unclear	Provision of health brochures only	Baseline<br>6 months<br>12 months<br>24 months	1. Cardiovascular risk score;<br>2. Diabetes risk score;<br>3. Dietary behaviour (Food Frequency Questionnaire);<br>4. Time spent in PA and SB (SQUASH; a subscale of AQuAA);<br>5. Smoking behaviour;<br>6. Determinants of behavioural change;<br>7. Medical care utilisation;<br>8. BMI, waist-hip circumferences;<br>9. Cost-effectiveness and cost-utility in the societal perspective;<br>10. Quality of life (EQ-5D);<br>11. Blood pressure;<br>12. Blood biochemistry.
**Lane (2010)**<br><br>Ireland	To assess the impact of a community	**Inclusion:**<br>1. A population sample of women participating in a mass 10 km event;	176<br><br>I: 85<br>C: 91	**Age:**<br>21-49: 84%<br><br>**Gender:**	Randomised controlled<br><br>Parallel	2 booklets delivered by post – Booklet 1	Placebo treatment – a healthy eating and nutrition	Baseline<br>6 weeks	1. Time spent in sitting;<br>2. Time spent in sufficient PA levels;<br>3. Time spent in total PA (All

For peer review only

Supplementary file 3. Characteristics of 17 included RCTs_27.05.21

	based, low-contact intervention on the physical activity habits of insufficiently active women.	2.Consented to follow-ups 2 and 6 months afterwards;<br>3. Those who had relapsed to insufficient levels of physical activity were invited.		100%<br><br>**Ethnicity:**<br>Not reported	groups<br><br>Community	targeted the earliest stages of motivational readiness, and step-by-step guide to increase motivation. Booklet 2 targeted already motivated and active stage with information about moderate intensity PA, and staying active.	booklet, delivered by post.		of above by bespoke self-report questionnaire);<br>4. Readiness to change (exercise motivational stage).
**Matson (2018)**<br><br>USA	To decrease sitting; increase standing time and light physical activity.<br><br>(Pilot trial)	**Inclusion:**<br>1. Kaiser Permanente Washington (KPWA) members;<br>2. Age >60;<br>3. BMI 30–50 kg/m²;<br>4. Not residing in long-term care or skilled nursing, no diagnosis of dementia, and no serious mental or a potentially terminal illness.<br>**Exclusion:**<br>1. Unable to stand, were not able to walk one block;<br>2. Participating in another intervention study;	60<br><br>I: 29<br>C: 31	Age:<br>I: 69.0 (4.7)<br>C: 67.8 (5.2)<br><br>Gender:<br>68.3%<br><br>Ethnicity:<br>95.0% Not Hispanic or Latino<br>1.7% Hispanic or Latino<br>3.3% Unknown	Randomised controlled<br><br>Parallel groups<br><br>KPWA primary care clinics	2 health coaching sessions; 4 follow-up health coaching phone calls; and written materials, and email reminders. A wrist-worn device programmed to serve as an outward reminder	Healthy living intervention usually available to the KPWA members<br><br>12 weeks	Baseline<br>12 weeks	1. Time spent in sitting (total time, and number of periods of sitting for ≥30 minutes continuously);<br>2. Daily number of sit-to-stand transitions (breaks from sitting) (Both of above by accelerometer);<br>3. Short Physical Performance Battery;<br>4. Blood pressure;<br>5. Fasting glucose level;<br>6. Total cholesterol level;<br>7. Depressive symptoms (PHQ-8);<br>8.Adverse events.

For peer review only

Supplementary file 3. Characteristics of 17 included RCTs_27.05.21

		3. Reported sitting time of less than 7 hours per day;<br>4. Could not communicate by phone, or speak and read English.				strategy for taking breaks from sitting.<br><br>12 weeks			
**Matthews (2016)**<br><br>UK	Increase walking, reduce sedentary behaviour.	**Inclusion:**<br>1. Aged 18-65;<br>2. Ambulatory and able to walk unaided for 10 minutes at a time, based on self/carer report;<br>3. Any level of intellectual disabilities;<br>4. Not currently taking part in any other research study.<br>**Exclusion:**<br>1. Wheelchair user or significant mobility problems;<br>2. Severe challenging behaviour, or other needs requiring constant one-to-one support from staff;<br>3. Involved in regular physical activity - meeting current public health recommendations for physical activity, for six months or more.	102<br><br>I: 54<br>C: 48	**Age:**<br>I: 44.9 (13.5)<br>C: 47.7 (12.3)<br><br>**Gender:**<br>44.1%<br><br>**Ethnicity:**<br>Not reported	Cluster randomised controlled<br><br>Intellectual disabilities community-based organisations (cluster unit)	Walk Well programme – 3 face-to-face physical activity consultations, written resources for participants and carers, and an individualised, structured walking programme<br><br>12 weeks	12-week waiting list control	Baseline<br>12 weeks<br>24 weeks	1. Daily step count (accelerometer);<br>2. Time spent in SB; MVPA; total PA (accelerometer; IPAQ-S);<br>3. BMI; waist circumference;<br>4. Quality of life (EQ-5D; Subjective Vitality Scale);<br>5. Self-Efficacy for Activity for Persons with Intellectual Disability and Self-Efficacy for Exercise Scale.
**Poston (2013)**<br><br>UK	Behavioural intervention comprising dietary and physical activity	**Inclusion:**<br>1. Pregnant with booking BMI ≥30;<br>2. Singleton pregnancy, gestational age >15^{+0} weeks and <17^{+6} weeks' gestation.<br>**Exclusion:**	183<br><br>I: 94<br>C: 89	**Age:**<br>I: 30.4 (5.7)<br>C: 30.7 (4.9)<br><br>**Gender:**<br>100%<br><br>**Ethnicity:**	Randomised controlled<br><br>Parallel groups<br><br>Antenatal clinics	One-to-one appointment with the health trainer; weekly group sessions for 8 consecutive	Usual antenatal care	Baseline (15^{+0} -18^{+6} weeks' gestation)<br>27^{+0} -28^{+6} weeks' gestation<br>34^{+0} -36^{+0}	1. Attitudinal assessment questionnaire - perceived benefits and barriers and confidence to carry out the dietary and PA behaviours;<br>2. Quality of life (EQ-5D);<br>3. Edinburgh Post Natal Depression Score (EPDS);

For peer review only

Supplementary file 3. Characteristics of 17 included RCTs_27.05.21

	changes to improve glycaemic control in obese pregnant women.<br><br>(Feasibility trial)	1. Gestation <15+0 weeks and >17+6 weeks;<br>2. Pre-existing diabetes;<br>3. Pre-existing essential hypertension (treated);<br>4. Pre-existing renal disease, multiple pregnancies, systemic lupus erythematosus (SLE), antiphospholipid syndrome, sickle cell disease; thalassemia; celiac disease, currently prescribed metformin; thyroid disease or current psychosis.		56.3% White<br>38.3% Black<br>1.6% Asian<br>3.8% Other		weeks from approximately 19 weeks' gestation; dietary advice, and physical activity level advice; plus usual antenatal care.<br><br>8 weeks		weeks' gestation	4. Dietary assessment;<br>5. Time spent in SB; light PA; MVPA (accelerometer; RPAQ);<br>6. Maternal outcomes: diagnosis of GDM and pre-eclampsia, gestational weight gain, mode of delivery, blood loss at delivery, inpatient nights, detailed clinical and family history, health in current pregnancy, early pregnancy data (ultrasound scan, nuchal screening), blood pressure, routine blood results;<br>7. Neonatal outcomes: birthweight, anthropometry, inpatient nights.
**School of Public Health, HKU (2017)**<br><br>Hong Kong	Healthier lifestyle by adopting Zero Time Exercise (ZTEx), and enhance positive family communication and personal and family wellbeing.	**Inclusion:**<br>1. Aged ≥18 years;<br>2. Parents/grandparents with ≥1 child/grandchild aged 3–17;<br>3. Primary education or higher; and able to read and write Chinese;<br>**Exclusion:**<br>Serious health conditions that might prevent from participating in low intensity physical activity.	728<br><br>I: 386<br>C:342	**Age:**<br>Majority aged 30-49<br>I: 87%<br>C: 84%<br><br>**Gender:**<br>92.1%<br><br>**Ethnicity:**<br>Not reported	Cluster randomised controlled<br><br>Integrated Family Service Centres (cluster unit)	Physical activity intervention – 4 group sessions over 12 months; biweekly/ monthly mobile messages to improve physical activity habit.<br><br>12 months	Healthy eating intervention – similar structural design as intervention group.<br><br>12 months	Baseline<br>3 months<br>6 months<br>12 months	1. Time spent in SB; PA (IPAQ-C);<br>2. Physical fitness performance (hand grip strength; time spent standing on 1 leg; foot pedalling duration);<br>3. Dietary habits;<br>4. Self-reported wellbeing (personal-health; happiness; family harmony).
**Spittaels (2007)**	Increase physical activity.	**Inclusion:**<br>1. Aged 25-55;<br>2. No history of	526<br><br>I:	**Age:**<br>I:<br>Group 1: 39.7	Randomised controlled	Group 1. Online-tailored	Online non-tailored standard	Baseline<br>6 months	1. Time spent in PA; SB (IPAQ).

Supplementary file 3. Characteristics of 17 included RCTs_27.05.21

Belgium		cardiovascular disease;<br>3. Internet access (including email access) either at home or at work.<br><br>**Exclusion:**<br>Not specified.	Group 1: 174<br>Group 2: 175<br><br>C: 177	(8.9)<br>Group 2: 39.3 (8.7)<br><br>C: 40.9 (8.0)<br><br>Gender: 30.6%<br><br>**Ethnicity:**<br>Not reported	Parallel groups<br><br>Internet	physical activity advice + 8-week stage-based reinforcement emails.<br><br>Group 2. Online-tailored physical activity advice.<br><br>6 months	physical activity advice – based on information present in the computer-tailored programme.		In addition, in 1 of 6 worksites (n= 57):<br>2. Time spent in MVPA (accelerometer);<br>3. BMI; body fat; blood pressure; heart rate at rest.
**Stathi (2019)**<br><br>UK	Promote active ageing in socially disengaged, inactive older adults.<br><br>(Feasibility trial)	**Inclusion:**<br>1. Sedentary retired adults aged ≥65, reported spending <20 min per week in the past month in MVPA;<br>2. Capable of walking at least 200m.<br><br>**Exclusion:**<br>1. Disease or disability that seriously precluded participation in out-of-house activities, diagnosis of dementia;<br>2. Already meeting current PA recommendations, and regularly engaging with local groups and Activities.	39 Participants:<br><br>I: 22<br>C: 17<br><br>(15 voluntary Activators)	**Age:**<br>I: 72.9 (7.3)<br>C: 75 (6.4)<br><br>**Gender:**<br>43.6%<br><br>**Ethnicity:**<br>97% White	Randomised controlled<br><br>Parallel groups<br><br>Community	ACE (Active, Connected, Engaged) intervention –<br>One-to-one support from a peer volunteer (activator) to attend local activities continuously.<br><br>6 months	Waiting-list control group, and received written materials about local initiatives.	Baseline<br>6 months	1. Number of out of house activities;<br>2. Time spent in SB; lifestyle PA (accelerometer);<br>3. Lower limb function (SPPB);<br>4. Wellbeing (life-satisfaction; subjective wellbeing; resilience; and vitality);<br>5. Self-perceived barriers to activity in the neighbourhood.
**Williams 2019**	Reduce sedentary	Inclusion:<br>1. A diagnosis of any	40	Age:<br>I+C: 43 years	Randomised controlled	WTW intervention	Treatment as usual which	Baseline<br>17 weeks	1. Time spent in SB; light PA; MVPA (accelerometer);

For peer review only

Supplementary file 3. Characteristics of 17 included RCTs_27.05.21

UK	behaviour, increase physical activity.<br><br>(Pilot study)	serious mental illness;<br>2. Meeting any one of the following criteria: i) overweight, ii) at risk of or have diabetes, iii) in the clinician's view, have a sedentary lifestyle, iv) or smoke tobacco;<br>3. Ability to provide informed consent and understands English;<br>4. Aged ≥18 years.	I: 20<br>C: 20	(20–56)<br><br>Gender: 45%<br><br>Ethnicity:<br>50% Black<br>27.5% White<br>12.5% Mixed<br>7.5 Asian<br>2.5 Other	Parallel groups<br><br>3 community mental health teams	including an initial education session, fortnightly coaching, provision of pedometers and access to a weekly walking group.<br><br>17 weeks	consisted of care coordination plus written information on the benefits of increasing activity levels.	6 months	2. Self-report SB and PA (IPAQ);<br>3. Motivation to engage in PA (BREQ-2);<br>4. Blood biochemistry;<br>5. Blood pressure;<br>6. BMI; waist circumference;<br>7. Mental Wellbeing (WEMWBS);<br>8. Functional mobility (TUG test).

Keys:

6MWT = 6-minute Walk Test; ACR/EULAR 2010 criteria = American College of Rheumatology/ European League Against Rheumatism 2010 criteria; ARIC = Atherosclerosis Risk in Communities; AQuAA = Activity Questionnaire for Adolescents & Adults; ASSIGN score = a cardiovascular risk score developed by Dundee University (2006); BCG = Behaviour Change Group; BMI = Body Mass Index; BP = blood pressure; BREQ-2 = Behavioural Regulation in Exercise Questionnaire-2; C = Control group; CVD = Cardiovascular disease; DINE = Dietary Instrument for Nutrition Education; EPDS = Edinburgh Post Natal Depression Score; EQ-5D/6D = European Quality of Life-5 dimensions/6 dimensions; EQ-VAS = European Quality of Life-Visual Analogue Scale; GI = glycaemic index; GP = General practitioner; GPPAQ = General Practice PA Questionnaire; HADS = Hospital Anxiety and Depression Scale; HCP = Health care provider; I = Intervention group; IDDM = insulin-dependent diabetes mellitus; IPAQ = International Physical Activity Questionnaire; IPAQ-C = International Physical Activities Questionnaire-Chinese version; IPAQ-S = International Physical Activity Questionnaire-Short version; KPWA = Kaiser Permanente Washington; MHAQ = Modified Stanford Health Assessment Questionnaire; MI = myocardial infarction; MVPA = Moderate to vigorous physical activity; n = Number of persons; PA = Physical activity; PHQ-8 = Patient Health Questionnaire; RA = Rheumatoid Arthritis; RAQoL = RA Quality of Life; RCT = Randomised Controlled Trial; RPAQ = Recent Physical Activity Questionnaire; SB = Sedentary behaviour; SCORE = Systematic Coronary Risk Evaluation; SD = standard deviation; SDAI = Simple disease activity index; SMART = Specific, Measurable, Achievable, Relevant and Time specific; SPPB = Short Physical Performance Battery; SQUASH = Short Questionnaire to Assess Health Enhancing Physical Activity; STC = Starting the Conversation questionnaire; T2DM = Type 2 Diabetes Mellitus; TEG = Traditional Exercise Group; TUG test = Timed Get Up and Go Test; WEMWBS = Warwick-Edinburgh Mental Wellbeing Scale; WHOQOL = World Health Organization Quality of Life; WTW = Walk this Way

For peer review only

Supplementary file 4.Characteristics of 17 included process evaluations_27.05.21

**Process Evaluation studies**				
**Study** *(Author (Year)*, **Country**	**Aims** *(whether process evaluation was pre-specified before commencing RCT)*	**Sample size and sampling method**	**Study Design (Data collection methods, e.g., mixed methods)**	**Frameworks for process evaluation**
**Adams (2012)**<br><br>USA	To explore overweight and obese women's perceptions of benefits, challenges and effectiveness of the intervention to reduce SB and increase PA. (Pre-specified)	I: n= 47<br>All participants in the intervention group were asked to complete the questionnaires at the mid-point of the intervention, and intervention end or withdrawing.<br><br>1 researcher<br>The researcher leading the PhD project.	Mixed methods:<br>1. By completing online questionnaires in different weeks during the intervention period, the participants evaluated their perceived benefits and barriers, frequency of using the intervention materials, and the effectiveness and ease of use of the intervention elements; and were asked to provide suggestions for improvement.<br>2. The researcher recorded her observations of the challenges, benefits, and costs in implementing the intervention.<br>3. Attendance and retention data were collected to determine attrition.	Not specified
**Albright (2015)**<br><br>USA	To quantify and compare the barriers to MVPA, frequency of achieving MVPA goals, and the relation of persistent barriers to achievement of goals. (Uncertain whether pre-specified or not)	I: n= 115<br>Study records of all participants in intervention group were used.<br><br>Staff conducted the telephone counselling sessions<br>Sessions were recorded, then selected for evaluation (Selection method and number of staff included were unclear – assuming random selection of the records).	1. Checklist to assess fidelity in 80 of the 1,586 recorded telephone counselling sessions.<br>2. Quantified information from telephone counselling sessions to evaluate goals set and achieved, and barriers.<br>3. Study records for assessing the use of intervention materials and attritions.	Not specified
**Benedetti (2020)**<br><br>Brazil	To conduct a comprehensive programme evaluation including all dimensions of RE-AIM using quantitative and qualitative data. (Uncertain whether pre-specified or not)	Participants in the programme<br>Sample size and sampling method not specified, assuming the BCG group only.<br><br>Staff<br>Professionals delivering the programmes, community health workers, and local and city administrators overseeing public health centers. Sample size and sampling method not specified.	Mixed methods:<br>1. 12 focus groups and 32 interviews with participants in the programme, staff delivering the intervention, or those overseeing the venues at the end of the trial.<br>2. Quantitative data in study records about participation, treatment effects, and fidelity.<br>3. Checklist for assessing implementation.	Framework: RE-AIM Framework (Glasgow et al., 1999)

For peer review only - http://bmjopen.bmj.com/site/about/guidelines.xhtml

Supplementary file 4.Characteristics of 17 included process evaluations_27.05.21

**Berendsen (2015)**<br><br>The Netherlands	To provide an insight into possible barriers and facilitators in execution and sustainability of lifestyle interventions in primary care.<br>(Pre-specified)	<u>I: n= 247, C: n= 164</u><br>All participants in intervention and control groups.<br><br><u>25 Health Care Providers</u><br>8 physiotherapists, 7 dietitians, 10 lifestyle advisors (who were practice nurses/ dietitian/ physiotherapists) were selected for the interviews (sampling method not specified).	Mixed methods:<br>1. Face-to-face, semi-structured interviews with HCPs at the end of the trial on fidelity, dose, context and strategy for implementation, and sustainability.<br>2. Questionnaires to participants every 3 months about dose and satisfaction.<br>3. HCP registries and logbooks completed during the trial about dose, fidelity, and attrition.	Frameworks: RE-AIM Framework (Glasgow et al., 1999); Steckler & Linnan (2002); Saunders et al. (2005); Grant et al. (2013)
**Biddle (2017)**<br><br>UK	To understand the trial outcome findings from the delivery of the workshop and participant behaviour change strategies.<br>(Pre-specified)	<u>I: n= 71 (then n= 45 at 6 weeks after the workshop; n=10 at 12 months)</u><br>All participants provided feedback immediately after the workshop, and were contacted at 6 weeks afterwards. Invitations sent to 28 participants at the end of the trial (12 months).<br><br><u>2 Educator/ Facilitator</u><br>All the workshop educator and facilitator were interviewed at the end of the trial.	Mixed methods:<br>1. Evaluation sheet completed by participants immediately after the educational workshop.<br>2. Phone interviews 6 weeks after the workshop.<br>3. Phone interviews at the end of the trial on following the intervention, awareness of risk, and suggestions for improvement.<br>4. Face-to-face interview with each workshop educator/ facilitator at the end of the trial on intervention delivery, anticipated effectiveness of the intervention, and suggestions for improvement.	Framework: MRC Guidance (Craig et al., 2008)
**Blunt (2018)**<br><br>Canada	To examine the acceptability of the intervention programme.<br>(Pre-specified)	<u>I: n= 13</u><br>All participants (n= 39) who attended the follow-up assessment at 12 months were invited to participate in an interview; 13/32 agreed participants purposefully chosen, according to baseline measures, e.g., average step count, and self-rated health.<br><br><u>12 Coaches</u><br>All coaches delivered the intervention, except 1 was unavailable due to scheduling conflicts.	1. Semi-structured interviews with coaches upon programme completion at 6 months, exploring experiences, barriers, and facilitators in delivering the intervention, and suggestions for improvement.<br>2. Semi-structured interview with participants at 12 months about the experience making health behaviour changes, programme successes and challenges, and suggestions for improving intervention.	Not specified
**Elramli (2017)**<br><br>UK	To explore participant views regarding the effectiveness of WARA intervention.<br>(Pre-specified)	<u>I: n= 10</u><br>Participants were chosen from the 3 recruiting hospitals, including both genders, who did and did not change PA level and step counts.	Semi-structured 30-minute phone interview at 6 months to explore participant's views about the effectiveness and overall views of the intervention.	Not specified
**Harris (2018)**	To examine the mechanisms of change by under-standing of	<u>Nurse-supported group I: 295 completed by participants, 251 completed by nurses for participants</u>	Mixed methods:<br>1. Semi-structured phone interviews with participants at the end of the trial, to explore their	Framework: MRC Guidance (Moore et al., 2015)

For peer review only - http://bmjopen.bmj.com/site/about/guidelines.xhtml

Supplementary file 4.Characteristics of 17 included process evaluations_27.05.21

UK	how the intervention was delivered and received, and how this may have affected the outcomes.<br>(Pre-specified)	All participants (n= 346) and nurses asked to complete the alliance questionnaires.<br><br><u>Nurse-supported group: n= 21, Postal group: n= 22</u><br>Semi-structured interviews: Participants consented at baseline, completed intervention at 12 months, selected according to step-count change, and baseline characteristics.<br><br><u>7 Nurses</u><br>All 8 nurses were invited to focus group/ interviews; 1 was unavailable and did not participate.	experiences.<br>2. Semi-structured focus groups/ interviews with nurses at the end of the trial to explore experiences of delivering PA consultations.<br>3. Patient alliance questionnaire and nurse alliance questionnaire on quality of delivery and participant responsiveness, covering different intervention aspects (e.g., working together and goal-setting, number of appointments).<br>4. Intervention session audio-records and checklists for fidelity and dose.<br>5. Return of participant's PA diary for participation, fidelity, and dose.<br>6. Trial administrative records about participation, dose, and fidelity.	
**Lakerveld (2012)**<br><br>The Netherlands	To describe the intervention's reach, effectiveness in terms of process outcomes, adoption, and implementation of intervention.<br>(Pre-specified)	<u>I: n= 267</u><br>All participants (n =314) were asked to complete the questionnaire.<br><br><u>8 Practice nurses</u><br>All the nurses delivering the intervention.	1. Trial records for participations, dose, and treatment effects.<br>2. Questionnaires to participants at 6 months to evaluate satisfaction and effects on determinants of lifestyle behavioural change.<br>3. Questionnaires to nurses at 6 months to evaluate the training and their confidence in delivering the intervention.<br>4. 2 counselling sessions conducted by each nurse was tape-recorded to assess the nurse's competence.	Framework: RE-AIM Framework (Dzewaltowski et al., 2004)
**Lane (2010)**<br><br>Ireland	To explore the effectiveness and acceptability of intervention booklets.<br>(Aim is not specified, but assumed according to the reported results; and process evaluation is assumed to be pre-specified)	<u>I: n= 85</u><br>Participants in the intervention group were contacted.	3 weeks and 6 weeks after baseline data were recorded:<br>Questionnaires were mailed or emailed to participants.	Not specified
**Matson (2018)**	Collecting qualitative results to further inform the feasibility	<u>I: n= 22</u><br>The health coaches reported that 23 of all 29 participants were available, interested, or	Semi-structured exit interviews with participants within 10 days of the final follow-up, to explore their experiences and perceived health impact of the	Not specified

For peer review only - http://bmjopen.bmj.com/site/about/guidelines.xhtml

Supplementary file 4.Characteristics of 17 included process evaluations_27.05.21

USA	and acceptability of the interventions. (Pre-specified)	appropriate for the interview, thus the 23 participants were invited, but 1 participant declined.	intervention.	
**Matthews (2016)**<br><br>UK	To explore the feasibility of a 12-week walking intervention for adults with intellectual disabilities, in relation to context, recruitment and retention, reach, implementation and fidelity.<br>(Pre-specified)	I: n= 20<br>Participants who had and did not have successful outcomes.<br><br>6 Key stakeholders<br>The health professional delivering the intervention; the researcher responsible for intervention delivery and management; 1 participant with positive study outcomes; 1 participant with no significant outcomes; 1 carer; a day centre manager	Mixed methods:<br>All conducted after the end of intervention:<br>1. Semi-structured interviews or focus groups with participants to explore their attitudes towards physical activity and walking, perceived benefits, drawbacks and impact of increased activity, subjective feelings of wellbeing, and any changes in view during the intervention period.<br>2. Interviews with key stakeholders to gain insight from a variety of individuals involved in the study.<br>3. Data input spreadsheet which recorded multiple elements including attendance, reasons for withdrawal from the study, for gaining insight regarding recruitment, retention and reach of the intervention.	Frameworks: MRC Guidance (Moore et al., 2015), WHO (2001); RE-AIM Framework (Glasgow et al., 2012); Steckler & Linnan (2002)
**Poston (2013)**<br><br>UK	To refine the intervention protocol through process evaluation of intervention fidelity.<br>(Pre-specified)	I: n= 9, C: n= 12<br>Participants recruited from each study site, using a maximum diversity sampling approach, following an informed consent procedure.<br><br>130 audio diaries from Health trainers<br>Number of Health trainers completed included, or sampling method not specified.	Mixed methods:<br>All conducted after the end of intervention:<br>1. 17 face-to-face and 4 telephone semi-structured interviews with participants during their pregnancy, to capture their experiences and perceptions of the trial and intervention.<br>2. Audio diaries of health trainers in which they reflected on the fidelity and feasibility of the intervention delivery.<br>3. Study database for evaluating attendance.	Framework: Steckler & Linnan (2002)
**School of Public Health, HKU (2017)**<br><br>Hong Kong	To explore the opinions and experiences of the programme; to evaluate the effectiveness of the programme.<br>(Pre-specified)	I: n= 24, C: n= 8<br>Participants who attended all the 4 sessions were invited.<br><br>8 Social workers and 1 Clerical staff<br>Sampling method not specified.	All conducted at the end of the trial:<br>1. Focus groups with participants to explore their experiences, and the impact of the intervention on their living habits and wellbeing.<br>2. Interviews with staff to collect comments about this study, and suggestions for future improvement.<br>3. Fidelity checks conducted for every session to ensure the quality and implementation of the intervention. Methods and results not reported.	Not specified.
**Spittaels (2007)**	To investigate the effectiveness of	Tailored advice+emails group: n= 128,<br>Tailored advice group: n= 139, C: n= 156	All completed at the end of intervention:<br>1. Questionnaire to all participants to investigate	Not specified

For peer review only - http://bmjopen.bmj.com/site/about/guidelines.xhtml

Supplementary file 4.Characteristics of 17 included process evaluations_27.05.21

Belgium	intervention outside laboratory.<br>(Uncertain whether pre-specified or not)	All participants were asked to complete the questionnaire; included participants were those responded.	whether participants remembered the advice, read the advice, and considered the advice had had a positive impact on their physical activity behaviour.<br>2. Further questions to the Tailored advice+emails intervention group to investigate the number of emails received and read, and their opinion on the provision of emails.	
**Stathi (2019)**<br><br>UK	To determine the relative usefulness of different intervention components, to identify ways to refine or improve the intervention.<br>(Pre-specified)	I: n= 20<br>Sampling method not specified.<br><br>13 Activators<br>Sampling method not specified.<br><br>2 Coordinators<br>Sampling method not specified.	Mixed methods:<br>All conducted at the end of intervention:<br>1. Quantitative process evaluation via a self-administered questionnaire which assessed changes in confidence to get out and about, social support, autonomy, competence, and relatedness.<br>2. 14 semi-structured exit interviews and 7 focus groups conduced with participants, activators and coordinators, to evaluate the effectiveness and suggestions of intervention elements.<br>3. Trial records for evaluating recruitment rate, attendance, completion rate, and acceptability of the intervention.	Framework: MRC Guidance (Moore et al., 2015)
**Williams 2019**<br><br>UK	To establish the feasibility and acceptability of the Walk this Way (WTW) intervention<br>(Pre-specified)	I: n= 5<br>Participants who agreed to be interviewed; sampling method unclear.	Mixed methods:<br>1. Semi-structured interviews to evaluate how participants experienced the intervention, and suggestions for improving the intervention.<br>2. Trial records for calculating recruitment rate, attendance, number of participants completed the intervention and refused outcomes measurements.	Not specified.

Keys: ACR/EULAR 2010 criteria = American College of Rheumatology/ European League Against Rheumatism 2010 criteria; ARIC = Atherosclerosis Risk in Communities; BCG = Behaviour Change Group; BMI = Body Mass Index; C = Control group; CVD = Cardiovascular disease; GP = General practitioner; HCP = Health care provider; I = Intervention group; IPAQ = International Physical Activity Questionnaire; MRC: Medical Research Council; MVPA = Moderate to vigorous physical activity; n = number of persons; PA = Physical activity; PhD = Doctor of Philosophy; RCT = Randomised controlled trial; SB = Sedentary behaviour; SCORE = Systematic Coronary Risk Evaluation; T2DM = Type 2 Diabetes Mellitus; sTEG = Traditional Exercise Group; WHO: World Health Organisation

References for process evaluation theoretical frameworks:

Dzewaltowski, D. A., Glasgow, R. E., Klesges, L. M., Estabrooks, P. A., & Brock, E. (2004). RE-AIM: Evidence-based standards and a web resource to improve translation of research into practice. Annals of Behavioral Medicine, 28(2), 75-80.

For peer review only - http://bmjopen.bmj.com/site/about/guidelines.xhtml

Supplementary file 4.Characteristics of 17 included process evaluations_27.05.21

Glasgow RE, Vogt TM, Boles SM. Evaluating the public health impact of health promotion interventions: the RE-AIM framework. Am J Public Health. 1999;89(9):1322–7.

Glasgow R, Boles S, Vogt T: Reach Effectiveness Adoption Implementation Maintenance (RE-AIM). [19-Feb-2012]; 2012. www.re-aim.org.

Grant A, Treweek S, Dreischulte T, Foy R, Guthrie B. Process evaluations for cluster-randomised trials of complex interventions: a proposed framework for design and reporting. Trials. 2013;14:15.

Moore GF, Audrey S, Baerker M, Bond L, Bonell C, Hardeman W, et al. Process evaluation of complex interventions: Medical Research Council guidance. BMJ. 2015;350:h1258.

Saunders RP, Evans MH, Joshi P. Developing a process-evaluation plan for assessing health promotion program implementation: a how-to guide. Health Promot Pract. 2005;6(2):134–47.

Steckler A, Linnan L. Process Evaluation for Public Health Interventions and Research. San Francisco: Jossey-Bass; 2002.

World Health Organisation [WHO]. Process Evaluation Workbook. Geneva: WHO; 2001.

For peer review only

Supplementary file 5. Delivery and mechanisms_27.05.21

Study (Year)	Intended delivery (aim/ intervention description)	Actual delivery (difference from the intended delivery)	Intended mechanism (theoretical model/ logic model)
**Adams (2012)**	**On Our Feet intervention** – combination of 2 face-to-face interactive group sessions, and 6 weekly email messages. 1-2 Weeks were led in-person by the researcher. 3-6 Weeks were conducted over the internet, mainly by email.<br><br>Participants were given feedback on their initial levels of SB and PA, were led through a goal setting activity and provided with self-monitoring tools, e.g., Actigraph activity monitor. Positively-framed email messages that contained peer-modelled alternatives to sitting and additional behavioural feedback were sent weekly.<br><br>**Control group** – waitlist control.	(Adaptations)<br>1. Due to schedule conflict for 1 chapter, the initial presentation and the goal setting activity took place at the same meeting instead of respective weeks. Participants received extra email and phone contact to answer any questions during the second week.<br>2. While the same visual aids were used in the initial presentation in each chapter, the depth of explanation for each chapter varied according to the participants' questions.<br>3. Proposed group activity on emotions regarding sitting and some segments of the presentation were reduced or removed because of the time limit for the sessions.<br>4. Software problems causing inaccurate estimates of SB provided to some participants.	The intervention focused on improving self-efficacy in the Social Cognitive Theory, by addressing 4 self-efficacy construct – mastery experiences, modelling, verbal and social persuasion, and emotional and physiological states. It combined the various stages of changes in the Transtheoretical Model, to reduce SB and increase PA.<br><br>In the group sessions, video and demonstrations modelled the intervention exercises. Participants set goals and rated their confidence in achieving the goal, which was intended to increase recognition of self-efficacy. The self-monitoring tools assisted the re-evaluation of SB. Tailored feedback on behaviour change facilitated mastery experiences. Group discussions, uses of behavioural cues, and positively-framed emails encouraged and prompted continuous behaviour changes.
**Albright (2015)**	**TTCW intervention** – telephone counselling sessions and a website, tailored to address a woman's specific MVPA benefits and barriers over a 12-month intervention.<br>*17 Telephone counselling:*<br>The health educator discussed MVPA goals, anticipated barriers and resolutions with participants; tracked MVPA goals (type of activity, duration, and intensity); and provided tailored suggestions on the TTCW website, by email, or mail.<br>*Schedule of counselling calls:*<br>Phase 1: weekly calls (for month 1); Phase 2: biweekly calls (2 Months and 3 Months); and Phase 3: monthly calls (4 Months to 12	(Adaptations)<br>1. In TTCW group, only 75% of participants set incremental MVPA goals with a health educator during the intervention period.<br><br>2. Some initial PA goals were set at light intensity, because the participants were relatively inactive at the beginning of the intervention.	The tailored TTCW intervention aimed to positively alter the key mediators of PA – personal, social, and environmental factors, to enhance self-efficacy and reduce barriers, using the Social Cognitive theory and Transtheoretical Model theory.<br><br>Health educators provided counselling calls, using Motivational interviewing, to encourage goals settings, problem-solving, self-monitoring, and self-reinforcement, to integrate PA into daily lives; while preparing the participants to prepare and progress through the stages of change.

For peer review only

Supplementary file 5. Delivery and mechanisms_27.05.21

	Months).<br>*TTCW website:*<br>Contained various resources designed to facilitate MVPA, e.g. behaviour-change tip, calendar listing "baby-friendly" exercise sessions in the community, and newsletters. Participants were informed that the website would be updated 2-3 times per month.<br><br>**SWO (control group)** – "standard" PA information was available on the SWO website, e.g., information about how to become more physically active via links to credible sources (i.e., American Heart Association, etc.). Participants in this group did not receive any telephone calls or goal-setting advice about MVPA.		The TTCW website provided information about supportive environments for the participants to exercise; and suggestions about obtaining social support for PA.
**Benedetti (2020)**	Reported as actually delivered interventions.	**BCG –** the behavioural change programme that was adapted from "Active Living Every Day" (ALED), delivered by specifically trained nutrition and exercise science professionals working at the HCs. The sessions included a series of topics related to behaviour change, aiming at a more active lifestyle.<br><br>**TEG** - received a 12-week exercise class conducted at the local HCs, led by exercise professionals employed by the HCs; 3 times per week for 60 minutes. Each session included warm-up, aerobic exercise at 50–80% of maximum aerobic power, resistance training, and cool-down. Participants' heart rate and ratings of perceived effort were tracked during each session.	The BCG was adapted from "Active Living Every Day," or ALED, from the USA (Bors 2009).<br><br>A series of behaviour change topics were delivered through 12 structured weekly meetings, aiming to achieve a more active lifestyle. The topics included finding new opportunities to be active, overcoming challenges, setting goals and rewarding, gaining confidence, enlisting support, avoiding pitfalls, step by step, positive planning, making lasting changes.
**Berendsen (2015)**	(Protocol)<br>**Supervised programme:** 6-7 individual meetings, and 26–34 group meetings with PT.	(Differences)<br>1. In both programmes the number of meetings with all HCPs was lower than planned in the protocol. Participants of the Supervised	Beweegkuur provided a wide-ranging lifestyle counselling by means of Motivational Interviewing and incorporating the concepts from Self-Determination Theory.

For peer review only - http://bmjopen.bmj.com/site/about/guidelines.xhtml

Supplementary file 5. Delivery and mechanisms_27.05.21

	**Start-up programme (Control):** 6 individual meetings with PT.<br><br>**Both programmes** comprised 6 individual coaching meetings LSA, 3 individual meetings with a dietitian, and 7 dietary group meetings, for 1 year.<br>The initial individual meetings with the HCPs were to set personal (exercise and nutritional) goals, and identify barriers to a healthy lifestyle through motivational interviewing, which were the basis for meetings. At the end of the programme, each participant met with the LSA to evaluate the lifestyle changes and conclude the intervention.	programme attended, compared to participants of the Start-up programme, more meetings with physiotherapists, but fewer with lifestyle advisors and dietitians.<br>2. No PT group meetings were planned in the protocol for the control Start-up group, but some PTs organised over 9 meetings. Some PT of the start-up programme only planned group meetings, instead of the intended individual meetings with each participant.<br>3. For both groups, 3 individual meetings with the dietitians were planned in the protocol, but the Start-up group received a median of 4 meetings (7 meetings at 75th percentile). On the other hand, some participants did not prefer individual meetings which added fees to participants.<br>4. Some dietitians did not plan individual meetings, and therefore felt there was no opportunity to set individual goals.<br>5. Not all participants reported that they set goals with the PA and dietitian; nor the LSA had explicitly concluded the intervention.<br>6. Not all HCPs were trained in Motivational Interviewing techniques.	All HCPs addressed goals and barriers in the different aspects of lifestyle, to promote participant's motivation for behaviour change, problem-solving skills, and thus promoting participant's sustainable self-efficacy and environment to engage in long-term PA and healthy dietary behaviour.<br><br>It has been hypothesised that the additional amount of guidance within the Supervised programme provided additional contacts and guidance, as a hypothesis that the increase in effects on physical activity would lead to bigger treatment effects.
**Biddle (2017)**	(Protocol)<br>A comprehensive health assessment, including blood tests, was conducted at the trial baseline clinic. Results were sent to all participants (intervention and control groups) and discussed in the educational workshops with each participant.<br><br>**STAND Intervention** – A 3-hour group-based educational workshop, based on the DESMOND and PREPARE structured education protocols, delivered by trained educators; plus a motivational follow-up phone call (6 Weeks) to	Delivered as intended.	STAND intervention started with a letter sent to participants at risk of T2DM and an invitation for risk tests, then discussing with an educator about the risk information and amount of SB time, by using the Commonsense Model of Illness.<br><br>The workshop was based on Commonsense Model and Dual Process Theory, in which the trained educators provided information on risk factors and complications relating to T2DM. Participants were encouraged to assess their own health risk, and to identify their modifiable

For peer review only - http://bmjopen.bmj.com/site/about/guidelines.xhtml

Supplementary file 5. Delivery and mechanisms_27.05.21

	review and support participants' behaviour change progress. The 'Gruve' (MUVE, Inc., USA: www.muveinc.com) was provided to participants, for self-monitoring on time spent sedentary and in PA, and prompting for break from prolonged times of inactivity. Text messages were sent to participants to encourage adherence to goals and use of the Gruve.<br><br>**Control group** – received an information leaflet focusing on key illness perceptions of being at risk of T2DM, the importance of increasing physical activity and decreasing sedentary behaviour.		risks.<br><br>Social Cognitive Theory and Behavioural Choice Theory were also employed in the workshop content, to aid participants identifying health risks associated with excess SB, strategies to reduce SB in their daily life, identifying barriers, and setting goals and action plans.<br><br>The self-monitoring tool, the Gruve, was provided to facilitate self-regulation of SB.
**Blunt (2018)**	(Protocol)<br>**The HealtheSteps™ programme** – provided individuals with a specific plan of action to improve their PA levels, healthy eating habits, and reduce sedentary behaviour.<br>*Active phase (0-6 Months):*<br>1. bi-monthly in-person coaching to set prescriptions for physical activity, exercise, and healthy eating; provided by 1 trained HealtheSteps™ coach throughout this phase.<br>2. Access to a Tyze Personal Networks (an online social network to connect with coaches and other participants); phone coaching supports; and a free HealtheSteps™ smartphone app (providing virtual coach, heart rate monitor, step counter, and tracking option to monitor progress).<br>*Maintenance phase I (7-12 Months):* in-person coaching removed, but participants had access to the full suite of eHealth technology supports.<br>*Maintenance phase II (13-18 Months):* access to the full suite of eHealth technology supports removed, and participants only had access to	(Adaptations)<br>The central research team scheduled coaching sessions for some coaches, resulting that some participants had different coaches at each session.	HealtheSteps™ was based on the Social Cognitive theory of self-regulation. The mobile app, online tools and resources, and initial supports from the coaches facilitated positive health behaviour changes and self-management of own risk factors for chronic disease.<br><br>Individualised lifestyle prescriptions were given to participants in the initial phase, using Motivational Interviewing and SMART goal setting principles (specific, measurable, attainable, realistic, and timely for the participant). These aimed to produce positive behaviour change and overcome potential barriers.

For peer review only

Supplementary file 5. Delivery and mechanisms_27.05.21

	publicly available resources and tools.<br><br>**Comparator group (waitlist control)** – This group continued with usual activities without intervention from the study team for the first 6-month period. After the 6 Months follow-up measurements, participants were given the opportunity to start the 6-month HealtheSteps™ programme.		
**Elramli (2017)**	Reported as actually delivered interventions.	**The WARA intervention consisted of 2 components** – *PA component:* a pedometer supported walking programme, aiming to increase participant's average daily step count by 3000 steps above their baseline value, on at least 5 days of the week by 6 months, and to maintain for up to 12 months; and to comply with the UK physical activity guidelines (2011) recommended of a total of 150 minutes per week.<br>*Educational component:* 6 weekly interactive group (up to 6 persons) sessions, each lasted 1 hour; and two booster sessions (at 3 and 6 Months) providing support to participants to evaluate their PA levels and barriers.<br>A WARA booklet was provided to participants, describing the importance of walking, strengthening exercise, reducing SB, and a healthy diet for health benefits.<br><br>**Control group –** 1-hour single education group session (up to 6 persons), included topic regarding the importance of physical activity and healthy diet.	The WARA programme was based on the Social Cognitive Theory, focusing on self-efficacy; and incorporated behaviour change techniques, particularly self-monitoring, feedback, and social support.<br><br>The group education sessions aimed to provide social support; increase the participant's awareness and knowledge of their condition, and encourage PA increase. Therefore, the participant's self-efficacy increase.<br><br>Setting goal of step-count, using pedometer and PA diary, facilitated self-monitoring with feedback from the pedometer, thus increased individual motivation to achieve behaviour change.<br><br>The WARA booklet provided health information which further increased the participant's knowledge and awareness (self-efficacy) of self-management and PA for RA.
**Harris (2018)**	(Protocol)<br>**Pedometer-plus-nurse-support group** – Pedometer and written instructions for a 12-week walking intervention, based on the participant's usual step-count provided. In addition, 3 PA consultations with a practice	(Adaptations)<br>1. Nurses and participants adapted and tailored step count target to individual circumstances, e.g., adjustments were made to the intervention to accommodate religious observances, such as Ramadan and Christmas;	The intervention resources used behaviour change techniques (BCTs).<br><br>3 PA consultations with the practice nurse were divided into 3 stages – First steps, Continuing the changes, and Building lasting habits. They

For peer review only

Supplementary file 5. Delivery and mechanisms_27.05.21

	nurse, individually or as a couple.<br><br>**Pedometer-alone group –** a pedometer, and a 12-week pedometer-based walking programme, posted to the participants. The programme was based on the participant's baseline step-count. On study completion (1 year from baseline), participants in this group were offered a single practice nurse PA consultation.<br><br>**Control group** – No PA intervention. They were offered to choose either receiving a pedometer and the written 12-week pedometer-based walking programme, by post, or as part of a single practice nurse consultation.	during illness; and changes in weather.<br>2. Nurses adapted participant's preferences for interventional materials when tailoring advice, e.g., counting walking by time instead of step-count; whether to use the optional handouts or not.<br>3. Not all participants altered their walking targets; some might have decreased PA level as the target.	included motivational interviewing, health information about PA, suggestions to increase PA, action planning, goal setting, self-monitoring, relapse prevention, which aimed to effect positive changes in participant's step count, PA and SB times; thus longer-term changes in walking habits and health benefits.<br><br>The patient handbook provided the same information as in the nurse consultations.<br><br>Step count diary provided suggestions and instruction for the 12 weeks walking programme. Participants could set goals, self-monitor with feedback from pedometer to increase step count.
**Lakerveld (2012)**	(Protocol)<br>**Intervention group** – Each participant was free to choose the own target lifestyle component(s) (smoking, physical activity or diet). Nurse practitioner provided the CBP to increase participant's motivation and ability to change their dietary pattern, physical activity or smoking behaviour, maximum of 6 individual 30-minute counselling sessions (weekly then reduced to every 2-3 weeks, for 2-4 months); then 3-monthly telephone booster sessions for 12 months. The total intervention period, including booster calls, will be 16 months. The MI and PST counselling methods were used.<br><br>**Control group** – Received written information about their risk of developing T2DM and CVD, and brochures of health guidelines regarding physical activity, healthy diet, and smoking cessation.	(Adaptations)<br>Actual intervention duration is unclear: The number of sessions and schedule described in the results report (Lakerveld et al., 2013) matched the protocol; but the report stated the intervention generally lasted up to 6 months.	The cognitive behavioural programme (CBP) applied the Theory of Planned Behaviour (TPB) and the theory of self-regulation, with 2 counselling techniques - Motivational interviewing (MI), and problem-solving treatment (PST).<br><br>A nurse practitioner used MI to explore the participant's attitude and intention to make lifestyle behaviour change, then resolve the ambivalence between the goal and the actual situation. Afterwards, the nurse practitioner used PST to prompt the participant to find solutions for barriers and reinforcing perceived control for behaviour change. When setting new goals was needed, the same process would be started again.<br><br>The nurse practitioner guided the participant to gradually increase the sense of mastery over difficulties and be more active in planning and

For peer review only

Supplementary file 5. Delivery and mechanisms_27.05.21

			implementing activities.
**Lane (2010)**	Reported as actually delivered interventions.	**Intervention group** – Participants answered a question about the stages of change at baseline. The answer determined either both intervention booklets or just one of them to be posted. The booklets provided information on physical activities and motivation to change, tailored to the participant's readiness to change.<br><br>**Control group** – Received a healthy eating and nutrition booklet, developed by the Irish Heart Foundation, An Bord Bia and the Health Promotion Unit, by post, as placebo treatment.	The tailored intervention applied the trans-theoretical model (TTM), which posits that individuals move through stages of change while learning and adopting new behaviours.<br><br>The intervention consisted of two print booklets, specific to the initial and later stages of motivational readiness. The booklets were adapted for Irish women to promote physical activity, which were broadly based on the TTM model.<br><br>The booklets contained information and structured approaches and strategies, designed to alter self-efficacy, social support, outcome expectancy and barriers to physical activity, tailored to the individual's readiness to change and may subsequently modify physical activity behaviour.
**Matson (2018)**	(Protocol)<br>**STAND intervention** – consisted of 6 health coaching sessions provided by a trained Health Coach, an educational information workbook, SB feedback charts, and a Jawbone UP band.<br>*6 health coaching Sessions:* 2 in-person sessions (first 2 weeks, 45-60 minutes each), providing and explaining the workbook, feedback chart, and Jawbone UP wristband to participants; discussing tailored reminder strategies and setting goals and action plan.<br>After that, *4 bi-weekly phone calls:* (20-40 minutes each) from the Health Coach, to review progress on goals and action plans, problem-solve barriers, use the workbook to guide participants on different types of reminder.<br><br>Based on data from participant's activPAL wear	Delivered as intended.	I-STAND intervention was based on behavioural theories, including social cognitive theory, the ecological model, and habit formation theory.<br><br>Health coaching sessions focused on using different types of reminders, building self-efficacy through motivational interviewing, problem-solving barriers, and setting personalised action plan and graded goals. (Social cognitive theory, habit formation theory)<br>The workbook and coaching sessions included social support, social environment and norms, evaluating participant's environment, to consider the possible changes. (Ecological model).<br>The wrist-worn Jawbone UP band device vibrated every 15 minutes of inactivity. This served as an outward reminder strategy for

For peer review only

Supplementary file 5. Delivery and mechanisms_27.05.21

	at baseline, SB feedback charts 1 Week, and 6 Week were provided to participants.<br><br>**Healthy Living Control group** – 1 in-person health coaching session: Participants were provided a health education workbook containing topics about ageing and instructed to work on 1 topic every 2 weeks using a goal-setting worksheet.<br><br>Every 2 weeks, participants received a check-in letter and asked to complete and return a review progress form.		disrupting the habitual SB, to promote behaviour change and new habits of taking breaks from sitting (habit formation theory).
**Matthews (2016)**	(Protocol)<br>**Walk Well intervention –** 12-week community-based walking programme, consisted of 3 physical activity consultations with a walking advisor; aimed to increase walking by 30-minutes on at least 5 days per week. Participants were provided with education booklets, a pedometer and step diary.<br><br>**Waiting list control group** – were advised to continue with their daily activity for 12-weeks, following which they were invited to participate in the Walk Well intervention.	(Adaptations)<br>1. Some participants experienced difficulty in reading the pedometer and recording step counts in the diary, thus adapted the diary to an alternative "tick box" to indicate having walk(s).<br>2. The physical activity consultations were refined and streamlined to focus on the core components, and flexible options of additional behaviour change techniques for adults with intellectual disability.<br>3. Walking groups were not planned, but expected by some participants, thus arranged by the care centres and carers.	Walk Well was based on the Social Cognitive theory and Trans-theoretical Model.<br><br>The PA consultations method focused on 4 core behaviour change techniques: goal setting; self-monitoring; developing self-efficacy; and mobilising social support. Furthermore, the walking advisor tailored the use of additional behaviour change techniques according to the participant's needs. The aim was autonomy and motivation of the participants to lead a more active lifestyle.<br><br>Input and engagement from carers provided social support from them directly, and their arrangement for group walks among participants.<br><br>The education booklets with visual images and appropriate text provided and reinforce health information.<br><br>Pedometer and step diary complemented the PA consultation, to motivate the participant to set goals and self-monitor step count.

For peer review only

Supplementary file 5. Delivery and mechanisms_27.05.21

**Poston (2013)**	Reported as actually delivered interventions.	Participants were recruited in early 2nd trimester (>15+0 weeks to <17+6 weeks' gestation) to allow adequate time for the intervention programme that was planned to end at each participant's 27+0 and 28+6 weeks' gestation.<br><br>All women attended routine antenatal care appointments and received advice regarding diet and physical activity (PA) in accordance with local policies, which draw on UK NICE guidelines.<br><br>**Intervention group –** participants attended a one-to-one appointment with the HT, provided with a pedometer, a logbook for setting goals and self-monitoring, and a DVD of exercise regime for pregnancy. After that, 8 weekly group sessions from approximately 19 weeks' gestation. The programme included dietary advice choosing low GI food and reducing saturated fats, and increasing daily PA level during pregnancy safely.<br><br>**Control group** – standard care, with additional appointments with the study midwife at 27+0 - 28+6 and 34+0-36+6 weeks', where possible coinciding with routine antenatal visits.	The intervention was based on the Control Theory, and Social Cognitive theory.<br><br>Participants were provided with a pedometer, logbook, an exercise DVD, to set, self-monitor, and achieve SMART (Specific, Measurable, Achievable, Relevant, and Time Specific) goals for diet and PA, using self-regulation techniques from the Control Theory.<br><br>The group sessions facilitated self-identification of benefits and barriers to behaviour change, which facilitated self-efficacy, and provided social support.
**SPH HKU (2017)**	Reported as actually delivered interventions.	**PA group** – received 4 group sessions: 2.5-hour interactive knowledge and motivation enhancement core session at baseline, a 1.5-hour experience sharing booster session at 3 Months, 2.5-hour tea gathering family session at 6 Months, and a Holistic Health session at 1 Year. 16 monthly/bi-weekly health-related text messages to mobile phone for knowledge enhancement and as reminders till one year after baseline.<br><br>**Control group** – received the same intervention framework and methods and the same number and duration of sessions, about Healthy diet.	The PA group intervention was guided by the Health Action Process Approach (HAPA), which proposes motivation, goal setting and planning enhance intention, thus promote its conversion to action. The intervention aimed to enhance knowledge, self-efficacy, and motivation in relation to practising ZTEx<br><br>The conceptual framework proposed that the participants pass the intervention information positively and encourage their family to practise the actions together. Through these family actions and communication, the wellbeing and

For peer review only

Supplementary file 5. Delivery and mechanisms_27.05.21

		Fidelity evaluated but not reported.	harmony of the family were enhanced.<br><br>The strategies included:<br>1. Introducing information on the consequences of physical inactivity, obesity and ZTEx (risk perception);<br>2. Enhancing skills and confidence in the ability to do ZTEx (exercise self-efficacy);<br>3. Associating the health behaviour to the positive outcomes of the trainees (outcome expectations); and<br>4. Introducing cognitive dissonance, i.e., a discrepancy between participants' belief (including a pledge to eat) and behaviour (failure or potential failure to act) to promote intrinsic motivation to change behaviours.<br><br>The mechanism of changes for the Healthy diet intervention (control) was the same, but focusing on healthy diet only.
**Spittaels (2007)**	Reported as actually delivered interventions.	**Tailored information and reinforcement emails group:**<br>*Tailored advice:* Participants completed a questionnaire about their PA and psychosocial determinants on the study's intervention website; subsequently, the tailored advice containing normative PA feedback and suggestions to increase PA levels were produced from it. Participants having intentions to increase PA levels were encouraged to make an action plan.<br><br>*Emails:* After receiving the first tailored advice, participants received regular emails (5 emails in 8 weeks), which asked participants to identify their current stages of change, then referred to a corresponding website with personalised information to encourage behaviour changes.	According to each individual's stage of changes, the tailored advice was provided to participants based in Transtheoretical model. The content applied the constructs of Theory of Planned Behaviour, i.e., intentions, attitudes, self-efficacy, social support, knowledge, benefits and barriers to physical activity.<br><br>Participants indicated with positive intentions to increase their PA levels in the online questionnaire were then encouraged by the website to make a personal action plan to implement behaviour changes.<br><br>Reinforcement emails assessed and followed the participant's stage of change, then directed the participant to pertinent online advice to further encourage behaviour changes.

For peer review only

Supplementary file 5. Delivery and mechanisms_27.05.21

		**Tailored information group:** Participants received the tailored advice online but did not receive reinforcement emails.<br><br>**Standard advice (Control):**<br>Participants received standard physical activity advice from a website, based on information presented to the other 2 groups, but not individually-tailored, e.g., the benefits of PA, current public health recommendations, the difference intensity PAs, and suggestions to be more physically active.	
**Stathi (2019)**	Reported as actually delivered interventions.	Activators attended a 2-day training course, and received an intervention delivery manual. They were trained on the protocol for types and frequency of interactions with the participants; also encouraged to be flexible according to individual needs.<br><br>Each participant was invited to attend a 6-month programme:<br>*Motivation stage (first 2 weeks)* – 2 one-to-one meetings with an activator to support motivation, build rapport, review local activities, and consider and address any barriers to participation.<br>*Action stage (1-3 Months)* – ≥3 visits to local initiatives with the activator.<br>*Maintenance stage (3-6 Months*) – Support provided by telephone, and ≥2 further visits with the activator to encourage the participant to attend local activities independently.<br>Participants could engage in a wide range of activities at the Action and Maintenance stage, e.g., bowling, ballroom dancing, lunch clubs, walking groups, and art classes. 2 social events were organised for all participants and activators to facilitate within group support and encourage more local engagement.	Intended processes of behaviour change during the three stages of the ACE intervention followed the principles of Self Determination Theory, to facilitate the participant's developing autonomous motivation, confidence, and competence for getting out and about.<br><br>In the Motivation stage, the participant engaged in social support from the activator, understood the process, and explored and enhanced motivation for actions. In Action stage, the participant made plans with the activator to try out interested activities and monitored progress. In Maintenance stage, the participant was encouraged to continue with the activities more independently, while the support from the activator was reduced.<br><br>It was shypothesised that participants in the ACE intervention would attend more out-of-house activities, and better motivation to lead an active lifestyle in the long term.

For peer review only - http://bmjopen.bmj.com/site/about/guidelines.xhtml

Supplementary file 5. Delivery and mechanisms_27.05.21

		The control group received written materials about local initiatives only, but were offered the intervention at the end of study period.	
**Williams (2019)**	(Protocol)<br>**Walk This Way intervention** – amended from the Walk, Address sensations, Learn about exercise, encourage exercise behaviour for persons with schizophrenia spectrum disorders ('WALC-S') programme<br>*Initial group education session:* 5-10 participants; participants were provided a pedometer for self-monitoring and calendar for recording; setting goals for increasing habitual walking level.<br>*Continuing support and coaching:* every 2 weeks (20-30 minutes), an assigned coach met the participant to review the participant's walking calendar, identify and address barriers and facilitators to increase PA and decrease SB, and provide motivational support to the participant to reach.<br>Weekly walking group: the coaches arranged and invited all participants to an optional weekly group walk (2 hours).<br><br>**Control condition** – Received written information on the benefits of increasing activity levels. This advice was given in accordance with the NHS Foundation Trust policy on physical health.	Delivered as intended.	The Walk this Way intervention employed the COM-B model of behaviour change principles to address capability, opportunity, and motivational barriers to reducing SB and increasing PA.<br><br>The Initial education session aimed to enhance motivation and self-efficacy to make behaviour change.<br><br>Health coaching sessions used the REACH© model of coaching, emphasising individual's accountability involves thinking, feeling, and doing to achieve the self-identified goals. Health information of PA, support and motivation for goal attainment were provided to facilitate the participant to increase walking into daily routine independently.<br><br>The participant's walking goal was set with SMART (Specific, Measurable, Attainable, Realistic and Timely), self-monitored by pedometer and calendar; the step count and factors affecting attainment were discussed with the coach.<br><br>Weekly regular group walk was optional, which provided social support to the participants.

Keys: * = Data from associated publications; ACE = Active, Connected, Engaged intervention; BCG = Behaviour change group; BMI = Body Mass Index; C = Control group; CBP = Cognitive behavioural programme CVD = Cardiovascular disease; DESMAND = Diabetes education and self management for ongoing and newly diagnosed; DM = Diabetes Mellitus; FU = Follow-up; GI = Glycaemic Index; GP = General practitioner; HC = Health centre; HCP = Health care provider; HT = Health trainer; I = Intervention group; LSA = Lifestyle advisor; MVPA = Moderate-to-vigorous physical activity; PA = Physical activity; PREPARE = Prediabetes risk education and physical activity recommendation and encouragement; PT = Physiotherapist; SD = Standard deviation; SMART = Specific, Measurable, Achievable,

For peer review only

Supplementary file 5. Delivery and mechanisms_27.05.21

Relevant, and Time Specific; STAND = Sedentary Time ANd Diabetes; SWO = Standard website-only; TEG = Traditional exercise group; TTCW = Tailored telephone counselling plus website; WARA = Walk for Rheumatoid Arthritis; ZTEx = Zero Time Exercise

For peer review only

Supplementary file 6. Implementation data_27.05.21

Study (Year)	Fidelity (delivering the intervention as per protocol)	Recruitment (recruiting participants and sites)	Retention (participants remaining in the intervention or control/usual care group)	Reach (dose received and participant engagement)
**Adams (2012)**	Only qualitative data reported.	10 clusters invited.<br>7 clusters recruited (needed active membership n≥12).	I: n= 40 (85.1%)<br>C: n= 24 (85.7%)<br><br>Primary reasons for leaving the study:<br>55% (6/11) Having to wear the activity monitors.<br>18% (2/11) Time commitment too great.<br>18% (2/11) Had not understood length of study.<br>9% (1/11) Went out of town unexpectedly.	23/40 (58%) participants always used 2 of 3 intervention elements<br><br>Overall satisfaction with the programme (Likert scale, 1= not at all, 5= very satisfied):<br>39.5% (17/43) participants rated very satisfied (highest %).<br>97.7% (42/43) participants rated at least "3= somewhat" or above.
**Albright (2015)**	5% (80/1586) recorded telephone counselling sessions evaluated against a checklist of the essential intervention components:<br><br>88% fidelity over the 12-month intervention to the essential intervention components.<br><br>96% calls covered barriers to MVPA discussion.<br>97% calls covered assessing participant's previous MVPA goal.<br>100% calls covered setting the participant's next MVPA goal.<br><br>The two components most	Community recruitment:<br>272 via adverts, e.g., magazines, radio stations;<br>170 randomised,<br><br>Kaiser Permanente recruitment:<br>3844 Postcards sent out;<br>1176 calls made;<br>419 interested in joining;<br>141 randomised.	I: n= 115 (74.7%)<br>C: n= 127 (80.9%)<br><br>Most frequent reasons for failure to complete the intervention:<br>13% Pregnancy.<br>9.5% Too busy.<br>6.1% Discontinued participation, no given reason.<br>3.5% Family/job issues.	TTCW group:<br>90.4% of the participants receiving ≥13 of the 17 scheduled calls.<br><br>78.3% of the participants viewed the website at least once.<br><br>75% of participants set incremental MVPA goals with a health educator during the counselling sessions over the 12-month intervention period.<br><br>Level of achieving set MVPA goals in the 3 phases among all participants:<br><br>High level (≥100% of MVPA goal achieved or exceeded):<br>40.6% of the time during Phase 1 (weekly calls).<br>39.9% of time during Phase 2 (biweekly

For peer review only

Supplementary file 6. Implementation data_27.05.21

	frequently not delivered:<br>Pedometer steps (asked in 68.8% calls).<br>MVPA resources (offered in 80% calls).			calls).<br>42.0% of time during Phase 3 (monthly calls).<br><br>Moderate level (50-99% MVPA goal achieved):<br>23.5% of the time during Phase 1.<br>28.4% of the time during Phase 2.<br>21.1% of the time during Phase 3.<br><br>Low level (0-49% MVPA goal achieved):<br>35.8% of the time during Phase 1.<br>31.7% of the time during Phase 2.<br>36.9% of the time during Phase 3.
**Benedetti (2020)**	Checklist to assess implementation, including programme fidelity, instructor knowledge, classroom, schedule, participants' attention and attendance:<br>All analysed items achieved an average of 98% fidelity.	2 of 5 health districts in Florianopolis were interested in participating, consisting 20 of 50 HCs.<br>6 HCs were interested, and had the physical structure and human resources to offer the programmes, thus were recruited.<br><br>4,071 older adults across the 6 HCs;<br>24.2% (985) individuals were considered eligible;<br>11.5% (114) of eligible participants recruited.	Post-intervention (3 months):<br>BCG: n= 18 (50%)<br>TEG: n= 33 (63.5%)<br>C: n= 23 (88.5%)<br><br>6 months:<br>BCG: n= 17 (47.2%)<br>TEG: n= 32 (61.5%)<br>C: n= 21 (80.8%)<br><br>12 months:<br>BCG: n= 13 (36.1%)<br>TEG: n= 28 (53.8%)<br>C: n= 17 (65.4%)	Overall, 49% of participants attended at least 75% of all sessions, with disengagement occurring mostly in the first three weeks of the study (42%).<br><br>Both intervention groups showed relatively high disengagement rates (BCG 50% vs. TEG 37%) with individuals in the BCG presenting lower rates of overall attendance (27% vs. 47%).
**Berendsen (2015)**	Fidelity:<br>24/25 interviewed HCPs were trained in Motivational Interviewing, and applied MI with the participants.<br><br>100% PTs made an exercise plan with the participants.	30 clusters invited.<br><br>411 participants recruited (with 2 to 30 subjects per cluster, 76.9% of participants referred by the GP).<br><br>Eligibility based on baseline data:<br>- 48.9% met the inclusion criteria.<br>- 10.0% healthy BMI/no comorbidities.	28 clusters remained<br><br>Participants:<br>I: n= 196 (79.4%)<br>C: n= 126 (76.8%)<br><br>From recorded data, the main reasons of drop-outs were health issues (31.5%),	**% = median of attended / planned number of meetings:**<br><br>LSA meetings:<br>I: 50.0%; C:66.7%<br>PT group meetings:<br>I: 47.1% to 61.5%; C: 0% (planned n= 0)<br>PT individual meetings:<br>I: 0% (planned 6 to 7); C: 33.3%

For peer review only - http://bmjopen.bmj.com/site/about/guidelines.xhtml

Supplementary file 6. Implementation data_27.05.21

	84.8% of the participants set exercise goals or made an exercise plan with an HCP. 79.9% Exercise plans or goals were made with PT, if participants attended any individual meeting with a PT.<br><br>5/6 dietitians made nutritional plans with the participants. 73.9% of the participants made set nutritional plan or goals with an HCP. 91.7% of the plans or goals were made with the dietitian, if participants attended any individual meeting with a dietitian.<br><br>96.9% participants reported LSA had explained the intervention clearly at the beginning.<br><br>226 participants (from both IG and CG) completed a questionnaire after 12 months:<br>40.7% Reported the LSA had explicitly concluded the intervention.<br>41.2% Reported the intervention was not concluded.<br>18.1% Did not know.<br><br>Dose Delivered:<br>1 PT in start-up programme	- 16.8% higher weight-related risk than the target population.<br>- 24.3% of participants' eligibility could not be checked.	and personal reasons (10.1%).	Dietitian group meetings:<br>I: 42.9%; C: 28.6%<br>Dietitian individual meetings:<br>I: 33.3%; C: 133.3%<br><br>Satisfaction (on scale of 1–10, 10 is best):<br>Mean range (across meeting types):<br>I: 7.1 – 8.0<br>C: 7.1 – 7.3<br>Overall programme (Mean (SD)):<br>I: 7.7 (1.5)<br>C: 7.1 (1.8)

For peer review only

Supplementary file 6. Implementation data_27.05.21

	only planned group meetings with all HCPs, instead of the individual meetings intended per protocol.<br><br>4 dietitians typically offered individual meetings with participants, as per protocol. The other 4 dietitians only planned individual meetings according to participant's preference.			
**Biddle (2017)**	Not reported	Not reported	*I: n= 41 (43.6%)<br>*C: n= 68 (73.1%)<br><br>Reasons for failure to complete the intervention or loss to follow-ups:<br>24.5% (23/94) Did not receive allocated intervention in the intervention group.<br>16% (30/187) No longer want to participate.<br>13.4% (25/187) Failed to attend FU appointment.	23/94 (24%) allocated to intervention group did not attend the structured education workshop.<br>45/94 (47.9%) took part in Week 6 phone progress reviews<br><br>26/31 (84%) participants used the accelerometer daily initially, but this fell to 13/31 participants at 6 weeks.<br><br>25/31 (81%) participants felt the accelerometer as helpful at 6 weeks.<br><br>Workshop feedback:<br>Behaviour change plans for future (6 weeks):<br>4/38 (11%) referred to strategies to sit less<br>17/38 (45%) planned for physical activity<br>Others referred to desired health outcomes<br><br>"Best bits" of the workshop (mentioned most frequently): 1. information on diabetes; 2. the atmosphere of the workshop; 3. Receiving personal data on

Supplementary file 6. Implementation data_27.05.21

				sitting levels and health.<br><br>Behaviour change strategies attempted as reported by participants:<br>18 strategies mentioned to sit less and 8 strategies to move more.
**Blunt (2018)**	Only qualitative data reported	***How recruited participants heard about the study:**<br>51 (45%) from posters or handouts;<br>28 (25%) received an email from the study site advertising the project;<br>15 (13%) from an in-person study recruiter;<br>12 (11%) referred by their health care provider (HCP) and/or HCP team;<br>6 (5%) by word of mouth;<br>1 (1%) other unspecified methods<br>Five did not specify how they heard about the study	*6 months:<br>I: n= 44 (74.6%)<br>C: n= 46 (78.0%)<br><br>3.4% (I: n= 2) Did not attend any session<br>6.8% (I: n= 5, C: n= 3) Personal/health reasons<br>3.4% (I: n= 3, C: n= 1) Time commitment<br>5.9% (I: n= 2, C: n= 5) No longer interested<br><br>*12 months:<br>I: n= 37 (63%)<br><br>*18 months:<br>I: n= 35 (59%)	***Attendance:**<br>5% attended no sessions;<br>17% attended 1 session;<br>10% attended 2 sessions;<br>20% attended 3 sessions;<br>48% attended all 4 sessions.<br>Across all sites, 40 participants (68%) were classified as programme completers.<br>Among participants who completed the intervention programme, 30% attended 3 in-person sessions, 70% attended all 4 sessions.
**Elramli (2017)**	Not reported	320 participants invited:<br>106 (33.1%) did not respond;<br>122 (38.1%) ineligible;<br>92 (28.8%) assessed for eligibility;<br>76 (23.8%) randomised	3 months:<br>I: n= 36 (92.3%)<br>C: n= 26 (70.3%)<br><br>6 months:<br>I: n= 37 (94.9%)<br>C: n= 22 (59.5%)	Intervention attendance:<br>26 (66.7%) participants attended all 8 education sessions (6 sessions and 2 booster sessions)<br>28 (71.8%) attended 6 sessions<br>71.8 % attended the first booster session<br>76.9% attended the second booster session<br><br>Control group attendance:<br>21 (56.8%) participants attended the single group education session
**Harris (2018)**	Nurse session attendance and session content delivered	11,015 people invited to participate;<br>6,399 did not respond;	3 months:<br>Postal: n= 335 (98.8%)	Diary returned:<br>Postal: 268/339 (79%)

For peer review only - http://bmjopen.bmj.com/site/about/guidelines.xhtml

Supplementary file 6. Implementation data_27.05.21

	recorded by the nurses after each session.<br><br>Fidelity to content delivered was high in all sessions; the mean number of items delivered in session one was 11 (range 10–11); six (range 5–6) in sessions 2 and 3.<br><br>Duration of sessions reported by nurses and measured from records were not very far from the recommendation (±≤30% difference maximum).	548 were excluded as a result of self-reported PA guideline achievement; 1,023/10,467 (10%) were randomised.	Nurse: n= 335 (96.8%)<br>C: n= 335 (99.1%)<br><br>12 months:<br>Postal: n= 319 (94.1%)<br>Nurse: n= 317 (91.6%)<br>C: n= 329 (97.3%)<br><br>4.3% (Postal: n=15/339, Nurse: n=25/346, C: n=4/338) Withdrawn<br>1.4% (Postal: n=5/339, Nurse: n=4/346, C: n=5/338) Not able to be contacted	Nurse: 281/346 (81%)<br><br>Pedometer use (every day or most days) during 12-week intervention:<br>Postal: 238/294 (81%)<br>Nurse: 269/303 (89%)<br><br>Attending nurse sessions:<br>255/346 (74%) attended all three sessions.<br>258/263 (98%) attended session 3, and reported still using the pedometer and diary every day or sometimes.
**Lakerveld (2012)**	Only qualitative data reported	8,193 people of 12 general practices were invited according the age (30-50 years) and absence of DM or CVD.<br><br>2,401 (29.3%) responded positively;<br>1,186 (14.5%) declined;<br>921 (11.2%) of those who accepted invitation met the waist circumference inclusion criterion;<br>772 (9.4%) attended screening at clinic and consented;<br>622 (7.6%) fully eligible and randomised.	End of intervention (6 months):<br>I: n= 267 (85.0%)<br>C: n= 269 (87.3%)<br><br>12 months:<br>I: n= 249 (79.3%)<br>C: n= 253 (82.1%)<br><br>24 months:<br>I: n= 236 (75.2%)<br>C: n= 244 (79.2%)<br><br>Reasons for loss to follow-up:<br>15.1% (I: n=42/308, C: n=52/314) Unable to attend<br>3.7% (I: n=9/308, C: n=14/314) Withdrew consent<br>1.1% (I: n=5/308, C: n=2/314) Became pregnant<br>1.3% (IG n=5/308, C:	*207 (66%) participants received at least 1 face-to-face session, 78% of them were content with the sessions.<br><br>The median number of attended sessions was 2 (out of a max of 6).

For peer review only

Supplementary file 6. Implementation data_27.05.21

			n=3/314) Unable to contact<br>0.2% (I: n=1/308, C: n=0/314) Died<br>1.4% (I: n=2/308, C: n=7/314) Diagnosed type 2 DM	
**Lane (2010)**	Not reported	11,205 women registered for the Women's Mini Marathon completed a survey about their PA habits.<br><br>Consented respondents were followed up 2 months and 6 months afterwards respectively:<br>2,020 of them provided records of PA changes at both follow-ups;<br>414 of them were identified as having relapsed to insufficient levels of PA and invited to participate in the trial;<br>176 consented to participate.	Follow-up response rate (end of trial at 6 Weeks):<br>I: n= 55 (65%)<br>C: n= 57 (63%)	76% of Intervention group participants responded at 3 Weeks:<br>97% received the booklet(s)<br>90% found the booklet(s) useful<br>50% reported increase in PA levels<br>28.5% felt greater levels of motivation which led to PA increase<br>16% felt they had more knowledge on being active which led to PA increase<br>5% attributed the PA increase to training for the Mini Marathon for the following year<br><br>At end of trial (6 Weeks), receipt and use of materials provided:<br>95% of intervention group participants<br>80% of control group participants
**Matson (2018)**	Not reported	Not reported	*I: n= 29 (100%)<br>*C: n= 25 (80.6%)	Only qualitative data reported
**Matthews (2016)**	Only qualitative data reported	Sample was deemed representative of adults with intellectual disabilities: 91% (n = 93) had mild or moderate intellectual disability.	*End of intervention (12 weeks):<br>I: n= 45 (83.3%)<br>C: n= 43 (89.6%)<br><br>*24 weeks:<br>I: n= 42 (77.8%)<br>C: n= 40 (83.3%)<br><br>Reasons for loss to follow-up:<br>32.4% (I: n=20/54, C: n=13/48) Did not want to	*54 participants were assigned to intervention, and received the intervention.<br><br>*71% took part in all 3 planned face-to-face physical activity consultations.<br><br>*26% took part in 2 consultations<br>*3% took part in 1 consultation

For peer review only

Supplementary file 6. Implementation data_27.05.21

			continue<br>1% (I: n=1/54) Ill-health	
**Poston (2013)**	Goals were set at all group sessions, of which 88% were considered SMART by HTs according to their diaries.	According to information from the Health and Social Care Information Centre (2013), approximately 1:5 pregnant women would be eligible for inclusion.<br><br>473/656 (72%) eligible people declined to participate (43.0% of those who declined were in the lowest quintile for Index of deprivation indicating the most severe deprivation);<br>38% participated.	End of intervention:<br>I: n= 79 (84.0%)<br>C: n= 75 (84.3%)	82/94 (88%) attended at least one group session, and 60 (64%) attended 4 or more.<br><br>42 women (45%) received material from all eight sessions, 6 by full attendance (6%) and 36 when partly/wholly covered by subsequent phone contact.<br><br>Mean of 6.1 (SD 2.6) sessions were attended or partly/wholly covered for the intervention group.
**School of Public Health, HKU (2017)**	Fidelity checks were conducted for every session of the programmes, which ensured the quality of the intervention and the implementation of the key elements in the intervention.	8 participating Integrated Family Service Centres to recruit around 600 eligible parents.<br><br>728 (121.3% of target) randomised.	Trial Core session (baseline):<br>I: n= 357 (92.5%)<br>C: n= 316 (92.4%)<br><br>3 months:<br>I: n= 335 (86.8%)<br>C: n= 306 (89.5%)<br><br>6 months:<br>I: n= 328 (85.0%)<br>C: n= 298 (87.1%)<br><br>End of intervention -12 months:<br>I: n= 309 (80.1%)<br>C: n= 284 (83.0%)<br><br>Reasons for absence from sessions included occupied with other activities, took care of family, illness, could not be contacted, and abroad; the exact number of participants dropped out for each of these reasons cannot	Physical activity group: (386 randomised)<br>357 (92.5%) attended core (1st) session<br>355 (92.0%) attended booster session at 3 months<br>313 (81.1%) attended tea gathering at 6 months<br>281 (72.8%) attended Family Holistic Health session at 1 year.<br><br>Healthy diet group: (342 randomised)<br>316 (92.4%) attended core (1st) session<br>306 (89.5%) attended booster session at 3 months<br>292 (85.4%) attended tea gathering at 6 months<br>268 (78.4%) attended Family Holistic Health session at 1 year.<br><br>Participant's feedback at end of Physical activity programme (on a scale of 0-10, 10 is best) (Mean (SD)):<br>9.0 (1.2) Quality of intervention content<br>9.0 (1.2) Level of utility of the intervention<br><br>100% participants would recommend this

For peer review only

Supplementary file 6. Implementation data_27.05.21

			be ascertained.	intervention programme to their friends and families
**Spittaels (2007)**	Not reported	8,000 employees targeted via 6 worksites using email messages, posters and internal newsletters;<br>570 (7.1%) responded positively;<br>562 (7.0%) returned the baseline questionnaire with the informed consent, and then randomised.<br><br>~65% of participants met the minimal recommendations for physical activity at baseline despite explicit recruitment of inactive participants<br><br>31% participants were female, males comprising the majority of employees in the two biggest worksites for recruitment<br><br>Male participants already had high baseline physical activity scores compared to the general male population (72% vs. 57% meeting the recommendations), whereas female participants were more representative of the population (47% vs. 48% meeting the recommendations).	End of intervention:<br>Tailored advice+emails: n= 116 (66.7%)<br>Tailored advice: n= 122 (69.7%)<br>C: n= 141 (79.7%)	Recalled having received the tailored advice (% participants):<br>97% Tailored advice+emails group<br>94% Tailored advice group<br>53% Control group<br><br>Tailored advice+emails group satisfaction (% participants):<br>92% Received at least 3 of the 5 reinforcement emails<br>77% Read them completely<br>87% Satisfied by number of emails<br>86% Satisfied by frequency of emails<br>45% Felt emails were useful<br>33% Reported behavioural changes
**Stathi (2019)**	Not reported	2,000 mailed invitations were delivered in the target areas resulting in 230 responses from potential participants and activators (response rate 11.5%).<br><br>ACE participants: 154 (7.7%) requests for information packs. 65 (3.3%) people returned reply forms. 40 (2.0%) recruited.<br><br>Activators: 76 (3.8%) requests for information packs. 15 (0.8%) recruited after completing the training.	End of intervention:<br>Activator: n= 15 (100.0%)<br><br>Participants:<br>I: n= 19 (86.4%)<br>C: n= 13 (76.5%)<br><br>Reasons for dropping out prior to final measures:<br>7.7% (3/39) Ill-health<br>5.1% (2/39) Carer commitments	All participants who completed the intervention engaged with their activator at least 7 times as planned.<br><br>Of the 3 participants who dropped out: 2 met their activator less than 5 times but were contacted regularly by phone.

Supplementary file 6. Implementation data_27.05.21

			2.6% (1/39) Lack of time<br>2.6% (1/39) Moving to a different city	
**Williams (2019)**	Not reported	215 eligible service users contacted by letter and phone;<br>71 not interested;<br>104 not contactable;<br>40 (18.6%) recruited.	I: n= 16 (80.0%)<br>C: n= 17 (85.0%)	13 (65%) received intervention:<br>5 did not engage with intervention;<br>2 did not engage with intervention after education session.

Keys: * = Data from associated publications; ACE = Active, Connected, Engaged intervention; BCG = Behaviour change group; BMI = Body Mass Index; C = Control group; CVD = Cardiovascular disease; DM = Diabetes Mellitus; FU = Follow-up; GP = General practitioner; HC = Health centre; HCP = Health care provider; HT = Health trainer; I = Intervention group; LSA = Lifestyle advisor; MVPA = Moderate-to-vigorous physical activity; n = number of persons; PA = Physical activity; PT = Physiotherapist; SD = Standard deviation; SMART = Specific, Measurable, Achievable, Relevant, and Time Specific; TEG = Traditional exercise group; vs = versus

For peer review only

Supplementary file 7_SB results from RCTs associated with included PEs_27.05.21

**Study (Year)** Number of participants randomised	**Unit of outcome**	**Outcome measure(s) for SB**	**Intervention group Baseline**	**Control group Baseline**	**Intervention group End of intervention[a]**	**Control group End of intervention[a]**
**Adams (2012)**<br><br>I: 47 C: 28	1. Mean % of SB time per day (SD)<br>2. Mean sitting hours per week (SD)	1. Accelerometer<br><br>2. IPAQ	(n= 40)<br>SB: 47.42% (10.77)<br><br>Sitting time: 57.99 (29.70)	(n= 24)<br>SB: 50.7% (13.78)<br><br>Sitting time: 45.18 (34.88)	(n= 40)<br>SB: 49.16% (10.23)<br><br>Sitting time: 46.00 (28.91)	(n= 24)<br>SB: 50.39% (14.92)<br><br>Sitting time: 40.33 (40.68)
**Albright (2015)**<br><br>I: 138 C:140	Mean sitting hours per day (SD)	Active Australia Survey	Traveling to/from work: 1.19 (0.71)*<br>While at work: 2.02 (2.18)<br>Watching TV: 2.05 (1.33)<br>Using a computer at home: 1.27 (0.98)<br>Other leisure time (movies, dining out): 1.38 (1.01)<br>While holding/feeding baby: 2.93 (1.78)	Traveling to/from work: 1.41 (0.82)*<br>While at work: 2.52 (2.5)<br>Watching TV: 1.91 (1.36)<br>Using a computer at home: 1.41 (1.18)<br>Other leisure time (movies, dining out): 1.31 (1.05)<br>While holding/feeding baby: 3.20 (2.08)	Not published	Not published
**Benedetti (2020)**<br><br>BCG: 36<br>TEG: 52<br>C: 26	Baseline: Mean SB minutes per week (SD)<br><br>End of trial: Mean SB minutes per day (SE)	Accelerometer	BCG: 498.5 (113.6)<br>TEG: 529.8 (107.3)	522.8 (86.7)	Change between baseline and end of intervention (3 months):<br>BCG: -14.3 (56.3) [c]<br>TEG: -4.1 (62.2) [c]<br>*Change between baseline and end of trial (12 months):*<br>*BCG: -10.9 (59.9)* [c]<br>*TEG: 4.2 (78.6)* [c]	Change between baseline and end of intervention (3 months):<br>-25.6 (77.9) [c]<br>*Change between baseline and end of trial (12 months): -26.7 (68.3)* [c]
**Berendsen (2015)**<br><br>I: 247 C: 164	Not published	Accelerometer	Not published	Not published	Not published	Not published

For peer review only

Supplementary file 7_SB results from RCTs associated with included PEs_27.05.21

**Biddle (2017)**<br><br>1: 94 C:93	Mean SB hours per day (95% CI)	1. Actigraph (worn on waistband)<br>2. ActivPAL (worn on thigh)	Actigraph (n= 76): 10.83 (10.50, 11.17) [c]<br>ActivPal (n= 60): 8.91 (8.59, 9.24) [c]	Actigraph (n= 80): 11.01 (10.76, 11.26) [c]<br>ActivPal (n= 57): 9.02 (8.73, 9.30) [c]	Outcomes not measured at end of intervention (6 Weeks).<br>*Change between baseline and end of trial (12 months)*<br>Actigraph *(n= 38): -0.29 (-0.75, 0.17)* [c]<br>*ActivPal (time change, n=32): 0.64 (0.13, 1.16)* [c]	Outcomes not measured at end of intervention (6 Weeks).<br>*Change between baseline and end of trial (12 months)*<br>*Actigraph n= 49): -0.23 (-0.60, 0.14)* [c]<br>*ActivPal (n=29): 0.58 (0.06, 1.09)* [c]
**Blunt (2018)**<br><br>I: 59 C:59	Mean sitting minutes per day (SD)	IPAQ	360 (315)	360 (240)	Mean difference between groups (only measured at end of active intervention phase – 6 months): -0.08 (-0.16, -0.006)* [c]	
**Elramli (2017)**<br><br>I: 39 C: 37	Mean SB hours per day (SE)	1. ActivPal<br><br>2. IPAQ	ActivPal SB: 18.0 (0.27) [c]<br>IPAQ weekday sitting: 5.3 (0.31)<br>IPAQ weekend sitting: 5.3 (0.36)	ActivPal SB: 18.5 (0.2) [c]<br>IPAQ weekday sitting: 4.7 (0.41)<br>IPAQ weekend sitting: 4.6 (0.38)	ActivPal SB: 17.2 (0.3) [c]<br>IPAQ weekday sitting: 4.2 (0.33)**<br>IPAQ weekend sitting: 3.9 (0.33)	ActivPal SB: 18.7 (0.41) [c]<br>IPAQ weekday sitting: 5.7 (0.53)**<br>IPAQ weekend sitting: 5.1 (0.63)
**Harris (2018)**<br><br>Postal: 339<br>Nurse: 346<br>C: 338	Mean SB minutes per day (SD, or 95% CI)	Accelerometer	Postal: 614 (71)<br>Nurse: 619 (78)	613 (86)	Mean difference between groups at end of intervention (3 months)<br>Postal versus control: -2 (-12, 7) [c]<br>Nurse versus control: -7 (-16, 3) [c]<br>Nurse versus Postal: -4 (-13, 5) [c]<br>Mean difference between groups at *end of trial (12 months)*<br>*Postal versus control: 1 (-8, 10)* [c]<br>*Nurse versus control: -0.2 (-9, 9)* [c]<br>*Nurse versus Postal: -1 (-10, 8)* [c]	
**Lakerveld (2012)**<br><br>I: 314 C: 308	Mean SB minutes per day (SD)	A subscale of AQuAA	253.7 (146.9) [c]	255.4 (124.5) [c]	Outcomes not measured at end of intervention<br>*End of trial (Month 24): 231.5 (122.2)* [c]	Outcomes not measured at end of intervention<br>*End of trial (Month 24): 233.0 (140.7)* [c]

For peer review only

Supplementary file 7_SB results from RCTs associated with included PEs_27.05.21

**Lane (2010)**<br><br>I: 85 C: 91	Mean sitting time minutes per week (SD)	Frequently used validated questions selected for the trial from other population-level PA interventions.	335.9 (194.9)	310.1 (224.7)	371.4 (170.1)	369.5 (152.6)
**Matson (2018)**<br><br>I: 29 C: 31	Mean sitting time minutes over last 7 days (SD)[b]	ActivPAL	Not published	Not published	Change between baseline and end of intervention (n= 29): -70.1 (104) [b]	Change between baseline and end of intervention (n= 25): 6.5 (69) [b]
**Matthews (2016)**<br><br>I: 54 C: 48	Mean% of time per day spent in SB (SD)	Accelerometer	64.2% (10.5)	66.9% (11.3)	(n= 42)<br>66.4% (10.0) [c]	(n= 40)<br>65.9% (12.0) [c]
**Poston (2013)**<br><br>I: 94 C: 89	Mean SB time minutes per day (SD)	1. Accelerometer<br><br>2. RPAQ	Accelerometer (n= 68): 1165 (91) [c]<br>RPAQ (n= 79): 1009 (187) [c]	Accelerometer (n= 72): 1172 (95) [c]<br>RPAQ (n= 80): 1007 (207) [c]	Accelerometer (n= 36): 1197 (77) [c]<br>RPAQ (n= 56): 1020 (226) [c]	Accelerometer (n= 39): 1175 (86) [c]<br>RPAQ (n= 54): 1068 (177) [c]
**School of Public Health HKU (2017)**<br><br>I: 357 C:316	Mean sitting hours in a working day (SD)	IPAQ-C	4.47 (2.47)*	4.11 (2.38)*	4.3	4.2
**Spittaels (2007)**<br><br>I:<br>Group 1 (tailored advice + email): 116<br>Group 2 (tailored advice): 122<br><br>C: 141	Mean sitting minutes per day (SD)	IPAQ	Group 1:<br>Weekday: 482 (183)<br>Weekend day: 308 (160)<br>Group 2:<br>Weekday: 492 (202)<br>Weekend day: 296 (160)	Weekday: 470 (217)<br>Weekend day: 309 (182)	Group 1:<br>Weekday: 443 (168)<br>Weekend day: 276 (131)<br><br>Group 2:<br>Weekday: 438 (172)<br>Weekend day: 268 (141)	Weekday: 419 (181)<br>Weekend day: 271 (139)
**Stathi (2019)**<br><br>I: 22 C: 17	Mean SB minutes per day (SD, +/ 95% CI)	Accelerometer	681.5 (74.9)	616.2 (112.3)	Change between baseline and end of intervention:	Change between baseline and end of intervention:

For peer review only

Supplementary file 7_SB results from RCTs associated with included PEs_27.05.21

					13.1 (77.2) (-26.6, 52.8)	-8.7 (70.7) (-57.6, 75.1)
**Williams (2019)**<br><br>I: 20 C: 20	Mean SB minutes per day (SE)	Accelerometer	577.2 (9.8)	549.2 (19.1)	End of intervention (17 weeks, n= 16): 520.9 (36.2)*<br><br>*End of trial (6 months, n= 8): 508.2 (19.4)**	End of intervention (17 weeks, n= 17): 637.9 (30.4)*<br><br>*End of trial (6 months, n= 13): 661.2 (33.5)**

Supplementary file 3: Sedentary behaviour measured (at baseline and end of the trial) in the randomised controlled trials associated with the included process evaluation studies

Keys: [a] = Results available from the assessment immediately after the intervention, unless otherwise specified; [b] = unclear if adjusted for covariates; [c] = data were adjusted for covariates; * = p value <0.05 for comparison between intervention and control groups (where reported); ** = p value <0.025 for comparison between intervention and control groups reported as accepted statistical significance ; *Italic font* = End of trial results, if available from publications; AQuAA = Activity Questionnaire for Adolescents & Adults; BCG = Behaviour Change Group; C = Control group; FU = Follow-up; I = Intervention group; IPAQ = International Physical Activity Questionnaire; IPAQ-C = International Physical Activities Questionnaire-Chinese version; n = number of persons included in the analysis; RPAQ = Recent Physical Activity Questionnaire; SB = Sedentary behaviour; SD = Standard deviation; SE = Standard error; TEG = Traditional Exercise Group

For peer review only

Supplementary file 8 characteristics of 24 excluded studies 27.05.21

Study	Reason for exclusion
Ashe 2013	Results of process evaluation not available.
Burton 1995	Ineligible study design: The study did not involve process evaluation.
Cohen 2017	Ineligible study design: The participants were not all assessed at all timepoints throughout the trial. The data from each time point were not obtained from the same sample group throughout the study.
Coll-Planas 2019	Results of process evaluation not available.
Douglas 2019	Ineligible study design: The study is not RCT.
Gray 2018	Sedentary behaviour was not measured in the RCT.
Gummelt 2017	Sedentary behaviour was not measured in the RCT.
Hammerback 2012	Sedentary behaviour was not measured in the RCT.
Harvey 2016	Ineligible study design: The study did not involve process evaluation of exploration of the intervention.
Holt 2019	Sedentary behaviour was not measured in the RCT.
Hsu 2013	Sedentary behaviour was not measured in the RCT.
Jayaprakash 2016	Sedentary behaviour was not measured in the RCT: Sedentary behaviour was measured at baseline, but not throughout the trial as an outcome.
Lai 2019	Ineligible study design: The study was not a RCT.
Maddison 2020	Sedentary behaviour was not measured in the RCT.
McAuley 2013	Ineligible study design: The study did not involve process evaluation.
Orme 2017	Ineligible study design: The evaluation of feasibility did not involve process evaluation or qualitative evaluation.
Rovniak 2014	Sedentary behaviour was not measured in the RCT.
Sazlina 2015	Results of process evaluation not available.
Seguin 2019	Sedentary behaviour was not measured in the RCT.
Sheppard 2016	Sedentary behaviour was not measured in the RCT.
Stevens 2015	Sedentary behaviour was not measured in the RCT.
Thomsen 2016	Ineligible study design: The study did not involve process evaluation or qualitative evaluation.
Thompson 2008	Results of process evaluation not available.
Thornton 2018	Ineligible comparator: The eligible intervention was assigned to the control group, not the experimental intervention group in this study.
Tiedemann 2015	Sedentary behaviour was not measured in the RCT.
van de Glind 2017	Results of process evaluation not available.
van der Wardt 2019	Sedentary behaviour was not measured in the RCT.
Varela-Mato 2016	Ineligible setting: The intervention was delivered at workplace.
Voorn 2016	Sedentary behaviour was not measured in the RCT.
Yeung 2020	Ongoing: Study not completed.
Zabaleta-Del-Olmo 2018	Results of process evaluation not available.

Keys: RCT = Ramdomised Controlled Trial

For peer review only

	SCREENING QUESTIONS		1. QUALITATIVE STUDIES					2. RANDOMIZED CONTROLLED TRIALS					3. NON-RANDOMIZED STUDIES					4. QUANTITATIVE DESCRIPTIVE STUDIES					5. MIXED METHODS STUDIES				
**Authors (Year)**	**S1**	**S2**	**1.1**	**1.2**	**1.3**	**1.4**	**1.5**	**2.1**	**2.2**	**2.3**	**2.4**	**2.5**	**3.1**	**3.2**	**3.3**	**3.4**	**3.5**	**4.1**	**4.2**	**4.3**	**4.4**	**4.5**	**5.1**	**5.2**	**5.3**	**5.4**	**5.5**
Adams (2012)	Yes	Yes	Yes	Yes	Yes	Yes	Yes	Yes	Yes	Yes	No	Yes						Yes	Yes	Yes	Yes	No	Yes	Yes	Yes	Yes	Yes
Albright (2015)	No	Can't tell						Can't tell	Yes	Can't tell	No	No						Yes	Yes	Yes	Yes	Yes					
Benedetti 2015	Yes	Yes	Yes	Yes	Yes	Yes	Yes	Yes	Yes	Can't tell	Can't tell	No						Yes	Yes	Yes	Can’t tell	Yes	Yes	Yes	Yes	Yes	Yes
Berendsen (2015)	Yes	Yes	Yes	Yes	Yes	Yes	Yes	No	Yes	Can't tell	Yes	Can't tell						Yes	Yes	Yes	Yes	Yes	Yes	Yes	Yes	Yes	Yes
Biddle (2017)	Yes	Yes	Yes	Yes	Yes	Yes	Yes	Yes	Yes	No	No	No						Yes	Yes	Yes	Yes	Yes	Yes	Yes	Yes	Yes	Yes
Blunt (2018)	Yes	Yes	Yes	Yes	Yes	Yes	Yes	Yes	Yes	Yes	No	No															
Elramli (2017)	Yes	Yes	Yes	Yes	Yes	Yes	Yes	No	Yes	No	No	No															
Harris (2018)	Yes	Yes	Yes	Yes	Yes	Yes	Yes	Yes	Yes	Yes	No	Yes						Yes	Yes	Yes	Yes	Yes	Yes	Yes	Yes	Yes	Yes
Lakerveld (2012)	Yes	No						Yes	Yes	Yes	No	Yes						Yes	Yes	Yes	Yes	Yes					
Lane (2010)	Yes	Yes						Can't tell	Yes	Yes	No	Yes						Yes	Yes	Yes	Yes	Yes					
Matson (2018)	Yes	Yes	Yes	Yes	Yes	Yes	Yes	Yes	Yes	Yes	Yes	Can't tell															
Matthews (2016)	Yes	Yes	Yes	Yes	Yes	Yes	Yes	Can't tell	Yes	Yes	No	Yes						Yes	Yes	Yes	Yes	Yes	Yes	Yes	Yes	Yes	Yes
Poston (2013)	Yes	Yes	Yes	Yes	No	No	No	Yes	Yes	Yes	Can't tell	Yes						Yes	Yes	Yes	Yes	Yes	Yes	No	No	Can't tell	No
SPH HKU (2017)	Yes	Yes	Yes	Yes	No	No	No	Can't tell	No	Yes	Can't tell	No															
Spittaels (2007)	Yes	Yes						Can't tell	Yes	Yes	Can't tell	Yes						Yes	Yes	Yes	Yes	Yes					
Stathi (2019)	Yes	Yes	Yes	Yes	No	No	No	Can't tell	No	Yes	Can't tell	Yes						Yes	Yes	Yes	Yes	Yes	Yes	No	No	Can't tell	No
Williams (2019)	Yes	Yes	Yes	Yes	No	No	No	Yes	Yes	No	Yes	No						Yes	Yes	Yes	Yes	Yes	Yes	Yes	Yes	No	No

S1. Are there clear research questions? S2. Do the collected data allow to address the research questions? 1.1. Is the qualitative approach appropriate to answer the research question? 1.2. Are the qualitative data collection methods adequate to address the research question? 1.3. Are the findings adequately derived from the data? 1.4. Is the interpretation of results sufficiently substantiated by data? 1.5. Is there coherence between qualitative data sources, collection, analysis and

interpretation? 2.1. Is randomization appropriately performed? 2.2. Are the groups comparable at baseline? 2.3. Are there complete outcome data? 2.3. Are there complete outcome data? 2.4. Are outcome assessors blinded to the intervention provided? 2.5 Did the participants adhere to the assigned intervention? 3.1 Are the participants representative of the target population? 3.2 Are measurements appropriate regarding both the outcome and intervention? 3.3 Are there complete outcome data? 3.4 Are the confounders accounted for in the design and analysis? 3.5 During the study period, is the intervention administered (or exposure occurred) as intended? 4.1. Is the sampling strategy relevant to address the research question? 4.2. Is the sample representative of the target population? 4.3. Are the measurements appropriate? 4.4. Is the risk of nonresponse bias low? 4.5. Is the statistical analysis appropriate to answer the research question? 5.1. Is there an adequate rationale for using a mixed methods design to address the research question? 5.2. Are the different components of the study effectively integrated to answer the research question? 5.3. Are the outputs of the integration of qualitative and quantitative components adequately interpreted? 5.4. Are divergences and inconsistencies between quantitative and qualitative results adequately addressed? 5.5. Do the different components of the study adhere to the quality criteria of each tradition of the methods involved?

For peer review only

Supplementary file 10_search strategies 27th October 2021

Our information specialist updated a few of the MeSH terms that weren't available in the previous searches e.g. sedentary behaviour/ screen time/ and sitting position/.

Database: CINAHL (EBSCOost), search modes - Boolean/Phrase:

S1 (MH "Life Style, Sedentary")

S2 TI (sedentary or sitting or sedentariness or sedentarism)

S3 TX ((sedentary or sitting or seated) N5 (behavio* or lifestyle* or life-style* or pattern* or leisure or time or bout*)))

S4 TX((inactiv* or "no exercise" or nonexercise or "non exercise") N3 (adult* or men or women or male or males or female or females or individual* or people or person or population* or senior or seniors or elderly))

S5 TX((sedentary) N3 (adult* or men or women or male or males or female or females or individual* or people or person or population* or senior or seniors or elderly))

S6 TX ((light or low) N1 "physical activ*")

S7 TX "physical* inactiv*"

S8 TX ("leisure time" N5 ("physical* activ*" or passive or inactiv*))

S9 TX "physical activity level*"

S10 (MH "Sitting")

S11 TX ((sitting or lying) N2 posture*)

S12 TX ((uninterrupted or long* or prolong* or extend* or continu* or protracted or sustain* or period* or duration* or time*) N5 (reclin* or sit or sitting or seated or lying))

S13 TX ("sit less" or "sitting less")

S14 TX ((decrease or reduc* or discourag* or lessen*) N3 (sit or sitting or stand or standing or "physical* inactiv*" or sedentar*))

S15 (MH "Screen Time")

S16 TX (time N5 (computer* or television or tv or "video game*" or videogame* or gaming or screen or media))

S17 TX ((watch* or view*) N5 (television or tv))

S18 TX(play* N5 ("video game*" or videogame* or "computer game*"))

S19 TI ((computer* or television or tv or video game* or videogame* or gaming) and (sedentary or "physical* activity" or sitting or seated or underactiv* or "under activ*")))

S20 MH randomized controlled trials

S21 MH single-blind studies

S22 MH double-blind studies

S23 MH random assignment

S24 MH pretest-posttest design

For peer review only

S25 MH cluster sample

S26 TI (randomised OR randomized)

S27 AB (random*)

S28 TI (trial)

S29 MH (sample size)

S30 AB (assigned OR allocated OR control)

S31 MH (placebos)

S32 PT (randomized controlled trial)

S33 AB (control W5 group)

S34 MH (crossover design)

S35 MH (comparative studies)

S36 AB (cluster W3 RCT)

S37 MH animals+

S38 MH (animal studies)

S39 TI (animal model*)

S40 S37 OR S38 OR S39

S41 MH (human)

S42 S40 not S41

S43 S20 OR S21 OR S22 OR S23 OR S24 OR S25 OR S26 OR S27 OR S28 OR S29 OR S30 OR S31 OR S32 OR S33 OR S34 OR S35 OR S36

S44 S43 NOT S42

S45 (MH "Program Evaluation")

S46 (MH "Process Assessment (Health Care)")

S47 TX "program* evaluat*"

S48 TX ((process evaluat*))

S49 S45 OR S46 OR S47 OR S48

S50 S1 OR S2 OR S3 OR S4 OR S5 OR S6 OR S7 OR S8 OR S9 OR S10 OR S11 OR S12 OR S13 OR S14 OR S15 OR S16 OR S17 OR S18 OR S19

S51 S43 AND S49 AND S50

Database: SPORTDiscus (EBSCOHost), search modes - Boolean/Phrase:

For peer review only

S1 SU Sedentary Lifestyle

S2 TI (sedentary or sitting or sedentariness or sedentarism)

S3 TX ((sedentary or sitting or seated) N5 (behavio* or lifestyle* or life-style* or pattern* or leisure or time or bout*))

S4 TX((inactiv* or "no exercise" or nonexercise or "non exercise") N3 (adult* or men or women or male or males or female or females or individual* or people or person or population* or senior or seniors or elderly))

S5 TX((sedentary) N3 (adult* or men or women or male or males or female or females or individual* or people or person or population* or senior or seniors or elderly))

S6 TX ((light or low) N1 "physical activ*")

S7 TX "physical* inactiv*"

S8 TX ("leisure time" N5 ("physical* activ*" or passive or inactiv*))

S9 TX "physical activity level*"

S10 SU sitting

S11 TX ((sitting or lying) N2 posture*)

S12 TX ((uninterrupted or long* or prolong* or extend* or continu* or protracted or sustain* or period* or duration* or time*) N5 (reclin* or sit or sitting or seated or lying))

S13 TX ("sit less" or "sitting less")

S14 TX ((decrease or reduc* or discourag* or lessen*) N3 (sit or sitting or stand or standing or "physical* inactiv*" or sedentar*))

S15 TX (time N5 (computer* or television or tv or "video game*" or videogame* or gaming or screen or media))

S16 TX ((watch* or view*) N5 (television or tv))

S17 TX(play* N5 ("video game*" or videogame* or "computer game*")) OR AB (play* N5 ("video game*" or videogame* or "computer game*")

S18 TI ((computer* or television or tv or "video game*" or videogame* or gaming) and (sedentary or "physical* activity" or sitting or seated or underactiv* or "under activ*"))

S19 ((DE "RANDOMIZED controlled trials")))

S20 TX "allocat* random*"

S21 TX "random* assign*"

S22 TI (randomised OR randomized)

S23 TI (trial)

S24 AB (assigned OR allocated OR control)

S25 AB (control W5 group)

S26 TX placebo*

S27 TX clinic* n1 trial*

For peer review only

S28 S19 OR S20 OR S21 OR S22 OR S23 OR S24 OR S25 OR S26 OR S27

S29 SU program evaluation

S30 TX "program* evaluat*"

S31 TX "process evaluat*"

S32 S29 OR S30 OR S31

S33 S1 OR S2 OR S3 OR S4 OR S5 OR S6 OR S7 OR S8 OR S9 OR S10 OR S11 OR S12 OR S13 OR S14 OR S15 OR S16 OR S17 OR S18

S34 S28 AND S32 AND S33

Database: Cochrane Database of Systematic Reviews (Wiley):

#1 MeSH descriptor: [Sedentary Behavior] this term only
#2 ((sedentary or sitting or sedentariness or sedentarism)):ti (Word variations have been searched)
#3 ((sedentary or sitting or seated) near/5 (behavio* or lifestyle* or “life style*” or pattern* or leisure or time or bout*)):ti,ab,kw (Word variations have been searched)
#4 ((inactiv* or "no exercise" or nonexercise or "non exercise") near/3 (adult* or men or women or male or males or female or females or individual* or people or person or population* or senior or seniors or elderly)):ti,ab,kw (Word variations have been searched)
#5 (sedentary near/3 (adult* or men or women or male or males or female or females or individual* or people or person or population* or senior or seniors or elderly)):ti,ab,kw (Word variations have been searched)
#6 (((light or low) near/1 "physical activ*")):ti,ab,kw (Word variations have been searched)
#7 ("physical activity level*"):ti,ab,kw
#8 ("physical* inactiv*"):ti,ab,kw
#9 ("leisure time" near/5 ("physical* activ*" or passive or inactiv*)):ti,ab,kw (Word variations have been searched)
#10 MeSH descriptor: [Sitting Position] explode all trees
#11 ((sitting or lying) near/2 posture*):ti,ab,kw
#12 ((uninterrupted or long* or prolong* or extend* or continu* or protracted or sustain* or period* or duration* or time*) near/5 (reclin* or sit or sitting or seated or lying)):ti,ab,kw
#13 (("sit* less" or "sitting less")):ti,ab,kw
#14 ((light or low) near/1 "physical activ*"):ti,ab,kw
#15 ((decrease or reduc* or discourag* or lessen*) near/3 (sit or sitting or stand or standing or "physical* inactiv*" or sedentar*)):ti,ab,kw
#16 MeSH descriptor: [Screen Time] this term only
#17 (time near/5 (computer* or television or tv or "video game*" or videogame* or gaming or screen or media)):ti,ab,kw
#18 ((watch* or view*) near/5 (television or tv)):ti,ab,kw
#19 (play* near/5 ("video game*" or videogame* or computer game*)):ti,ab,kw
#20 ((computer* or television or tv or "video game*" or videogame* or gaming) and (sedentary or "physical* activity*" or sitting or seated or underactiv* or "under activ*")):ti,ab,kw
#21 {or #1-#20}
#22 (("program* evaluation*")):ti,ab,kw
#23 MeSH descriptor: [Outcome and Process Assessment, Health Care] this term only
#24 MeSH descriptor: [Process Assessment, Health Care] this term only
#25 ("process evaluation*"):ti,ab,kw
#26 {or #22-#25}
#27 #21 and #26
#28 #21 and #26 in Cochrane Reviews

Database: Cochrane Central Register of Controlled Trials (Wiley):

#1 MeSH descriptor: [Sedentary Behavior] this term only
#2 ((sedentary or sitting or sedentariness or sedentarism)):ti (Word variations have been searched)
#3 ((sedentary or sitting or seated) near/5 (behavio* or lifestyle* or “life style*” or pattern* or leisure or time or bout*)):ti,ab,kw (Word variations have been searched)

For peer review only

#4 ((inactiv* or "no exercise" or nonexercise or "non exercise") near/3 (adult* or men or women or male or males or female or females or individual* or people or person or population* or senior or seniors or elderly)):ti,ab,kw (Word variations have been searched)
#5 (sedentary near/3 (adult* or men or women or male or males or female or females or individual* or people or person or population* or senior or seniors or elderly)):ti,ab,kw (Word variations have been searched)
#6 (((light or low) near/1 "physical activ*")):ti,ab,kw (Word variations have been searched)
#7 ("physical activity level*"):ti,ab,kw
#8 ("physical* inactiv*"):ti,ab,kw
#9 ("leisure time" near/5 ("physical* activ*" or passive or inactiv*)):ti,ab,kw (Word variations have been searched)
#10 MeSH descriptor: [Sitting Position] explode all trees
#11 ((sitting or lying) near/2 posture*):ti,ab,kw
#12 ((uninterrupted or long* or prolong* or extend* or continu* or protracted or sustain* or period* or duration* or time*) near/5 (reclin* or sit or sitting or seated or lying)):ti,ab,kw
#13 (("sit* less" or "sitting less")):ti,ab,kw
#14 ((light or low) near/1 "physical activ*"):ti,ab,kw
#15 ((decrease or reduc* or discourag* or lessen*) near/3 (sit or sitting or stand or standing or "physical* inactiv*" or sedentar*)):ti,ab,kw
#16 MeSH descriptor: [Screen Time] this term only
#17 (time near/5 (computer* or television or tv or "video game*" or videogame* or gaming or screen or media)):ti,ab,kw
#18 ((watch* or view*) near/5 (television or tv)):ti,ab,kw
#19 (play* near/5 ("video game*" or videogame* or computer game*)):ti,ab,kw
#20 ((computer* or television or tv or "video game*" or videogame* or gaming) and (sedentary or "physical* activity*" or sitting or seated or underactiv* or "under activ*")):ti,ab,kw
#21 {or #1-#20}
#22 (("program* evaluation*")):ti,ab,kw
#23 MeSH descriptor: [Outcome and Process Assessment, Health Care] this term only
#24 MeSH descriptor: [Process Assessment, Health Care] this term only
#25 ("process evaluation*"):ti,ab,kw
#26 {or #22-#25}
#27 #21 and #26
#28 #21 and #26 in Trials

<u>AMED (Allied and Complementary Medicine) (OVID) <1985 to October 2021>:</u>

1 Sedentary Lifestyle/
2 (sedentary or sitting or sedentariness or sedentarism).ti.
3 (sedentary adj3 (adult? or men or women or male or males or female or females or individual? or people or person or population? or senior or seniors or elderly)).tw.
4 ((inactiv* or no exercise or nonexercise or non exercise) adj3 (adult? or men or women or male or males or female or females or individual? or people or person or population? or senior or seniors or elderly)).tw.
5 (sedentary adj3 (adult? or men or women or males or females or individual? or people or population? or senior or seniors or elderly)).tw.
6 ((light or low) adj physical activ*).tw.
7 physical* inactiv*.tw. (248)
8 (leisure time adj5 (physical* activ* or passive or inactiv*)).tw.
9 "physical activity level*".tw.
10 sitting/
11 ((sitting or lying) adj2 posture*).tw.
12 ((uninterrupted or long* or prolong* or extend* or continu* or protracted or sustain* or period* or duration* or time*) adj5 (reclin* or sit or sitting or seated or lying)).tw.
13 (sit less or sitting less).tw.
14 ((decrease or reduc* or discourag* or lessen*) adj3 (sit or sitting or stand or standing or physical* inactiv* or sedentar*)).tw.
15 (time adj5 (computer* or television or tv or video game? or videogame? or gaming or screen or media)).tw.
16 ((watch* or view*) adj5 (television or tv)).tw.
17 (play* adj5 (video game? or videogame? or computer game?)).tw.
18 ((computer* or television or tv or video game? or videogame? or gaming) and (sedentary or physical* activity* or sitting or seated or underactiv* or under activ*)).ti.
19 or/1-18 [sedentary behaviour]
20 process evaluat*.mp.
21 "Outcome and Process Assessment"/
22 program* evaluat*.mp.

For peer review only

23 or/20-22 [process evaluation]
24 19 and 23 [sedentary behaviour and process evaluation]

Database: Embase Classic+Embase (OVID) <1947 to 2021 October 22>:

1 Sedentary Lifestyle/
2 sedentary time/
3 (sedentary or sitting or sedentariness or sedentarism).ti.
4 ((sedentary or sitting or seated) adj5 (behavio* or lifestyle* or life-style* or pattern* or leisure or time or bout*)).tw,kw.
5 ((inactiv* or no exercise or nonexercise or non exercise) adj3 (adult? or men or women or male or males or female or females or individual? or people or person or population? or senior or seniors or elderly)).tw,kw.
6 (sedentary adj3 (adult? or men or women or male or males or female or females or individuals or people or person or population? or senior or seniors or elderly)).tw,kw.
7 physical* inactiv*.tw,kw.
8 (leisure time adj5 (physical* activ* or passive or inactiv*)).tw,kw.
9 physical activity level*.tw,kw.
10 ((sitting or lying) adj2 posture*).tw,kw.
11 sitting/
12 ((uninterrupted or long* or prolong* or extend* or continu* or protracted or sustain* or period* or duration* or time*) adj2 (reclin* or sit or sitting or seated or lying)).tw,kw.
13 (sit less or sitting less).tw,kw.
14 ((light or low) adj physical activ*).tw,kw.
15 ((decrease or reduc* or discourag* or lessen*) adj3 (sit or sitting or stand or standing or physical* inactiv* or sedentar*)).tw,kw.
16 screen time/
17 (time adj5 (computer* or television or tv or video game? or videogame? or gaming or screen or media)).tw,kw.
18 ((watch* or view*) adj5 (television or tv)).tw.
19 (play* adj5 (video game? or videogame? or computer game?)).tw,kw.
20 ((computer* or television or tv or video game? or videogame? or gaming) and (sedentary or physical* activity* or sitting or seated or underactiv* or under activ*)).ti.
21 or/1-20 [sedentary behaviour]
22 Randomized controlled trial/
23 Controlled clinical study/
24 22 or 23
25 Random*.tw.
26 randomization/
27 intermethod comparison/
28 placebo.tw.
29 (compare or compared or comparison).ti.
30 ((evaluated or evaluate or evaluating or assessed or assess) and (compare or compared or comparing or comparison)).ab.
31 (open adj label).tw.
32 ((double or single or doubly or singly) adj (blind or blinded or blindly)).tw.
33 double blind procedure/
34 parallel group*1.tw.
35 (crossover or cross over).tw.
36 ((assign* or match or matched or allocation) adj5 (alternate or group*1 or intervention*1 or patient*1 or subject*1 or participant*1)).tw.
37 (assigned or allocated).tw.
38 (controlled adj7 (study or design or trial)).tw.
39 (volunteer or volunteers).tw.
40 human experiment/
41 trial.ti.
42 or/25-41
43 42 or 24
44 (random* adj sampl* adj7 ("cross section*" or questionnaire*1 or survey* or database*1)).tw. not (comparative study/ or controlled study/ or randomi?ed controlled.tw. or randomly assigned.tw.)
45 Cross-sectional study/ not (randomized controlled trial/ or controlled clinical study/ or controlled study/ or randomi?ed controlled.tw. or control group*1.tw.)
46 (((case adj control*) and random*) not randomi?ed controlled).tw.
47 (Systematic review not (trial or study)).ti.
48 (nonrandom* not random*).tw.
49 "Random field*".tw.

For peer review only

50 (random cluster adj3 sampl*).tw.
51 (review.ab. and review.pt.) not trial.ti.
52 "we searched".ab. and (review.ti. or review.pt.)
53 "update review".ab.
54 (databases adj4 searched).ab.
55 (rat or rats or mouse or mice or swine or porcine or murine or sheep or lambs or pigs or piglets or rabbit or rabbits or cat or cats or dog or dogs or cattle or bovine or monkey or monkeys or trout or marmoset*1).ti. and animal experiment/
56 Animal experiment/ not (human experiment/ or human/)
57 or/44-56
58 43 not 57 [Cochrane Highly Sensitive Search Strategy for identifying controlled trials in Embase: (2018 revision); Ovid format (Glanville et al 2019b) Validated Search Filter]
59 program evaluat*.mp.
60 health care quality/
61 process evaluat*.mp.
62 or/59-61 [process evaluation]
63 21 and 58 and 62

<u>Database: APA PsycInfo (OVID) <1806 to October Week 3 2021>:</u>

1 Sedentary behavior/
2 (sedentary or sitting or sedentariness or sedentarism).ti.
3 ((sedentary or sitting or seated) adj5 (behavio* or lifestyle* or life-style* or pattern* or leisure or time or bout*)).tw.
4 ((inactiv* or no exercise or nonexercise or non exercise) adj3 (adult? or men or women or male or males or female or females or individual? or people or person or population? or senior or seniors or elderly)).tw.
5 (sedentary adj3 (adult? or men or women or male or males or female or females or individual? or people or person or population? or senior or seniors or elderly)).tw.
6 ((light or low) adj physical activ*).tw.
7 physical* inactiv*.tw.
8 (leisure time adj5 (physical* activ* or passive or inactiv*)).tw.
9 "physical activity level*".tw.
10 ((sitting or lying) adj2 posture*).tw.
11 ((uninterrupted or long* or prolong* or extend* or continu* or protracted or sustain* or period* or duration* or time*) adj5 (reclin* or sit or sitting or seated or lying)).tw.
12 (sit less or sitting less).tw.
13 ((decrease or reduc* or discourag* or lessen*) adj3 (sit or sitting or stand or standing or physical* inactiv* or sedentar*)).tw.
14 screen time/
15 (time adj5 (computer* or television or tv or video game? or videogame? or gaming or screen or media)).tw.
16 ((watch* or view*) adj5 (television or tv)).tw.
17 (play* adj5 (video game? or videogame? or computer game?)).tw.
18 ((computer* or television or tv or video game? or videogame? or gaming) and (sedentary or physical* activity* or sitting or seated or underactiv* or under activ*)).ti.
19 or/1-18 [sednetary behaviour]
20 clinical trials/ or treatment effectiveness evaluation/ or placebo/
21 (random* or RCT or RCTs).tw.
22 (clinical* adj5 trial*).tw.
23 ((control or treatment or experiment* or intervention) adj5 (group* or subject* or patient*)).tw.
24 ((control or experiment* or conservative) adj5 (treatment or therapy or procedure or manage*)).tw.
25 ((singl* or doubl* or tripl* or trebl*) adj5 (blind* or mask*)).tw.
26 (cross over or crossover).tw.
27 (placebo* or sham).tw.
28 rial.ti.
29 (assign* or allocat*).tw.
30 controls.tw.
31 or/20-30 [RCTs]
32 program evaluat*.mp.
33 process evaluat*.mp.
34 evaluation/
35 or/32-34 [process evaluation terms]
36 19 and 31 and 35 [sedentary behaviour and RCTs and process evaluations]

For peer review only

Database: Ovid MEDLINE(R) All <1946 to October 22, 2021>:

1 Sedentary behavior/
2 (sedentary or sitting or sedentariness or sedentarism).ti.
3 ((sedentary or sitting or seated) adj5 (behavio* or lifestyle* or life-style* or pattern* or leisure or time or bout*)).tw,kf.
4 ((inactiv* or no exercise or nonexercise or non exercise) adj3 (adult? or men or women or male or males or female or females or individual? or people or person or population? or senior or seniors or elderly)).tw,kf.
5 (sedentary adj3 (adult? or men or women or male or males or female or females or individual? or people or person or population? or senior or seniors or elderly)).tw,kf.
6 physical* inactiv*.tw,kf.
7 (leisure time adj5 (physical* activ* or passive or inactiv*)).tw,kf.
8 physical activity level*.tw,kf.
9 sitting position/
10 ((sitting or lying) adj2 posture*).tw,kf.
11 ((uninterrupted or long* or prolong* or extend* or continu* or protracted or sustain* or period* or duration* or time*) adj5 (reclin* or sit or sitting or seated or lying)).tw,kf.
12 (sit less or sitting less).tw,kf.
13 ((light or low) adj "physical activ*").tw,kf.
14 ((decrease or reduc* or discourag* or lessen*) adj3 (sit or sitting or stand or standing or physical* inactiv* or sedentar*)).tw,kf.
15 screen time/
16 (time adj5 (computer* or television or tv or video game? or videogame? or gaming or screen or media)).tw,kf.
17 ((watch* or view*) adj5 (television or tv)).tw,kf.
18 (play* adj5 (video game? or videogame? or computer game?)).tw,kf.
19 ((computer* or television or tv or video game? or videogame? or gaming) and (sedentary or physical* activity* or sitting or seated or underactiv* or under activ*)).ti.
20 or/1-19 [sedentary behaviour]
21 program* evaluat*.mp.
22 "Outcome and Process Assessment (Health Care)"/
23 "Process Assessment (Health Care)"/
24 process evaluat*.mp.
25 or/21-24 [process evaluation]
26 randomized controlled trial.pt.
27 controlled clinical trial.pt.
28 randomized.ab.
29 placebo.ab.
30 drug therapy.fs.
31 randomly.ab.
32 trial.ab.
33 groups.ab.
34 26 or 27 or 28 or 29 or 30 or 31 or 32 or 33
35 exp animals/ not humans.sh.
36 34 not 35 [Cochrane RCT filter 2008, sensitivity maximimising]
37 20 and 25 and 36 [sedentary behaviour and process evaluation and RCTs]

Database: Web of Science: Indexes=SCI-EXPANDED, SSCI, CPCI-S, CPCI-SSH, ESCI (Clarivate), Timespan= 1900-2021:

1 TI=((sedentary or sitting or sedentariness or sedentarism))

2 TS=(((sedentary or inactiv* or "no exercise" or nonexercise or "non exercise") near/3 (adult* or men or women or male or males or female or females or individual* or people or person or population* or senior or seniors or elderly)))

3 TS=(((sedentary or sitting or seated) near/5 (behavio* or lifestyle* or "life style*" or pattern* or leisure or time or bout*)))

4 TS=((light or low*) near/1 "physical activ*")

5 TS=("physical* inactiv*")

# 6	TS=("leisure time" near/5 ("physical* activ*" or passive or inactiv*))
# 7	TS=("physical activity level*")
# 8	TS=((sitting or lying) near/2 posture)
# 9	TS=(((uninterrupted or long* or prolong* or extend* or continu* or protracted or sustain* or period* or duration* or time*) near/5 (reclin* or sit or sitting or seated or lying)))
# 10	TS=("sit less" or "sitting less")
# 11	TS=(((decrease or reduc* or discourag* or lessen*) near/3 (sit or sitting or stand or standing or "physical* inactiv*" or sedentar*)))
# 12	TS=((time*) near/3 (computer* or television or tv or "video game*" or videogame* or gaming or screen or media))
# 13	TS=(((watch* or view*) near/5 (television or tv)))
# 14	TS=((play* near/5 ("video game*" or videogame* or "computer gam*")))
# 15	TI=(((computer* or television or tv or "video game*" or videogame* or gaming) and (sedentary or "physical* activit*" or sitting or seated or underactiv* or "under activ*")))
# 16	#15 OR #14 OR #13 OR #12 OR #11 OR #10 OR #9 OR #8 OR #7 OR #6 OR #5 OR #4 OR #3 OR #2 OR #1
# 17	TS=((random* or RCT or placebo or clinical Near/1 trial*))
# 18	TS=(("program* evaluat*"))
# 19	TS=(("process evaluat*"))
# 20	#19 OR #18
# 21	#20 AND #17 AND #16

For peer review only
